# A practical flow synthesis of 1,2,3-triazoles†

**DOI:** 10.1039/d2ra04727f

**Published:** 2022-10-11

**Authors:** Dawid Drelinkiewicz, Richard J. Whitby

**Affiliations:** School of Chemistry, Faculty of Engineering and Physical Sciences, The University of Southampton Southampton UK rjw1@soton.ac.uk

## Abstract

A robust and versatile protocol for synthesis of 1-monosubstituted and 1,4-disubstituted 1*H*-1,2,3-triazoles was established under continuous flow conditions using copper-on-charcoal as a heterogeneous catalyst. This methodology allowed for the synthesis of a diverse set of substituted 1,2,3-triazoles with good functional group tolerance and high yields. 2-Ynoic acids were also used as small-chain alkyne donors in a decarboxylation/cycloaddition cascade, allowing gaseous reagents to be bypassed, delivering desired triazoles in high yields. The developed methodology was used to synthesize an antiepileptic agent, rufinamide, which was obtained in 96% isolated yield.

## Abstract 2

Copper-on-charcoal is an excellent heterogeneous catalyst for the alkyne–azide cycloaddition reaction performed under continuous flow conditions. 2-Ynoic acids undergo decarboxylation/cycloaddition cascade giving triazoles bearing small alkyl chains.

## Graphical abstract

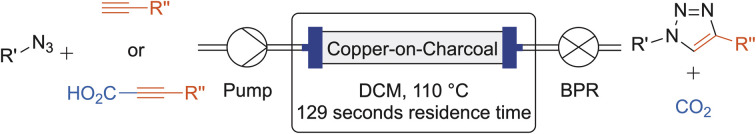

## Introduction

**Chart 1:**
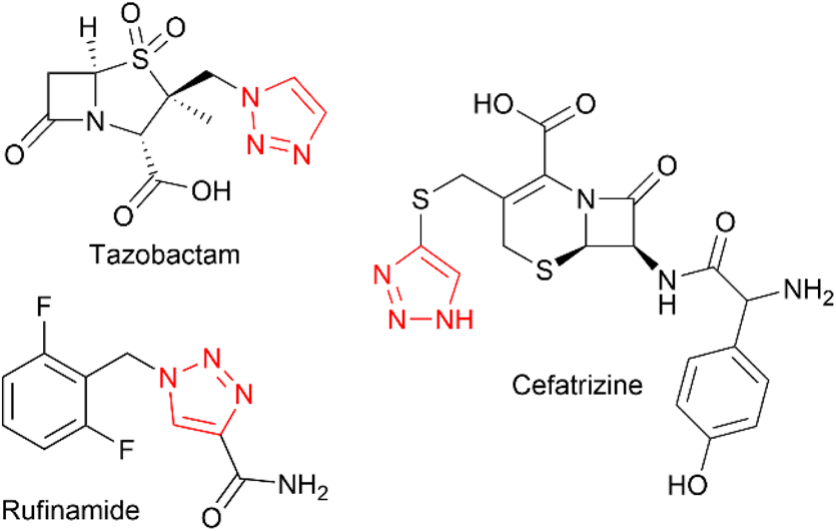
Example bioactive molecules containing 1,2,3-triazole moiety.

The 1,2,3-triazole moiety is of great importance in the fields of chemistry and chemical biology, due to its unique properties, inert nature, and ability to mimic amide bonds.^1^ This motif is very often seen in experimental drug candidates and approved drugs, such as tazobactam, cefatrizine or rufinamide (Chart 1).

The copper catalysed 1,3-dipolar Huisgen cycloaddition of azides to alkynes, known as the CuAAC reaction, reported in 2002 by the groups of Meldal^2^ and Sharpless^3^ revolutionised the field of triazole synthesis allowing for formation of these structures using a reliable, regioselective and high-yielding process. It became the protypical example of a ‘click’ reaction as defined by Sharpless.^4^ The CuAAC reaction has seen applications in many areas, including bioconjugation chemistry,^5–8^ dendrimer and polymer synthesis,^9–12^ synthesis of peptidomimetics,^13,14^ combinatorial drug chemistry,^15–18^ and materials science.^19–26^

Although many homogenous catalytic systems have been developed^27^ our particular need was a for a process which could operate under continuous flow conditions, so a heterogeneous catalyst was desired. One of the first developed for the CuAAC reaction was copper(i) iodide coordinated to a dimethylaminomethyl moiety in Amberlyst A-21 resin.^28^ Unfortunately, high metal leaching limits its use, though can be ameliorated using Quadrapure™ TU scavenging resin. Aminopropyl-silica-supported copper nanoparticles gave excellent results in the CuAAC reaction, with low copper leaching,^29^ but its synthesis using a metal vapour reactor inhibits adoption. A flow copper capillary reactor was reported in a single click reaction delivering rufinamide in modest yield and high productivity,^30^ but a CNC and 3D metal printing technique is required to construct it, limiting its applicability. Alternatively, inductively heated copper wire,^31,32^ or copper tubes^33,34^ were reported to catalyse the CuAAC reaction using high temperatures. The use of copper powder is convenient and reported to work well,^35^ but we were unable to obtain useful activity even when activating the surface of copper to copper oxide or copper iodide (see ESI†). In 2006, Lipshutz and Taft reported the use of copper-on-charcoal, which can be synthesized in a simple process from cheap and commercially available materials and was an excellent catalyst for the CuAAC batch reaction.^36^ An elegant study in 2014 by Buckley *et al.*^37^ indicated that copper(ii) hydroxynitrate known as Gerhardite is the catalytically active species. In 2010, Kappe *et al.*^38^ used copper-on-charcoal in a flow experiments for a simple example of the CuAAC reaction demonstrating excellent yields. Although mechanistic studies indicated a homogenous mechanism of operation, leaching of copper was low. Notably, no additive or base was used in the reaction. Given the convenient preparation of copper-on-charcoal we chose to investigate its scope for the continuous flow CuAAC synthesis of various 1-monosubstituted and 1,4-disubstituted-1*H*-1,2,3-triazoles.

## Results and discussion

### Optimisation of reaction conditions

Copper-on-charcoal was synthesized accordingly to the original procedure published by Lipshutz and Taft in 2006^36^ and stored in a closed box under normal air atmosphere. The catalytic activity just after the synthesis of Cu/C and after 12 months was the same demonstrating excellent shelf storage life. The synthesis of 1,4-diphenyl-1*H*-1,2,3-triazole (3a) from phenyl azide (1a) and phenylacetylene (2a) was chosen for optimisation studies (Table 1). Dichloromethane (DCM) was chosen as the main solvent as it provided good solubility for the wide range of triazoles formed and did not interfere with in-line monitoring by IR. At the temperature used its vapour pressure is around 7 bar, well withing the capabilities of the flow system and back-pressure regulator used. Other solvents such as tetrahydrofuran, 2-methyl tetrahydrofuran, acetone and toluene worked but much lower concentrations needed to be used to avoid blockage of the flow system. Solvents such as dimethylformamide and dimethylsulfoxide provided reasonable solubility, but interfered with IR monitoring and were more difficult to remove from the products during work-up.

**Table 1:** Optimisation of the CuAAC reaction conditions

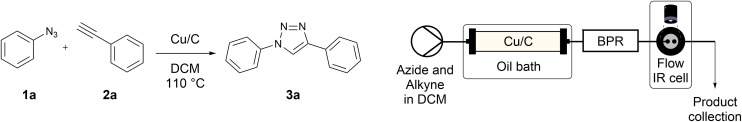						
Entry	Azide [M]	Alkyne [M]	Temp [°C]	Residence time^a^ [s]	IR yield^b^ [%]	Isolated yield [%]
1	0.100	0.117	70	65	14	15
2			80	65	22	23
3			90	65	30	28
4			90	97	39	43
5		0.125	90	97	44	47
6			100	387	68	65
7			110	387	>90	86
8			110	97	>90	92
9		0.130	110	97	>90	94
10			110	129	>90	100

a Residence time was estimated based on flow rates and reactor volume.

b Above 90% reaction yield saturation of IR absorption measurement was observed.

A simple flow reaction setup with a Vapourtec R2+ unit delivering the premixed reagents to the catalytic column filled with copper-on-charcoal followed by an IR measurement cell for *in situ* reaction monitoring was used. All performed reactions were done on 1.0 mmol scale using the same catalytic column filled with 860 mg of Cu/C. Azide concentration was set to 0.1 M, as further increase could result in blockages in the flow system due to triazole precipitation. An optimal reaction conditions allowing for quantitative formation of desired 1,4-diphenyl-1*H*-1,2,3-triazole were achieved at 110 °C and approximately 129 seconds residence time using DCM as a solvent with azide and alkyne concentrations of 0.10 M and 0.13 M, respectively.

### Catalyst productivity determination under optimised conditions

**Fig. 1:**
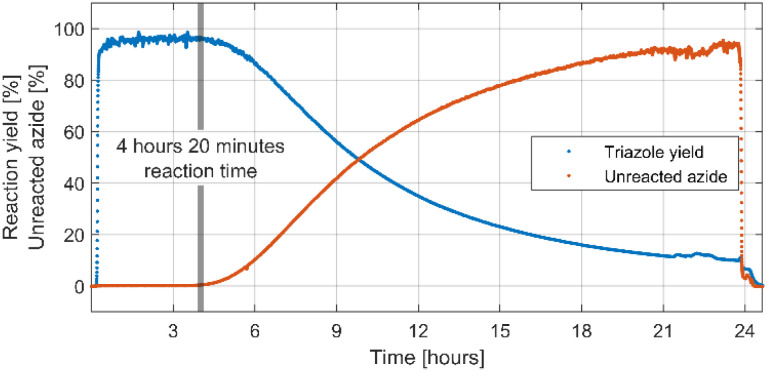
Triazole yield derived from IR absorbance of triazole at 1033 cm^−1^ and amount of unreacted azide derived from IR absorbance of azide at 2062 cm^−1^ in the 24 hours long experiment. Note that, although the spectroscopic triazole yield is less than 100% in first 4 hours and 20 minutes of experiment, it is due to the spectrometer saturation.

After successful optimisation a 24 hours-long continuous processing of phenyl azide and phenylacetylene using optimised reaction conditions was performed to obtain information on catalyst lifetime. Analysis of IR spectra obtained from in-line reaction monitoring (Fig. 1) showed that for the first 4 hours and 20 minutes of experiment yield and conversion was steady and quantitative, but after that time a decrease was observed reaching 10% yield after 24 hours of continuous processing. A preparative 4 hours-long experiment was then performed using a new catalyst column giving 1,4-diphenyl-1*H*-1,2,3-triazole not contaminated with starting materials in 96.2% isolated yield. A simple work-up procedure consisting of removal of solvent *in vacuo*, washing of obtained residue with cold water and cold hexane then drying afforded desired off-white solid. This process delivered 3.83 grams of triazole product over 4 hours elution time resulting in productivity of 957.5 mg h^−1^ or 4.33 mmol h^−1^. As judged from in line IR monitoring reaction proceeded with quantitative conversion throughout the whole experiment.

### Methodology scope evaluation

**Chart 2:**
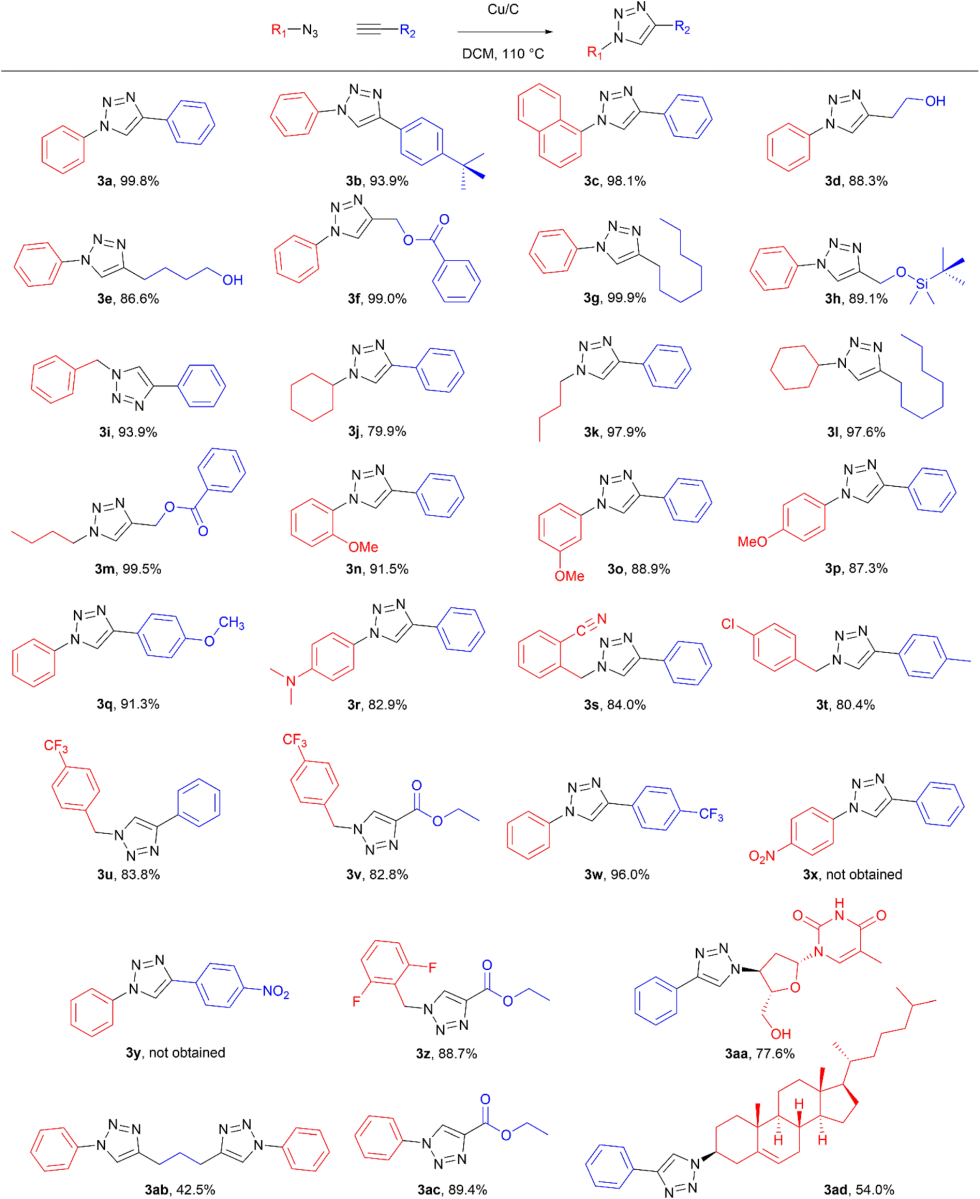
Scope of the copper-on-charcoal catalysed CuAAC reaction in continuous flow system. Reported yields are isolated yields.

To evaluate the scope of the developed methodology, a set of azides and a set of alkynes were tested in the flow setup under the optimised reaction conditions‡‡Synthesis of 1,4-diphenyl-1*H*-1,2,3-triazole (3a) using Cu/C catalysed flow click reaction as a general procedure example: flow stream containing 0.10 M solution of phenyl azide (1.0 eq.) and 0.13 M solution of phenylacetylene (1.3 eq.) in DCM was directed with 0.75 mL min^−1^ to the catalytic column (stainless steel Restek 4.6 mm ID × 150 mm column filled with 860 mg Cu/C, total volume of 2.49 mL, effective volume of 1.61 mL, 1.01 mmol Cu per 1.0 g of Cu/C, 0.869 mmol Cu) submerged in an oil bath at 110 °C. Additional cooling coil (1.0 m, 1.00 mm ID, stainless steel) submersed in water (at approx. 23 °C) was placed after the reactor to cool down reaction mixture. IR measurement cell was placed after the BPR (250 psi). Flow equipment was controlled with python script. Reaction mixture was passed through Cu/C catalytic columns for 800 s resulting in elution of 10.0 mL of reaction mixture, which was collected, solvent was then removed and obtained solid residue was preadsorbed onto silica and purified by column chromatography (SiO_2_; *n*-hexane : AcOEt 9 : 1 to 5 : 5, *R*_f_ = 0.15 in 10% AcOEt in *n*-hexane) and was isolated as a white solid, 220.8 mg (99.8% yield). Mp: 184.0–185.0 °C, lit. 183–184 °C;^53^^1^H NMR (400 MHz, CDCl_3_) *δ* (ppm) = 8.20 (s, 1H), 7.92 (m, 2H), 7.80 (m, 2H), 7.56 (m, 2H), 7.50–7.44 (m, 3H), 7.38 (m, 1H). ^13^C NMR (101 MHz, CDCl_3_) *δ* (ppm) = 148.6, 137.3, 130.4, 130.0, 129.1, 128.9, 128.6, 126.0, 120.7, 117.7. in which various 1,2,3-triazoles containing aliphatic and/or aromatic substituents were obtained in a high to quantitative yields (Chart 2). Purification on a short pad of silica using ethyl acetate : *n*-hexane afforded the desired 1,4-disubstituted-1*H*-1,2,3-triazoles in high purity. Both aromatic and aliphatic alkynes and azides worked well, and there was good functional group tolerance. When 1,6-heptadiyne was used as an alkyne donor, stoichiometry of the reaction was changed to 1.0 : 0.7 of azide and alkyne, respectively, to allow for formation of the bis addition product (3ab). The flow rate was reduced to give a longer reaction time equal to 242 seconds and the bis-triazole was obtained in 42.5% yield. Traces of monocyclized product were also detected. Further increase of temperature (to 120 °C) and residence time (to 323 seconds) slightly increased the reaction yield and delivered desired bicyclization product in 54.8% yield. Although most of the reactions proceeded smoothly, triazoles containing 4-nitrophenyl moiety (3x and 3y) at either the 1- or 4-position precipitated in the reaction column and only small amounts could be isolated before the system blocked. Lower concentrations (down to 0.005 M) and alternative solvents (methanol, acetone, DMSO) did not solve the problem. The reaction worked well using batch conditions (see ESI†). Similarly, when 1-ethynyl-4-(trifluoromethyl)benzene was reacted with phenyl azide, precipitation of product 3w occurred resulting in flow system blockage. Further four-fold dilution of reaction mixture allowed for the successful synthesis of the triazole 3w. Click reactions with azides of natural products, azidothymidine and 3β-azido-5-cholestene delivered desired triazoles 3aa and 3ad in 77.6% and 54.0% yields, respectively. Note that reaction utilizing azidothymidine was performed using ethanol as solvent, due to low solubility of azidothymidine in DCM.

### Application of 2-ynoic acids as terminal alkyne donors

When propiolic acid (2b) was used as the alkyne moiety in the cyclization reaction an *in situ* copper-assisted decarboxylation occurred to give 1-monosubstituted triazoles (Table 2, 3ae and 3af). Gas bubbles, presumably CO_2_, were observed in the flow system after the back-pressure regulator. Even at lower reaction temperatures, (50 °C, 6% conversion), only decarboxylated product was detected in the reaction mixture. Passing the 1-(2,6-difluorobenzyl)-1*H*-1,2,3-triazole-4-carboxylic acid through the Cu/C column under our standard conditions resulted only in recovery of the carboxylic acid, confirming that decarboxylation step precedes the cycloaddition reaction. The reaction extended to the terminally substituted propiolic acids 2c and 2d to give 1,4-disubstituted triazoles (Table 2, 3ag, 3ah, and 3ai) where decarboxylation must precede cycloaddition. The use of propiolic acids instead of gaseous small-chain alkynes, which require special equipment for performing a liquid–gaseous reaction in flow systems, is a great advantage. The decarboxylation/click cascade reaction with propiolic acid has been reported using the sodium ascorbate/copper iodide reductive catalytic system with addition of bases such as CsCO_3_ and DBU.^39,40^ Higher 2-ynoic acids have also been used, although they required microwave irradiation or presence of additives such as l-proline and K_2_CO_3_.^41,42^

**Table 2:** Scope of the Cu/C catalysed flow click reaction utilizing 2-ynoic acids and trimethylsilylacetylene as alkyne donors under optimised conditions

Azide	Alkyne	Triazole	Yield [%]
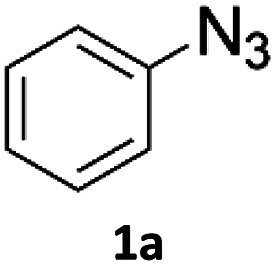	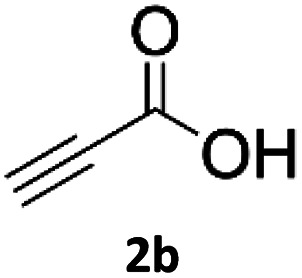	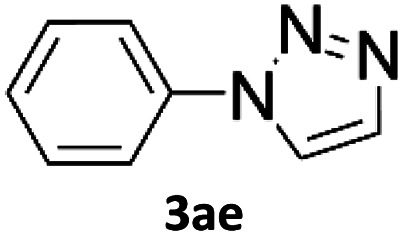	98.6
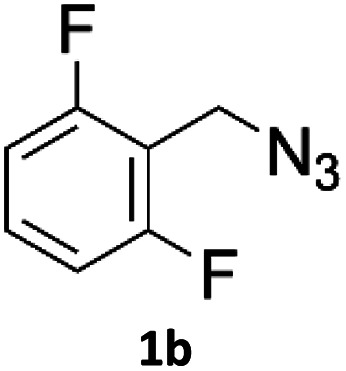	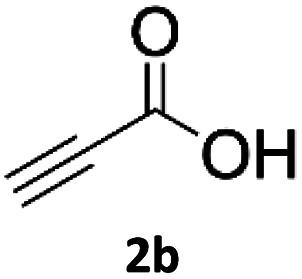	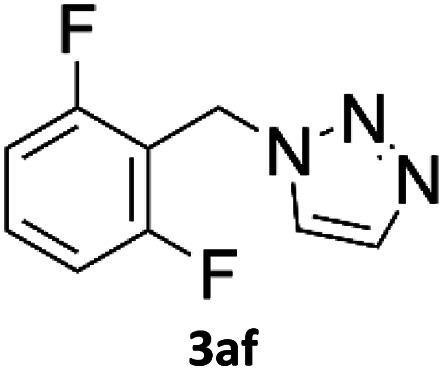	96.4
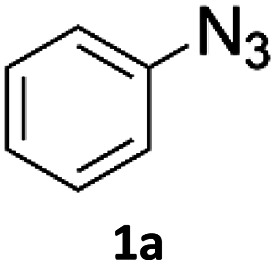	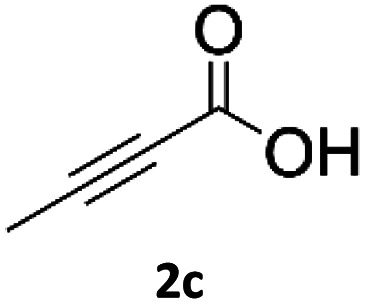	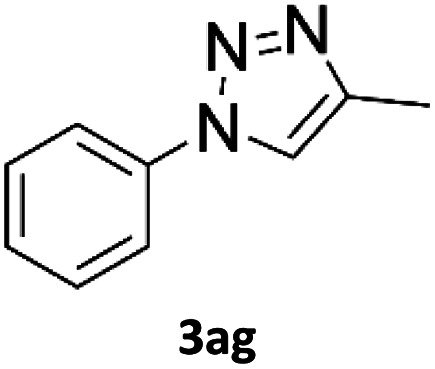	82.3
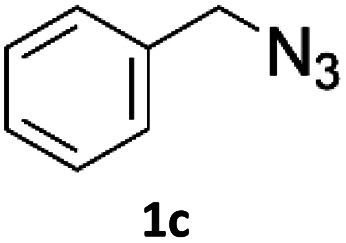	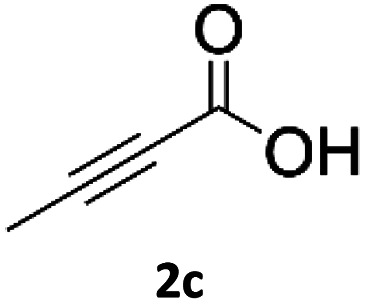	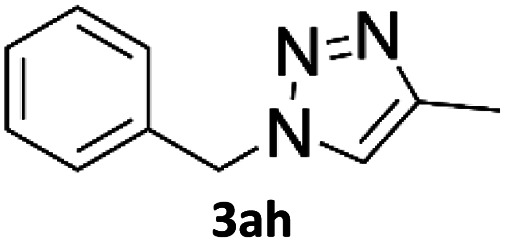	82.2
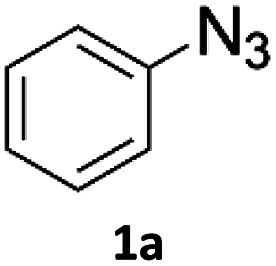	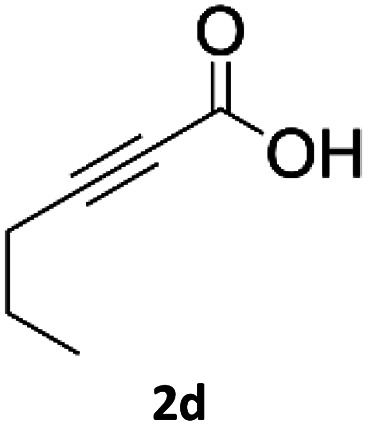	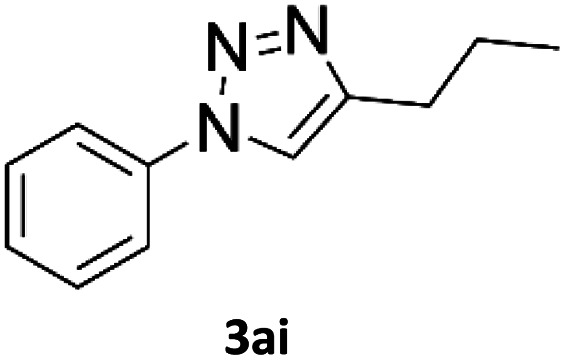	83.0
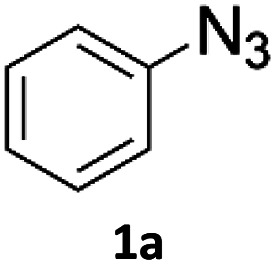	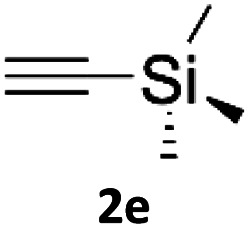	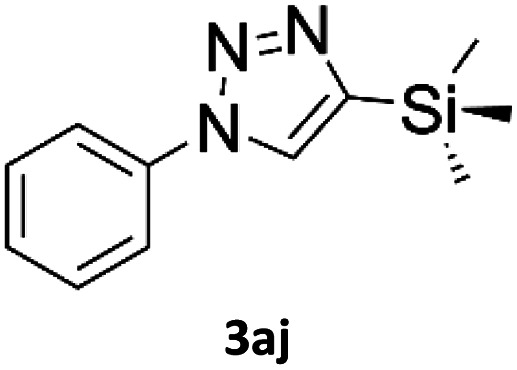	9.2
		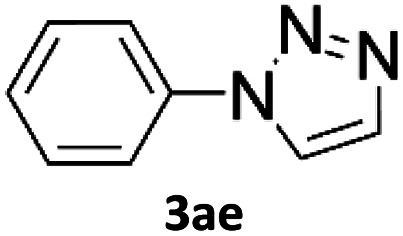	75.4

In comparison with other methods reported before^39–42^ using 2-ynoic acids, no additives are required in our method allowing a simple work-up consisting of removal of solvent with subsequent washing of the obtained solid to deliver 1,2,3-triazoles in a high purity. Utilising trimethylsilylacetylene (Table 2, 2e) under our standard conditions gave TMS-substituted triazole (3aj, 9% isolated yield) and desilylated 1-phenyl-1*H*-1,2,3-triazole (3ae, 75% isolated yield).^43–45^ With lower temperatures (from 50 °C to 90 °C) and higher residence time (323 seconds) reactions delivered mixtures of products in which both regioisomers of trimethylsilylacetylene cycloaddition and the desilylated 1-phenyl-1*H*-1,2,3-triazole (3ae) were formed.

### Synthesis of API as an application example

As an example of potential application of the developed methodology 1-(2,6-difluorobenzyl)-1*H*-1,2,3-triazole-4-carboxyamide (4), also known as Rufinamide was synthesized. Rufinamide is an anticonvulsant medication used to treat various seizure disorders^46^ and Lennox-Gastaut syndrome,^47^ and was originally researched by Novartis Pharma, AG and first approved by FDA in 2008.

The direct formation of Rufinamide by the CuAAC reaction between 2,6-difluorobenzyl azide and propiolamide in batch has been reported using various conditions and catalysts.^48–51^ Its synthesis under flow conditions from propiolamide, formed in-line from ethyl propiolate, using copper tubing was reported by Jamison *et al.*^52^ with 92% yield and 20 : 1 1,4- to 1,5-regioselectivity.

**Scheme 1:**
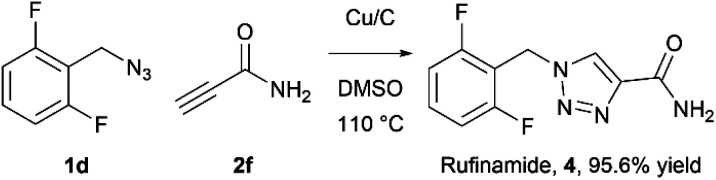
Synthesis of rufinamide in Cu/C catalysed click flow reaction.

The rufinamide synthesis in flow using copper-on-charcoal as catalyst was performed using difluorobenzyl azide (1d) and propiolamide (2f) (Scheme 1.) under optimised conditions, only replacing DCM with DMSO, to allow for complete dissolution of propiolamide. Rufinamide was obtained in 95.6% yield as single 1,4-regioisomer, after simple removal of solvent *in vacuo*, washing of obtained solid with water to remove impurities and drying under vacuum.

## Conclusions

In summary, a new and versatile protocol was developed for the synthesis of 1-mono and 1,4-disubstituted 1,2,3-triazoles in a flow system using copper-on-charcoal as a cheap and robust catalyst, which delivered desired products in high yields and good functional group tolerance. A great practical advantage is that the reaction does not require added base. Moreover, 24 hours long continuous experiment between phenyl azide and phenylacetylene delivered 17.3 mmol (96.2% isolated yield) of 1,4-diphenyl-1*H*-1,2,3-triazole over first 4 hours of reaction time without any drop in conversion or yield. 1,2,3-triazoles bearing small alkyl group at 4-position were also obtained using 2-ynoic acids as precursors for decarboxylation/cycloaddition cascade allowing for bypassing the usage of flammable and dangerous small terminal alkynes and expensive gas–liquid flow equipment. As an example of potential application of developed protocol, rufinamide, an antiepileptic API, was synthesized in 95.6% isolated yield after a simple work-up without the use of chromatographic methods.

## Author contributions

D. Drelinkiewicz: conceptualization, formal analysis, investigation, methodology, validation, visualization, writing – original draft, writing – review & editing. R. J. Whitby: conceptualization, funding acquisition, resources, supervision, writing – review & editing.

## Conflicts of interest

There are no conflicts to declare.

## Supplementary Material

RA-012-D2RA04727F-s001
