# Supplementary material for: A practical flow synthesis of 1,2,3-triazoles
Source: RSC Adv. 2022 Oct 11;12(45):28910–5. doi: 10.1039/d2ra04727f (PMC9551675; doi:10.1039/d2ra04727f)
Supplement: RA-012-D2RA04727F-s001 [file RA-012-D2RA04727F-s001.pdf]

Electronic Supplementary Material (ESI) for RSC Advances.
This journal is © The Royal Society of Chemistry 2022

A Practical Flow Synthesis of 1,2,3-Triazoles

Dawid Drelinkiewicz[a], Richard J. Whitby[a]

[a] School of Chemistry, Faculty of Engineering and Physical Sciences, The University of Southampton, University Road, Southampton, SO17 1BJ, UK

Supporting Information

Table of Contents

1. General methods: 3

2. Experimental Procedures: 4

Procedure A: Synthesis of Copper-on-Charcoal[21]: 4

Procedure B: General procedure for Copper-on-Charcoal catalysed CuAAC reaction performed in flow system: 4

Procedure C: Calibration of IR spectrometer: 5

3. Trials employing metallic copper powder as CuAAC reaction catalyst: 6

Procedure D: Tests of metallic copper as CuAAC reaction catalysts: 6

Procedure E: Activation of metallic copper surface with iodine: 10

Procedure F: Activation of metallic copper surface with hydrogen peroxide: 10

4. Procedures, Purification and Characterization Data of 1*H*-1,2,3-Triazoles: 13

1,4-Diphenyl-1*H*-1,2,3-triazole (3a): 13

1-Phenyl-4-(4-*tert*-butylphenyl)-1*H*-1,2,3-triazole (3b): 13

1-(Naphthalen-1-yl)-4-phenyl-1*H*-1,2,3-triazole (3c): 13

2-(1-Phenyl-1*H*-1,2,3-triazol-4-yl)ethan-1-ol (3d): 13

4-(1-Phenyl-1*H*-1,2,3-triazol-4-yl)butan-1-ol (3e): 14

(1-Phenyl-1*H*-1,2,3-triazol-4-yl)methyl benzoate (3f): 14

4-Octyl-1-phenyl-1*H*-1,2,3-triazole (3g): 14

4-(((*tert*-Butyldimethylsilyl)oxy)methyl)-1-phenyl-1*H*-1,2,3-triazole (3h): 14

1-Benzyl-4-phenyl-1*H*-1,2,3-triazole (3i): 15

1-Cyclohexyl-4-phenyl-1*H*-1,2,3-triazole (3j): 15

1-Butyl-4-phenyl-1*H*-1,2,3-triazole (3k): 15

1-Cyclohexyl-4-octyl-1*H*-1,2,3-triazole (3l): 15

(1-Butyl-1*H*-1,2,3-triazol-4-yl)methyl benzoate (3m): 16

1-(2-Methoxyphenyl)-4-phenyl-1*H*-1,2,3-triazole (3n): 16

1-(3-Methoxyphenyl)-4-phenyl-1*H*-1,2,3-triazole (3o): 16

1-(4-Methoxyphenyl)-4-phenyl-1*H*-1,2,3-triazole (3p): 16
1-Phenyl-4-(4-methoxyphenyl)-1*H*-1,2,3-triazole (3q): 17
N,N-Dimethyl-4-(4-phenyl-1H-1,2,3-triazol-1-yl)aniline (3r): 17
2-((4-Phenyl-1H-1,2,3-triazol-1-yl)methyl)benzonitrile (3s): 17
1-(4-Chlorobenzyl)-4-(p-tolyl)-1H-1,2,3-triazole (3t): 17
4-Phenyl-1-(4-(trifluoromethyl)benzyl)-1H-1,2,3-triazole (3u): 18
Ethyl 1-(4-(trifluoromethyl)benzyl)-1H-1,2,3-triazole-4-carboxylate (3v): 18
1-Phenyl-4-(4-(trifluoromethyl)phenyl)-1H-1,2,3-triazole (3w): 18
1-(4-Nitrophenyl)-4-phenyl-1*H*-1,2,3-triazole (3x): 18
1-Phenyl-4-(4-nitrophenyl)-1*H*-1,2,3-triazole (3y): 19
1-(2,6-Difluorobenzyl)-1*H*-1,2,3-triazolyl-4-carboxylic acid ethyl ester (3z): 19
3′-Deoxy-3′-(4-phenyl-1H-1,2,3-triazol-1-yl)thymidine, 1-((2*R*,4*S*,5*S*)-5-(hydroxymethyl)-4-(4-phenyl-1*H*-1,2,3-triazol-1-yl)tetrahydrofuran-2-yl)-5-methylpyrimidine-2,4(1*H*,3*H*)-dione (3aa): 20
1,3-Bis(1-phenyl-1*H*-1,2,3-triazol-4-yl)propane (3ab): 20
1-Phenyl-1*H*-1,2,3-triazolyl-4-carboxylic acid ethyl ester (3ac): 21
1-(3β)-Cholest-5-en-3-yl-4-phenyl-1*H*-1,2,3-triazole, 1-((3*S*,8*S*,9*S*,10*R*,13*R*,14*S*,17*R*)-10,13-dimethyl-17-((*R*)-6-methylheptan-2-yl)-2,3,4,7,8,9,10,11,12,13,14,15,16,17-tetradecahydro-1*H*-cyclopenta[*a*]phenanthren-3-yl)-4-phenyl-1*H*-1,2,3-triazole (3ad): 21
1-Phenyl-1*H*-1,2,3-triazole (3ae): 22
1-(2,6-Difluorobenzyl)-1*H*-1,2,3-triazole (3af): 22
4-Methyl-1-phenyl-1*H*-1,2,3-triazole (3ag): 22
1-Benzyl-4-methyl-1*H*-1,2,3-triazole (3ah): 22
1-Phenyl-4-propyl-1*H*-1,2,3-triazole (3ai): 23
1-Phenyl-4-(trimethylsilyl)-1*H*-1,2,3-triazole (3aj): 23
Rufinamide, 1-(2,6-difluorobenzyl)-1*H*-1,2,3-triazole-4-carboxamide (4): 23
1-(2,6,-Difluorobenzyl)-1H-1,2,3-triazole-4-carboxylic acid: 23
5. Copies of NMR, UV-Vis, FT-ATR-IR and MS spectra of 1,2,3-triazoles: 25
6. References 87

1. General methods:

All solvents and commercially available chemicals were used as received.

NMR spectra were obtained on a Bruker DPX400 MHz spectrometer. ^{1}H chemical shifts are reported as values in ppm referenced to the deuterated solvent main peak. The following abbreviations are used to assign multiplicity: s = singlet, d = doublet, t=triplet, q = quartet, qu = quintet, sx = sextet, hpt = heptet, oct = octet, br = broad. Coupling constants, J, are measured in Hertz (Hz) and if indicated are reported as $^{X}J_{Y\text{-}Z}$, where X indicates number of bonds between coupled nuclei and Y-Z indicates the nuclei. ^{13}C chemical shifts are reported as values in ppm referenced to the main peak of deuterated solvent and are proton decoupled and fluorine coupled. If indicated that neutral $CDCl_3$ was used as a solvent, chloroform-d_1 was filtrated through K_2CO_3 directly prior to use. If indicated, in the ^{13}C NMR spectra the nature of carbons (CH, CH_2 or CH_3) was determined by DEPT-135 experiment.

Mass spectrometry samples were analysed using a MaXis (Bruker Daltonics, Bremen, Germany) time of flight (TOF) mass spectrometer. Samples were introduced to the mass spectrometer via a Dionex Ultimate 3000 autosampler and UHPLC pump. Ultrahigh performance liquid chromatography was performed using a Waters, Acquity UPLC BEH C18 (50 mm x 2.1 mm 1.7um) column. Gradient elution from 5% acetonitrile (0.2% formic acid) to 100% acetonitrile (0.2% formic acid) was performed in five minutes at 0.6 mL/min. High resolution positive/negative ion electrospray ionisation mass spectra were recorded.

All *in situ* IR measurements were recorded on Bruker ALPHA FT-IR at room temperature using OPUS™ Software in transmission mode. Data was measured from 4000 to 375 cm^{-1} with 2 cm^{-1} resolution. Harrick's DLC 2™ Demountable Liquid Flow Cell with ZnSe windows and 500 µm spacers were connected to the flow system at the indicated positions. Absorptions are given in atomic unit [a. u.] and wavelength are given in wavenumbers [cm^{-1}]. Note that recorded IR spectra are saturated at regions of used solvents absorption. Fourier transform attenuated total reflectance infrared spectroscopy (FT-ATR-IR) spectra were recorded by using Thermo Scientific Nicolet IR 200 FT-IR spectrometer with a single-reflection ATR head and OMNIC™ software.

UV-Vis spectroscopy measurements were recorded with the Ocean Optics DH-2000-BAL UV spectrometer at room temperature.

Molar extinction coefficients were determined by plotting absorption vs concentration graph and using Beer-Lambert law, accordingly to the equation below, by fitting a linear function to the obtained data.

$$A = \varepsilon \cdot c \cdot l$$

Where: A – absorption [a.u.]
ε – molar extinction coefficient [M^{-1} cm^{-1}]
c – sample concentration [M]
l – path length [cm]

Thin-layer chromatography was carried out on Merck silica gel plates, which were visualised under UV irradiation of 254 nm and/or by staining with aqueous $KMnO_4$, methanolic H_2SO_4, PMA or iodine. Column chromatography was performed with Merck silica gel 60 using solvent ratios as volumes before mixing described in the method.

The Vapourtec® R series Integrated Flow Chemistry System (R2+) was the platform used for the flow experiments. Grant Optima™ TXF200 heated oil bath with high-temperature silicone oil was used for precise reaction temperature control.

2. Experimental Procedures:

Azides of low molecular weight (more than 25% nitrogen content) should not be isolated from solution since the **concentrated material is likely to be dangerously explosive**. Disposal procedure for organic azides can be found in *Hazardous Laboratory Chemicals Disposal Guide*.[1]

The following compounds were prepared by the literature methods and had characterisation data consistent with literature: phenyl azide[2], 1-azido-2-methoxybenzene[3], 1-azido-3-methoxybenzene[3], 1-azido-4-methoxybenzene[3], 1-azido-4-nitrobenzene[4], benzyl azide[5], cyclohexyl azide[6], *n*-butyl azide[7], 2,6-difluorobenzyl azide[8], 1-naphthalene azide[9], propargyl benzoate[10], 1-ethynyl-4-methoxybenzene[11], 1-ethynyl-4-nitrobenzene[12], 2-butynoic acid[13], 2-hexynoic acid[13,14], propiolamide[15], 4-azido-*N*,*N*-dimethylaniline[16], 2-(azidomethyl)benzonitrile[17], 4-chlorobenzyl azide[18], 4-(trifluoromethyl)benzyl azide[19], and 3β-azido-5-cholestene[20].

Procedure A: Synthesis of Copper-on-Charcoal[21]:

Activated carbon (50.0 g, Fisher Chemical C/4040/60) was added to a solution of $Cu(NO_3)_2{\cdot}2.5H_2O$ (10.7 g, 46.0 mmol) in deionized water (100 mL), and further deionized water (100 mL) was added to wash down the sides of the flask. The flask was loosely capped and stirred under air for 30 min and then submerged in an ultrasonic bath for 7 h. Subsequent washing with toluene and air drying (3 h) by vacuum filtration yielded "wet" Cu/C. The catalyst was further dried in vacuo at 90 °C for 4 h, then overnight at rt to obtain dry Cu/C.

Procedure B: General procedure for Copper-on-Charcoal catalysed CuAAC reaction performed in flow system:

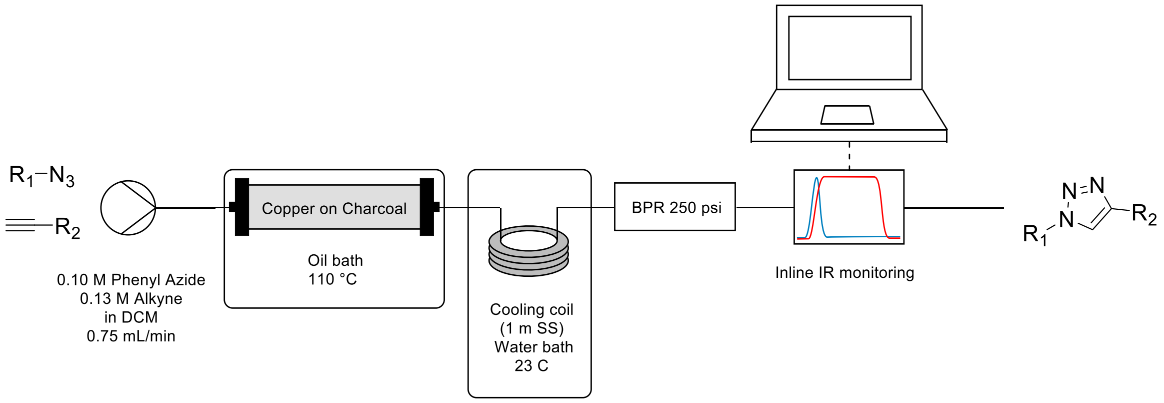

Flow stream containing 0.10 M solution of azide (1.0 eq.) and 0.13 M solution of alkyne (1.3 eq.) in DCM was directed with 0.75 mL·min^{-1} to the catalytic column (stainless steel Restek 4.6 mm ID x 150 mm column filled with 860±7 mg Cu/C, total volume of 2.49 mL, effective volume of 1.61 mL, 1.01 mmol Cu per 1.0 g of Cu/C, 0.869±0.007 mmol Cu) submerged in an oil bath at 110 °C. Additional cooling coil (1 m, 1.00 mm ID, stainless steel) submersed in water (at approx. 23 °C) was placed after the reactor to cool down reaction mixture. IR measurement cell was placed after the BPR (250 psi). Flow equipment was controlled with python script. Reaction mixture was passed through Cu/C catalytic columns for 800 s resulting in elution of 10.0 mL of reaction mixture, which was collected, solvent was then removed and obtained solid residue was preadsorbed onto silica and purified by column chromatography.

Procedure C: Calibration of IR spectrometer:

Samples of 1,4-diphenyl-1*H*-1,2,3-triazole and phenyl azide with various concentrations were prepared and measured using IR spectrometer. The samples concentrations and maxima of absorptions at characteristic peaks are reported in Table S 1. and Table S 2.

Table S 1. 1,4-Diphenyl-1H-1,2,3-triazole samples concentrations and absorbances of characteristic peaks.

1,4-Diphenyl-1*H*-1,2,3-triazole			
Sample	Concentration [M]	Absorbance at 1032 cm^{-1}	Absorbance at 1494 cm^{-1}
1	$3.16\cdot10^{-2}$	0.459	0.549
2	$1.58\cdot10^{-2}$	0.243	0.289
3	$1.05\cdot10^{-2}$	0.174	0.205
4	$7.89\cdot10^{-3}$	0.135	0.159
5	$6.31\cdot10^{-3}$	0.086	0.103
6	$1.26\cdot10^{-3}$	0.018	0.022

Table S 2. Phenyl azide samples concentrations and measured absorbances of characteristic peak.

Phenyl Azide		
Sample	Concentration [M]	Absorbance at 2081 cm^{-1}
1	$1.57\cdot10^{-2}$	0.340
2	$7.86\cdot10^{-3}$	0.167
3	$3.93\cdot10^{-3}$	0.096
4	$1.97\cdot10^{-3}$	0.062

Molar extinction coefficients were then calculated using Beer-Lambert law by plotting maxima of absorptions vs samples concentration multiplied by IR cell path length and were determined as slope of fitted linear functions.

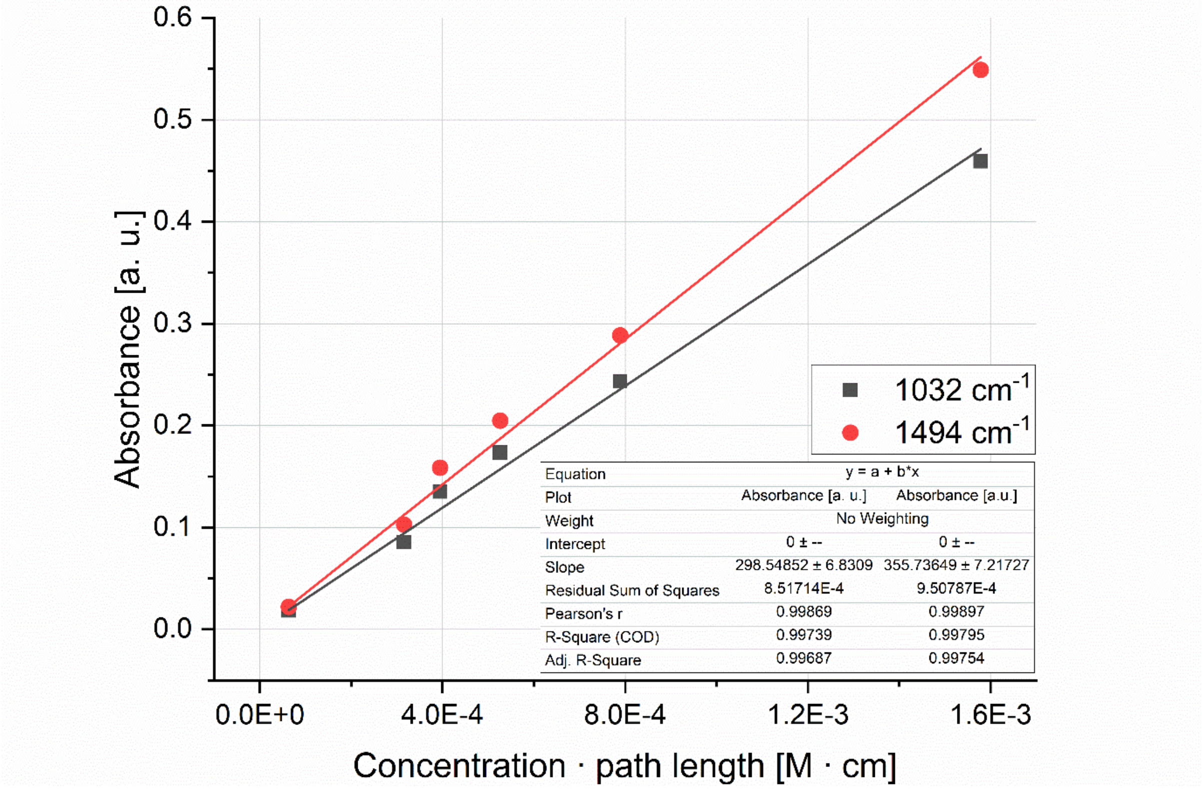

Figure S 1. IR absorption calibration curve for 1,4-diphenyl-1H-1,2,3-triazole at 1032 and 1494 cm^{-1}.

1,4-Diphenyl-1*H*-1,2,3-triazole: $\varepsilon^{DCM}_{1032\,cm^{-1}} = 298.5 \pm 6.8 \left[\frac{1}{M\cdot cm}\right]$

$$\text{1,4-Diphenyl-1}H\text{-1,2,3-triazole: } \varepsilon_{1494\,cm^{-1}}^{DCM} = 355.7 \pm 7.2 \left[\frac{1}{M \cdot cm}\right]$$

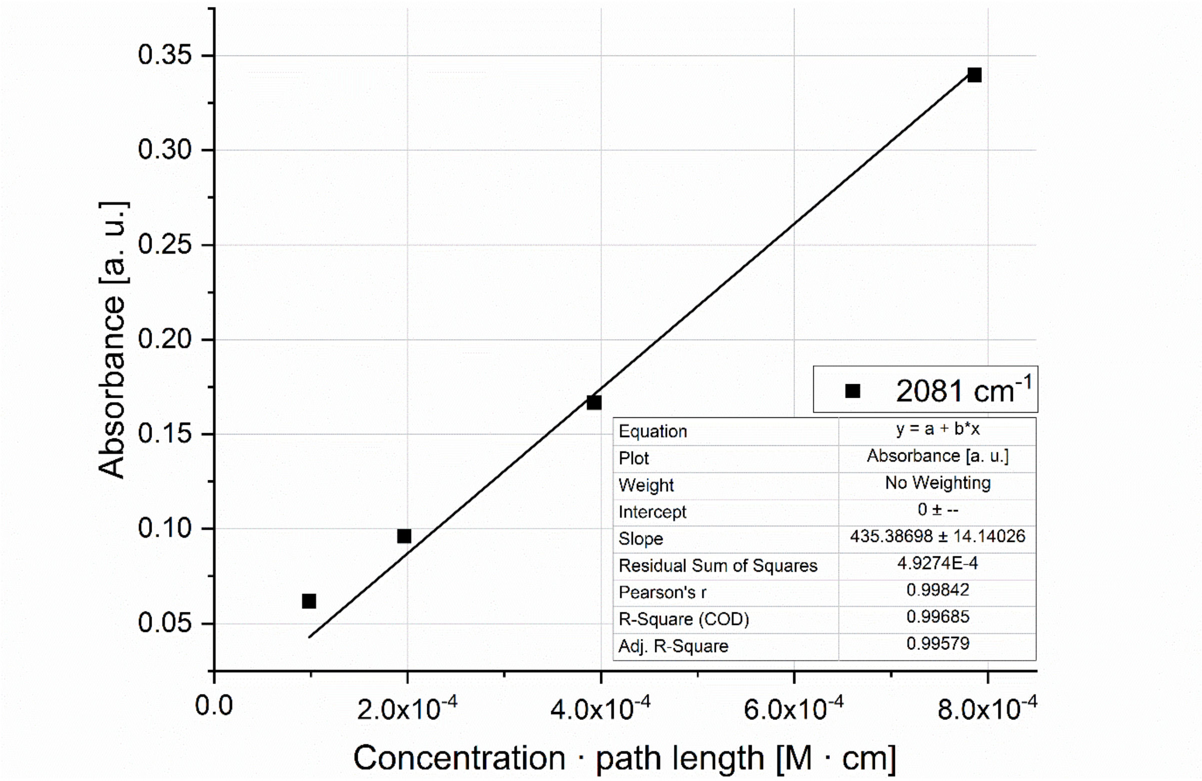

Figure S 2.IR absorption calibration curve for phenyl azide at 2081 cm-1.

$$\text{Phenyl Azide: } \varepsilon_{2081\,cm^{-1}}^{DCM} = 435.4 \pm 14.1 \left[\frac{1}{M \cdot cm}\right]$$

3. Trials employing metallic copper powder as CuAAC reaction catalyst:

Prior to CuAAC reaction catalysed by copper-on-charcoal, metallic copper powders were tested for their possible application as heterogeneous catalysts under continuous flow conditions. Two commercial copper powders were used:

- Cu powder, semispherical, -50+70 mesh (210-297 µm particles size), 99% purity, from AlfaAesar, catalogue number 45030.22, lot number M27D044
- Cu powder, spheroidal, 14-25 µm particles size, 98% purity, from MilliporeSigma, catalogue number 326453, lot number MKCG7684

Test reactions were performed in the following flow system:

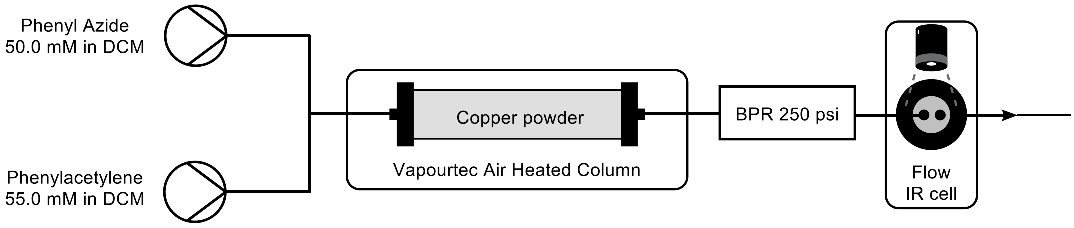

Figure S 3. Flow system used for screening of metallic copper powders in CuAAC reaction.

Procedure D: Tests of metallic copper as CuAAC reaction catalysts:

First flow stream containing 0.050 M solution of phenyl azide (1.0 eq.) in DCM and second flow stream containing a 0.055 M solution of phenylacetylene (1.1 eq.) in DCM were directed via the 3-way connector to the catalytic column (OmniFit 500 x 5mm or 1000 x 5mm) heated with hot air. IR

measurement flow cell was placed after the BPR (250 psi). Various flow rates and reactor temperatures were used.

Calibrated IR monitoring was used for *in situ* reaction monitoring and absorbances of reaction components at their specific absorption bands were plotted over time. Initial experiments were performed with 0.50 mL·min^{-1} flow rate for each pump, resulting in 30 seconds residence time, and reactor temperature of 50 °C. First experiment in which catalytic column was left empty was performed to determine if thermal alkyne-azide cycloaddition was taking place was performed, and as judged form IR monitoring (Figure S 4. Reaction components specific bands absorptions in the experiment with empty catalytic column.) , no product was formed. Note that slight increase of absorbance at 1494 cm^{-1} band is caused by close proximity of other absorbing bands.

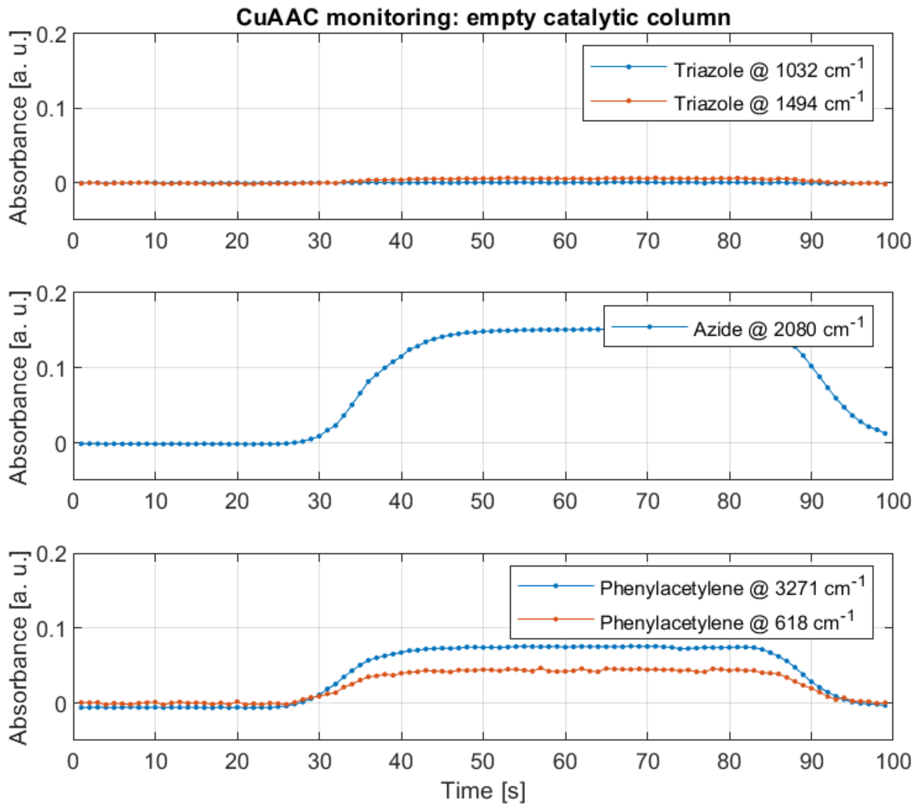

Figure S 4. Reaction components specific bands absorptions in the experiment with empty catalytic column.

In next experiments, catalytic column was filled with the copper powders and the same reaction conditions were applied as before. Unfortunately, neither experiment delivered desired 1,2,3-triazole, as judged from IR monitoring (Figure S 5., Figure S 6.)

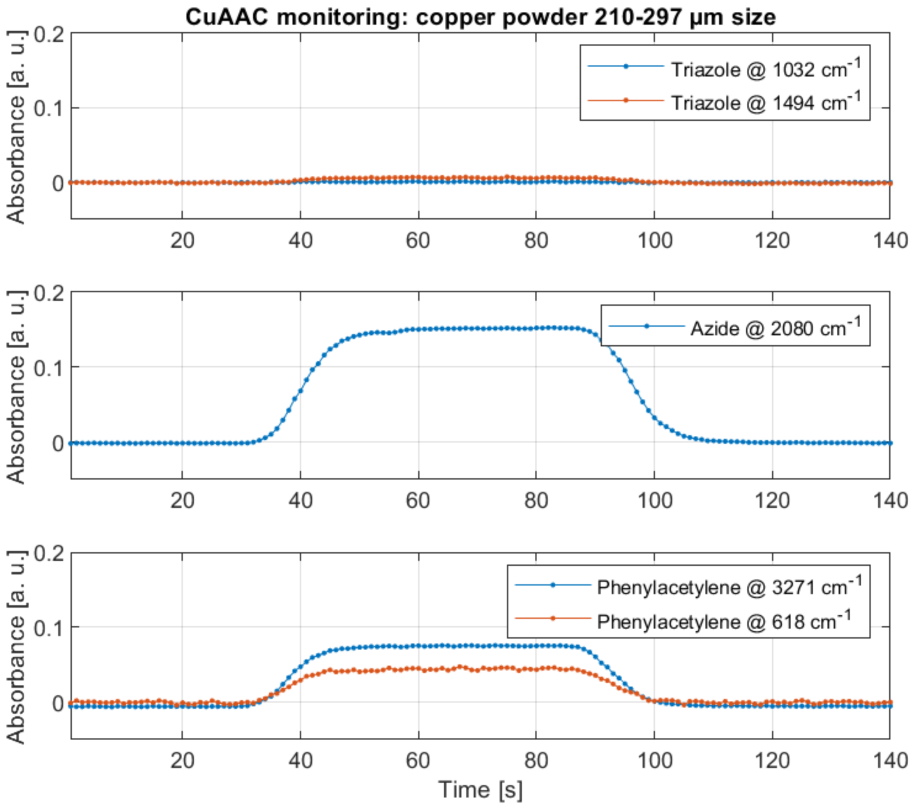

Figure S 5. Reaction components specific bands absorptions in the experiment with 210-297 μm copper powder.

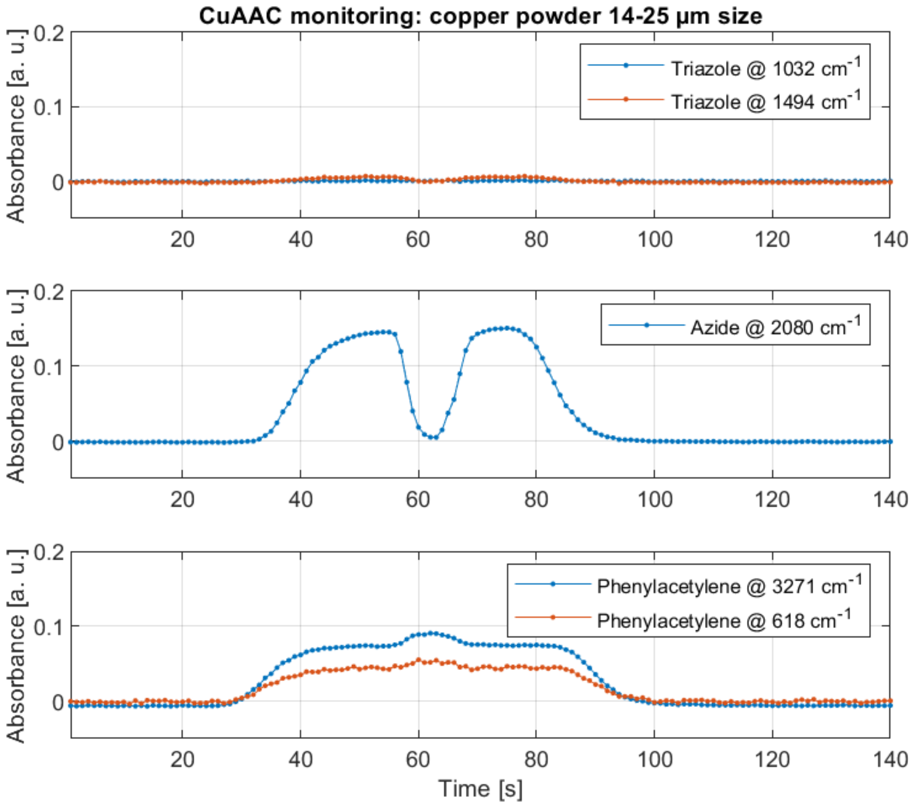

Figure S 6. Reaction components specific bands absorptions in the experiment with 14-25 μm copper powder.

Experiments in which temperature (up to 150 °C) and reaction residence time (up to 5 minutes) were increased were performed using the 210-297 µm size copper powder. Additionally, reagents concentrations were increased to 0.10 M and 0.11 M of phenyl azide and phenylacetylene, respectively. No significant amount product was observed in any of these experiments, as judged by *in situ* IR spectroscopy (Figure S 7.).

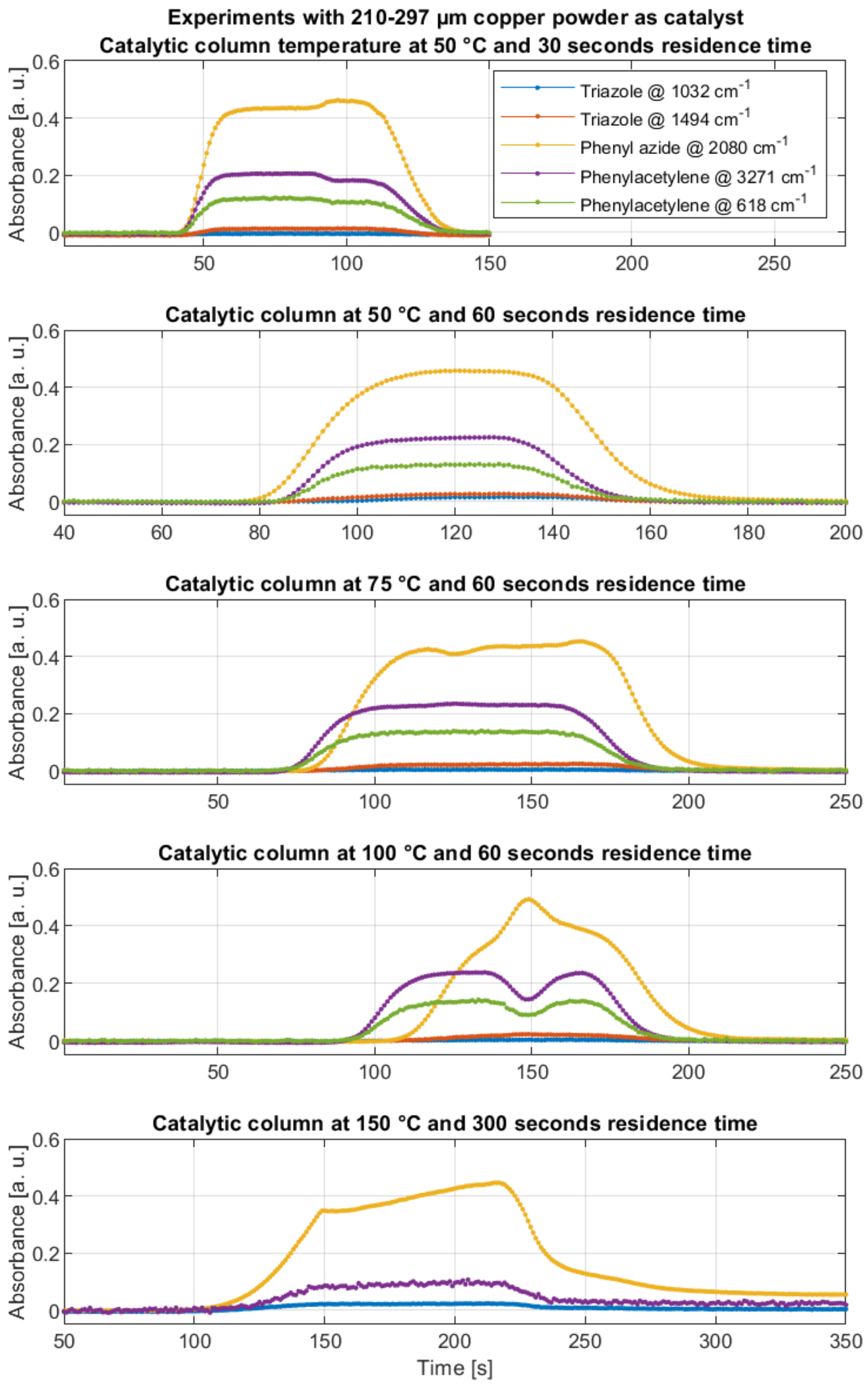

Figure S 7. Reaction components specific bands absorptions in the experiments with 210-297 µm copper powder using various catalytic column temperatures and residence times.

Moreover, experiments with addition of 1.0 equivalent of amine (such as triethylamine, diethylamine and DABCO) were performed at 75 °C and 60 seconds residence time, but no product was formed under these conditions.

It is well known, that CuAAC reaction is catalysed by Cu(I) species, it was then envisioned that metallic copper may need to be activated prior to the cycloaddition reaction, thus two different activation techniques were used, namely activation with elemental iodine to form copper(I) iodide and activation with hydrogen peroxide to form copper oxides species on the surface of the metallic copper.

Procedure E: Activation of metallic copper surface with iodine:
Iodine (0.7 g) was added to a stirring mixture of metallic copper (210-297 µm particles size, 0.4 g) in DCM (25.0 mL) and the mixture was loosely capped and stirred at rt for one hour. Then the mixture was filtrated, washed thoroughly with acetone, dried on air and used without further purification.

Procedure F: Activation of metallic copper surface with hydrogen peroxide:
The round-bottom flask containing metallic copper (210-297 µm particles size, 0.4 g) was evacuated and filled with nitrogen three times, then 30% hydrogen peroxide (50.0 mL) was transferred via cannula to the flask with copper powder. The resulting mixture was stirred at rt for one hour, then it was filtrated, washed thoroughly with water and acetone, dried on air, and used without further purification.

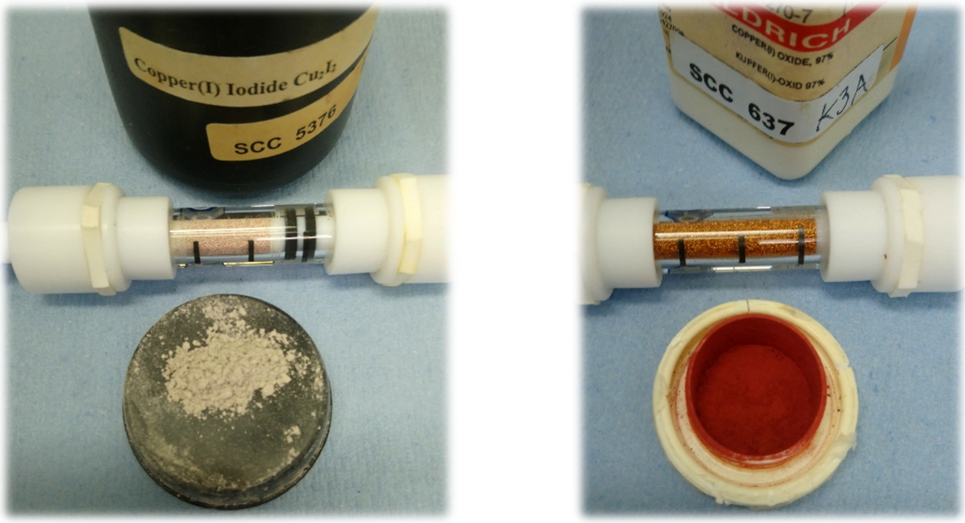

Figure S 8. Visual comparison of obtained activated copper powders with commercial samples of CuI and Cu_2O.
Left: Comparison of iodine activated copper powder with commercially available copper iodide.
Right: Comparison of hydrogen peroxide activated copper powder with commercially available copper(I) oxide.

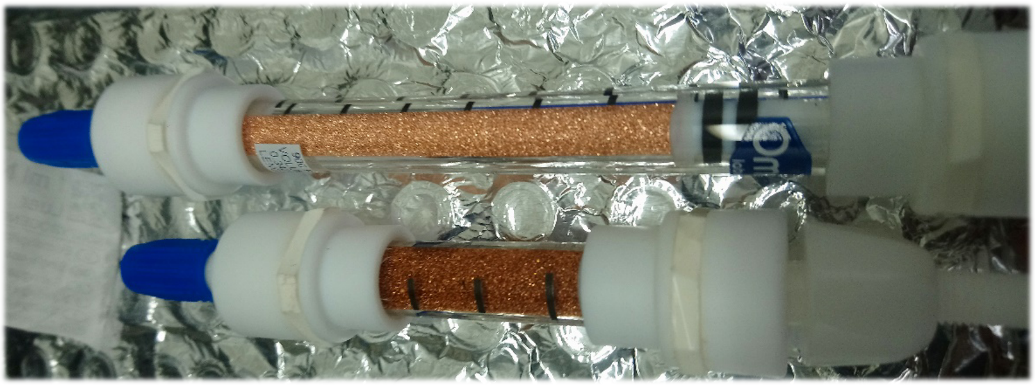

Figure S 9. Comparison of hydrogen peroxide activated copper (bottom) with untreated metallic copper (top).

Obtained treated copper powders were then placed inside the catalytic column and used in a click reaction using previous flow system. As judged from *in situ* IR monitoring (Figure S 10.), only negligible amounts of 1,4-diphenyl-1*H*-1,2,3-triazole were obtained, as the absorbance of both characteristic triazole bands at 1032 and 1494 cm^{-1} increased slightly during both experiments and were just distinguishable from the baseline.

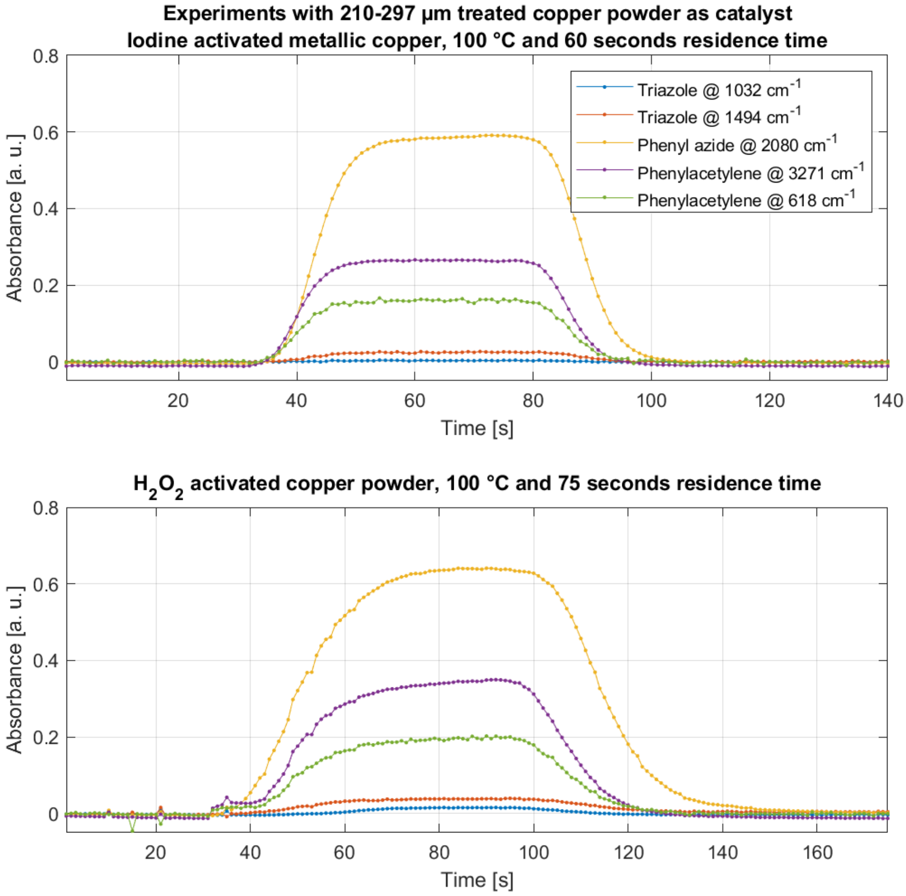

Figure S 10. Reaction components specific bands absorptions in the experiments with 210-297 µm treated copper powder.
Top: Reaction components IR absorbances in experiment using iodine treated copper powder.
Bottom: Reaction components IR absorbances in experiment using H_2O_2 treated copper powder.

Apart from chemical activation with iodine and hydrogen peroxide, thermal activation of copper powders under air atmosphere was performed in order to obtain mixed copper oxides or copper nanoparticles on surface of used metallic copper, and are described below:

- Copper powder (210-297 µm) was gently heated with a heat gun (approx. 165 °C) until the it changed colour to burgundy, suggesting formation of copper(I) oxides on the surface of the metallic copper.
- Copper powder (210-297 µm) was heated to 250 °C overnight to allow for high oxidation levels of copper.
- Copper powder (14-25 µm) was gently heated with a heat gun (approx. 165 °C) until it changed colour to burgundy/dark red, suggesting formation of copper(I) oxides on the surface of the metallic copper.
- Copper powder (14-25 µm) was heated to 250 °C overnight to allow for high oxidation levels of copper.

All described thermal activations resulted in change of colours of the metallic copper, but when obtained materials were applied as catalysts in the previously used flow system, none delivered desired 1,2,3-triazole, as judged form the IR monitoring. Additional reaction with 10-fold increased concentration, i.e. 1.0 M phenyl azide and 1.1 M phenylacetylene, using copper powder (14-25 µm) heated to 250 °C overnight as catalyst, catalytic column temperature of 100 °C and 60 seconds residence time delivered only traces of product (corresponding to 2.6% yield, Figure S 11.), as judged from the IR absorbance.

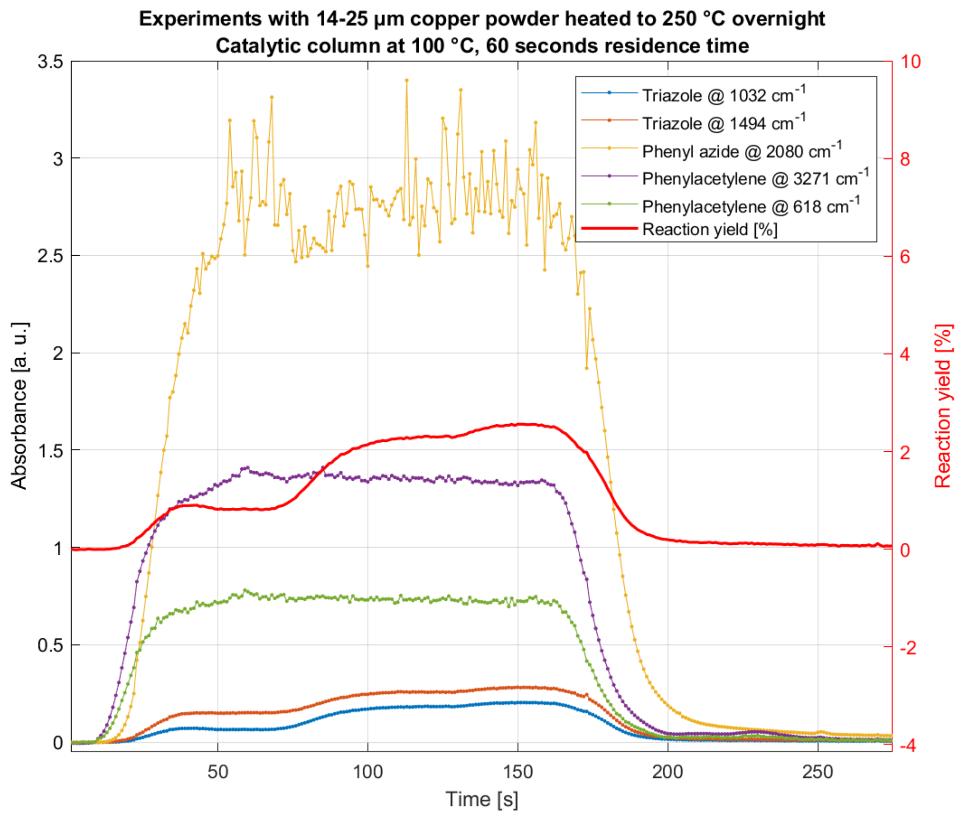

Figure S 11. IR monitoring of reaction with copper powder (14-25 µm) heated to 250 °C overnight, 10-fold increase in reagents concentration, 100 °C catalytic column temperature, and 60 seconds residence time. Note that absorbance fluctuations of phenyl azide band at 2080 cm^{-1} are caused by the saturation of the spectrometer due to high azide concentration.

4. Procedures, Purification and Characterization Data of 1*H*-1,2,3-Triazoles:

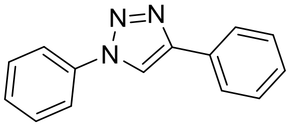

$C_{14}H_{11}N_3$
MW: 221.26
CAS 13148-78-2

1,4-Diphenyl-1*H*-1,2,3-triazole (3a): Prepared following the procedure B and purified by column chromatography (SiO_2; *n*-hexane:AcOEt 9:1 to 5:5, R_f = 0.15 in 10% AcOEt in *n*-hexane) and was isolated as a white solid, 220.8 mg (99.8% yield). **Mp:** 184.0 - 185.0 °C, lit. 183 – 184 °C [22]; **^{1}H NMR (400 MHz, $CDCl_3$) δ (ppm) =** 8.20 (s, 1H), 7.92 (m, 2H), 7.80 (m, 2H), 7.56 (m, 2H), 7.50 – 7.44 (m, 3H), 7.38 (m, 1H). **^{13}C NMR (101 MHz, $CDCl_3$) δ (ppm) =** 148.6, 137.3, 130.4, 130.0, 129.1, 128.9, 128.6, 126.0, 120.7, 117.7. [22]

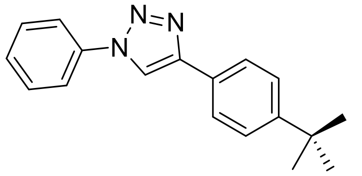

$C_{18}H_{19}N_3$
MW: 277.37
CAS 1683619-47-7

1-Phenyl-4-(4-*tert*-butylphenyl)-1*H*-1,2,3-triazole (3b): Prepared following the procedure B and purified by column chromatography (SiO_2; *n*-hexane:AcOEt 9:1 to 5:5, R_f = 0.20 in 10% AcOEt in *n*-hexane) and was isolated as a white solid, 257.4 mg (92.8% yield). **Mp:** 143.0 - 143.6 °C; **^{1}H NMR (400 MHz, $CDCl_3$) δ (ppm) =** 8.17 (s, 1H), 7.85 (m, 2H), 7.80 (m, 2H), 7.55 (m, 2H), 7.49 (m, 2H), 7.45 (m, 1H), 1.37 (s, 9H). **^{13}C NMR (101 MHz, $CDCl_3$) δ (ppm) =** 151.7, 145.6, 137.3, 129.9, 128.8, 127.6, 126.0, 125.8, 120.7, 117.4, 34.9, 31.4.[23]

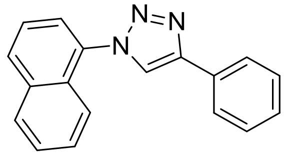

$C_{18}H_{13}N_3$
MW: 271.32
CAS 940891-83-8

1-(Naphthalen-1-yl)-4-phenyl-1*H*-1,2,3-triazole (3c): Prepared following the procedure B and purified by column chromatography (SiO_2; *n*-hexane:AcOEt 9:1 to 5:5, R_f = 0.18 in 10% AcOEt in *n*-hexane) and was initially isolated as a colourless oil, which after 96 hours at -20 °C changed to white solid, 266.2 mg (98.1% yield). **Mp:** 91.1 - 92.0 °C, lit. 89 - 91 °C [24]; **^{1}H NMR (400 MHz, $CDCl_3$) δ (ppm) =** 8.16 (s, 1H), 8.05 (dt, J=8.0, 1.3 Hz, 1H), 8.00 – 7.95 (m, 3H), 7.71 (m, 1H), 7.68 – 7.55 (m, 4H), 7.49 (m, 2H), 7.39 (m, 1H). **^{13}C NMR (101 MHz, $CDCl_3$) δ (ppm) =** 147.9, 134.4, 133.9, 130.6, 130.4, 129.1, 128.8, 128.6, 128.5, 128.1, 127.3, 126.1, 125.2, 123.7, 122.6, 122.4.[24]

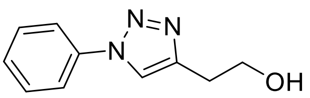

$C_{10}H_{11}N_3O$
MW: 189.22
CAS 1248403-05-5

2-(1-Phenyl-1*H*-1,2,3-triazol-4-yl)ethan-1-ol (3d): Prepared following the procedure B and purified by column chromatography (SiO_2; *n*-hexane:AcOEt 8:2 to 2:8, R_f = 0.00 in 10% AcOEt in *n*-hexane) and was isolated as a white solid, 167.1 mg (88.3% yield). **Mp:** 41.0 - 41.5 °C; **^{1}H NMR (400 MHz, $CDCl_3$) δ (ppm) =** 7.85 (t, J= 0.8 Hz, 1H), 7.73 (m, 2H), 7.52 (m, 2H), 7.43 (m, 1H), 4.03 (q, J=5.8 Hz, 2H), 3.05 (td, J=5.7 Hz, J=0.7 Hz, 2H), 2.48 (t, J=6.0 Hz, 1H). **^{13}C NMR (101 MHz, $CDCl_3$, neutral pH) δ (ppm) =** 146.4, 137.3, 129.9, 128.8, 120.7, 120.0, 61.8, 31.1, 28.9.[25]

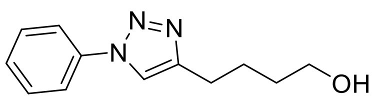

$C_{12}H_{15}N_3O$
MW: 217.27
CAS 875312-68-8

4-(1-Phenyl-1*H*-1,2,3-triazol-4-yl)butan-1-ol (3e): Prepared following the procedure B and purified by column chromatography (SiO_2; *n*-hexane:AcOEt 9:1 to 5:5, R_f = 0.15 in 10% AcOEt in *n*-hexane) and was isolated as a white solid, 188.1 mg (86.6% yield). **Mp:** 36.0 - 36.5 °C; **^{1}H NMR (400 MHz, $CDCl_3$) δ (ppm) =** 7.74 (br s, 1H), 7.71 (m, 2H), 7.51 (m, 2H), 7.42 (m, 1H), 3.71 (t, J=6.4 Hz, 2H), 2.85 (td, J=7.6, 0.7 Hz, 2H), 1.95 (m, 2H), 1.68 (m, 2H), 1.54 (br s, 1H). **^{13}C NMR (101 MHz, $CDCl_3$, neutral pH) δ (ppm) =** 148.9, 137.4, 129.8, 128.6, 120.6, 119.1, 62.7, 32.3, 25.7, 25.5.[12]

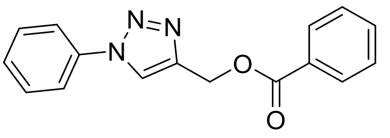

$C_{16}H_{13}N_3O_2$
MW: 279.30
CAS 141581-63-7

(1-Phenyl-1*H*-1,2,3-triazol-4-yl)methyl benzoate (3f): Prepared following the procedure B and purified by column chromatography (SiO_2; *n*-hexane:AcOEt 9:1 to 5:5, R_f = 0.11 in 10% AcOEt in *n*-hexane) and was isolated as a white solid, 276.4 mg (99.0% yield). **Mp:** 116.5 - 118.0 °C, lit. 110 – 111 °C [26]; **^{1}H NMR (400 MHz, $CDCl_3$) δ (ppm) =** 8.14 (s, 1H), 8.07 (m, 2H), 7.74 (m, 2H), 7.59 – 7.49 (m, 3H), 5.56 (s, 2H). **^{13}C NMR (101 MHz, $CDCl_3$) δ (ppm) =** 166.7, 143.8, 137.1, 133.4, 129.9, 129.9, 129.8, 129.1, 128.6, 122.4, 120.8, 58.2.[26]

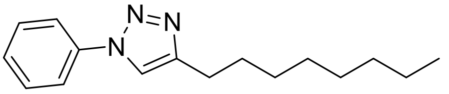

$C_{16}H_{23}N_3$
MW: 257.38
CAS 1222130-89-3

4-Octyl-1-phenyl-1*H*-1,2,3-triazole (3g): Prepared following the procedure B and purified by column chromatography (SiO_2; *n*-hexane:AcOEt 9:1 to 5:5, R_f = 0.23 in 10% AcOEt in *n*-hexane) and was isolated as a white solid, 257.0 mg (99.9% yield). **Mp:** 49.0 - 49.7 °C; **^{1}H NMR (400 MHz, $CDCl_3$) δ (ppm) =** 7.73 (m, 1H), 7.71 (m, 2H), 7.51 (m, 2H), 7.41 (m, 1H), 2.80 (td, J=7.8, 0.4 Hz, 2H), 1.74 (m, 2H), 1.46 – 1.24 (m, 10H), 0.88 (t, J=7.0 Hz, 3H). **^{13}C NMR (101 MHz, $CDCl_3$) δ (ppm) =** 149.3, 137.4, 129.8, 128.6, 120.6, 119.0, 32.0, 29.6, 29.5, 29.4, 29.4, 25.8, 22.8, 14.2.[27]

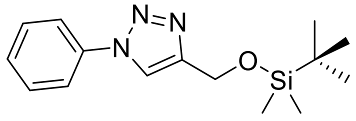

$C_{15}H_{23}N_3OSi$
MW: 289.45
CAS 2537681-22-2

4-(((*tert*-Butyldimethylsilyl)oxy)methyl)-1-phenyl-1*H*-1,2,3-triazole (3h): Prepared following the procedure B and purified by column chromatography (SiO_2; *n*-hexane:AcOEt 1:0 to 9:1, R_f = 0.29 in 10% AcOEt in *n*-hexane) and was isolated as a white solid, 257.8 mg (89.1% yield). **Mp:** 56.0 - 56.5 °C; **^{1}H NMR (400 MHz, $CDCl_3$) δ (ppm) =** 7.90 (t, J=0.8 Hz, 1H), 7.74 (m, 2H), 7.52 (m, 2H), 7.43 (m, 1H), 4.94 (d, J=0.8 Hz, 2H), 0.94 (s, 9H), 0.14 (s, 6H). **^{13}C NMR (101 MHz, $CDCl_3$) δ (ppm) =** 148.6, 137.3, 130.4, 130.0, 129.1, 128.9, 128.6, 126.0, 120.7, 117.7. **FT-ATR-IR IR** ν (cm^{-1}) = 3135, 2954, 2930, 2896, 2884, 2857, 1605, 1553, 1506, 1467, 1387, 1347, 1251, 1231, 1210, 1178, 1062, 1038, 1015, 1004, 973, 939, 908, 841, 773, 755, 708, 686, 676, 666, 644, 574. **UV-Vis (DCM) λ_{max} (ε):** 249 nm (11813 M^{-1} cm^{-1}). **LR-ESI-MS m/z:** 290.3098 (M + H^+); **HR-ESI-MS m/z:** 290.1689 (M + H^+), 312.1508 (M + Na^+); calcd for $C_{15}H_{24}N_3OSi$: 290.1683, for $C_{15}H_{23}N_3OSiNa$: 312.1503.

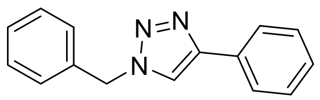

$C_{15}H_{13}N_3$
MW: 235.29
CAS 108717-96-0

1-Benzyl-4-phenyl-1*H*-1,2,3-triazole (3i): Prepared following the procedure B and purified by column chromatography (SiO_2; *n*-hexane:AcOEt 9:1 to 5:5, R_f = 0.23 in 20% AcOEt in *n*-hexane, R_f = 0.57 in 40% AcOEt in *n*-hexane, R_f = 0.79 in 60% AcOEt in *n*-hexane) and was isolated as a white solid, 221.0 mg (93.9% yield). **Mp:** 126.4-127.3 °C, lit. 128 - 130 °C [28]; **^{1}H NMR (400 MHz, $CDCl_3$) δ (ppm) =** 8.80 (m, 2H), 7.66 (s, 1H), 7.42 – 7.34 (m, 5H), 7.34 – 7.29 (m, 2H). **^{13}C NMR (101 MHz, $CDCl_3$) δ (ppm) =** 148.4, 134.9, 130.7, 129.3, 129.0, 128.3, 128.2, 125.9, 119.6, 54.4.[28]

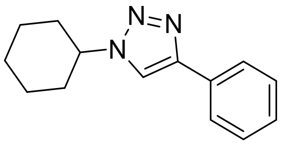

$C_{14}H_{17}N_3$
MW: 227.31
CAS 116436-13-6

1-Cyclohexyl-4-phenyl-1*H*-1,2,3-triazole (3j): Prepared following the procedure B and purified by column chromatography (SiO_2; *n*-hexane:AcOEt 9:1 to 5:5, R_f = 0.23 in 10% AcOEt in *n*-hexane) and was isolated as a white solid, 181.7 mg (79.9% yield). **Mp:** 112.7 – 113.3 °C, lit. 110 - 111 °C [29]; **^{1}H NMR (400 MHz, $CDCl_3$) δ (ppm) =** 7.83 (m, 2H), 7.76 (s, 1H), 7.41 (m, 2H), 7.32 (m, 1H), 4.49 (tt, J=11.8, 3.9 Hz, 1H), 2.26 (ddtd, J=12.8, 4.0, 2.6, 1.6 Hz, 2H), 1.94 (dt, J=13.8, 3.5 Hz, 2H), 1.85 – 1.74 (m, 3H), 1.48 (qt, J=13.3, 3.4 Hz, 2H), 1.30 (qt, J=12.9, 3.6 Hz, 1H). **^{13}C NMR (101 MHz, $CDCl_3$) δ (ppm) =** 147.4, 131.0, 128.9, 128.1, 125.8, 117.4, 60.3, 33.8, 25.3, 25.3.[29]

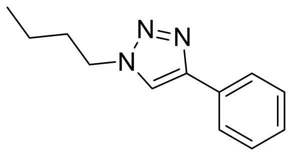

$C_{12}H_{15}N_3$
MW: 201.27
CAS 754982-92-8

1-Butyl-4-phenyl-1*H*-1,2,3-triazole (3k): Prepared following the procedure B and purified by column chromatography (SiO_2; *n*-hexane:AcOEt 8:2 to 4:6, R_f = 0.04 in 10% AcOEt in *n*-hexane) and was isolated as a white solid, 197.1 mg (97.9% yield). **Mp:** 50.2 – 50.9 °C, lit. 47 - 48 °C [30]; **^{1}H NMR (400 MHz, $CDCl_3$) δ (ppm) =** 7.83 (m, 2H), 7.74 (s, 1H), 7.42 (m, 2H), 7.33 (m, 1H), 4.40 (t, J=7.2 Hz, 2H), 1.94 (tt, J=7.6 Hz, J=7.5 Hz, 2H), 1.40 (m, 2H), 0.98 (t, J=7.4 Hz, 3H). **^{13}C NMR (101 MHz, $CDCl_3$) δ (ppm) =** 147.9, 130.9, 129.0, 128.2, 125.8, 119.5, 50.3, 32.5, 19.9, 13.6.[30]

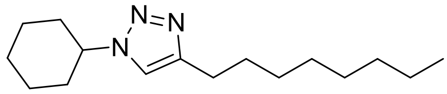

$C_{16}H_{29}N_3$
MW: 263.43
CAS 2590217-97-1

1-Cyclohexyl-4-octyl-1*H*-1,2,3-triazole (3l): Prepared following the procedure B and purified by column chromatography (SiO_2; *n*-hexane:AcOEt 9:1 to 5:5, R_f = 0.08 in 10% AcOEt in *n*-hexane, TLC stained with phosphomolybdic acid) and was isolated as a white solid, 257.2 mg (97.6% yield). **Mp:** 61.0 – 62.1 °C; **^{1}H NMR (400 MHz, $CDCl_3$) δ (ppm)** = 7.25 (t, J=0.7 Hz, 1H), 4.41 (tt, J=11.8, 3.9 Hz, 1H), 2.69 (td, J_t=7.8, 0.7 Hz, 2H), 2.19 (m, 2H), 1.91 (m, 2H), 1.80 – 1.61 (m, 5H), 1.45 (qt, J=13.3, 3.4 Hz, 2H), 1.39 – 1.20 (m, 11H), 0.88 (t, J=6.9 Hz, 3H). **^{13}C NMR (101 MHz, $CDCl_3$) δ (ppm) =** 148.2 (C), 118.2 (CH), 60.0 (CH), 33.8 (CH_2), 32.0 (CH_2), 29.7 (CH_2), 29.5 (CH_2), 29.5 (CH_2), 29.4 (CH_2), 26.0 (CH_2), 25.4 (CH_2), 25.4 (CH_2), 22.8 (CH_2), 14.3 (CH_3). **UV-Vis (DCM) λ_{max} (ε):** no absorption peaks at >250 nm. **FT-ATR-IR IR** ν (cm^{-1}) = 3117, 3063, 2922, 2852, 1557, 1467, 1446, 1375, 1336, 1281, 1214, 1201, 1156, 1055, 1030, 996, 894, 866, 848, 820, 768, 721, 661. **LR-ESI-MS m/z:** 264.3875 (M + H^+); **HR-ESI-MS m/z:** 264.2439 (M + H^+), 286.2254 (M + Na^+); calcd for $C_{16}H_{30}N_3$ 264.2434, for $C_{16}H_{29}N_3Na$ 286.2254.

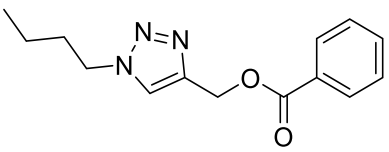

$C_{14}H_{17}N_3O_2$
MW: 259.31
CAS 1430799-22-6

(1-Butyl-1*H*-1,2,3-triazol-4-yl)methyl benzoate (3m): Prepared following the procedure B and purified by column chromatography (SiO_2; *n*-hexane:AcOEt 8:2 to 5:5, R_f = 0.54 in AcOEt:*n*-hexane 8:2) and was isolated as a white solid, 258.0 mg (99.5% yield). **Mp:** 75.0 – 75.7 °C; **^{1}H NMR (400 MHz, $CDCl_3$) δ (ppm) =** 8.05 (m, 2H), 7.67 (s, 1H), 7.55 (m, 1H), 7.43 (m, 2H), 5.48 (s, 2H), 4.35 (t, J=7.3 Hz, 2H), 1.90 (m, 2H), 1.37 (m, 2H), 0.96 (t, J=7.4 Hz, 3H).[31] **^{13}C NMR (101 MHz, $CDCl_3$) δ (ppm) =** 166.7, 143.0, 133.3, 130.0, 129.9, 128.5, 123.9, 58.3, 50.3, 32.4, 19.9, 13.6.

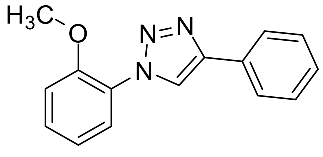

$C_{15}H_{13}N_3O$
MW: 251.29
CAS 940891-81-6

1-(2-Methoxyphenyl)-4-phenyl-1*H*-1,2,3-triazole (3n): Prepared following the procedure B and purified by column chromatography (SiO_2; *n*-hexane:AcOEt 9:1 to 7:3, R_f = 0.20 in AcOEt:*n*-hexane 1:9) and was isolated as a white solid, 230.0 mg (91.5% yield). **Mp:** 93.2 – 93.8 °C, lit. 90 – 92 °C [32]; **^{1}H NMR (400 MHz, $CDCl_3$) δ (ppm) =** 8.33 (s, 1H), 7.95 – 7.90 (m, 2H), 7.84 (dd, J=7.9 Hz, J=1.7 Hz, 1H), 7.49 – 7.41 (m, 3H), 7.13 (td, J=7.7 Hz, J=1.2 Hz, 1H), 7.11 (dd, J= 8.3 Hz, J=1.1 Hz, 1H), 3.92 (s, 3H). **^{13}C NMR (101 MHz, $CDCl_3$) δ (ppm) =** 151.3, 147.3, 130.8, 130.3, 129.0, 128.3, 126.5, 126.0, 125.6, 121.9, 121.4, 112.5, 56.2.[32]

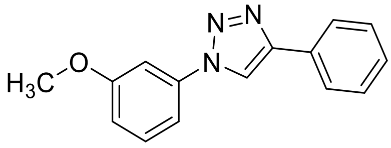

$C_{15}H_{13}N_3O$
MW: 251.29
CAS 1259900-48-5

1-(3-Methoxyphenyl)-4-phenyl-1*H*-1,2,3-triazole (3o): Prepared following the procedure B and purified by column chromatography (SiO_2; *n*-hexane:AcOEt 9:1 to 7:3, R_f = 0.17 in AcOEt:*n*-hexane 1:9) and was isolated as a white solid, 223.5 mg (88.9% yield). **Mp:** 112.7 – 113.1 °C, lit. 111 - 113 °C [33]; **^{1}H NMR (400 MHz, $CDCl_3$) δ (ppm) =** 8.20 (s, 1H), 7.93 (m, 2H), 7.50 – 7.35 (m, 5H), 7.32 (ddd, J=8.0 Hz, J=2.5 Hz, J=0.9 Hz, 1H), 7.00 (ddd, J=8.3 Hz, J=2.5 Hz, J=0.9 Hz, 1H), 3.91 (s, 3H). **^{13}C NMR (101 MHz, $CDCl_3$) δ (ppm) =** 160.7, 148.2, 138.0, 130.6, 129.9, 129.0, 128.6, 125.9, 117.8, 114.8, 112.4, 55.7.[33]

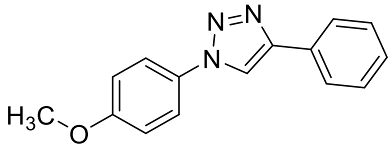

$C_{15}H_{13}N_3O$
MW: 251.29
CAS 116557-89-2

1-(4-Methoxyphenyl)-4-phenyl-1*H*-1,2,3-triazole (3p): Prepared following the procedure B and purified by column chromatography (SiO_2; *n*-hexane:AcOEt 9:1 to 7:3, R_f = 0.09 in AcOEt:*n*-hexane 1:9) and was isolated as a white solid, 219.4 mg (87.3% yield). **Mp:** 167.7 – 168.3 °C, lit. 166 - 169 °C [33]; **^{1}H NMR (400 MHz, $CDCl_3$) δ (ppm) =** 8.11 (s, 1H), 7.91 (m, 2H), 7.68 (m, 2H), 7.46 (m, 2H), 7.36 (m, 1H), 7.04 (m, 2H), 3.88 (s, 3H). **^{13}C NMR (101 MHz, $CDCl_3$) δ (ppm) =** 160.0, 148.3, 130.7, 130.4, 129.1, 128.5, 126.0, 122.4, 118.0, 115.0, 55.8.[33]

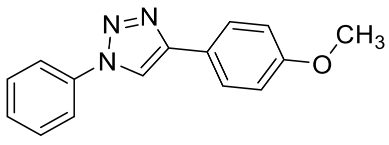

$C_{15}H_{13}N_3O$
MW: 251.29
CAS 68809-41-6

1-Phenyl-4-(4-methoxyphenyl)-1*H*-1,2,3-triazole (3q): Prepared following the procedure B and purified by column chromatography (SiO_2; *n*-hexane:AcOEt 9:1 to 6:4, R_f = 0.08 in 10% AcOEt in *n*-hexane) and was isolated as a white solid, 229.5 mg (91.3% yield). **Mp:** 152.0 – 152.7 °C, lit. 151 - 154 °C [34]; **^{1}H NMR (400 MHz, $CDCl_3$) δ (ppm) =** 8.11 (s, 1H), 7.85 (m, 2H), 7.80 (m, 2H), 7.55 (m, 2H), 7.46 (m, 1H), 7.00 (m, 2H), 3.87 (s, 3H). **^{13}C NMR (101 MHz, $CDCl_3$) δ (ppm) =** 160.3, 148.1, 130.0, 129.2, 127.6, 120.7, 117.1, 114.6, 55.5, 31.1.[34]

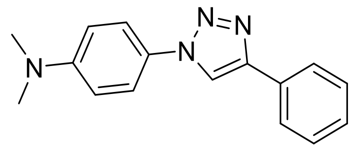

$C_{16}H_{16}N_4$
MW: 264.33200
CAS 1428739-69-8

N,N-Dimethyl-4-(4-phenyl-1H-1,2,3-triazol-1-yl)aniline (3r): Prepared following the procedure B, solvent was then removed from reaction mixture and obtained residue was dried *in vacuo*. Desired product was isolated as a brown solid (R_f= 0.15 in 20% AcOEt in *n*-hexane), 219.1 mg (82.9% yield). **Mp:** 168.8 -171.4 °C.[35] **^{1}H NMR (400 MHz, $CDCl_3$) δ (ppm) =** 8.07 (s, 1H), 7.91 (m, 2H), 7.60 (m, 2H), 7.45 (m, 2H), 7.36 (m, 1H), 6.80 (m, 2H), 3.04 (m, 6H). **^{13}C NMR (101 MHz, $CDCl_3$) δ (ppm) =** 150.8, 148.1, 130.7, 1299.0, 128.4, 126.9, 126.0, 122.1, 117.9, 112.5, 40.6.[35]

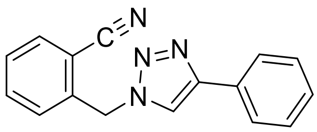

$C_{16}H_{12}N_4$
MW: 260.30000
CAS 1998733-55-3

2-((4-Phenyl-1H-1,2,3-triazol-1-yl)methyl)benzonitrile (3s): Prepared following the procedure B and purified by column chromatography (SiO_2; *n*-hexane:AcOEt 9:1 to 3:7, R_f = 0.05 in 10% AcOEt in *n*-hexane, R_f=0.33 in 30% AcOEt in *n*-hexane) and was isolated as a white solid, 218.6 mg (84.0% yield). **Mp:** 136.4 – 137.6 °C. **^{1}H NMR (400 MHz, $CDCl_3$) δ (ppm) =** 7.90 (s, 1H), 7.83 (m, 2H), 7.74 (ddd, J=7.7, 1.4, 0.6 Hz, 1H), 7.62 (td, J=7.8, 1.4 Hz, 1H), 7.48 (td, J=7.7, 1.2 Hz, 1H), 7.45 – 7.39 (m, 3H), 7.34 (m, 1H), 5.81 (s, 2H). **^{13}C NMR (101 MHz, $CDCl_3$) δ (ppm) =** 148.7, 138.4, 133.9, 133.2, 130.3, 129.8, 129.5, 129.0, 128.6, 125.9, 120.1, 117.2, 112.0, 51.9.[36]

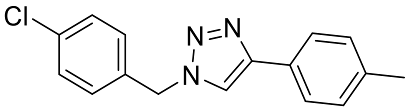

$C_{16}H_{14}ClN_3$
MW: 283.75900
CAS 1641553-42-5

1-(4-Chlorobenzyl)-4-(p-tolyl)-1H-1,2,3-triazole (3t): Prepared following the procedure B and purified by column chromatography (SiO_2; *n*-hexane:AcOEt 9:1 to 5:5, R_f = 0.06 in 10% AcOEt in *n*-hexane) and was isolated as a white solid, 228.61 mg (80.4% yield). **Mp**: 170.0 – 170.6 °C.[37] **^{1}H NMR (400 MHz, $CDCl_3$) δ (ppm) =** 7.69 (m, 2H), 7.62 (s, 1H), 7.36 (m, 2H), 7.19 – 7.25 (m, 4H), 5.54 (s, 2H), 2.37 (s, 3H). **^{13}C NMR (101 MHz, $CDCl_3$) δ (ppm) =** 148.6, 138.3, 135.0, 133.4, 129.7, 129.5, 127.7, 125.8, 53.6, 21.4.[38]

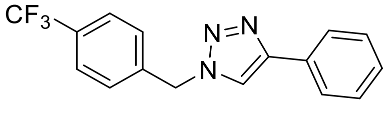

$C_{16}H_{12}F_3N_3$
MW: 303.28821
CAS 1252678-76-4

4-Phenyl-1-(4-(trifluoromethyl)benzyl)-1H-1,2,3-triazole (3u): Prepared following the procedure B and purified by column chromatography (SiO_2; *n*-hexane:AcOEt 9:1 to 3:4, R_f = 0.08 in 10% AcOEt in *n*-hexane) and was isolated as a white solid, 254.2 mg (83.8% yield). **Mp:** 139.2 – 139.8 °C.[39] **^{1}H NMR (400 MHz, $CDCl_3$) δ (ppm) =** 7.81 (m, 2H), 7.70 (s, 1H), 7.65 (d, J=8.1 Hz, 2H), 7.45 – 7.39 (m, 4H), 7.34 (m, 1H), 5.65 (s, 2H). **^{13}C NMR (101 MHz, $CDCl_3$) δ (ppm) =** 148.7, 138.8 (d, $^4J_{C-F}$=1.5 Hz), 131.2 (q, $^2J_{C-F}$=32.7 Hz), 130.4, 129.0, 128.5, 128.3, 126.3 (q, $^3J_{C-F}$=3.8 Hz), 125.9, 123.9 (q, $^1J_{C-F}$=272.3 Hz), 119.7, 53.7. **^{19}F NMR (376 MHz, $CDCl_3$) δ (ppm) =** -62.9. **^{19}F{^{1}H} NMR (376 MHz, $CDCl_3$) δ (ppm) =** -62.9.[39]

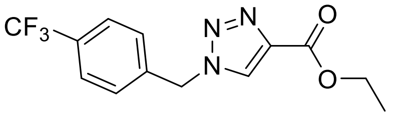

$C_{13}H_{12}F_3N_3O_2$
MW: 299.25321
CAS 2098091-44-0

Ethyl 1-(4-(trifluoromethyl)benzyl)-1H-1,2,3-triazole-4-carboxylate (3v): Prepared following the procedure B and purified by column chromatography (SiO_2; *n*-hexane:AcOEt 9:1 to 5:5, R_f=0.15 in 30% AcOEt in *n*-hexane) and was isolated as a slightly beige solid, 247.8 mg (82.8% yield). **Mp:** 136.1 – 136.8 °C. **^{1}H NMR (400 MHz, $CDCl_3$) δ (ppm) =** 8.02 (s, 1H), 7.66 (dt, J=8.1, 0.6 Hz, 2H), 7.40 (dt, J=8.0, 0.7 Hz, 2H), 5.65 (s, 2H), 4.41 (q, J=7.1 Hz, 2H), 1.39 (t, J=7.1 Hz, 3H). **^{13}C NMR (101 MHz, $CDCl_3$) δ (ppm) =** 160.7, 141.2, 137.8, 131.6 (q, J=32.9 Hz), 128.5, 127.5, 126.5 (q, J=3.7 Hz), 123.8 (q, J=274.6 Hz), 61.6, 53.9, 14.4. **^{19}F NMR (376 MHz, $CDCl_3$) δ (ppm) =** -63.0. **^{19}F{^{1}H} NMR (376 MHz, $CDCl_3$) δ (ppm) =** -63.0.[19]

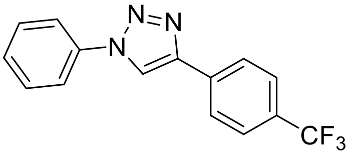

$C_{15}H_{10}F_3N_3$
MW: 289.26121
CAS 875312-72-4

1-Phenyl-4-(4-(trifluoromethyl)phenyl)-1H-1,2,3-triazole (3w): Flow stream containing 0.0250 M solution of azide (1.0 eq.) and 0.0325 M solution of alkyne (1.3 eq.) in DCM was directed with 0.75 mL·min^{-1} to the catalytic column (stainless steel Restek 4.6 mm ID x 150 mm column filled with 860±7 mg Cu/C, total volume of 2.49 mL, effective volume of 1.61 mL, 1.01 mmol Cu per 1.0 g of Cu/C, 0.869±0.007 mmol Cu) submerged in an oil bath at 110 °C. Additional cooling coil (1m, 1.00 mm ID, SS) submersed in water (at approx. 23 °C) was placed after the reactor to cool down reaction mixture. IR measurement cell was placed after the BPR (250 psi). Flow equipment was controlled with python script. Reaction mixture was passed through Cu/C catalytic columns for 800 s resulting in elution of 10.0 mL of reaction mixture. Solvent was removed from collected reaction mixture and obtained solid residue was dried *in vacuo*. Desired product was obtained as a white solid, 69.4 mg (96.0% yield). **Mp:** 232.5 – 232.9 °C, lit. 231.0 °C.[40] **^{1}H NMR (400 MHz, $CDCl_3$) δ (ppm) =** 8.29 (br s, 1H), 8.05 (d, J=8.1 Hz, 2H), 7.81 (m, 2H), 7.73 (d, J=8.0 Hz, 2H), 7.58 (m, 2H), 7.49 (m, 1H).[41] **^{13}C NMR (101 MHz, $CDCl_3$) δ (ppm) =** not recorded due to low solubility. **^{19}F NMR (376 MHz, $CDCl_3$) δ (ppm) =** -62.7. **^{19}F{^{1}H} NMR (376 MHz, $CDCl_3$) δ (ppm) =** -62.7.

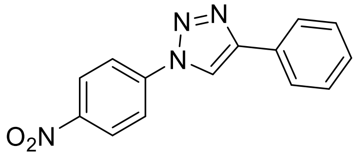

$C_{14}H_{10}N_4O_2$
MW: 266.26
CAS 2101-57-7

1-(4-Nitrophenyl)-4-phenyl-1*H*-1,2,3-triazole (3x): Copper-on-Charcoal (76.0 mg, 0.077 mmol Cu, 5 mol%) was added to a flask fitted with a stir bar and septum, then 1,4-dioxane (3.1 mL, 12.5 vol.) was added slowly to the sidewalls of the flask to rinse the catalyst down. While the heterogeneous solution is stirred, triethylamine (169.6 mg, 1.7 mmol, 1.1 eq.), phenylacetylene (171.1 mg, 1.7 mmol, 1.1 eq.), and 1-azido-4-nitrobenzene (250.0 mg, 1.5 mmol, 1.0 eq.) were added. Reaction mixture was then

heated to 60 °C and the reaction progress was monitored by TLC until complete consumption of azide has occurred. The mixture was then filtered through a pad of silica to remove the catalyst, and the filter cake was further washed with EtOAc to ensure complete transfer. The volatiles were removed in vacuo to give 254.3 mg (62.7% yield) of pure triazole. **Mp:** 253.0 – 254.1 °C, lit. 254 – 255 °C [42]; **^{1}H NMR (400 MHz, DMSO-d$_6$) δ (ppm) =** 9.54 (s, 1H), 8.51 (m, 2H), 8.27 (m, 2H), 7.97 (m, 2H), 7.53 (m, 2H), 7.42 (m, 1H). **^{13}C NMR (101 MHz, DMSO-d$_6$) δ (ppm) =** 120.0, 120.5, 125.4, 125.7, 128.6, 129.1, 129.8, 140.9, 146.7, 147.8.[42]

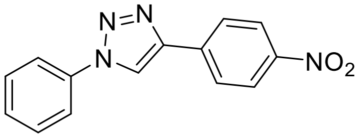

$C_{14}H_{10}N_4O_2$
MW: 266.26
CAS 68809-43-8

1-Phenyl-4-(4-nitrophenyl)-1*H*-1,2,3-triazole (3y): Copper-on-Charcoal (70.1 mg, 0.071 mmol Cu, 5 mol%) was added to a flask fitted with a stir bar and septum, then 1,4-dioxane (3.4 mL, 20.0 vol.) was added slowly to the sidewalls of the flask to rinse the catalyst down. While the heterogeneous solution is stirred, triethylamine (157.5 mg, 1.6 mmol, 1.1 eq.), 4-nitrophenylacetylene (229.1 mg, 1.6 mmol, 1.1 eq.), and phenyl azide (168.6 mg, 1.4 mmol, 1.0 eq.) were added. Reaction mixture was then heated to 60 °C and the reaction progress was monitored by TLC until complete consumption of azide has occurred. The mixture was then filtered through a pad of silica to remove the catalyst, and the filter cake was further washed with EtOAc to ensure complete transfer. The volatiles were removed in vacuo to give 212.3 mg (56.3% yield) of pure triazole. **Mp:** 249.7 – 250.9 °C, lit. 247 – 249 °C [43]; **^{1}H NMR (400 MHz, DMSO-d$_6$) δ (ppm) =** 9.58 (s, 1H), 8.40 (m, 2H), 8.23 (m, 2H), 7.97 (m, 2H), 7.76 (m, 1H), 7.66 (m, 2H), 7.56 (m, 1H). **^{13}C NMR (101 MHz, DMSO-d$_6$) δ (ppm) =** 146.9, 145.4, 136.7, 133.0, 130.0, 1429.1, 126.1, 124.5, 121.8, 120.2.[44]

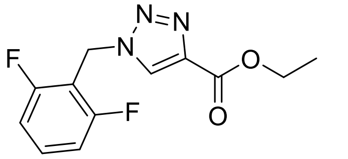

$C_{12}H_{11}F_2N_3O_2$
MW: 267.2358
CAS 869501-51-9

1-(2,6-Difluorobenzyl)-1*H*-1,2,3-triazolyl-4-carboxylic acid ethyl ester (3z): Prepared following the procedure B and purified by column chromatography (SiO_2; AcOEt:*n*-hexane 1:9, R_f = 0.09 in AcOEt:*n*-hexane 1:9) and was isolated as a white solid, 237.1 mg (88.7% yield). **Mp:** 114.6 – 114.9 °C; **^{1}H NMR (400 MHz, $CDCl_3$) δ (ppm) =** 8.08 (s, 1H), 7.40 (dd, $^{3}J_{H\text{-}H}$=8.5, $^{4}J_{H\text{-}F}$=6.5 Hz, 1H), 6.99 (m, 2H), 5.68 (t, $^{4}J_{H\text{-}F}$=5.7 Hz, 2H), 4.40 (q, $^{3}J_{H\text{-}H}$=7.1 Hz, 2H), 1.38 (t, $^{3}J_{H\text{-}H}$=7.1 Hz, 3H). **^{13}C NMR (101 MHz, $CDCl_3$) δ (ppm) =** 161.5 (dd, $^{1}J_{C\text{-}F}$=251.7, $^{3}J_{C\text{-}F}$=6.8 Hz, C), 160.8 (s, C), 140.7 (s, C), 132.0 (t, $^{3}J_{C\text{-}F}$=10.4 Hz, CH), 127.5 (t, $J_{C\text{-}F}$=1.7 Hz, CH), 112.1 (dd, $^{2}J_{C\text{-}F}$=19.2, $^{4}J_{C\text{-}F}$=5.7 Hz, CH), 110.2 (t, $^{2}J_{C\text{-}F}$=18.9 Hz, C), 61.5 (s, CH_2), 41.8 (t, $^{3}J_{C\text{-}F}$=4.1 Hz, CH_2), 14.4 (s, CH_3). **^{19}F NMR (376 MHz, $CDCl_3$) δ (ppm) =** -114.0 (t, J=6.9 Hz). **^{19}F{^{1}H} NMR (376 MHz, $CDCl_3$) δ (ppm) =** -114.1. **^{1}H NMR (400 MHz, DMSO-d$_6$) δ (ppm) =** 8.83 (br s, 1H), 7.52 (tt, $^{3}J_{H\text{-}H}$=8.5, $^{4}J_{H\text{-}F}$=6.7 Hz, 1H), 7.19 (m, 2H), 5.74 (t, $^{4}J_{H\text{-}F}$=1.15 Hz, 2H), 4.30 (q, $^{3}J_{H\text{-}H}$=7.1 Hz. 2H), 1.29 (t, $^{3}J_{H\text{-}H}$=7.1 Hz, 3H). **^{13}C NMR (101 MHz, DMSO-d$_6$) δ (ppm) =** 160.8 (dd, $^{1}J_{C\text{-}F}$=249.5, $^{3}J_{C\text{-}F}$=7.3 Hz, C), 160.1 (s, C), 138.7 (s, C), 131.9 (t, $^{3}J_{C\text{-}F}$=10.5 Hz, CH), 129.4 (s, CH), 112.0 (dd, $^{2}J_{C\text{-}F}$=18.9, $^{4}J_{C\text{-}F}$=5.8 Hz, CH), 110.8 (t, $^{2}J_{C\text{-}F}$=19.0 Hz, C), 60.6 (s, CH_2), 41.3 (t, $^{3}J_{C\text{-}F}$=3.8 Hz, CH_2), 14.1 (s, CH_3).[45] **^{19}F NMR (376 MHz, DMSO-d$_6$) δ (ppm) =** -114.6 (t, J=7.1 Hz). **^{19}F{^{1}H} NMR (376 MHz, DMSO-d$_6$) δ (ppm) =** -114.6. **UV-Vis (DCM) $λ_{max}$ (ε):** 262 nm (867 M^{-1} cm^{-1}). **FT-ATR-IR IR** ν (cm^{-1}) = 3144, 3089, 3019, 2985, 2942, 2902, 1722, 1626, 1592, 1522, 1468, 1445, 1378, 1350, 1209, 1198, 1157, 1137, 1104, 1045, 1013, 991, 893, 862, 793, 779, 764, 669, 678, 543. **LR-ESI-MS m/z:** 268.2016 (M + H^+); 557.3250 (2M + Na^+) **HR-ESI-MS m/z:**

222.0476 (M - EtO), 290.0716 (M + Na^+), 557.1527 (2M + Na^+); calcd for $C_{10}H_6F_2N_3O$ 222.0479, for $C_{12}H_{11}F_2N_3O_2$ 267.0819, for $C_{12}H_{11}F_2N_3NaO_2$ 290.0717, for $C_{24}H_{22}F_4N_6NaO_4$ 557.1536.

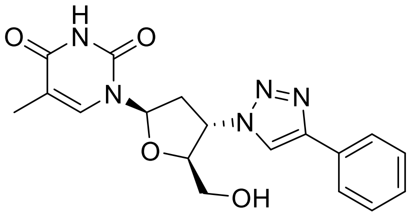

$C_{18}H_{19}N_5O_4$
MW: 369.38100
CAS 127728-29-4

3′-Deoxy-3′-(4-phenyl-1H-1,2,3-triazol-1-yl)thymidine, 1-((2*R*,4*S*,5*S*)-5-(hydroxymethyl)-4-(4-phenyl-1*H*-1,2,3-triazol-1-yl)tetrahydrofuran-2-yl)-5-methylpyrimidine-2,4(1*H*,3*H*)-dione (3aa):

Flow stream containing 0.10 M solution of azide (1.0 eq.) and 0.13 M solution of alkyne (1.3 eq.) in EtOH was directed with 0.75 mL·min^{-1} to the catalytic columns (stainless steel Restek 4.6 mm ID x 150 mm column filled with 860±7 mg Cu/C, total volume of 2.49 mL, effective volume of 1.61 mL, 1.01 mmol Cu per 1.0 g of Cu/C, 0.869±0.007 mmol Cu) submerged in an oil bath at 110 °C. Additional cooling coil (1m, 1.00 mm ID, SS) submersed in water (at approx. 23 °C) was placed after the reactor to cool down reaction mixture. IR measurement cell was placed after the BPR (250 psi). Flow equipment was controlled with python script. Reaction mixture was passed through Cu/C catalytic columns for 800 s resulting in elution of 10.0 mL of reaction mixture. Reaction mixture was collected, solvent was then removed and obtained solid residue was preadsorbed onto silica and purified by column chromatography (SiO_2; *n*-hexane:AcOEt:MeOH 7:3:0 to 3:5:2 v/v, R_f=0.11 in 70% AcOEt in *n*-hexane, R_f=0.43 in *n*-hexane:AcOEt:MeOH 3:6:1) and was isolated as a white solid, 286.7 mg (77.6% yield). **Mp:** 236.0 – 236.4 °C, lit. 232.0 – 234.0 °C.[46] **^{1}H NMR (400 MHz, DMSO-d$_6$) δ (ppm) =** 11.36 (br s, 1H), 8.78 (s, 1H), 7.87 (m, 1H), 7.84 (m, 2H), 7.47 (m, 2H), 7.35 (m, 1H), 6.45 (t, J=6.6 Hz, 1H), 5.41 (dt, J=8.6, 5.4 Hz, 1H), 5.30 (t, J=5.0 Hz, 1H), 4.29 (dt, J=5.5, 3.6 Hz, 1H), 3.74 (dt, J=11.3, 3.7 Hz, 1H), 3.67 (dt, J=12.0, 4.0 Hzf, 1H), 2.81 (ddd, J= 13.9, 6.7, 5.4 Hz, 1H), 2.70 (ddd, J=14.0, 8.7, 6.5 Hz, 1H), 1.82 (d, J=1.2 Hz, 3H). **^{13}C NMR (101 MHz, DMSO-d$_6$) δ (ppm) =** 163.7 (C), 150.4 (C), 146.6 (C), 136.3 (CH), 130.6 (C), 128.9 (CH), 128.0 (CH), 125.2 (CH), 121.0 (CH), 109.3 (C), 84.4 (CH), 83.9 (CH), 60.8 (CH_2), 59.4 (CH), 37.1 (CH_2), 12.3 (CH_3).[47] **LR-ESI-MS m/z:** 370.4 (M + H^+), 392.4 (M + Na^+), 739.7 (2M + H^+), 761.7 (2M + Na^+), 783.4 (2M + 2Na^+).

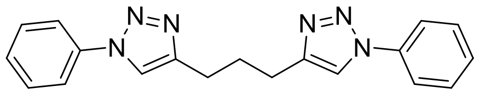

$C_{19}H_{18}N_6$
MW: 330.40
CAS 1947347-94-5

1,3-Bis(1-phenyl-1*H*-1,2,3-triazol-4-yl)propane (3ab):

Increased reaction time: Flow stream containing 0.10 M solution of azide (1.0 eq.) and 0.07 M solution of alkyne (0.7 eq.) in DCM was directed with 0.40 mL·min^{-1} to the catalytic columns (stainless steel Restek 4.6 mm ID x 150 mm column filled with 860±7 mg Cu/C, total volume of 2.49 mL, effective volume of 1.61 mL, 1.01 mmol Cu per 1.0 g of Cu/C, 0.869±0.007 mmol Cu) submerged in an oil bath at 110 °C. Additional cooling coil (1m, 1.00 mm ID, SS) submersed in water (at approx. 23 °C) was placed after the reactor to cool down reaction mixture. IR measurement cell was placed after the BPR (250 psi). Flow equipment was controlled with python script. Reaction mixture was passed through Cu/C catalytic columns for 1500 s resulting in elution of 10.0 mL of reaction mixture. Solvent was removed from collected reaction mixture and obtained residue was purified by column chromatography (SiO_2; AcOEt:*n*-hexane 1:9 to 9:1, R_f=0.20 in AcOEt:*n*-hexyne 4:6) and was isolated as a white solid, 70.2 mg (42.5% yield).

Increased temperature and residence time: Flow stream containing 0.10 M solution of azide (1.0 eq.) and 0.07 M solution of alkyne (0.7 eq.) in DCM was directed with 0.50 mL·min^{-1} to the catalytic columns (stainless steel Restek 4.6 mm ID x 150 mm column filled with 860±7 mg Cu/C, total volume of 2.49 mL, effective volume of 1.61 mL, 1.01 mmol Cu per 1.0 g of Cu/C, 0.869±0.007 mmol Cu) submerged in an oil bath at 120 °C. Additional cooling coil (1m, 1.00 mm ID, SS) submersed in water (at approx. 23 °C) was placed after the reactor to cool down reaction mixture. IR measurement cell was placed after the BPR (250 psi). Flow equipment was controlled with python script. Reaction mixture was passed through Cu/C catalytic columns for 1200 s resulting in elution of 10.0 mL of reaction mixture. Solvent was removed from collected reaction mixture and obtained residue was purified by column chromatography (SiO_2; AcOEt:*n*-hexane 1:9 to 9:1, R_f=0.20 in AcOEt:*n*-hexyne 4:6) and was isolated as a white solid, 90.5 mg (54.8% yield).

Mp: 138.0 – 139.1 °C; **^{1}H NMR (400 MHz, CDCl$_3$) δ (ppm) =** 7.79 (s, 2H), 7.72 (m, 4H), 7.51 (m, 4H), 7.42 (m, 2H), 2.93 (t, J=7.4 Hz, 4H), 2.22 (q, J=7.4 Hz, 2H). **^{13}C NMR (101 MHz, CDCl$_3$) δ (ppm) =** 148.4 (C), 137.4 (C), 129.8 (CH), 128.6 (CH), 120.6 (CH), 119.4 (CH), 29.1 (CH_2), 25.1 (CH_2).[48]

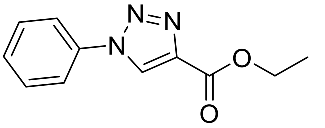

$C_{11}H_{11}N_3O_2$
MW: 217.2280
CAS 4915-97-3

1-Phenyl-1*H*-1,2,3-triazolyl-4-carboxylic acid ethyl ester (3ac): Prepared following the procedure B and purified by column chromatography (SiO_2; AcOEt:*n*-hexane 1:9 to 2:8, R_f = 0.05 in AcOEt:*n*-hexane 1:9) and was isolated as a white solid, 194.2 mg (89.4% yield). **Mp:** 86.0 – 86.7 °C [49]; **^{1}H NMR (400 MHz, CDCl$_3$) δ (ppm) =** 8.50 (s, 1H), 7.76 (m, 2H), 7.57 (m, 2H), 7.50 (m, 1H), 4.48 (q, J=7.1 Hz, 2H), 1.45 (t, J=7.1 Hz, 3H). **^{13}C NMR (101 MHz, CDCl$_3$) δ (ppm) =** 160.8, 141.1, 136.6, 130.1, 129.7, 125.6, 121.0, 61.7, 14.5.[50]

$C_{35}H_{51}N_3$
MW: 513.81400
CAS 1357271-66-9

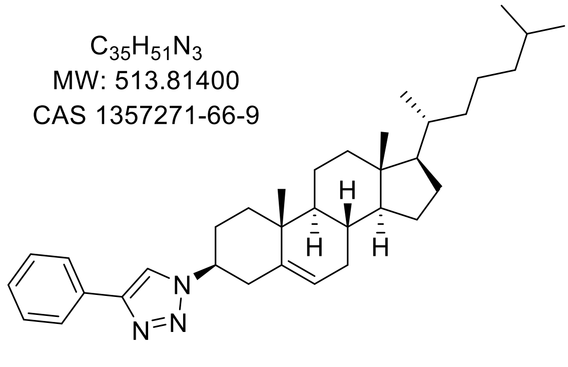

1-(3β)-Cholest-5-en-3-yl-4-phenyl-1*H*-1,2,3-triazole, 1-((3*S*,8*S*,9*S*,10*R*,13*R*,14*S*,17*R*)-10,13-dimethyl-17-((*R*)-6-methylheptan-2-yl)-2,3,4,7,8,9,10,11,12,13,14,15,16,17-tetradecahydro-1*H*-cyclopenta[*a*]phenanthren-3-yl)-4-phenyl-1*H*-1,2,3-triazole (3ad): Prepared following the procedure B and purified by column chromatography (SiO_2; *n*-hexane:AcOEt 9:1, R_f=0.36 in 10% AcOEt in *n*-hexane) and was isolated as a white, cloudy solid, 277.7 mg (54.0% yield). **Mp:** 233.9 – 234.5 °C, lit. 200.0 °C.[51] **^{1}H NMR (400 MHz, CDCl$_3$) δ (ppm) =** 7.83 (m, 2H), 7.78 (s, 1H), 7.42 (m, 2H), 7.32 (m, 1H), 5.48 (dt, J=5.5, 2.0 Hz, 1H), 4.43 (m, 1H), 2.82 (m, 1H), 2.60 (ddd, J=13.5, 4.4, 2.0 Hz, 1H), 2.20 – 2.09 (m, 2H), 2.09 – 2.00 (m, 3H), 1.85 (m, 1H), 1.57 – 1.43 (m, 5H), 1.38 – 0.99 (m, 18H), 0.93 (d, J=6.5 Hz, 3H), 0.87 (dd, J=6.6, 1.8 Hz, 6H), 0.7 (s, 3H). **^{13}C NMR (101 MHz, CDCl$_3$) δ (ppm) =** 147.5 (C), 139.4 (C), 131.0 (C), 129.0 (CH), 128.1 (CH), 125.8 (CH), 123.5 (CH), 117.5 (CH), 61.1 (CH), 56.84 (CH), 56.3 (CH), 50.2 (CH), 42.5 (C), 39.9 (CH_2), 39.8 (CH_2), 39.7 (CH_2), 38.0 (CH_2), 36.9 (C), 36.3 (CH_2), 35.9 (CH), 32.0

(CH_2), 32.0 (CH), 29.5 (CH_2), 28.4 (CH_2), 28.2 (CH), 24.4 (CH_2), 24.0 (CH_2), 23.0 (CH_3), 22.7 (CH_3), 21.2 (CH_2), 19.6 (CH_3), 18.9 (CH_3), 12.0 (CH_3).[51] **LR-ESI-MS m/z:** 514.6 ($M + H^+$).

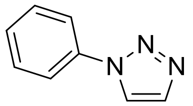

$C_8H_7N_3$
MW: 145.17
CAS 1453-81-2

1-Phenyl-1*H*-1,2,3-triazole (3ae): Prepared following the procedure B and purified by column chromatography (SiO_2; *n*-hexane:AcOEt 9:1 to 5:5, R_f = 0.10 in 10% AcOEt in *n*-hexane) and was isolated as a white solid, 143.2 mg (98.6% yield). **Mp:** 53.4 – 54. 1 °C, lit. 53 – 54 °C [52]; **^{1}H NMR (400 MHz, $CDCl_3$) δ (ppm) =** 8.00 (d, J=1.1 Hz, 1H), 7.85 (d, J=1.1 Hz, 1H), 7.75 (m, 2H), 7.54 (m, 2H), 7.45 (m, 1H). **^{13}C NMR (101 MHz, $CDCl_3$) δ (ppm) =** 137.2, 134.6, 129.9, 128.9, 121.8, 120.8.

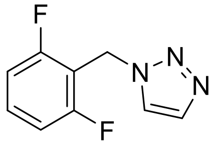

$C_9H_7F_2N_3$
MW: 195.17
CAS 1438893-75-4

1-(2,6-Difluorobenzyl)-1*H*-1,2,3-triazole (3af): Prepared following the procedure B and purified by column chromatography (SiO_2; *n*-hexane:AcOEt 8:2 to 5:5, R_f = 0.54 in AcOEt:*n*-hexane 8:2) and was isolated as a creme solid, 188.0 mg (96.3% yield). **Mp:** 75.0 – 75.4 °C; **^{1}H NMR (400 MHz, $CDCl_3$) δ (ppm)** = 7.69 (d, J=1.1 Hz, 1H), 7.60 (d, J=0.9 Hz, 1H), 7.36 (tt, $^3J_{H\text{-}H}$=8.4 Hz, $^4J_{H\text{-}F}$=6.5 Hz, 1H), 6.97 (m, 2H), 5.66 (t, $^4J_{H\text{-}F}$=1.3 Hz, 2H). **^{13}C NMR (101 MHz, $CDCl_3$) δ (ppm) =** 161.6 (dd, $^1J_{C\text{-}F}$=251.3 Hz, $^3J_{C\text{-}F}$=6.9 Hz), 134.2 (s), 131.6 (t, $^2J_{C\text{-}F}$=10.3 Hz), 123.5 (s), 112.0 (m), 111.1 (t, $^2J_{C\text{-}F}$=19.0 Hz), 41.3 (t, $^3J_{C\text{-}F}$=4.0 Hz).[53] **^{19}F{^{1}H} NMR (376 MHz, $CDCl_3$) δ (ppm) =**-114.3.

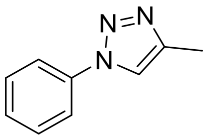

$C_9H_9N_3$
MW: 159.19
CAS 20320-20-1

4-Methyl-1-phenyl-1*H*-1,2,3-triazole (3ag): Prepared following the procedure B and purified by column chromatography (SiO_2; AcOEt:*n*-hexane 1:9 to 4:6, R_f = 0.17 in AcOEt:*n*-hexane 2:8) and was isolated as a white solid, 131.0 mg (82.3% yield). **Mp:** 81.1 – 81.8 °C, lit. 80 – 81 °C [54]; **^{1}H NMR (400 MHz, $CDCl_3$) δ (ppm) =** 7.74 – 7.68 (m, 3H), 7.51 (m, 2H), 7.41 (m, 1H), 2.44 (s, 3H). **^{13}C NMR (101 MHz, $CDCl_3$) δ (ppm) =** 144.2, 137.4, 129.8, 128.6, 120.6, 119.5, 11.0.[55]

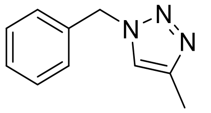

$C_{10}H_{11}N_3$
MW: 173.22
CAS 91258-00-3

1-Benzyl-4-methyl-1*H*-1,2,3-triazole (3ah): Prepared following the procedure B and purified by column chromatography (SiO_2; AcOEt:*n*-hexane 1:9 to 6:4, R_f=0.24 in AcOEt:*n*-hexane 4:6) and was isolated as a white solid, 176.9 mg (82.2% yield). **Mp:** 64.0 – 64.7 °C, lit. 64 – 65 °C [56]; **^{1}H NMR (400 MHz, $CDCl_3$) δ (ppm)** = 7.36 – 7.27 (m, 3H), 7.25 – 7.18 (m, 2H), 7.15 (q, J=0.8 Hz, 1H), 5.44 (t, J=0.6 Hz, 2H), 2.28 (d, J=0.8 Hz, 3H). **^{13}C NMR (101 MHz, $CDCl_3$) δ (ppm) =** 149.9 (C), 135.1 (C), 129.2 (CH), 128.7 (CH), 128.1 (CH), 121.2 (CH), 54.1 (CH_2), 11.0 (CH_3).[56]

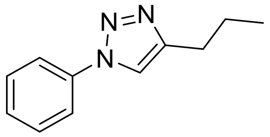

$C_{11}H_{13}N_3$
MW: 187.2460
CAS 1258275-35-2

1-Phenyl-4-propyl-1*H*-1,2,3-triazole (3ai): Prepared following the procedure B and purified by column chromatography (SiO_2; AcOEt:*n*-hexane 1:9 to 3:7, R_f=0.06 in AcOEt:*n*-hexane 1:9) and was isolated as a white solid, 155.4 mg (83.0% yield). **Mp:** 47.0 – 47.9 °C, lit. 47 – 48 °C [57]; **^{1}H NMR (400 MHz, $CDCl_3$) δ (ppm) =** 7.72 (m, 3H), 7.51 (m, 2H), 7.41 (m, 1H), 2.78 (td, J=7.7 Hz, J=0.7 Hz, 2H), 1.77 (tq, J=7.7 Hz, J=7.4 Hz, 2H), 1.02 (t, J=7.4 Hz, 3H). **^{13}C NMR (101 MHz, $CDCl_3$) δ (ppm) =** 149.1, 137.5, 129.8, 128.6, 120.6, 119.0, 27.8, 22.8, 14.0.[57]

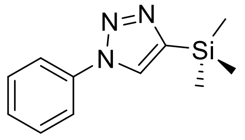

$C_{11}H_{15}N_3Si$
MW: 217.3470
CAS 138801-84-0

1-Phenyl-4-(trimethylsilyl)-1*H*-1,2,3-triazole (3aj): Prepared following the procedure B and purified by column chromatography (SiO_2; AcOEt:*n*-hexane 1:9 to 3:7, R_f = 0.41 in AcOEt:*n*-hexane 4:6) and was isolated as a white solid, 20.0 mg (9.2% yield). **Mp:** 89.7 – 90.4 °C, lit. 89 – 90 °C [58]; **^{1}H NMR (400 MHz, $CDCl_3$) δ (ppm) =** 7.94 (s, 1H), 7.74 (m, 2H), 7.51 (m, 2H), 7.42 (m, 1H), 0.38 (s, 9H). **^{13}C NMR (101 MHz, $CDCl_3$) δ (ppm) =** 147.5, 137.3, 129.8, 128.6, 127.3, 121.0, -1.0.[59]

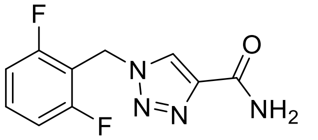

$C_{10}H_8F_2N_4O$
MW: 238.1978
CAS 106308-44-5

Rufinamide, 1-(2,6-difluorobenzyl)-1*H*-1,2,3-triazole-4-carboxamide (4): Flow stream containing 0.10 M solution of azide (1.0 eq.) and 0.13 M solution of alkyne (1.3 eq.) in DMSO was directed with 0.75 mL·min^{-1} to the catalytic columns (stainless steel Restek 4.6 mm ID x 150 mm column filled with 860±7 mg Cu/C, total volume of 2.49 mL, effective volume of 1.61 mL, 1.01 mmol Cu per 1.0 g of Cu/C, 0.869±0.007 mmol Cu) submerged in an oil bath at 110 °C. Additional cooling coil (1m, 1.00 mm ID, SS) submersed in water (at approx. 23 °C) was placed after the reactor to cool down reaction mixture. IR measurement cell was placed after the BPR (250 psi). Flow equipment was controlled with python script. Reaction mixture was passed through Cu/C catalytic columns for 800 s resulting in elution of 10.0 mL of reaction mixture. Solvent was removed from collected reaction mixture and obtained solid residue was washed with water, collected, and dried *in vacuo*. Rufinamide was obtained as a beige solid, 227.6 mg (95.6% yield). **Mp:** 241.1 – 242.6 °C, lit. 241 – 243 °C [60]; **^{1}H NMR (400 MHz, DMSO-d_6) δ (ppm) =** 8.54 (s, 1H), 7.83 (br s, 1H), 7.52 (tt, $^3J_{H-H}$=8.5 Hz, $^4J_{H-F}$=6.7 Hz, 1H), 7.46 (br s, 1H), 7.19 (m, 2H), 5.72 (s, 2H). **^{19}F NMR (376 MHz, DMSO-d_6) δ (ppm) =** -114.3 (t, J=7.1 Hz). **^{19}F{^{1}H} NMR (376 MHz, DMSO-d_6) δ (ppm) =** -114.3. **^{13}C NMR (101 MHz, DMSO-d_6) δ (ppm) =** 161.3 (s), 160.8 (td, $^1J_{C-F}$=249.4 Hz, $^3J_{C-F}$=7.4 Hz), 142.8 (s), 131.8 (t, $^3J_{C-F}$=10.5 Hz), 126.8 (s), 112.0 (dd, $^2J_{C-F}$=18.9 Hz, $^4J_{C-F}$=5.8 Hz), 111.0 (t, $^2J_{C-F}$=19.2 Hz), 41.2 (t, $^3J_{C-F}$=4.0 Hz).[8]

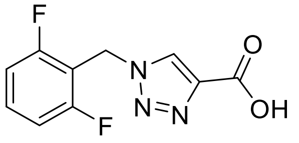

$C_{10}H_7F_2N_3O_2$
MW: 239.1818
CAS 166196-11-8

1-(2,6,-Difluorobenzyl)-1H-1,2,3-triazole-4-carboxylic acid: A mixture of ethyl 1-(2,6-difluorobenzyl)-1H-1,2,3-triazolyl-4-carboxylate (111.6 mg, 0.42 mmol, 1.0 eq.) and lithium hydroxide monohydrate (52.6 mg, 1.25 mmol, 3.0 eq.) in THF:H2O 1:1 (1.0:1.0 mL, 17.0 vol.) was stirred at 50 °C for 24h. THF was then distilled off, pH of the mixture was adjusted to 1 and white solid precipitated out. Solid was filtered off, washed with cold water and dried under vacuum. 1-(2,6-Difluorobenzyl)-1H-1,2,3-triazole-4-carboxylic acid was obtained as 66.2 mg of white solid (66.3% yield). **^{1}H NMR (400 MHz, DMSO-d_6)**

δ (ppm) = 13.14 (br s, 1H), 8.73 (s, 1H), 7.52 (tt, $^3J_{H-H}$=8.5, $^4J_{H-F}$=6.7 Hz, 1H), 7.18 (m, 2H), 5.72 (s, 2H). **^{13}C NMR (101 MHz, DMSO-d_6) δ (ppm) =** 161.5 (s, C), 160.8 (dd, $^1J_{C-F}$=249.5, $^3J_{C-F}$=7.4 Hz, C), 139.6 (s, C), 131.9 (t, $^3J_{C-F}$=10.4 Hz, CH), 129.2 (s, CH), 112.0 (m, CH), 110.9 (t, $^2J_{C-F}$=19.1 Hz, C), 41.2 (t, $^3J_{C-F}$=3.8 Hz, CH). **^{19}F NMR (376 MHz, DMSO-d_6) δ (ppm) =** -114.6 (t, J=7.1 Hz). **^{19}F{^{1}H} NMR (376 MHz, DMSO-d_6) δ (ppm) =** -114.6.[61]

5. Copies of NMR, UV-Vis, FT-ATR-IR and MS spectra of 1,2,3-triazoles:

Figure S 12. ^{1}H NMR spectrum of 1,4-diphenyl-1H-1,2,3-triazole **3a**. 29
Figure S 13. ^{13}C NMR spectrum of 1,4-diphenyl-1H-1,2,3-triazole **3a**. 29
Figure S 14. ^{1}H NMR spectrum of 1-phenyl-4-(4-tert-butyl)phenyl-1H-1,2,3-triazole **3b**. 30
Figure S 15. ^{13}C NMR spectrum of 1-phenyl-4-(4-tert-butyl)phenyl-1H-1,2,3-triazole **3b**. 30
Figure S 16. ^{1}H NMR spectrum of 1-naphthalenyl-4-phenyl-1H-1,2,3-triazole **3c**. 31
Figure S 17. ^{13}C NMR spectrum of 1-naphthalenyl-4-phenyl-1H-1,2,3-triazole **3c**. 31
Figure S 18. ^{1}H NMR spectrum of 1-(1-phenyl-1H-1,2,3-triazol-4-yl)ethan-1-ol **3d**. 32
Figure S 19. ^{13}C NMR spectrum of 1-(1-phenyl-1H-1,2,3-triazol-4-yl)ethan-1-ol **3d**. 32
Figure S 20. ^{1}H NMR spectrum of 4-(1-phenyl-1H-1,2,3-triazol-4-yl)butan-1-ol **3e**. 33
Figure S 21. ^{13}C NMR spectrum of 4-(1-phenyl-1H-1,2,3-triazol-4-yl)butan-1-ol **3e**. 33
Figure S 22. ^{1}H NMR spectrum of (1-phenyl-1H-1,2,3-triazol-4-yl)methyl benzoate **3f**. 34
Figure S 23. ^{13}C NMR spectrum of (1-phenyl-1H-1,2,3-triazol-4-yl)methyl benzoate **3f**. 34
Figure S 24. ^{1}H NMR spectrum of 4-octyl-1-phenyl-1H-1,2,3-triazole **3g**. 35
Figure S 25. ^{13}C NMR spectrum of 4-octyl-1-phenyl-1H-1,2,3-triazole **3g**. 35
Figure S 26. ^{1}H NMR spectrum of 4-(((tert-butyldimethylsilyl)oxy)methyl)-1-phenyl-1H-1,2,3-triazole **3h**. 36
Figure S 27. ^{13}C NMR spectrum of 4-(((tert-butyldimethylsilyl)oxy)methyl)-1-phenyl-1H-1,2,3-triazole **3h**. 36
Figure S 28.FT-ATR-IR spectrum of 4-(((tert-butyldimethylsilyl)oxy)methyl)-1-phenyl-1H-1,2,3-triazole **3h**. 37
Figure S 29. UV-Vis spectrum of 6.84E-5 M 4-(((tert-butyldimethylsilyl)oxy)methyl)-1-phenyl-1H-1,2,3-triazole **3h** in DCM. 37
Figure S 30. UV-Vis spectra of 4-(((tert-butyldimethylsilyl)oxy)methyl)-1-phenyl-1H-1,2,3-triazole **3h** in DCM. Concentrations of measured samples are described in the legend and are in mol·dm^{-3}. Note that the region between 200 and 230 nm is saturated due to solvent absorption. 38
Figure S 31. Absorption vs concentration graph of 4-(((tert-butyldimethylsilyl)oxy)methyl)-1-phenyl-1H-1,2,3-triazole **3h** for determination of molar extinction coefficient using Beer-Lambert law. 38
Figure S 32. LR-ESI-MS spectrum of 4-(((tert-butyldimethylsilyl)oxy)methyl)-1-phenyl-1H-1,2,3-triazole **3h**. 39
Figure S 33. HR-ESI-MS spectrum of 4-(((tert-butyldimethylsilyl)oxy)methyl)-1-phenyl-1H-1,2,3-triazole **3h**. 39
Figure S 34. ^{1}H NMR spectrum of 1-benzyl-4-phenyl-1H-1,2,3-triazole **3i**. 40
Figure S 35. ^{13}C NMR spectrum of 1-benzyl-4-phenyl-1H-1,2,3-triazole **3i**. 40
Figure S 36. ^{1}H NMR spectrum of 1-cyclohexyl-4-phenyl-1H-1,2,3-triazole **3j**. 41
Figure S 37. ^{13}C NMR spectrum of 1-cyclohexyl-4-phenyl-1H-1,2,3-triazole **3j**. 41
Figure S 38. ^{1}H NMR spectrum of 1-butyl-4-phenyl-1H-1,2,3-triazole **3k**. 42
Figure S 39. ^{13}C NMR spectrum of 1-butyl-4-phenyl-1H-1,2,3-triazole **3k**. 42
Figure S 40. ^{1}H NMR spectrum of 1-cyclohexyl-4-octyl-1H-1,2,3-triazole **3l**. 43
Figure S 41. DEPT-135 NMR spectrum of 1-cyclohexyl-4-octyl-1H-1,2,3-triazole **3l**. 43
Figure S 42. ^{13}C NMR spectrum of 1-cyclohexyl-4-octyl-1H-1,2,3-triazole **3l**. 44
Figure S 43. HSQC (^{1}H-^{13}C) NMR spectrum of 1-cyclohexyl-4-octyl-1H-1,2,3-triazole **3l**. 44
Figure S 44. FT-ATR-IR spectrum of 1-cyclohexyl-4-octyl-1H-1,2,3-triazole **3l**. 45
Figure S 45. UV-Vis absorption spectrum of 2.52E-3 M 1-cyclohexyl-4-octyl-1H-1,2,3-triazole **3l** in DCM. 45
Figure S 46. LR-ESI-MS spectrum of 1-cyclohexyl-4-octyl-1H-1,2,3-triazole **3l**. 46
Figure S 47. HR-ESI-MS spectrum of 1-cyclohexyl-4-octyl-1H-1,2,3-triazole **3l**. 46

Figure S 48. ^{1}H NMR spectrum of (1-butyl-1H-1,2,3-triazol-4-yl)methyl benzoate **3m**........................47
Figure S 49. ^{13}C NMR spectrum of (1-butyl-1H-1,2,3-triazol-4-yl)methyl benzoate **3m**.......................47
Figure S 50. ^{1}H NMR spectrum of 1-(2-methoxyphenyl)-4-phenyl-1H-1,2,3-triazole **3n**......................48
Figure S 51. ^{13}C NMR spectrum of 1-(2-methoxyphenyl)-4-phenyl-1H-1,2,3-triazole **3n**.....................48
Figure S 52. ^{1}H NMR spectrum of 1-(3-methoxyphenyl)-4-phenyl-1H-1,2,3-triazole **3o**......................49
Figure S 53. ^{13}C NMR spectrum of 1-(3-methoxyphenyl)-4-phenyl-1H-1,2,3-triazole **3o**.....................49
Figure S 54. ^{1}H NMR spectrum of 1-(4-methoxyphenyl)-4-phenyl-1H-1,2,3-triazole **3p**......................50
Figure S 55. ^{13}C NMR spectrum of 1-(4-methoxyphenyl)-4-phenyl-1H-1,2,3-triazole **3p**.....................50
Figure S 56. ^{1}H NMR spectrum of 1-phenyl-4-(4-methoxyphenyl)-1H-1,2,3-triazole **3q**......................51
Figure S 57. ^{13}C NMR spectrum of 1-phenyl-4-(4-methoxyphenyl)-1H-1,2,3-triazole **3q**.....................51
Figure S 58. ^{1}H NMR spectrum of N,N-Dimethyl-4-(4-phenyl-1H-1,2,3-triazol-1-yl)aniline **3r.**............52
Figure S 59. ^{13}C NMR spectrum of N,N-Dimethyl-4-(4-phenyl-1H-1,2,3-triazol-1-yl)aniline **3r.**...........52
Figure S 60. ^{1}H NMR spectrum of 2-((4-Phenyl-1H-1,2,3-triazol-1-yl)methyl)benzonitrile **3s.**.............53
Figure S 61. ^{13}C NMR spectrum of 2-((4-Phenyl-1H-1,2,3-triazol-1-yl)methyl)benzonitrile **3s.**............53
Figure S 62. ^{1}H NMR spectrum of 1-(4-Chlorobenzyl)-4-(p-tolyl)-1H-1,2,3-triazole **3t.** 54
Figure S 63. ^{13}C NMR spectrum of 1-(4-Chlorobenzyl)-4-(p-tolyl)-1H-1,2,3-triazole **3t.**.......................54
Figure S 64. ^{1}H NMR spectrum of 4-Phenyl-1-(4-(trifluoromethyl)benzyl)-1H-1,2,3-triazole **3u.** 55
Figure S 65. ^{13}C NMR spectrum of 4-Phenyl-1-(4-(trifluoromethyl)benzyl)-1H-1,2,3-triazole **3u.** 55
Figure S 66. ^{19}F NMR spectrum of 4-Phenyl-1-(4-(trifluoromethyl)benzyl)-1H-1,2,3-triazole **3u.**........56
Figure S 67. $^{19}F\{^{1}H\}$ NMR spectrum of 4-Phenyl-1-(4-(trifluoromethyl)benzyl)-1H-1,2,3-triazole **3u.** .56
Figure S 68. ^{1}H NMR spectrum of Ethyl 1-(4-(trifluoromethyl)benzyl)-1H-1,2,3-triazole-4-carboxylate **3v.**...56
Figure S 69. ^{13}C NMR spectrum of Ethyl 1-(4-(trifluoromethyl)benzyl)-1H-1,2,3-triazole-4-carboxylate **3v.**...57
Figure S 70. ^{19}F NMR spectrum of Ethyl 1-(4-(trifluoromethyl)benzyl)-1H-1,2,3-triazole-4-carboxylate **3v.**...57
Figure S 71. $^{19}F\{^{1}H\}$ NMR spectrum of Ethyl 1-(4-(trifluoromethyl)benzyl)-1H-1,2,3-triazole-4-carboxylate **3v.** 58
Figure S 72. ^{1}H NMR spectrum of 1-Phenyl-4-(4-(trifluoromethyl)phenyl)-1H-1,2,3-triazole **3w.**.......58
Figure S 73. ^{19}F NMR spectrum of 1-Phenyl-4-(4-(trifluoromethyl)phenyl)-1H-1,2,3-triazole **3w.** 58
Figure S 74. $^{19}F\{^{1}H\}$ NMR spectrum of 1-Phenyl-4-(4-(trifluoromethyl)phenyl)-1H-1,2,3-triazole **3w.** 59
Figure S 75. ^{1}H NMR spectrum of 1-(4-nitrophenyl)-4-phenyl-1H-1,2,3-triazole **3x**. 59
Figure S 76. ^{13}C NMR spectrum of 1-(4-nitrophenyl)-4-phenyl-1H-1,2,3-triazole **3x**. 60
Figure S 77. ^{1}H NMR spectrum of 4-(4-nitrophenyl)-1-phenyl-1H-1,2,3-triazole **3y**. 60
Figure S 78. ^{13}C NMR spectrum of 4-(4-nitrophenyl)-1-phenyl-1H-1,2,3-triazole **3y**. 61
Figure S 79. ^{1}H NMR spectrum of 1-(2,6-difluorobenzyl)-1H-1,2,3-triazole-4-carboxylic acid ethyl ester **3z**...61
Figure S 80. ^{13}C NMR spectrum of 1-(2,6-difluorobenzyl)-1H-1,2,3-triazole-4-carboxylic acid ethyl ester **3z**...62
Figure S 81. DEPT-135 NMR spectrum of 1-(2,6-difluorobenzyl)-1H-1,2,3-triazole-4-carboxylic acid ethyl ester **3z**...62
Figure S 82. ^{19}F NMR spectrum of 1-(2,6-difluorobenzyl)-1H-1,2,3-triazole-4-carboxylic acid ethyl ester **3z**...63
Figure S 83. $^{19}F\{^{1}H\}$ NMR spectrum of 1-(2,6-difluorobenzyl)-1H-1,2,3-triazole-4-carboxylic acid ethyl ester **3z**...63
Figure S 84. HSQC (^{1}H-DEPT-135) NMR spectrum of 1-(2,6-difluorobenzyl)-1H-1,2,3-triazole-4-carboxylic acid ethyl ester **3z**. 63

Figure S 85. ^{1}H NMR spectrum of 1-(2,6-difluorobenzyl)-1H-1,2,3-triazole-4-carboxylic acid ethyl ester **3z**. 64
Figure S 86. ^{13}C NMR spectrum of 1-(2,6-difluorobenzyl)-1H-1,2,3-triazole-4-carboxylic acid ethyl ester **3z**. 64
Figure S 87. DEPT-135 NMR spectrum of 1-(2,6-difluorobenzyl)-1H-1,2,3-triazole-4-carboxylic acid ethyl ester **3z**. 65
Figure S 88. ^{19}F NMR spectrum of 1-(2,6-difluorobenzyl)-1H-1,2,3-triazole-4-carboxylic acid ethyl ester **3z**. 65
Figure S 89. ^{19}F{^{1}H} NMR spectrum of 1-(2,6-difluorobenzyl)-1H-1,2,3-triazole-4-carboxylic acid ethyl ester **3z**. 65
Figure S 90. HSQC (^{1}H-DEPT-135) NMR spectrum of 1-(2,6-difluorobenzyl)-1H-1,2,3-triazole-4-carboxylic acid ethyl ester **3z**. 66
Figure S 91. FT-ATR-IR spectrum of 1-(2,6-difluorobenzyl)-1H-1,2,3-triazole-4-carboxylic acid ethyl ester **3z**. 66
Figure S 92. UV-Vis spectrum of 4.83E-4 M 1-(2,6-difluorobenzyl)-1H-1,2,3-triazole-4-carboxylic acid ethyl ester **3z** in DCM. 67
Figure S 93. UV-Vis spectra of 1-(2,6-difluorobenzyl)-1H-1,2,3-triazole-4-carboxylic acid ethyl ester **3z** in DCM. Concentrations of measured samples are described in the legend and are in mol·dm^{-3}. 67
Figure S 94. Absorption vs concentration graph of 1-(2,6-difluorobenzyl)-1H-1,2,3-triazole-4-carboxylic acid ethyl ester **3z** for determination of molar extinction coefficient using Beer-Lambert law. 68
Figure S 95. LR-ESI-MS spectrogram of 1-(2,6-difluorobenzyl)-1H-1,2,3-triazole-4-carboxylic acid ethyl ester **3z**. 68
Figure S 96. HR-ESI-Ms spectrogram of 1-(2,6-difluorobenzyl)-1H-1,2,3-triazole-4-carboxylic acid ethyl ester **3z**. 69
Figure S 97. ^{1}H NMR spectrum of 3′-Deoxy-3′-(4-phenyl-1H-1,2,3-triazol-1-yl)thymidine, 1-((2R,4S,5S)-5-(hydroxymethyl)-4-(4-phenyl-1H-1,2,3-triazol-1-yl)tetrahydrofuran-2-yl)-5-methylpyrimidine-2,4(1H,3H)-dione **3aa.** 69
Figure S 98. ^{13}C NMR spectrum of 3′-Deoxy-3′-(4-phenyl-1H-1,2,3-triazol-1-yl)thymidine, 1-((2R,4S,5S)-5-(hydroxymethyl)-4-(4-phenyl-1H-1,2,3-triazol-1-yl)tetrahydrofuran-2-yl)-5-methylpyrimidine-2,4(1H,3H)-dione **3aa.** 70
Figure S 99. DEPT-135 NMR spectrum of 3′-Deoxy-3′-(4-phenyl-1H-1,2,3-triazol-1-yl)thymidine, 1-((2R,4S,5S)-5-(hydroxymethyl)-4-(4-phenyl-1H-1,2,3-triazol-1-yl)tetrahydrofuran-2-yl)-5-methylpyrimidine-2,4(1H,3H)-dione **3aa.** 70
Figure S 100. HSQC (^{1}H-^{13}C) NMR spectrum of 3′-Deoxy-3′-(4-phenyl-1H-1,2,3-triazol-1-yl)thymidine, 1-((2R,4S,5S)-5-(hydroxymethyl)-4-(4-phenyl-1H-1,2,3-triazol-1-yl)tetrahydrofuran-2-yl)-5-methylpyrimidine-2,4(1H,3H)-dione **3aa.** 71
Figure S 101. HMBC (^{1}H-^{13}C) NMR spectrum of 3′-Deoxy-3′-(4-phenyl-1H-1,2,3-triazol-1-yl)thymidine, 1-((2R,4S,5S)-5-(hydroxymethyl)-4-(4-phenyl-1H-1,2,3-triazol-1-yl)tetrahydrofuran-2-yl)-5-methylpyrimidine-2,4(1H,3H)-dione **3aa.** 72
Figure S 102. LR-ESI-MS of 3′-Deoxy-3′-(4-phenyl-1H-1,2,3-triazol-1-yl)thymidine, 1-((2R,4S,5S)-5-(hydroxymethyl)-4-(4-phenyl-1H-1,2,3-triazol-1-yl)tetrahydrofuran-2-yl)-5-methylpyrimidine-2,4(1H,3H)-dione **3aa.** 72
Figure S 103. ^{1}H NMR spectrum of 1,3-bis(1-phenyl-1H-1,2,3-triazol-4-yl)propane **3ab**. 73
Figure S 104. DEPT-135 NMR spectrum of 1,3-bis(1-phenyl-1H-1,2,3-triazol-4-yl)propane **3ab**. 73
Figure S 105. ^{13}C NMR spectrum of 1,3-bis(1-phenyl-1H-1,2,3-triazol-4-yl)propane **3ab**. 74
Figure S 106. ^{1}H NMR spectrum of 1-phenyl--1H-1,2,3-triazolyl-4-carboxylic acid ethyl ester **3ac**. 74
Figure S 107. ^{13}C NMR spectrum of 1-phenyl--1H-1,2,3-triazolyl-4-carboxylic acid ethyl ester **3ac**. 75

Figure S 108. ^{1}H NMR spectrum of 1-(3β)-Cholest-5-en-3-yl-4-phenyl-1H-1,2,3-triazole, 1-((3S,8S,9S,10R,13R,14S,17R)-10,13-dimethyl-17-((R)-6-methylheptan-2-yl)-2,3,4,7,8,9,10,11,12,13,14,15,16,17-tetradecahydro-1H-cyclopenta[a]phenanthren-3-yl)-4-phenyl-1H-1,2,3-triazole **3ad.**......75
Figure S 109. ^{13}C NMR spectrum of 1-(3β)-Cholest-5-en-3-yl-4-phenyl-1H-1,2,3-triazole, 1-((3S,8S,9S,10R,13R,14S,17R)-10,13-dimethyl-17-((R)-6-methylheptan-2-yl)-2,3,4,7,8,9,10,11,12,13,14,15,16,17-tetradecahydro-1H-cyclopenta[a]phenanthren-3-yl)-4-phenyl-1H-1,2,3-triazole **3ad.**......76
Figure S 110. DEPT-135 NMR spectrum of 1-(3β)-Cholest-5-en-3-yl-4-phenyl-1H-1,2,3-triazole, 1-((3S,8S,9S,10R,13R,14S,17R)-10,13-dimethyl-17-((R)-6-methylheptan-2-yl)-2,3,4,7,8,9,10,11,12,13,14,15,16,17-tetradecahydro-1H-cyclopenta[a]phenanthren-3-yl)-4-phenyl-1H-1,2,3-triazole **3ad.**......76
Figure S 111. LR-ESI-MS spectrogram of 1-(3β)-Cholest-5-en-3-yl-4-phenyl-1H-1,2,3-triazole, 1-((3S,8S,9S,10R,13R,14S,17R)-10,13-dimethyl-17-((R)-6-methylheptan-2-yl)-2,3,4,7,8,9,10,11,12,13,14,15,16,17-tetradecahydro-1H-cyclopenta[a]phenanthren-3-yl)-4-phenyl-1H-1,2,3-triazole **3ad.**......77
Figure S 112. ^{1}H NMR spectrum of 1-phenyl-1H-1,2,3-triazole **3ae**. 77
Figure S 113. ^{13}C NMR spectrum of 1-phenyl-1H-1,2,3-triazole **3ae**. 77
Figure S 114. ^{1}H NMR spectrum of 1-(2,6-difluorobenzyl)-1H-1,2,3-triazole **3af**. 78
Figure S 115. ^{13}C NMR spectrum of 1-(2,6-difluorobenzyl)-1H-1,2,3-triazole **3af**. 78
Figure S 116. ^{19}F{^{1}H} NMR spectrum of 1-(2,6-difluorophenyl)-1H-1,2,3-triazole **3af**. 79
Figure S 117. ^{1}H NMR spectrum of 4-methyl-1-phenyl-1H-1,2,3-triazole **3af**. 79
Figure S 118. NMR spectrum of 4-methyl-1-phenyl-1H-1,2,3-triazole **3ag**. 79
Figure S 119. ^{1}H NMR spectrum of 1-benzyl-4-methyl-1H-1,2,3-triazole **3ah**. 80
Figure S 120. ^{13}C NMR spectrum of 1-benzyl-4-methyl-1H-1,2,3-triazole **3ah**. 80
Figure S 121. ^{1}H NMR spectrum of 1-phenyl-4-propyl-1H-1,2,3-triazole **3ai**. 81
Figure S 122.^{13}C NMR spectrum of 1-phenyl-4-propyl-1H-1,2,3-triazole **3ai**. 81
Figure S 123. ^{1}H NMR spectrum of I 1-phenyl-4-(trimethylsilyl)-1H-1,2,3-triazole **3aj**. 82
Figure S 124. ^{13}C NMR spectrum of 1-phenyl-4-(trimethylsilyl)-1H-1,2,3-triazole **3aj**. 82
Figure S 125. ^{1}H NMR spectrum of 1-(2,6-difluorobenzyl)-1H-1,2,3-triazole-4-carboxamide (rufinamide) **4**......83
Figure S 126. ^{13}C NMR spectrum of 1-(2,6-difluorobenzyl)-1H-1,2,3-triazole-4-carboxamide (rufinamide) **4**......83
Figure S 127.^{19}F NMR spectrum of 1-(2,6-difluorobenzyl)-1H-1,2,3-triazole-4-carboxamide (rufinamide) **4**......84
Figure S 128. ^{19}F{^{1}H} NMR spectrum of 1-(2,6-difluorobenzyl)-1H-1,2,3-triazole-4-carboxamide (rufinamide) **4**......84
Figure S 129. ^{1}H NMR spectrum of 1-(2,6-difluorobenzyl)-1H-1,2,3-triazole-4-carboxylic acid. 84
Figure S 130. ^{13}C NMR spectrum of 1-(2,6-difluorobenzyl)-1H-1,2,3-triazole-4-carboxylic acid. 85
Figure S 131. DEPT-135 NMR spectrum of 1-(2,6-difluorobenzyl)-1H-1,2,3-triazole-4-carboxylic acid. 85
Figure S 132. ^{19}F NMR spectrum of 1-(2,6-difluorobenzyl)-1H-1,2,3-triazole-4-carboxylic acid. 86
Figure S 133. ^{19}F{^{1}H} NMR spectrum of 1-(2,6-difluorobenzyl)-1H-1,2,3-triazole-4-carboxylic acid. ...86

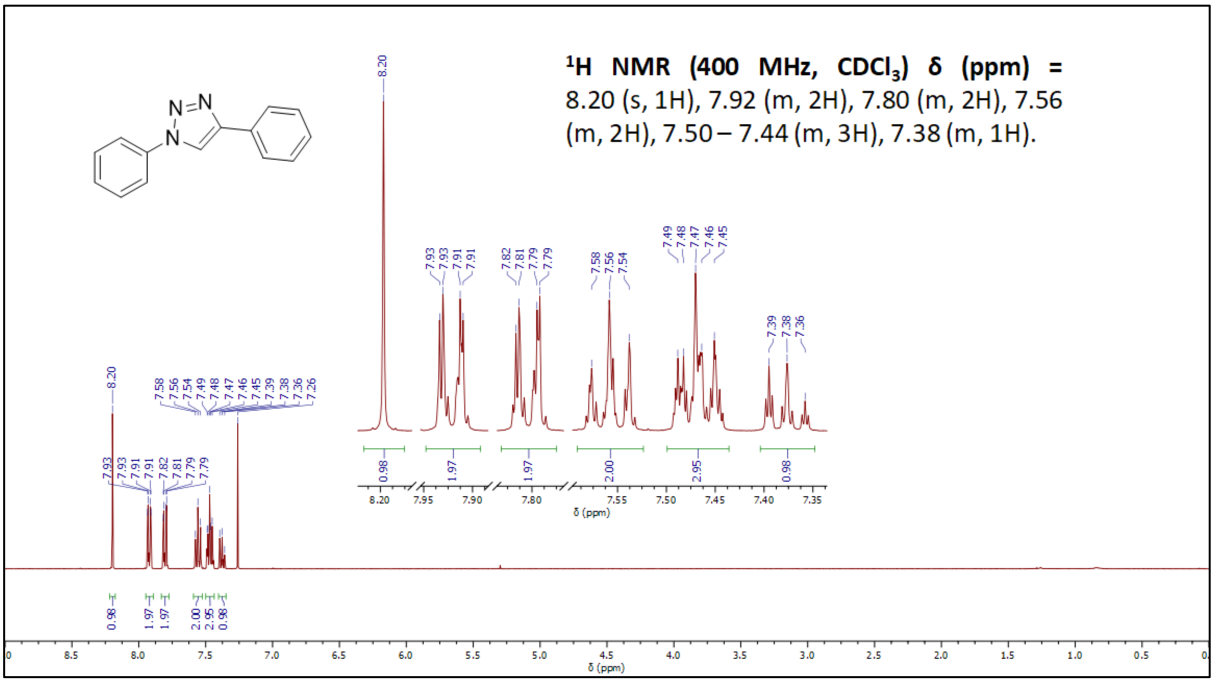

Figure S 12. ^{1}H NMR spectrum of 1,4-diphenyl-1H-1,2,3-triazole ***3a****.*

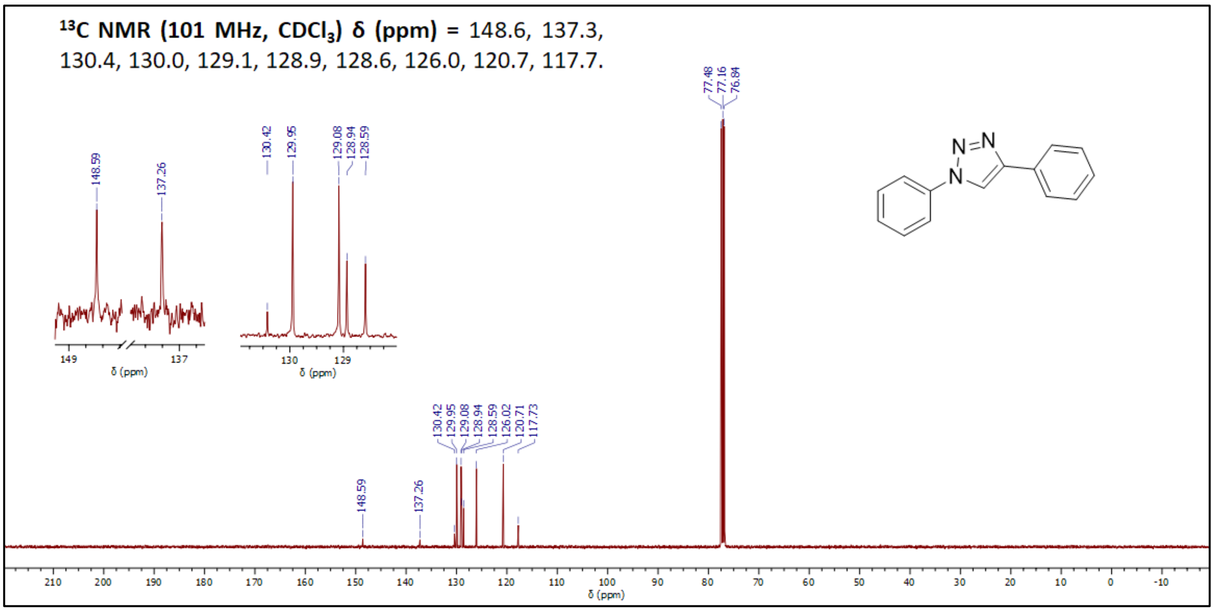

Figure S 13. ^{13}C NMR spectrum of 1,4-diphenyl-1H-1,2,3-triazole ***3a****.*

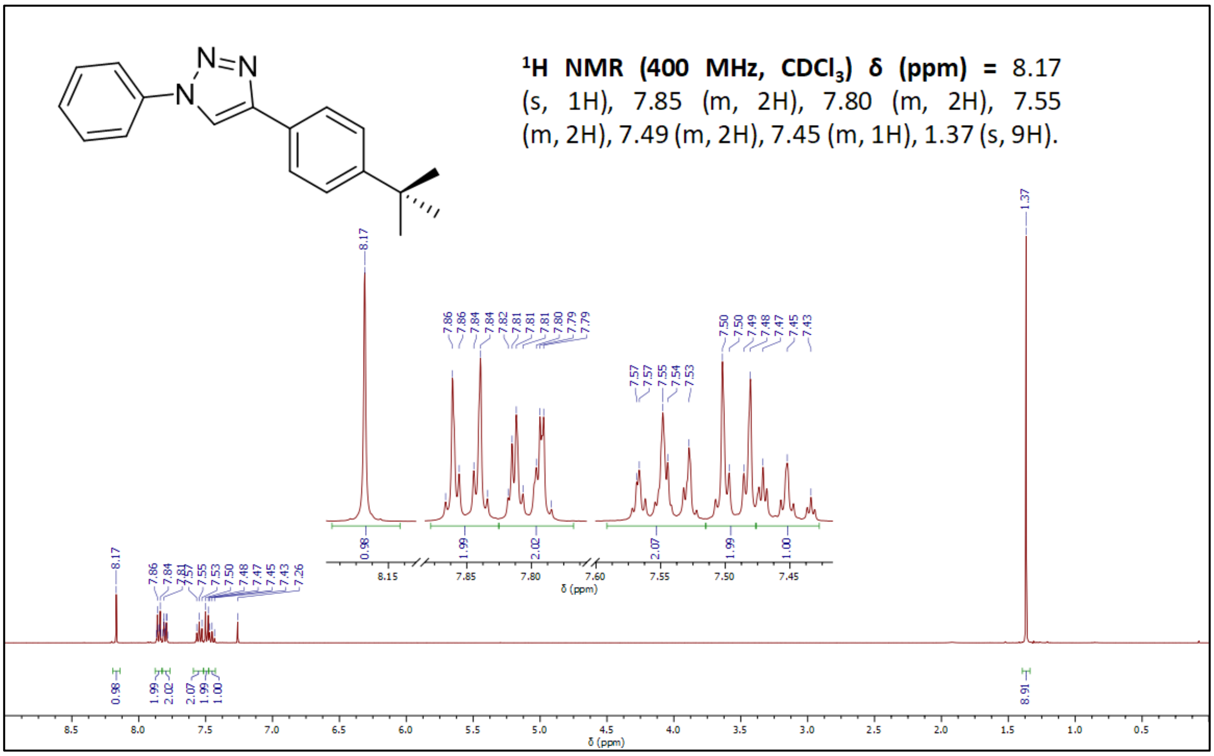

*Figure S 14. ¹H NMR spectrum of 1-phenyl-4-(4-tert-butyl)phenyl-1H-1,2,3-triazole **3b**.*

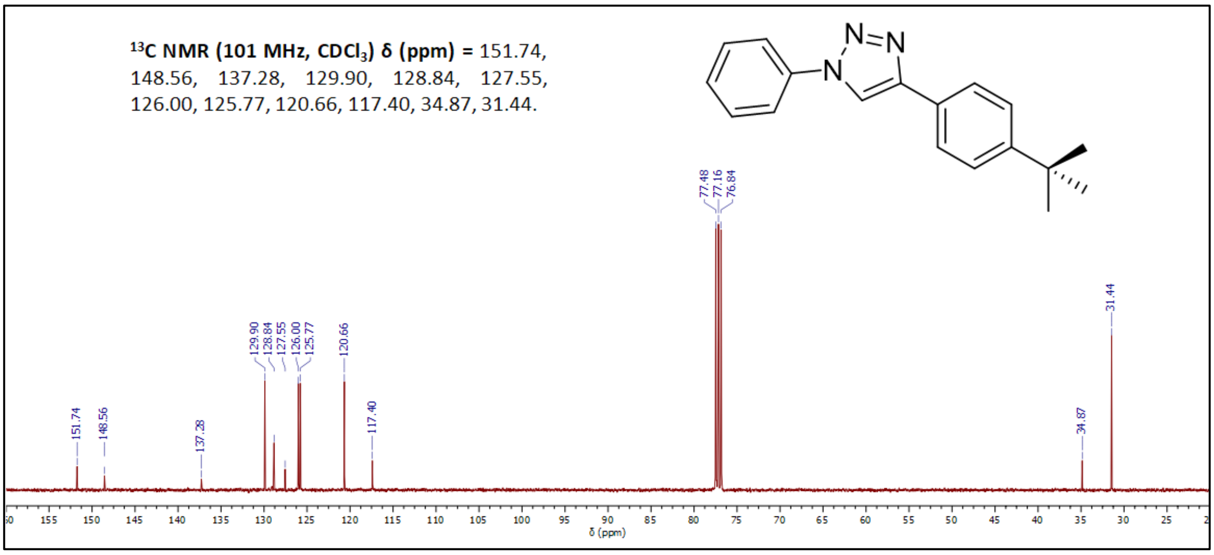

*Figure S 15. ¹³C NMR spectrum of 1-phenyl-4-(4-tert-butyl)phenyl-1H-1,2,3-triazole **3b**.*

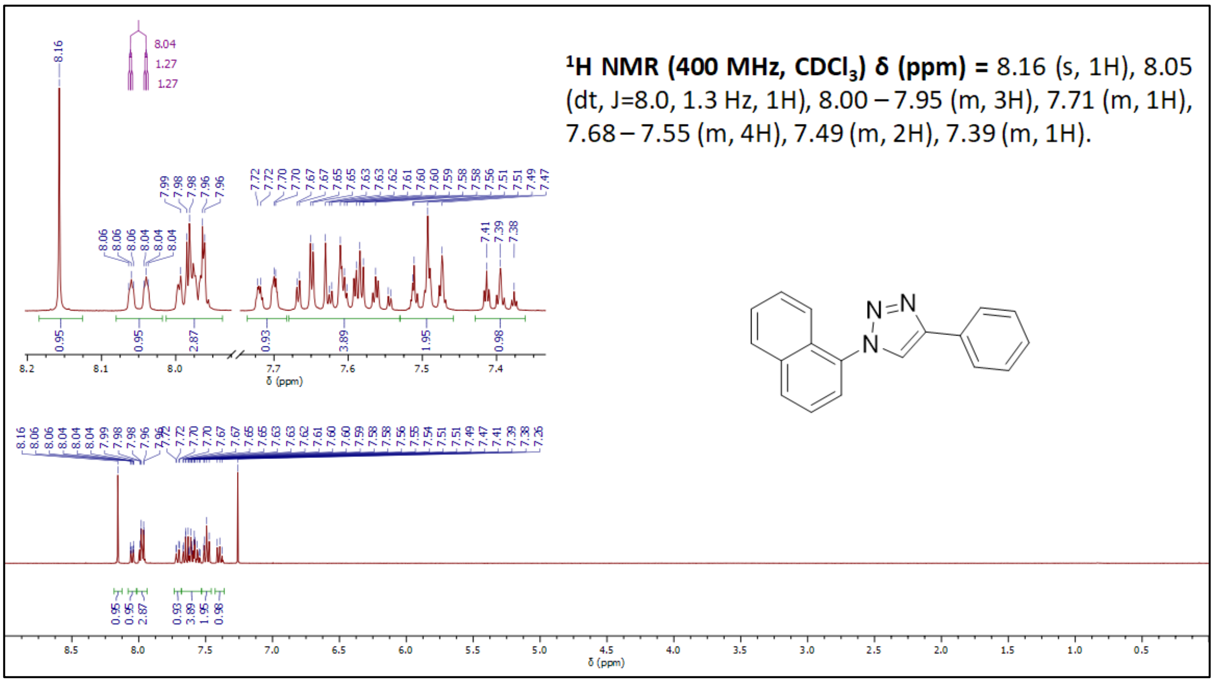

*Figure S 16. ^{1}H NMR spectrum of 1-naphthalenyl-4-phenyl-1H-1,2,3-triazole **3c**.*

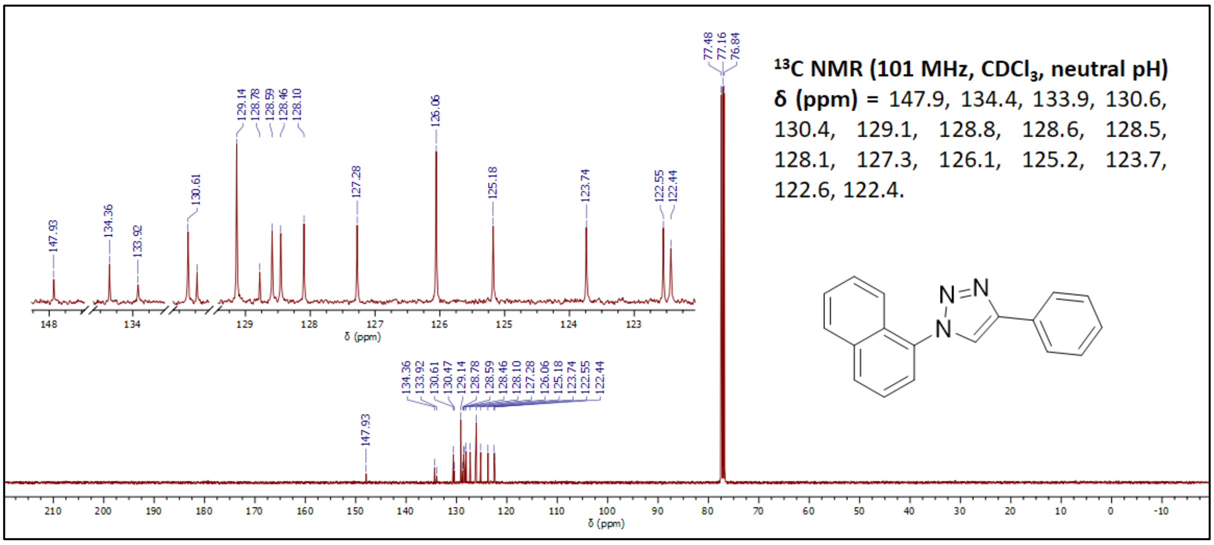

*Figure S 17. ^{13}C NMR spectrum of 1-naphthalenyl-4-phenyl-1H-1,2,3-triazole **3c**.*

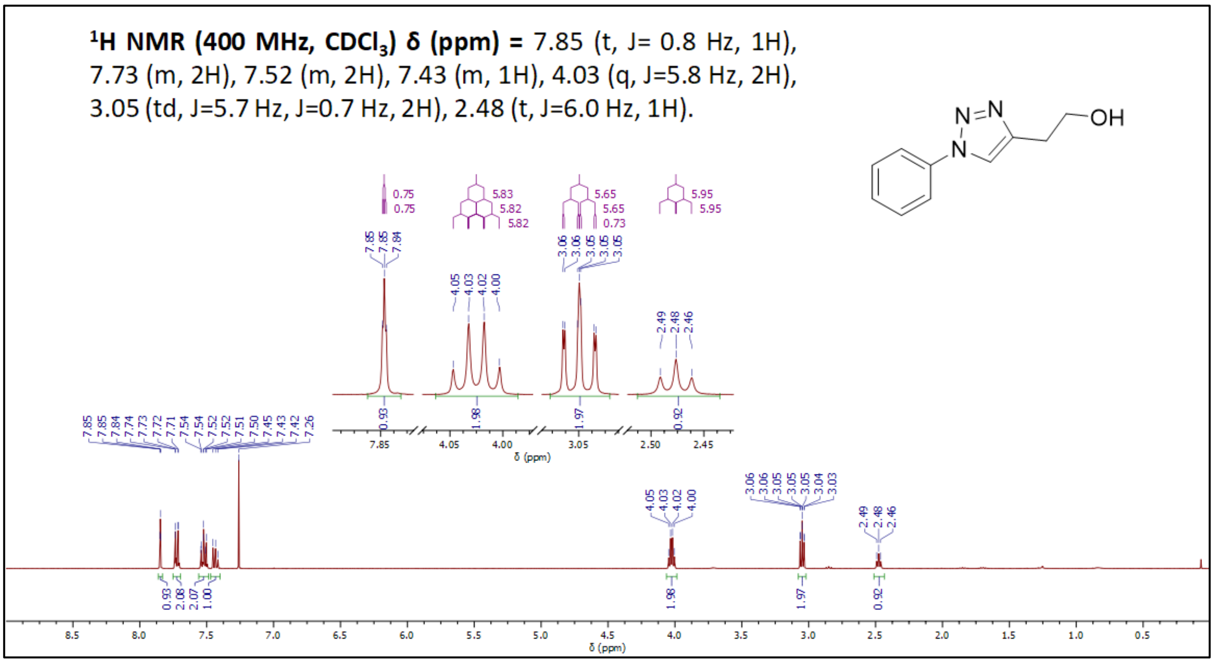

*Figure S 18. ¹H NMR spectrum of 1-(1-phenyl-1H-1,2,3-triazol-4-yl)ethan-1-ol **3d**.*

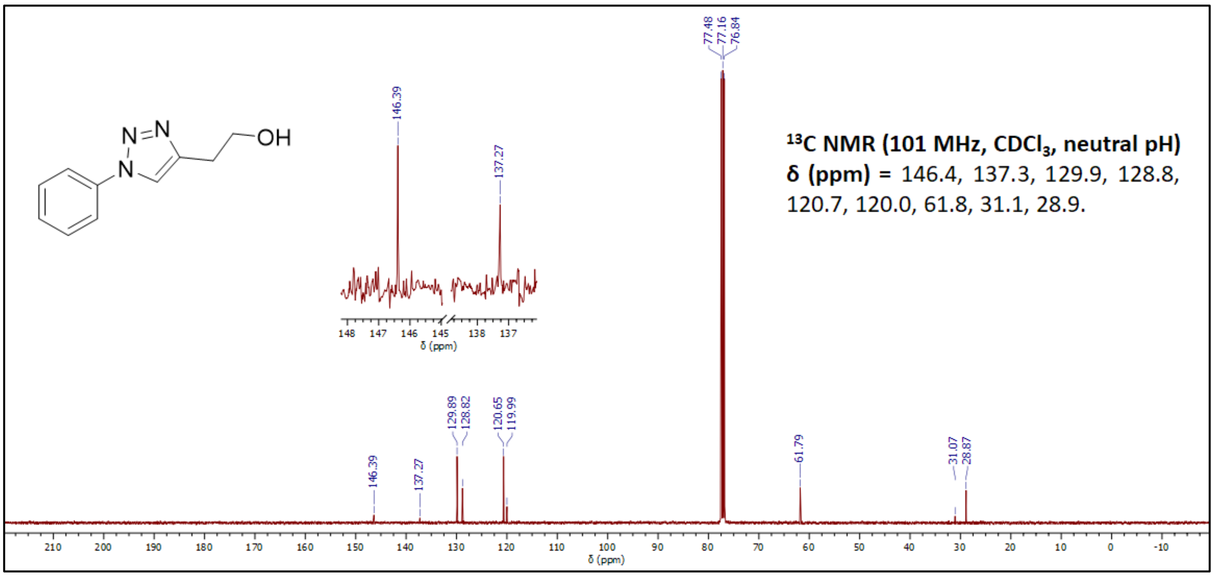

*Figure S 19. ¹³C NMR spectrum of 1-(1-phenyl-1H-1,2,3-triazol-4-yl)ethan-1-ol **3d**.*

^{1}H NMR (400 MHz, $CDCl_3$) δ (ppm) = 7.74 (br s, 1H), 7.71 (m, 2H), 7.51 (m, 2H), 7.42 (m, 1H), 3.71 (t, J=6.4 Hz, 2H), 2.85 (td, J=7.6, 0.7 Hz, 2H), 1.95 (m, 2H), 1.68 (m, 2H), 1.54 (br s, 1H).

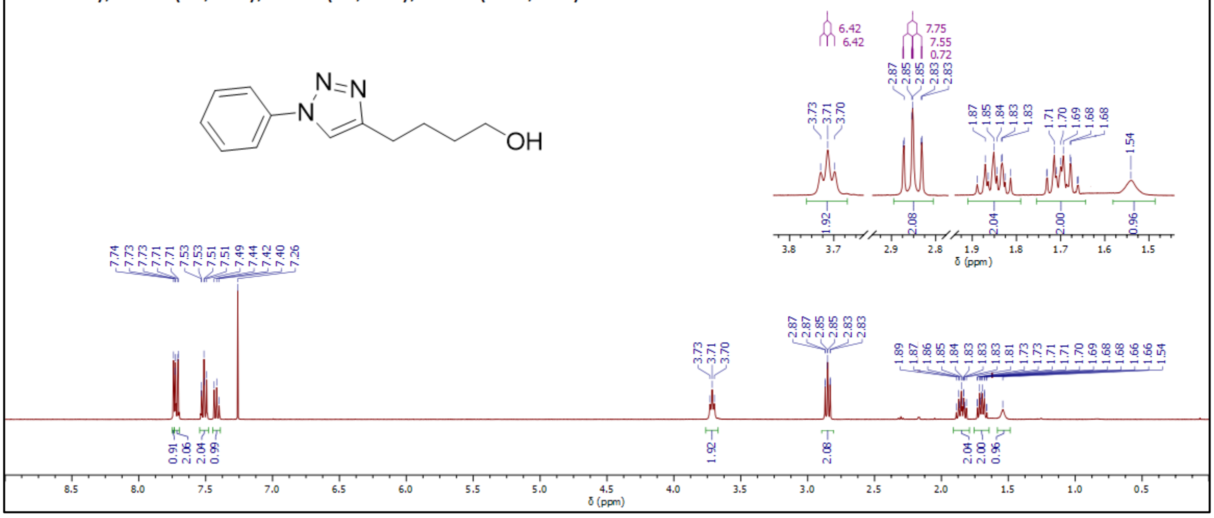

*Figure S 20. ^{1}H NMR spectrum of 4-(1-phenyl-1H-1,2,3-triazol-4-yl)butan-1-ol **3e**.*

^{13}C NMR (101 MHz, $CDCl_3$, neutral pH) δ (ppm) = 148.9, 137.4, 129.8, 128.6, 120.6, 119.1, 62.7, 32.3, 25.7, 25.5.

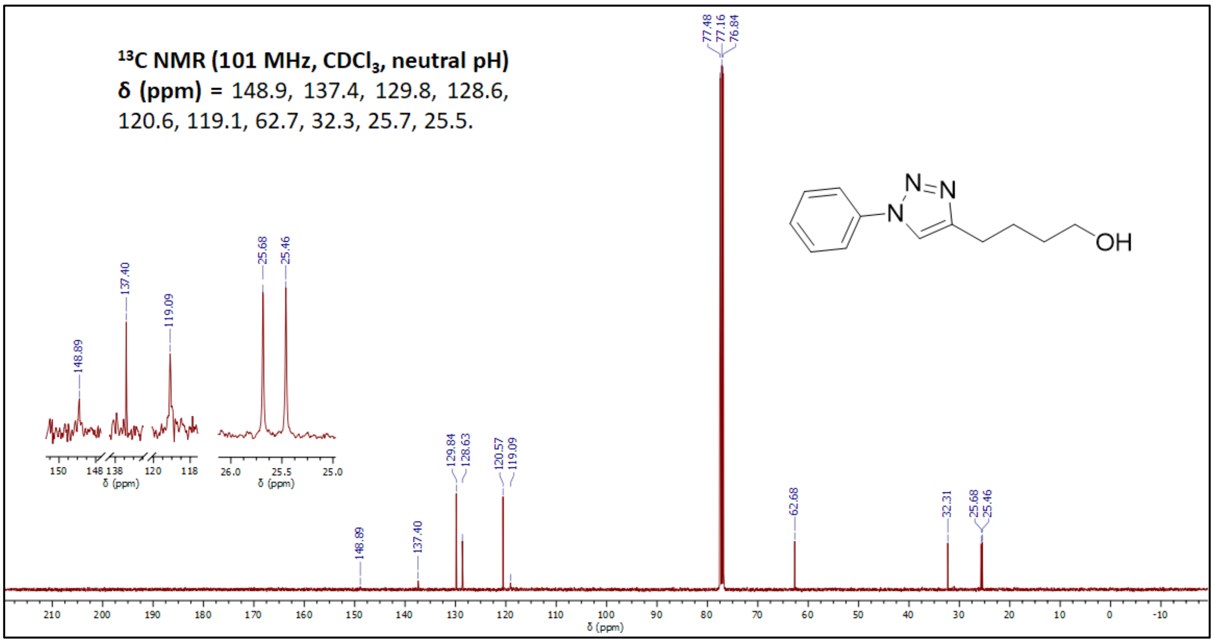

*Figure S 21. ^{13}C NMR spectrum of 4-(1-phenyl-1H-1,2,3-triazol-4-yl)butan-1-ol **3e**.*

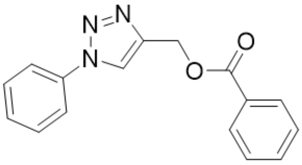

^{1}H NMR (400 MHz, CDCl$_3$) δ (ppm) = 8.14 (s, 1H), 8.07 (m, 2H), 7.74 (m, 2H), 7.59 – 7.49 (m, 3H), 5.56 (s, 2H).

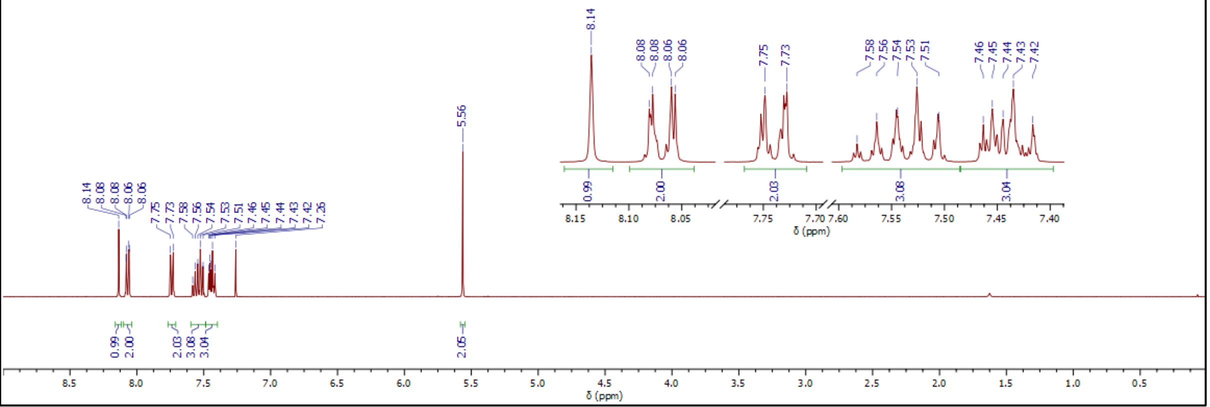

*Figure S 22. ^{1}H NMR spectrum of (1-phenyl-1H-1,2,3-triazol-4-yl)methyl benzoate **3f**.*

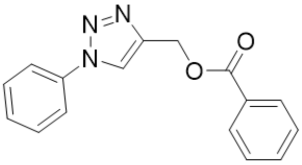

^{13}C NMR (101 MHz, CDCl$_3$) δ (ppm) = 166.7, 143.8, 137.1, 133.4, 129.9, 129.9, 129.8, 129.1, 128.6, 122.4, 120.8, 58.2.

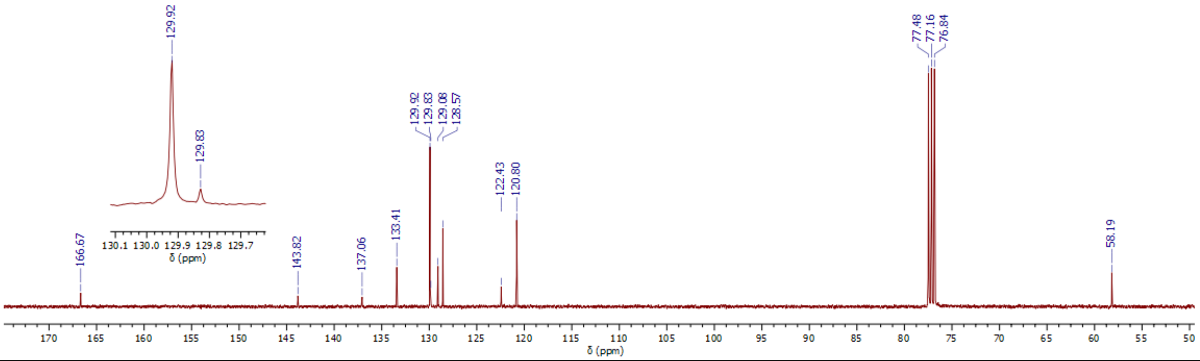

*Figure S 23. ^{13}C NMR spectrum of (1-phenyl-1H-1,2,3-triazol-4-yl)methyl benzoate **3f**.*

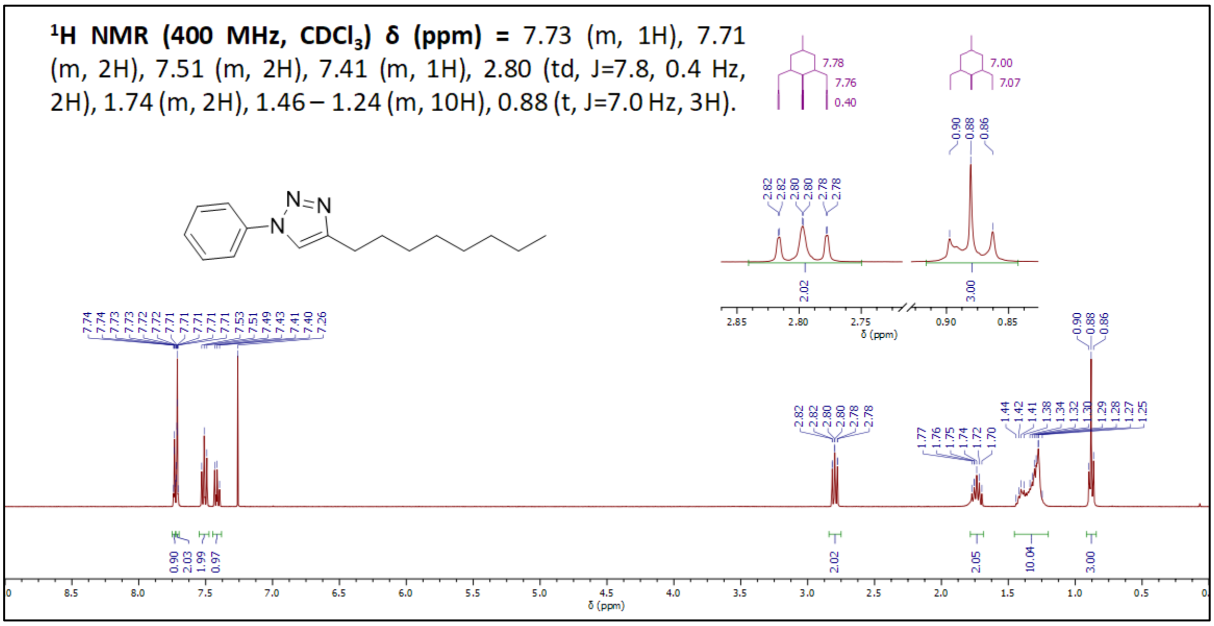

*Figure S 24. ¹H NMR spectrum of 4-octyl-1-phenyl-1H-1,2,3-triazole **3g**.*

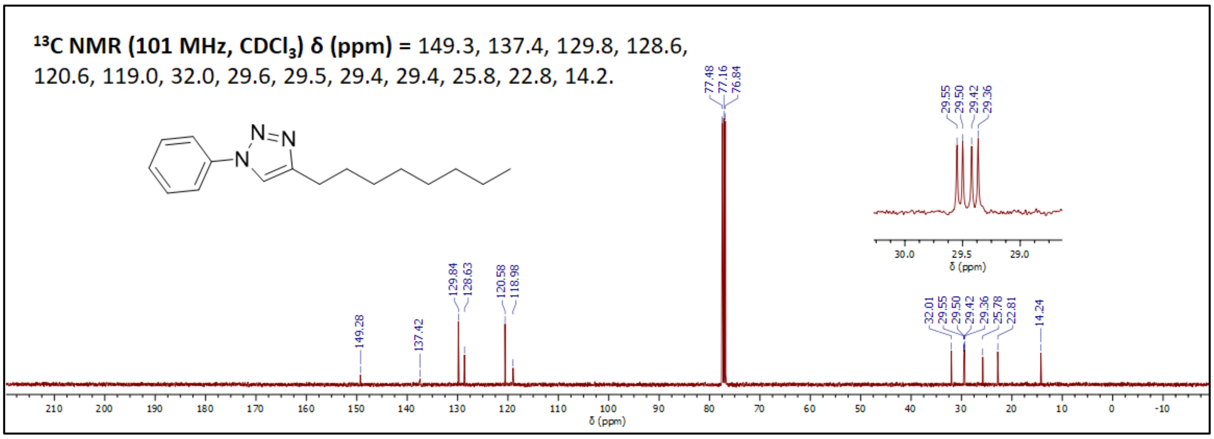

*Figure S 25. ¹³C NMR spectrum of 4-octyl-1-phenyl-1H-1,2,3-triazole **3g**.*

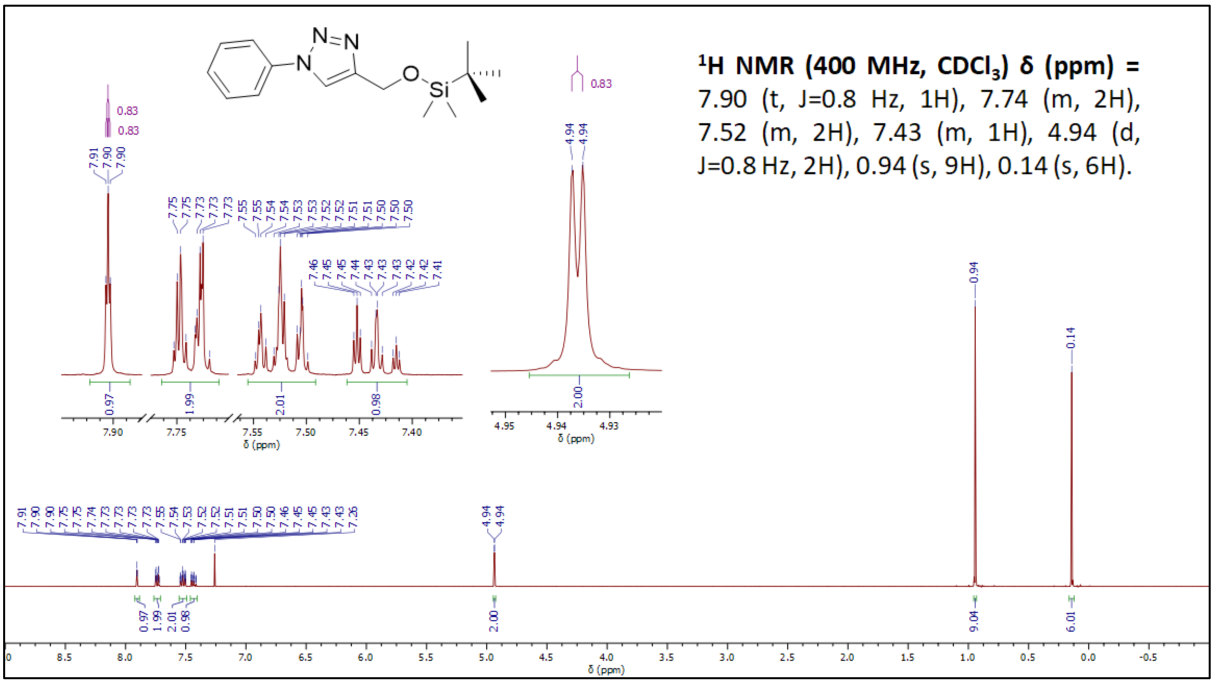

*Figure S 26. ¹H NMR spectrum of 4-(((tert-butyldimethylsilyl)oxy)methyl)-1-phenyl-1H-1,2,3-triazole **3h**.*

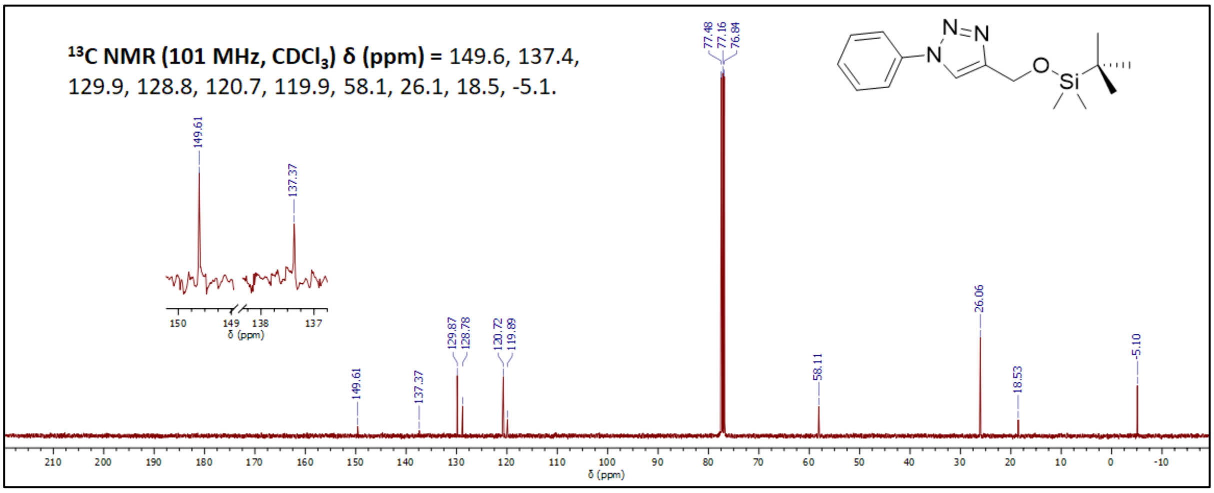

*Figure S 27. ¹³C NMR spectrum of 4-(((tert-butyldimethylsilyl)oxy)methyl)-1-phenyl-1H-1,2,3-triazole **3h**.*

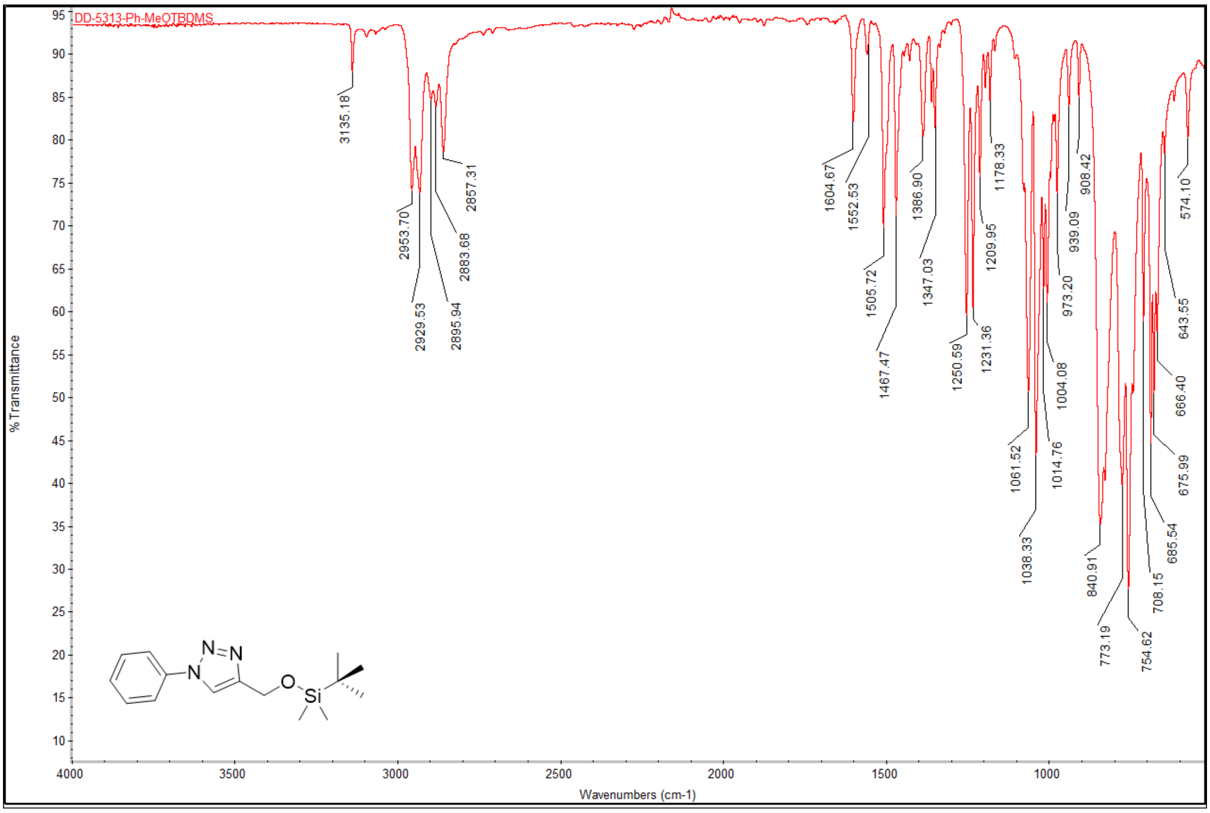

*Figure S 28.FT-ATR-IR spectrum of 4-(((tert-butyldimethylsilyl)oxy)methyl)-1-phenyl-1H-1,2,3-triazole **3h**.*

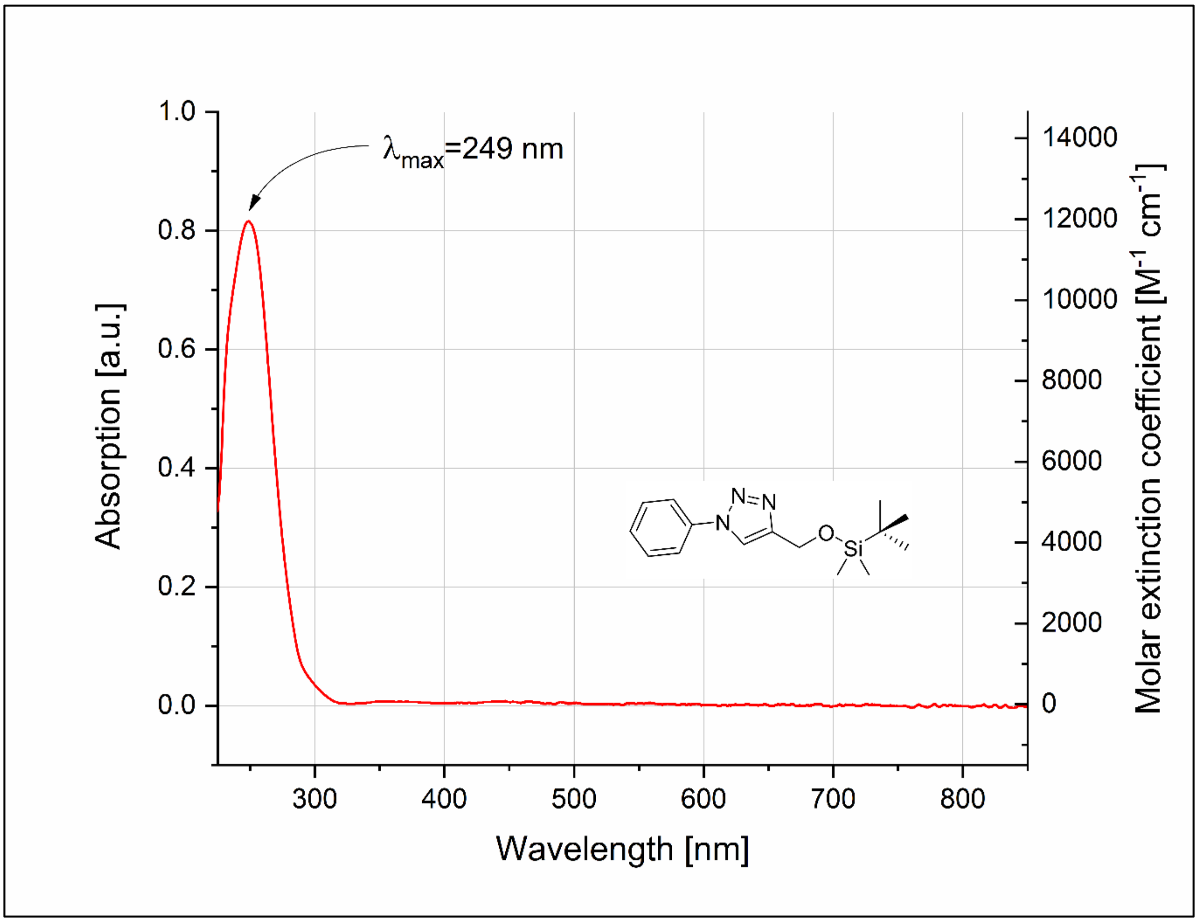

*Figure S 29. UV-Vis spectrum of 6.84E-5 M 4-(((tert-butyldimethylsilyl)oxy)methyl)-1-phenyl-1H-1,2,3-triazole **3h** in DCM.*

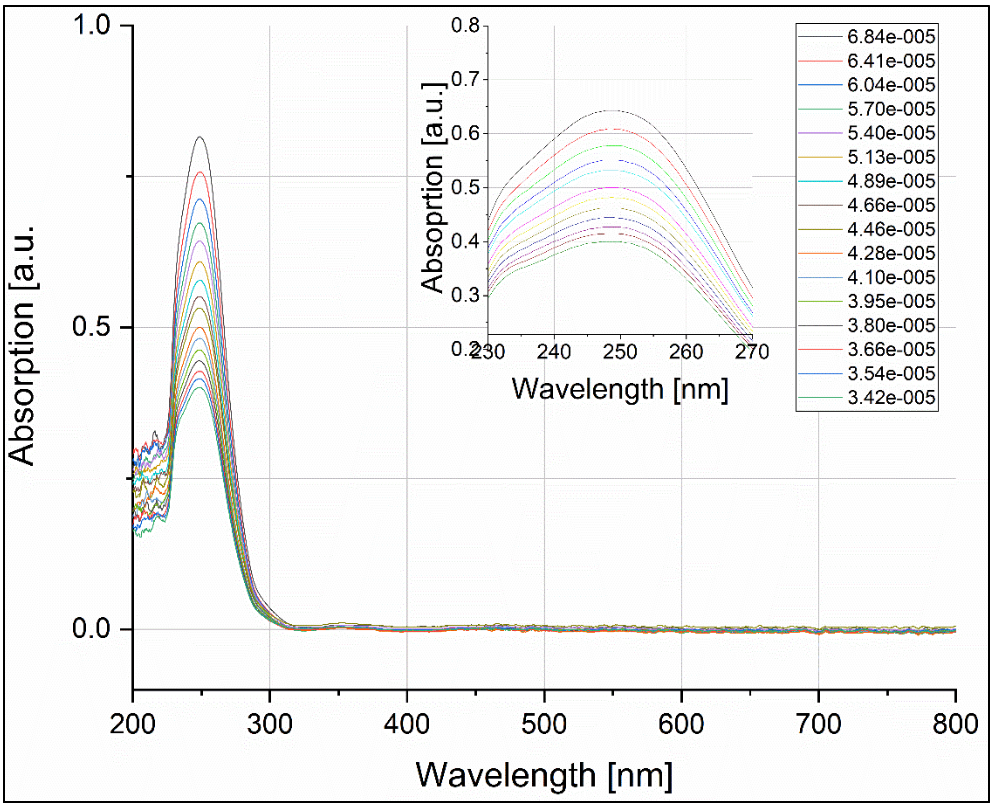

Figure S 30. UV-Vis spectra of 4-(((tert-butyldimethylsilyl)oxy)methyl)-1-phenyl-1H-1,2,3-triazole ***3h*** *in DCM. Concentrations of measured samples are described in the legend and are in mol·dm^{-3}. Note that the region between 200 and 230 nm is saturated due to solvent absorption.*

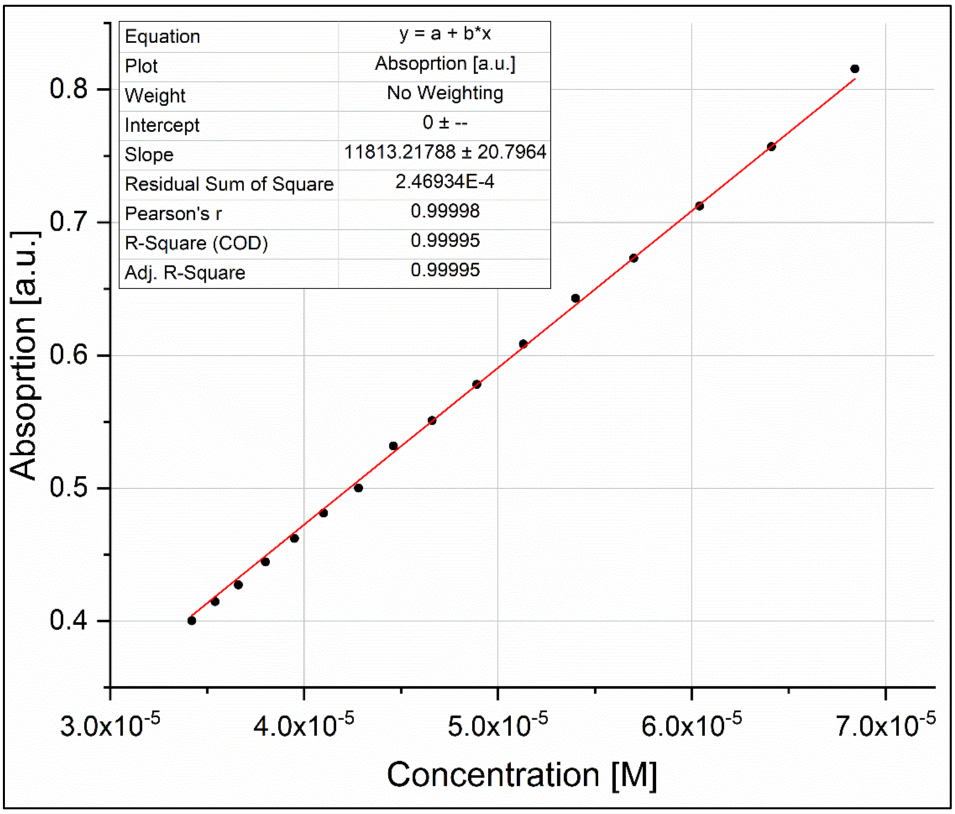

Figure S 31. Absorption vs concentration graph of 4-(((tert-butyldimethylsilyl)oxy)methyl)-1-phenyl-1H-1,2,3-triazole ***3h*** *for determination of molar extinction coefficient using Beer-Lambert law.*

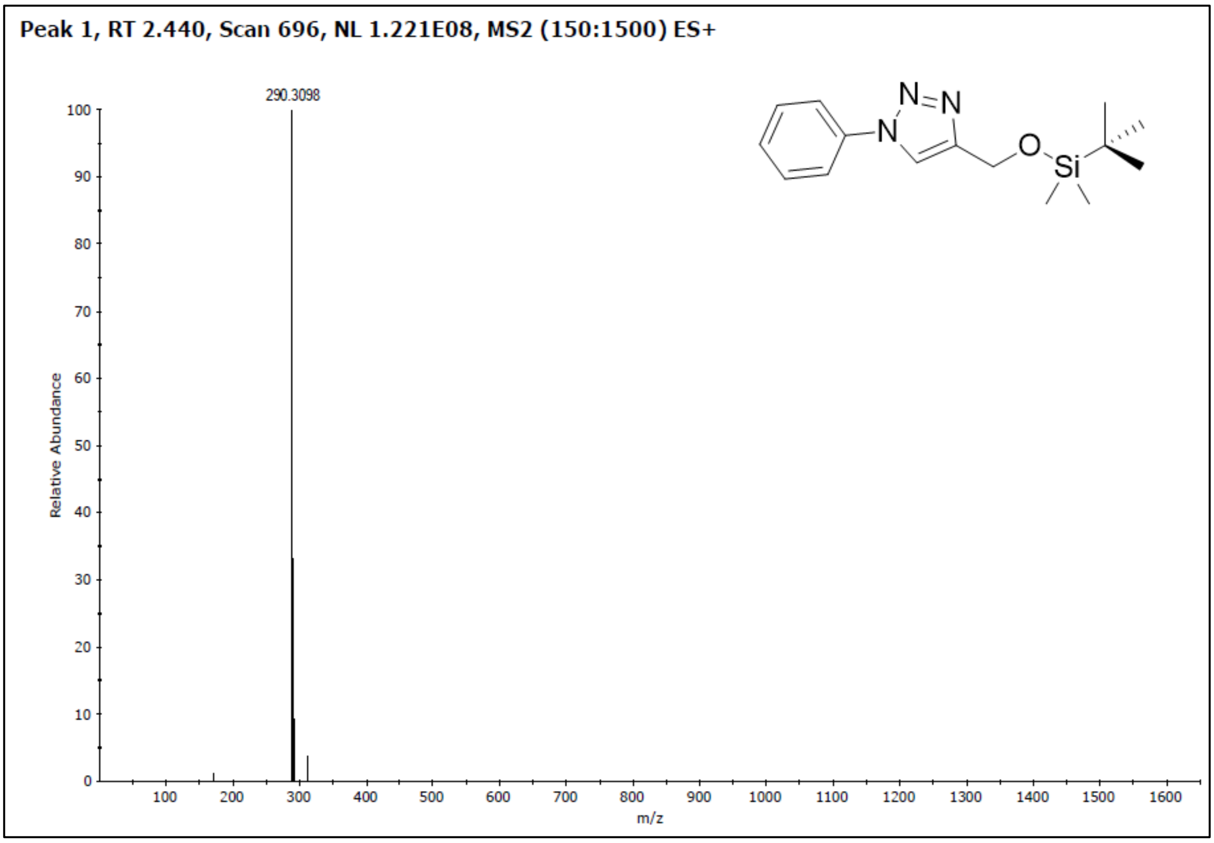

*Figure S 32. LR-ESI-MS spectrum of 4-(((tert-butyldimethylsilyl)oxy)methyl)-1-phenyl-1H-1,2,3-triazole **3h**.*

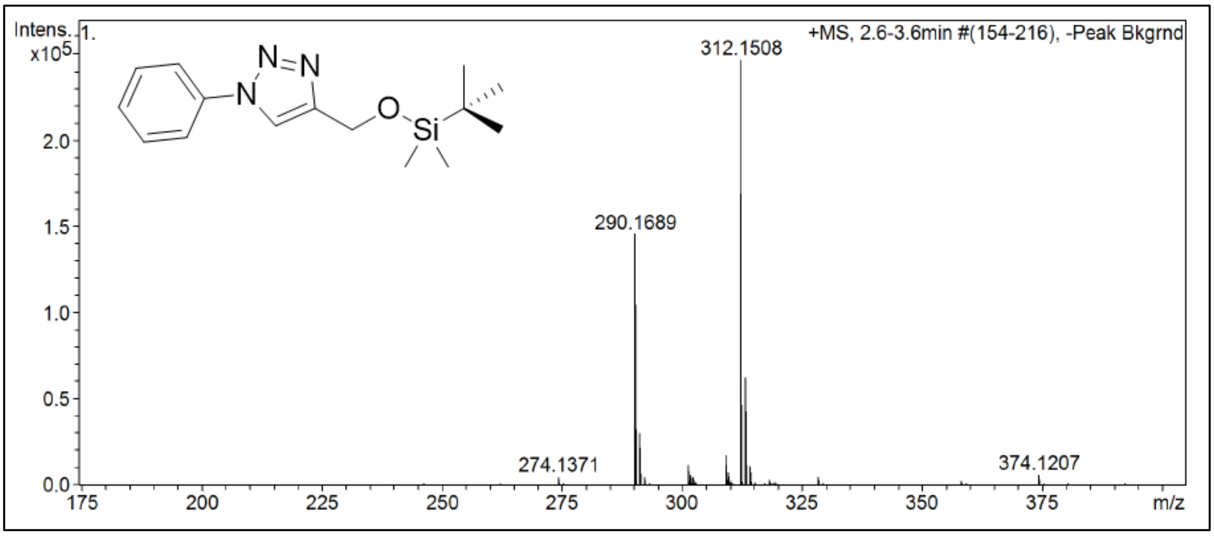

*Figure S 33. HR-ESI-MS spectrum of 4-(((tert-butyldimethylsilyl)oxy)methyl)-1-phenyl-1H-1,2,3-triazole **3h**.*

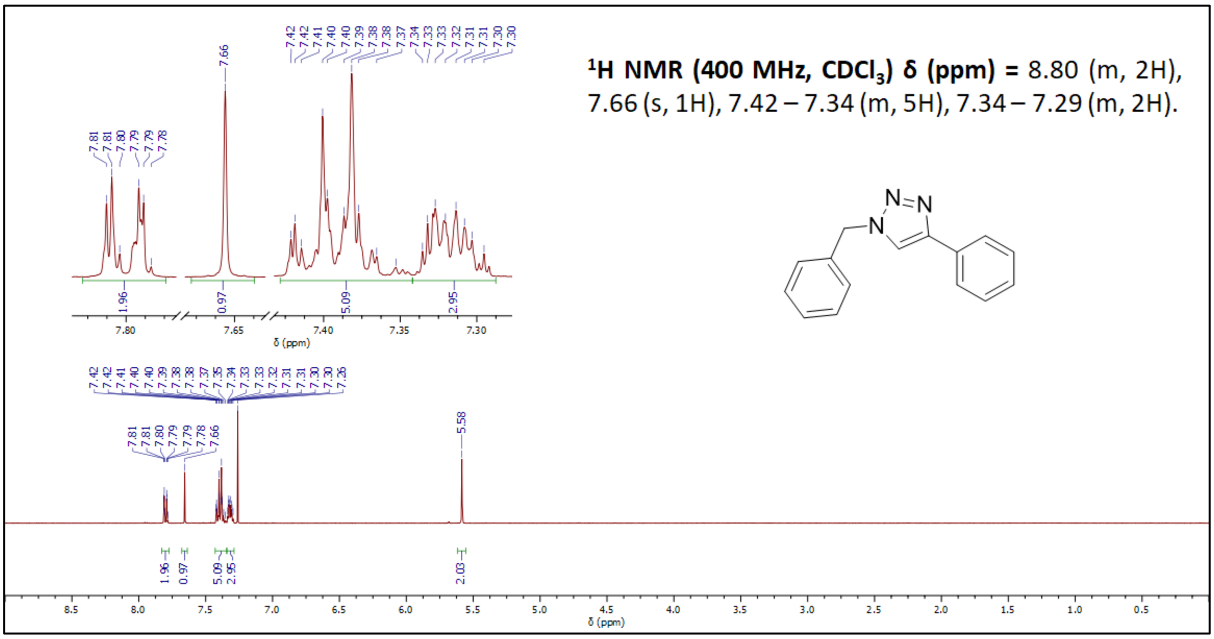

*Figure S 34. ^{1}H NMR spectrum of 1-benzyl-4-phenyl-1H-1,2,3-triazole **3i**.*

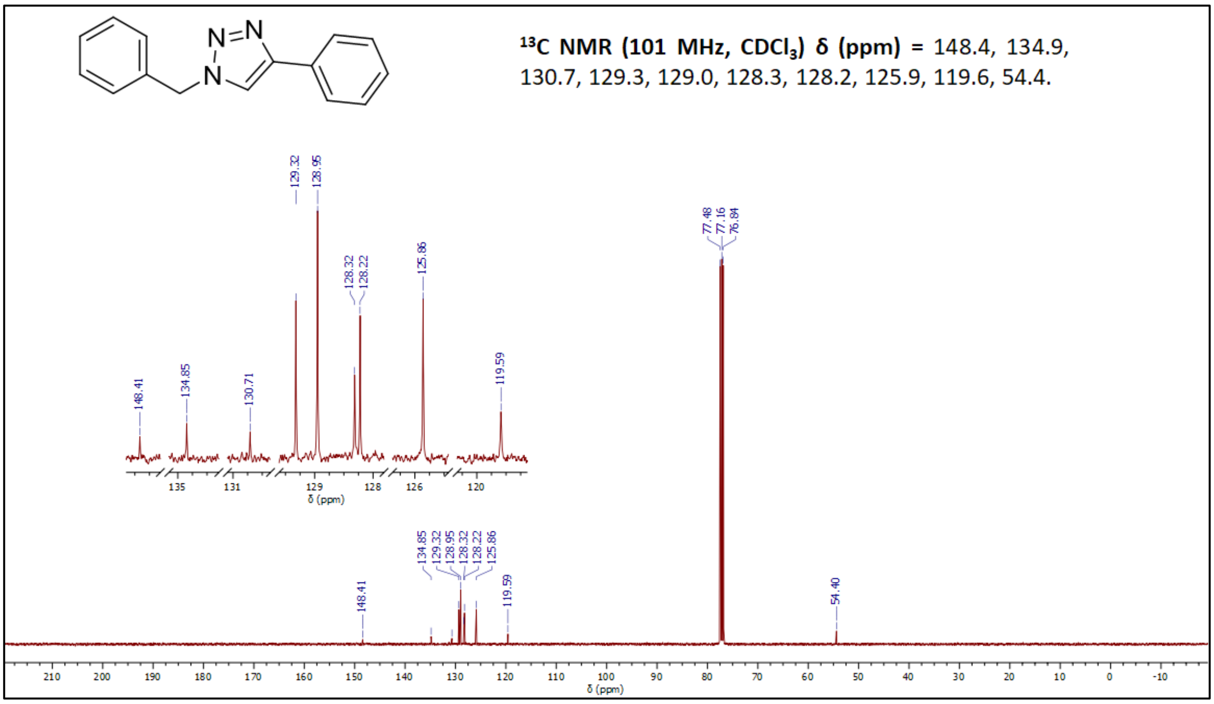

*Figure S 35. ^{13}C NMR spectrum of 1-benzyl-4-phenyl-1H-1,2,3-triazole **3i**.*

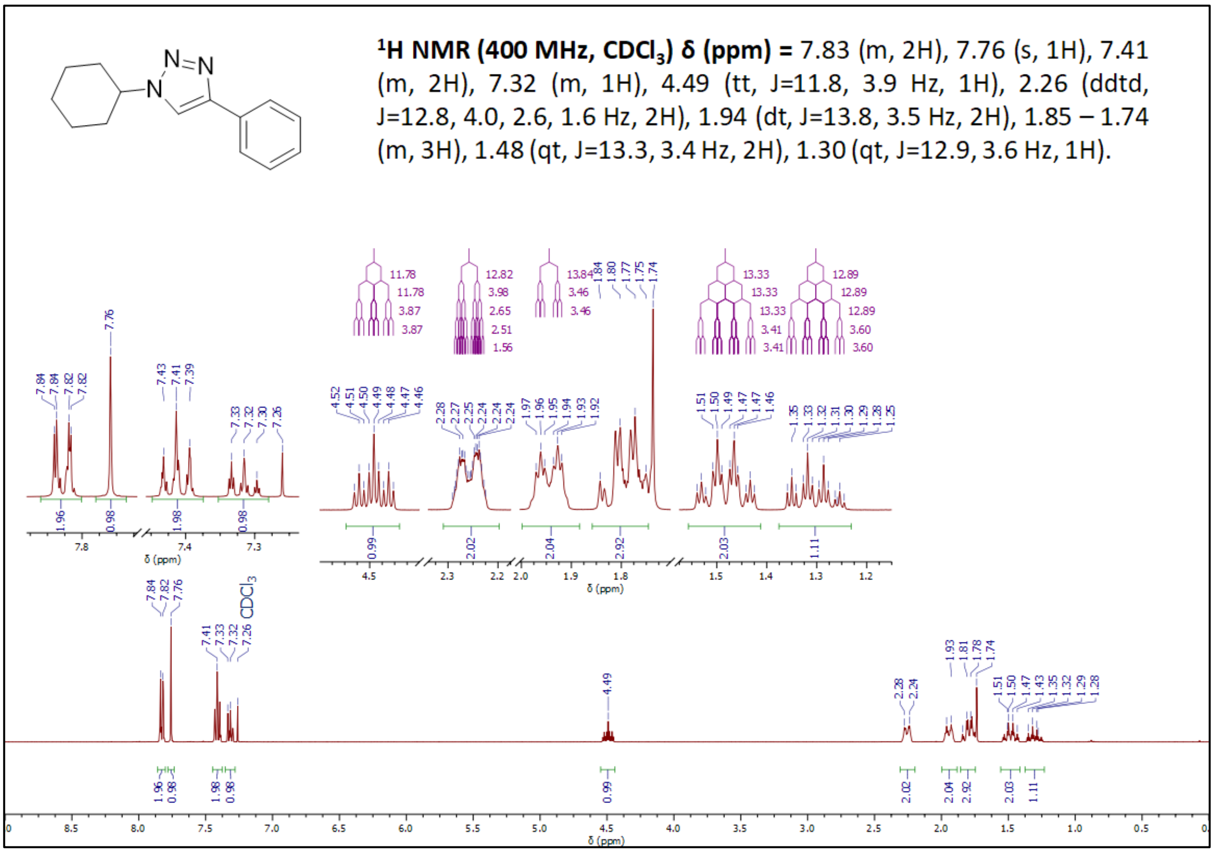

*Figure S 36. ¹H NMR spectrum of 1-cyclohexyl-4-phenyl-1H-1,2,3-triazole **3j**.*

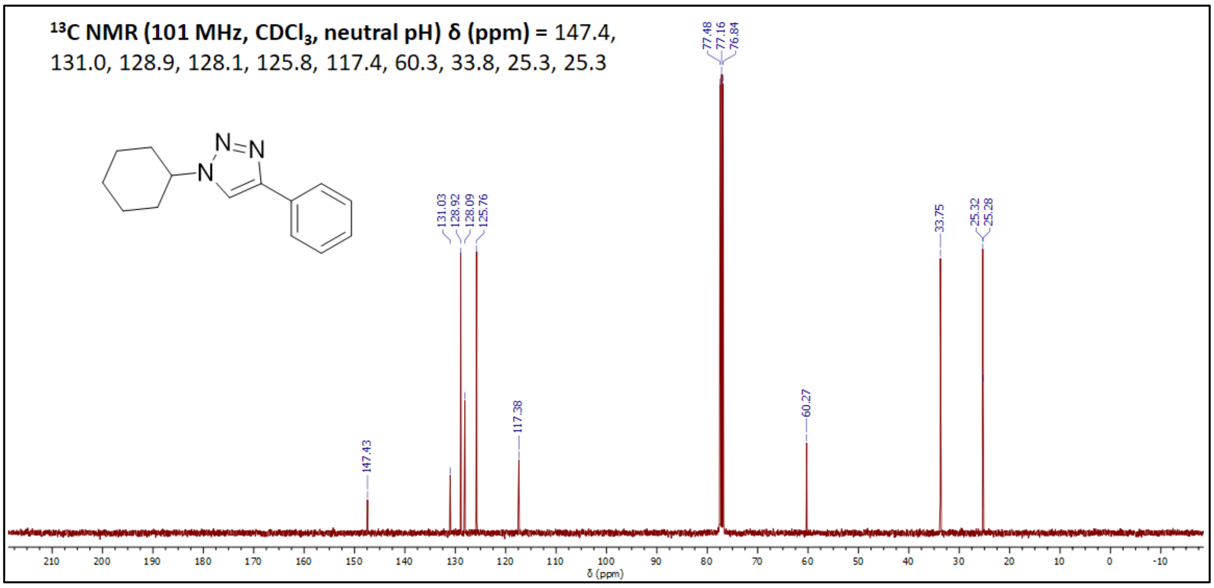

*Figure S 37. ¹³C NMR spectrum of 1-cyclohexyl-4-phenyl-1H-1,2,3-triazole **3j**.*

^{1}H NMR (400 MHz, $CDCl_3$) δ (ppm) = 7.83 (m, 2H), 7.74 (s, 1H), 7.42 (m, 2H), 7.33 (m, 1H), 4.40 (t, J=7.2 Hz, 2H), 1.94 (tt, J=7.6 Hz, J=7.5 Hz, 2H), 1.40 (m, 2H), 0.98 (t, J=7.4 Hz, 3H).

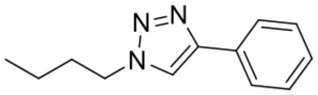

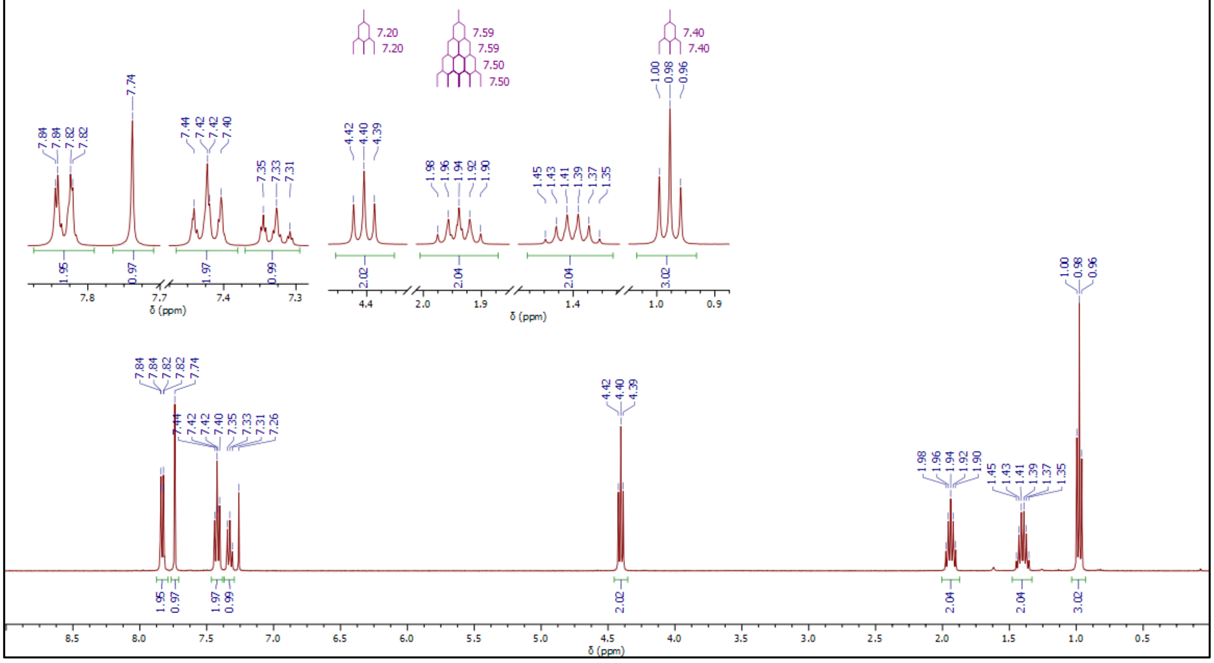

*Figure S 38. ^{1}H NMR spectrum of 1-butyl-4-phenyl-1H-1,2,3-triazole **3k**.*

^{13}C NMR (101 MHz, $CDCl_3$) δ (ppm) = 147.9, 130.9, 129.0, 128.2, 125.8, 119.5, 50.3, 32.5, 19.9, 13.6.

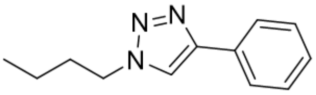

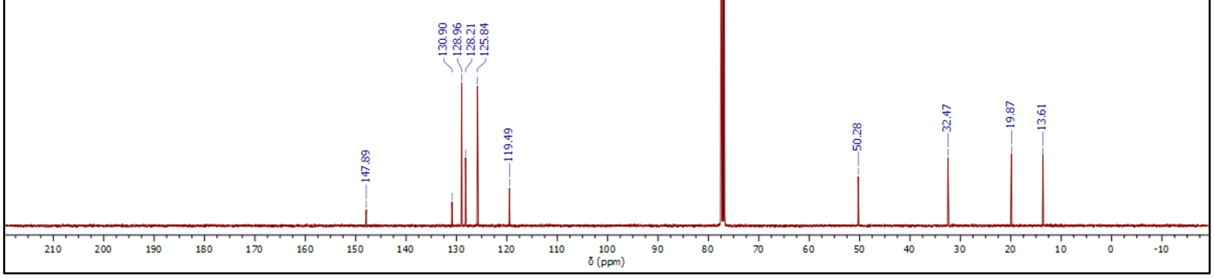

*Figure S 39. ^{13}C NMR spectrum of 1-butyl-4-phenyl-1H-1,2,3-triazole **3k**.*

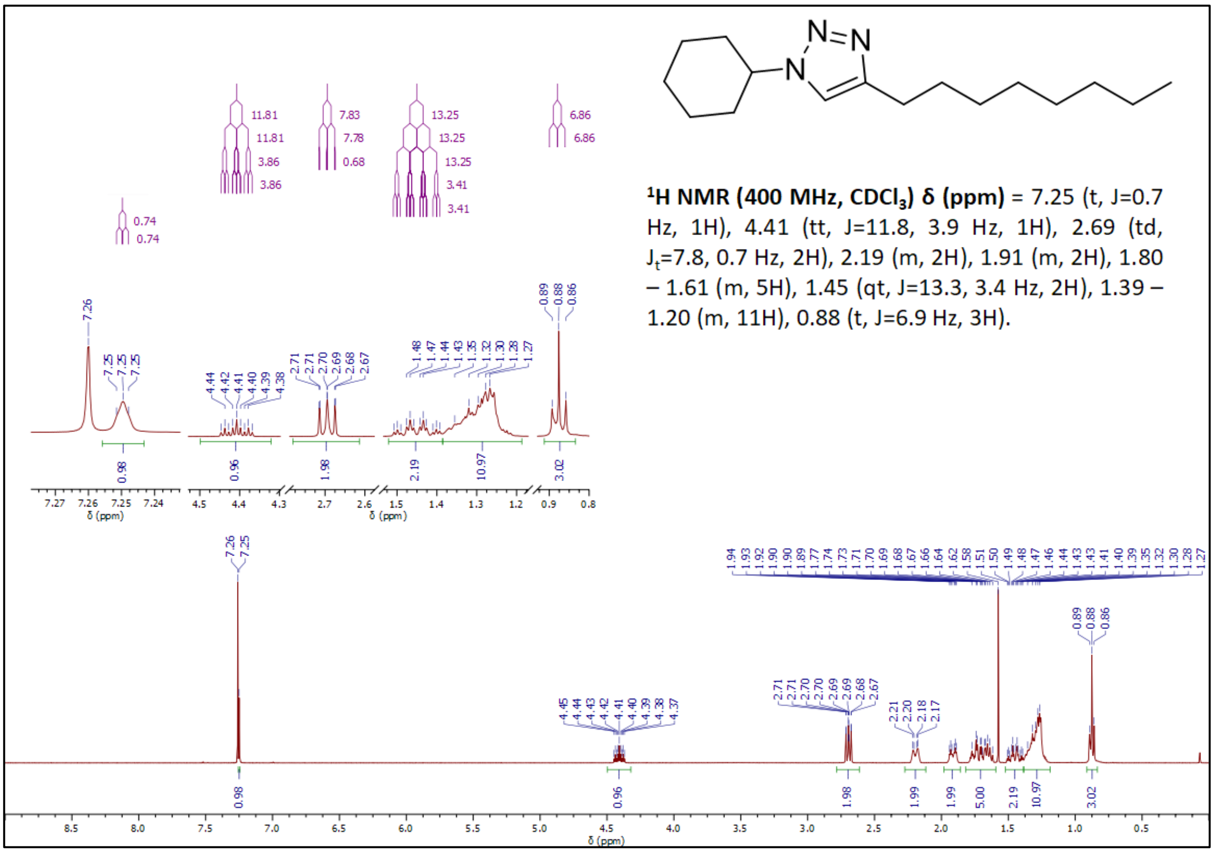

*Figure S 40. ¹H NMR spectrum of 1-cyclohexyl-4-octyl-1H-1,2,3-triazole **3l**.*

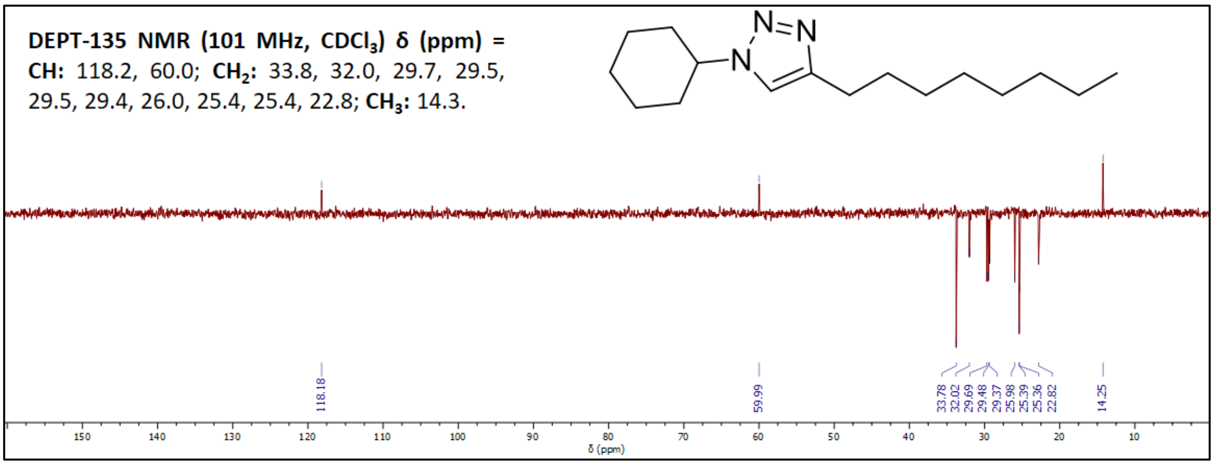

*Figure S 41. DEPT-135 NMR spectrum of 1-cyclohexyl-4-octyl-1H-1,2,3-triazole **3l**.*

^{13}C NMR (101 MHz, $CDCl_3$) δ (ppm) = 148.2, 118.2, 60.0, 33.8, 32.0, 29.7, 29.5, 29.5, 29.4, 26.0, 25.4, 25.4, 22.8, 14.3.

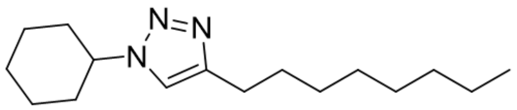

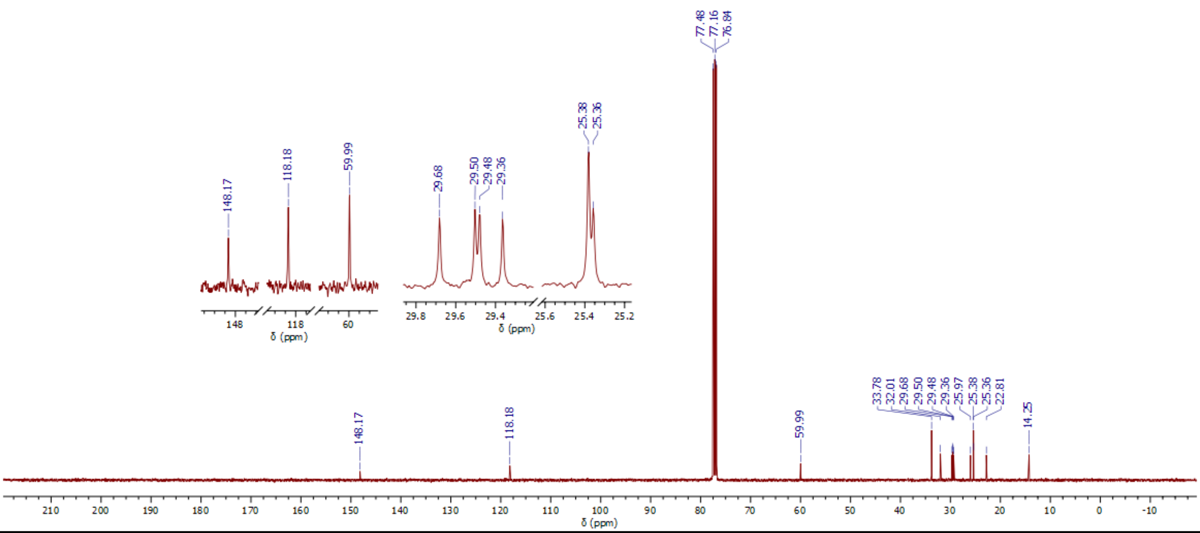

*Figure S 42. ^{13}C NMR spectrum of 1-cyclohexyl-4-octyl-1H-1,2,3-triazole **3l**.*

^{1}H–^{13}C NMR ((400, 101), $CDCl_3$) δ (ppm) = (7.25 118.03), (4.41 59.84), (2.69 25.87), (2.21 33.50), (1.93 25.30), (1.90 25.22), (1.78 25.23), (1.71 33.54), (1.66 29.44), (1.43 25.23), (1.32 29.21), (1.28 22.53), (1.26 31.92), (1.26 25.21), (0.88 14.14).

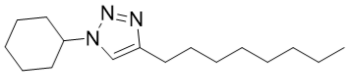

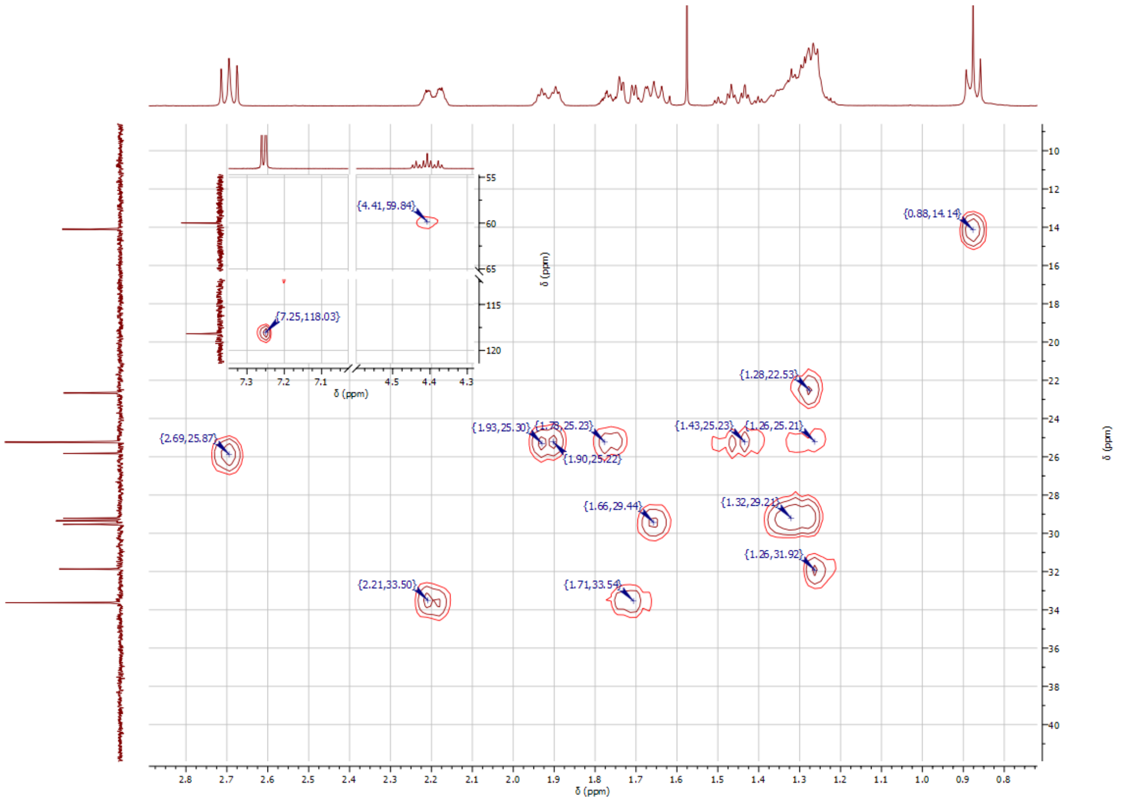

*Figure S 43. HSQC (^{1}H-^{13}C) NMR spectrum of 1-cyclohexyl-4-octyl-1H-1,2,3-triazole **3l**.*

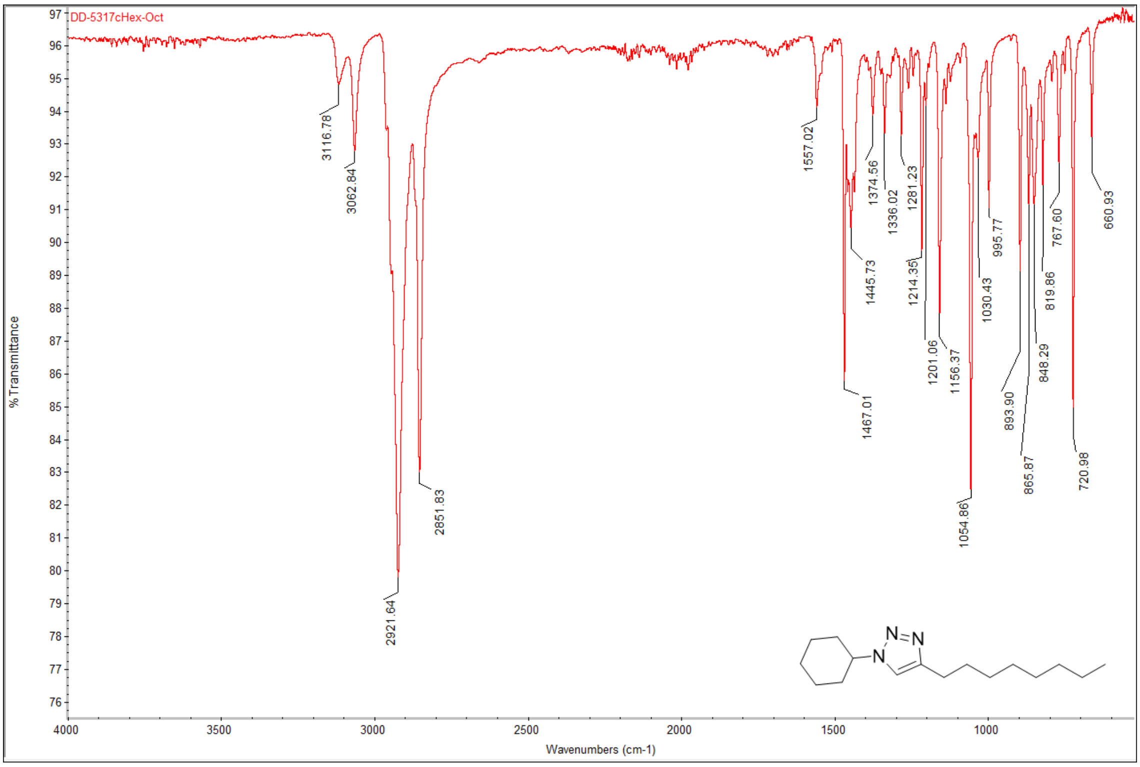

*Figure S 44. FT-ATR-IR spectrum of 1-cyclohexyl-4-octyl-1H-1,2,3-triazole **3l**.*

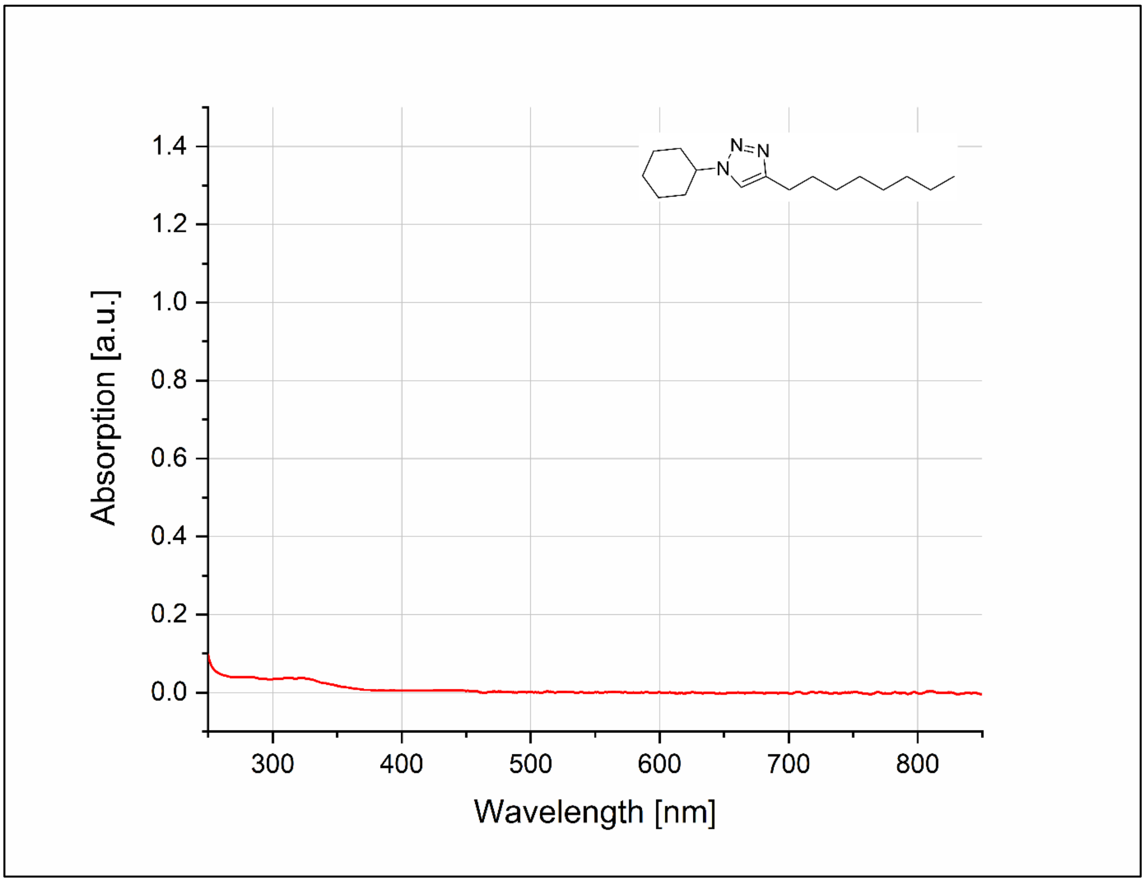

*Figure S 45. UV-Vis absorption spectrum of 2.52E-3 M 1-cyclohexyl-4-octyl-1H-1,2,3-triazole **3l** in DCM.*

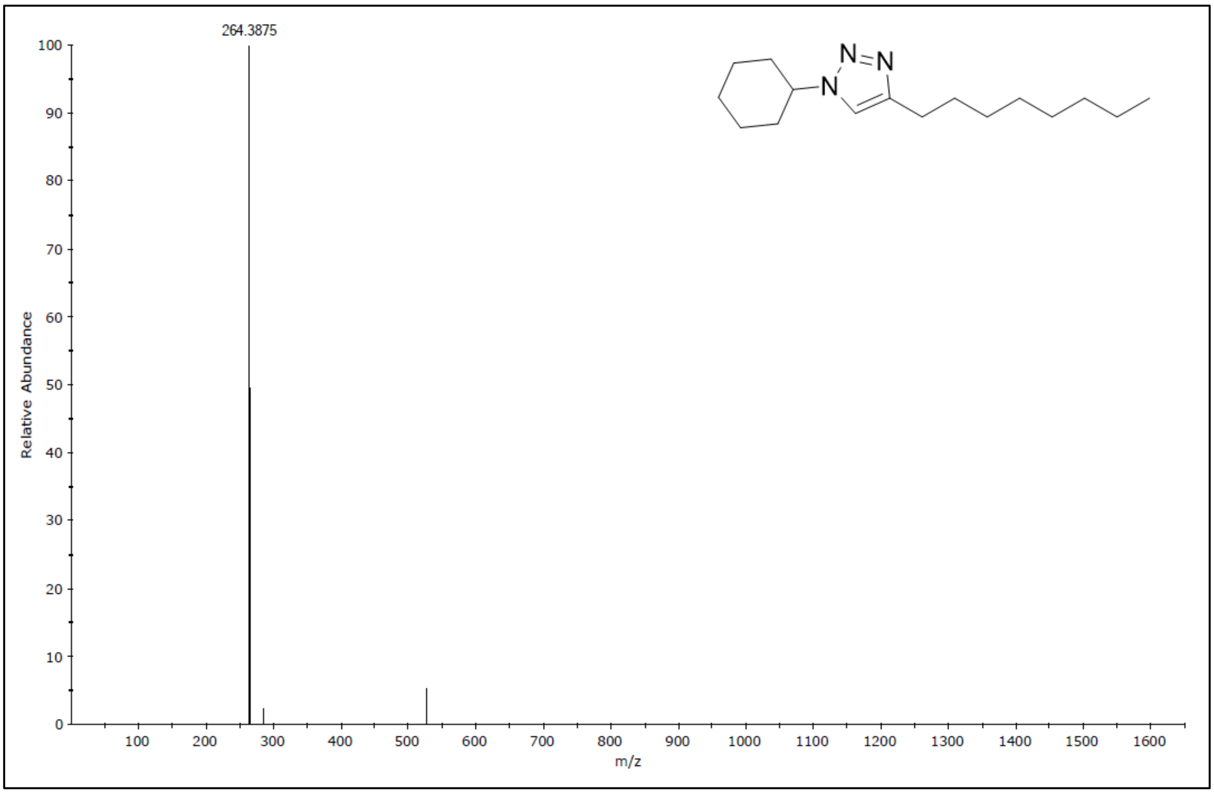

*Figure S 46. LR-ESI-MS spectrum of 1-cyclohexyl-4-octyl-1H-1,2,3-triazole **3l**.*

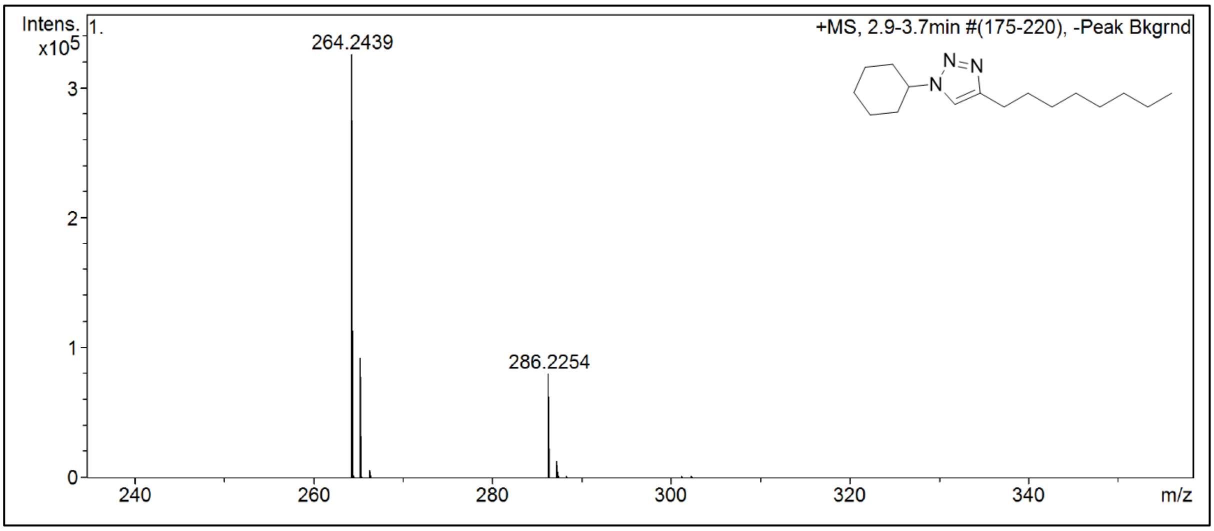

*Figure S 47. HR-ESI-MS spectrum of 1-cyclohexyl-4-octyl-1H-1,2,3-triazole **3l**.*

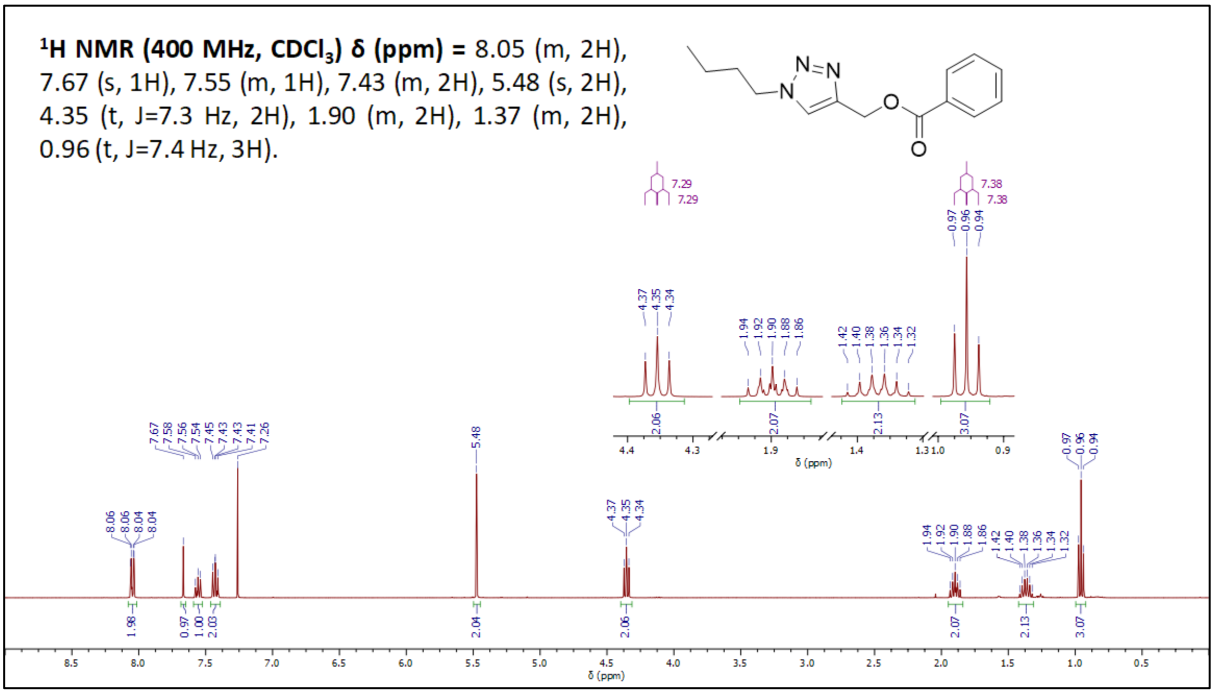

*Figure S 48. ^{1}H NMR spectrum of (1-butyl-1H-1,2,3-triazol-4-yl)methyl benzoate **3m**.*

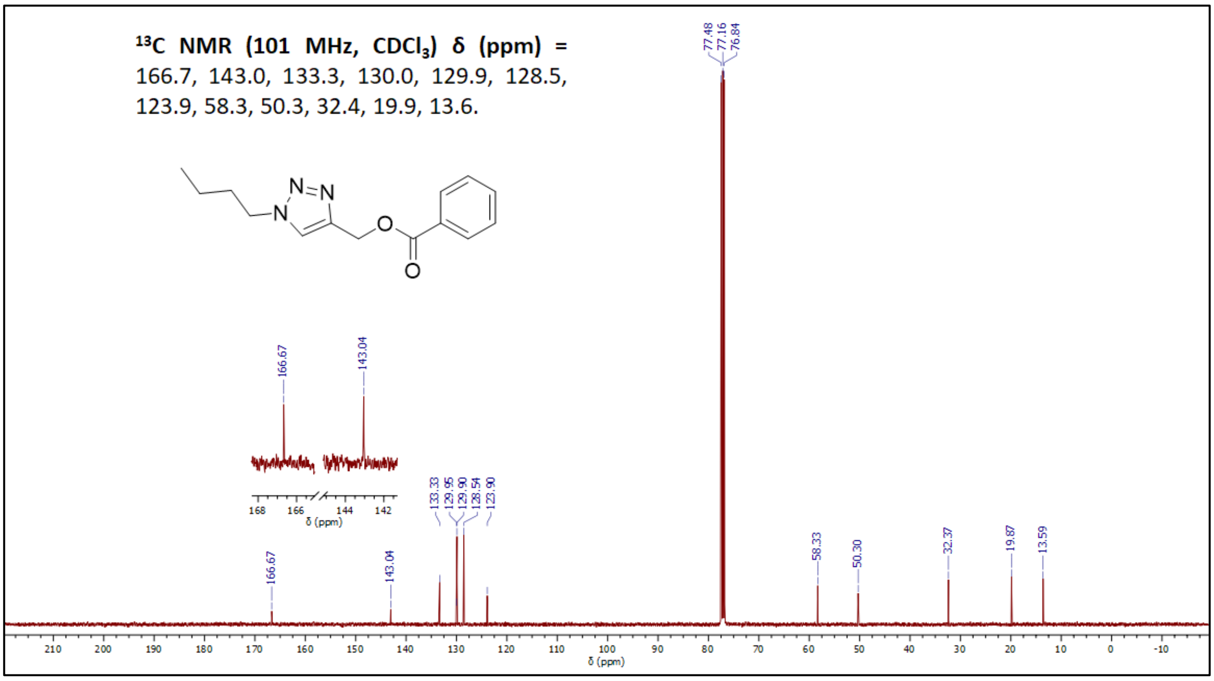

*Figure S 49. ^{13}C NMR spectrum of (1-butyl-1H-1,2,3-triazol-4-yl)methyl benzoate **3m**.*

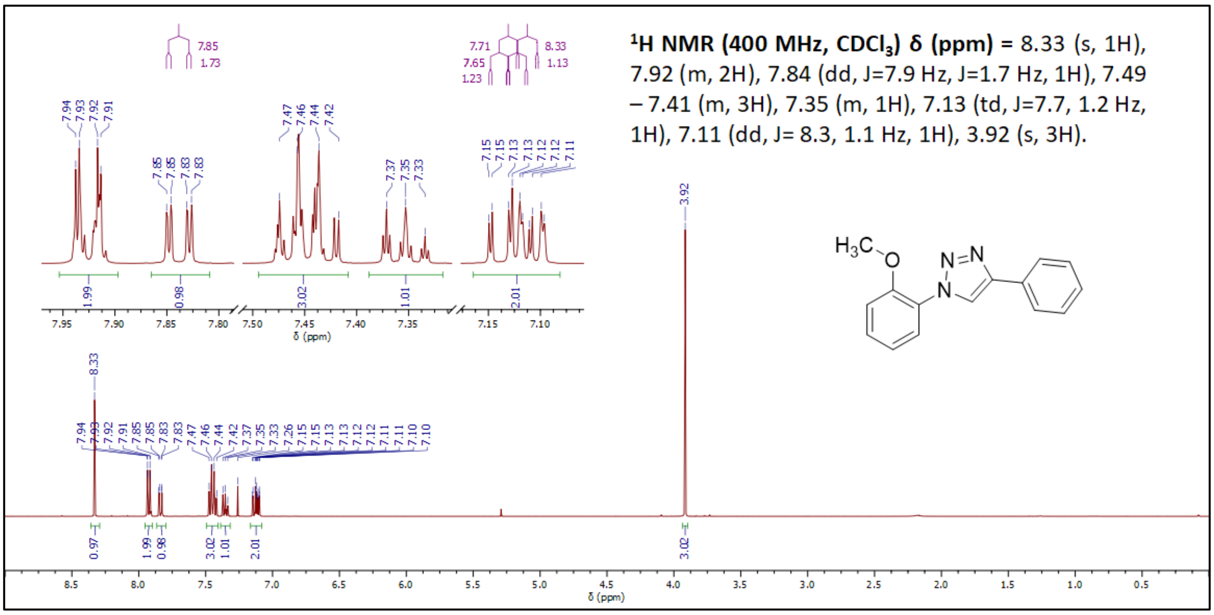

*Figure S 50. ^{1}H NMR spectrum of 1-(2-methoxyphenyl)-4-phenyl-1H-1,2,3-triazole **3n**.*

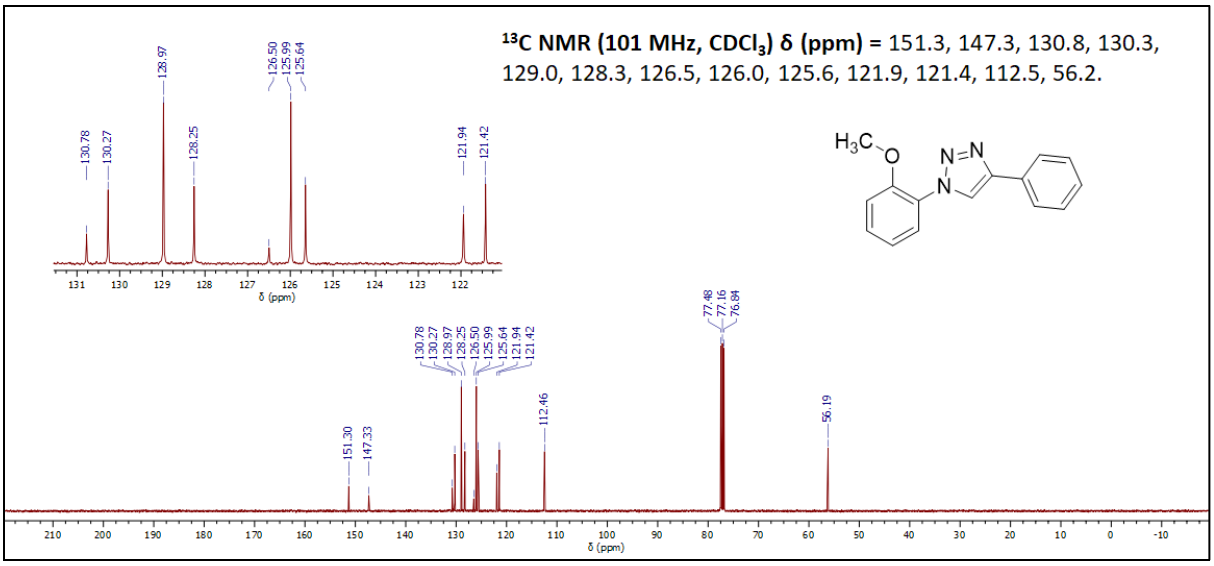

*Figure S 51. ^{13}C NMR spectrum of 1-(2-methoxyphenyl)-4-phenyl-1H-1,2,3-triazole **3n**.*

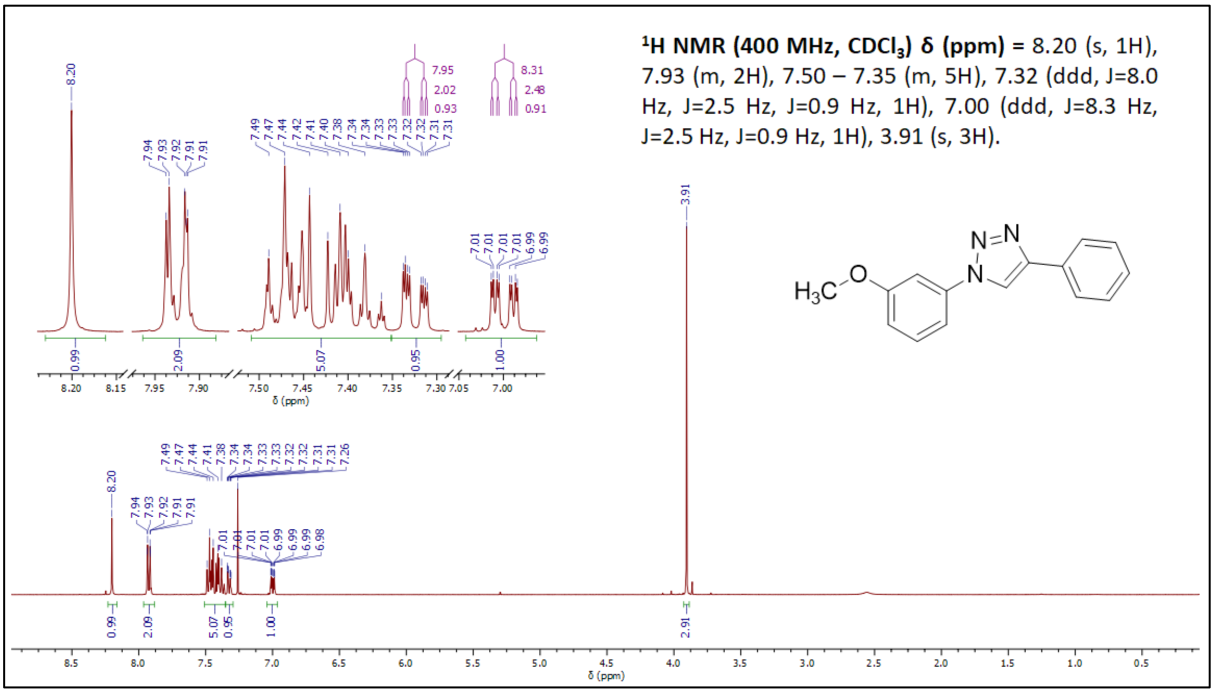

*Figure S 52. ^{1}H NMR spectrum of 1-(3-methoxyphenyl)-4-phenyl-1H-1,2,3-triazole **3o**.*

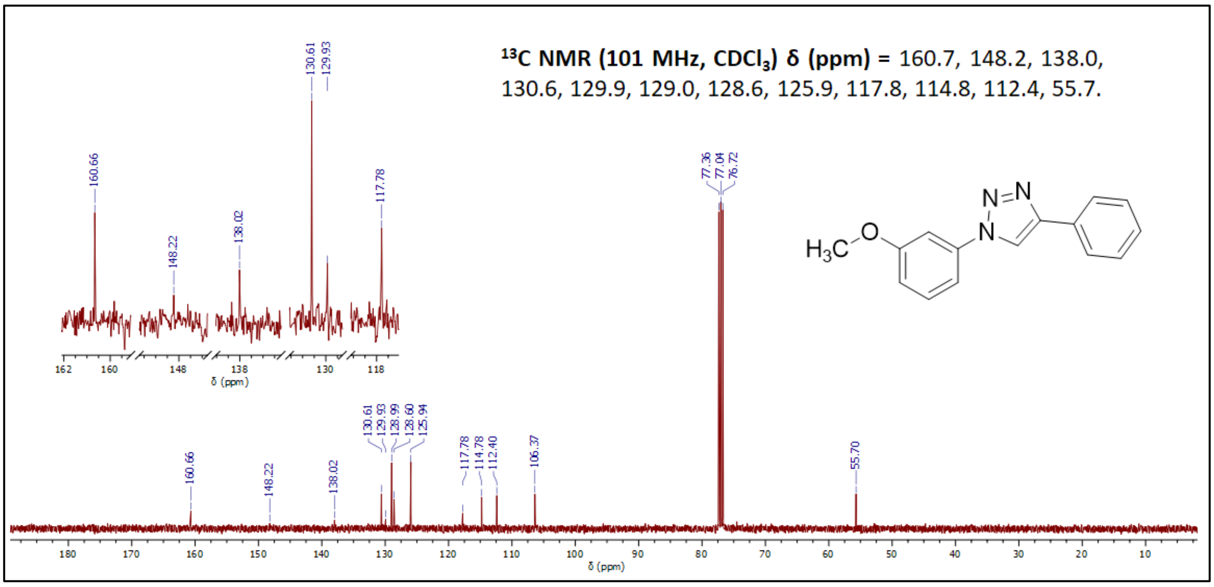

*Figure S 53. ^{13}C NMR spectrum of 1-(3-methoxyphenyl)-4-phenyl-1H-1,2,3-triazole **3o**.*

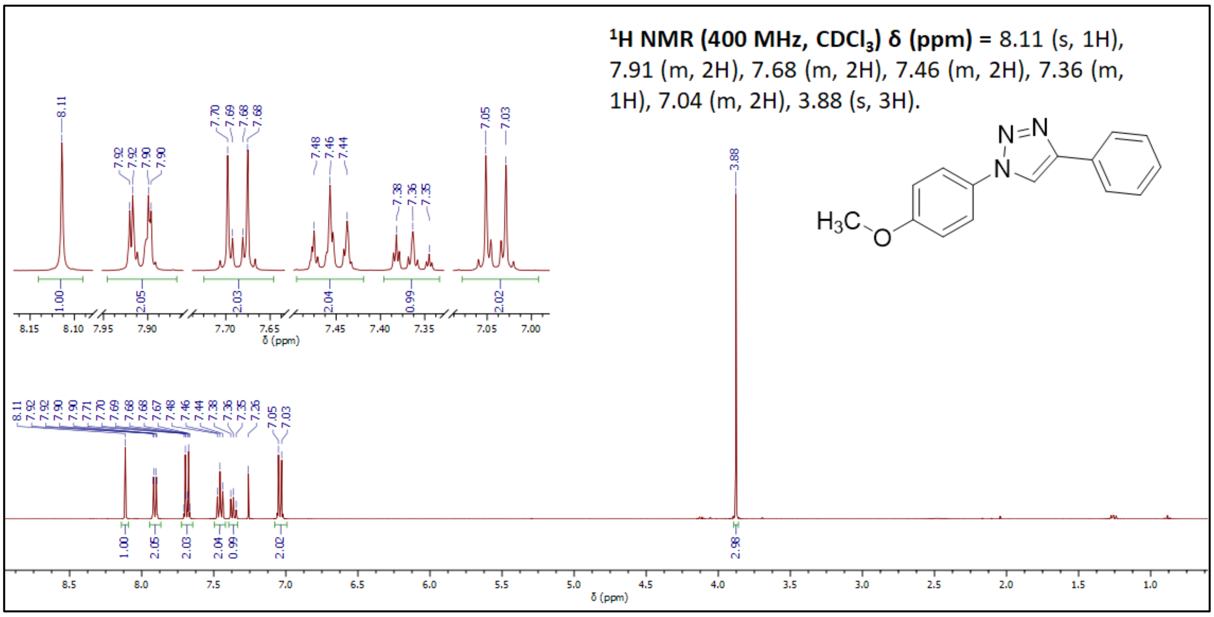

Figure S 54. ^{1}H NMR spectrum of 1-(4-methoxyphenyl)-4-phenyl-1H-1,2,3-triazole ***3p***

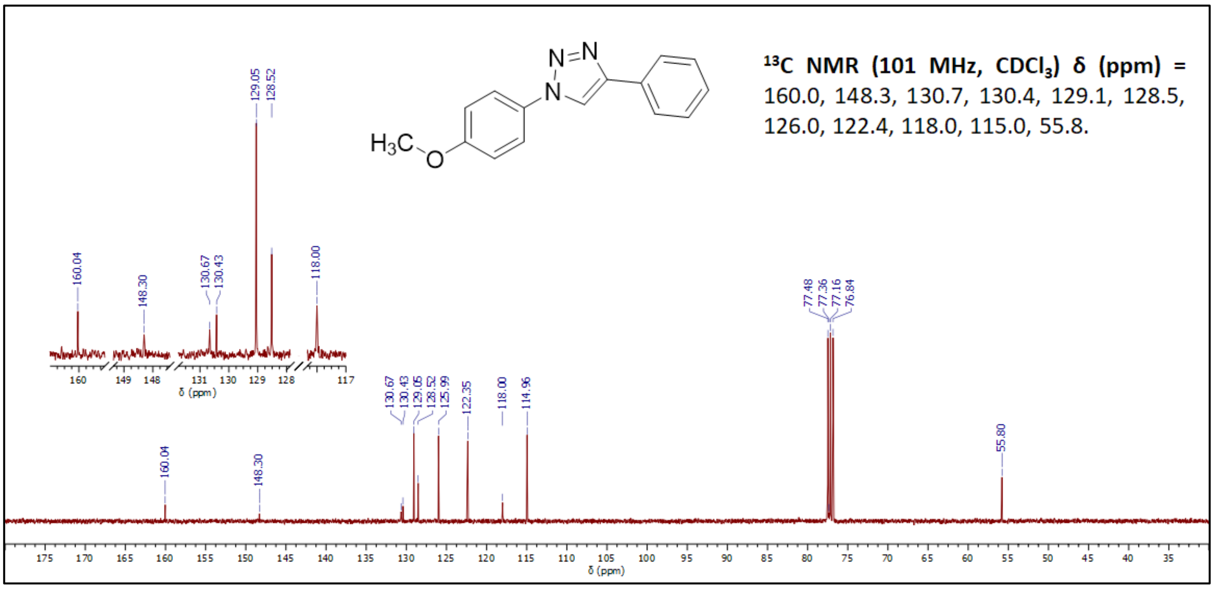

Figure S 55. ^{13}C NMR spectrum of 1-(4-methoxyphenyl)-4-phenyl-1H-1,2,3-triazole ***3p****.*

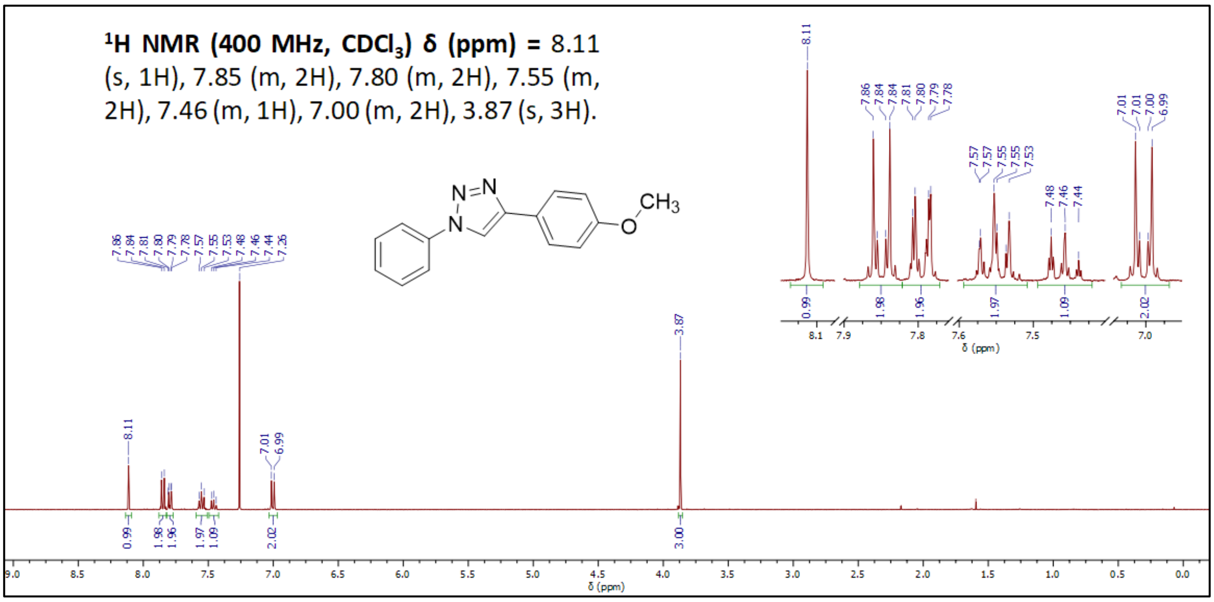

*Figure S 56. ¹H NMR spectrum of 1-phenyl-4-(4-methoxyphenyl)-1H-1,2,3-triazole **3q**.*

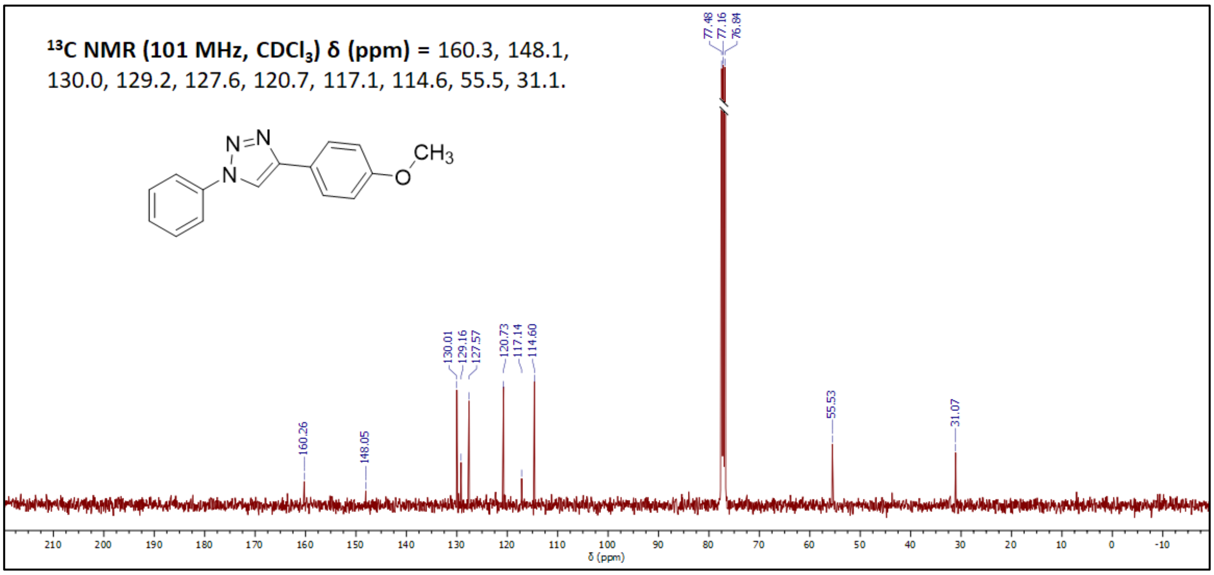

*Figure S 57. ¹³C NMR spectrum of 1-phenyl-4-(4-methoxyphenyl)-1H-1,2,3-triazole **3q**.*

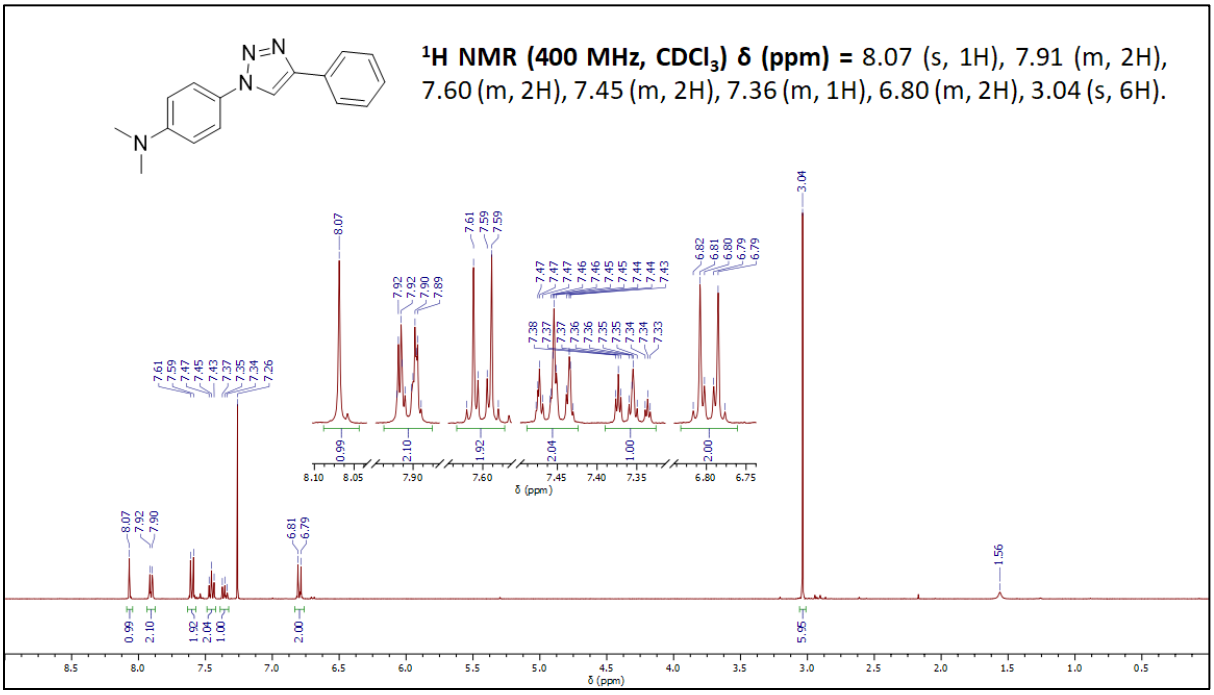

Figure S 58. ^{1}H NMR spectrum of N,N-Dimethyl-4-(4-phenyl-1H-1,2,3-triazol-1-yl)aniline ***3r.***

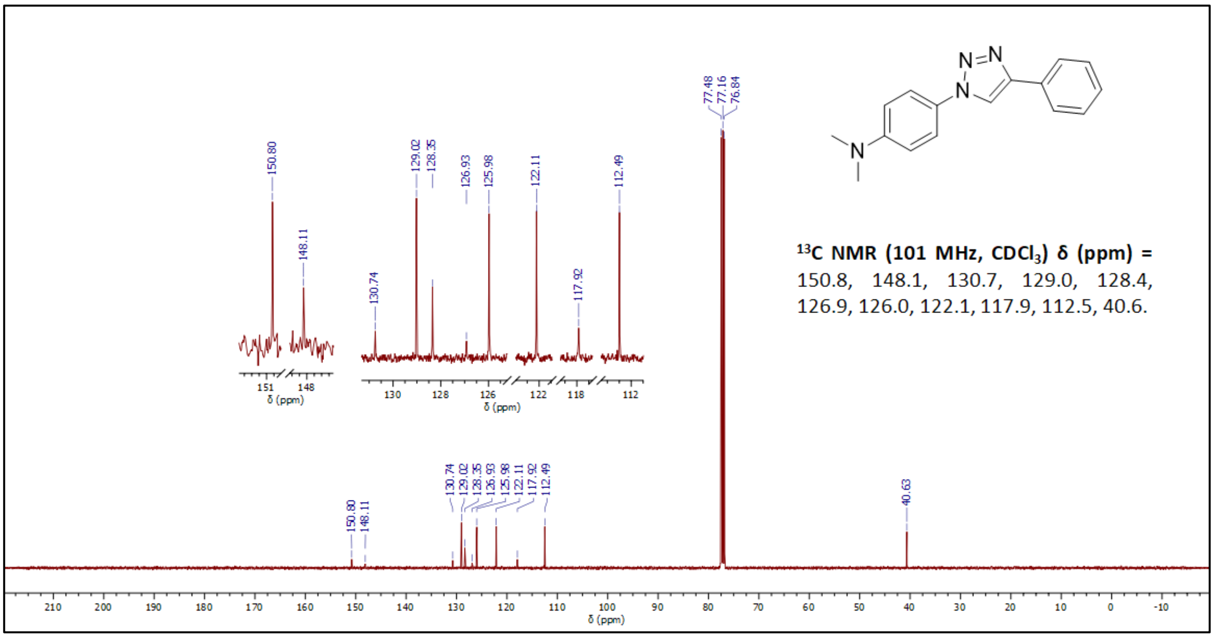

Figure S 59. ^{13}C NMR spectrum of N,N-Dimethyl-4-(4-phenyl-1H-1,2,3-triazol-1-yl)aniline ***3r.***

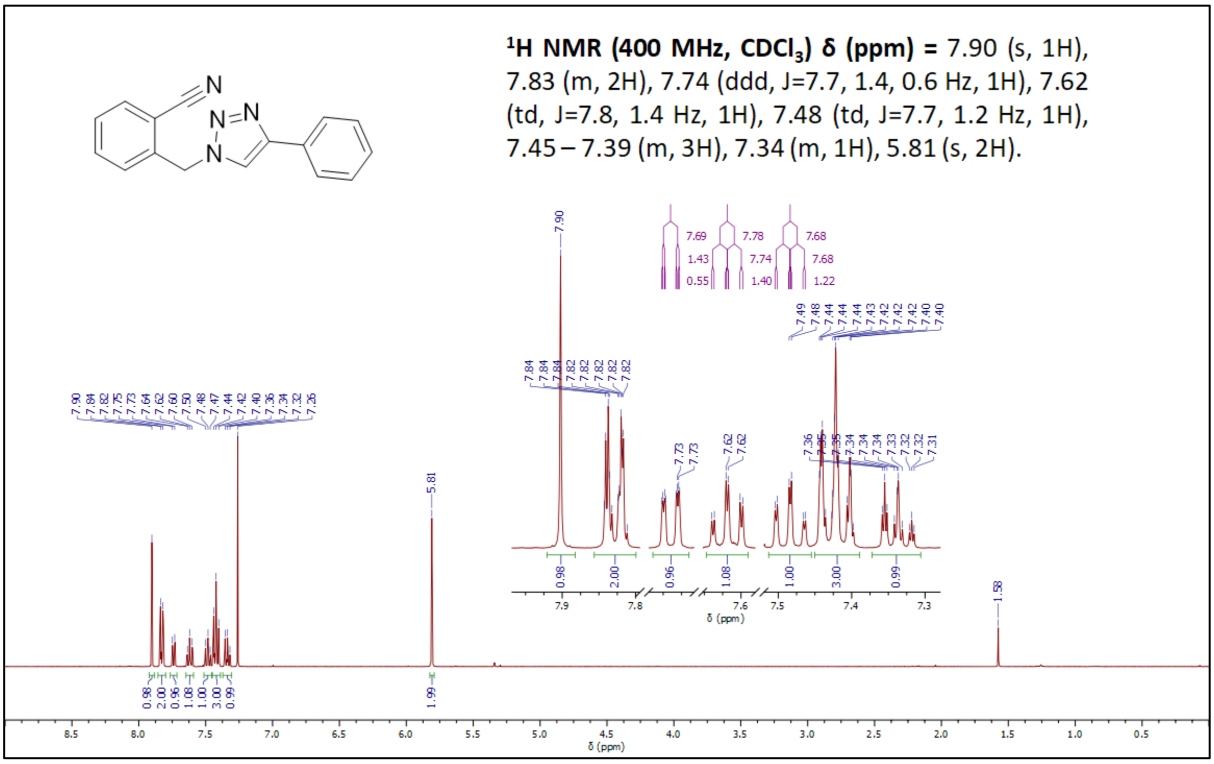

Figure S 60. ¹H NMR spectrum of 2-((4-Phenyl-1H-1,2,3-triazol-1-yl)methyl)benzonitrile ***3s.***

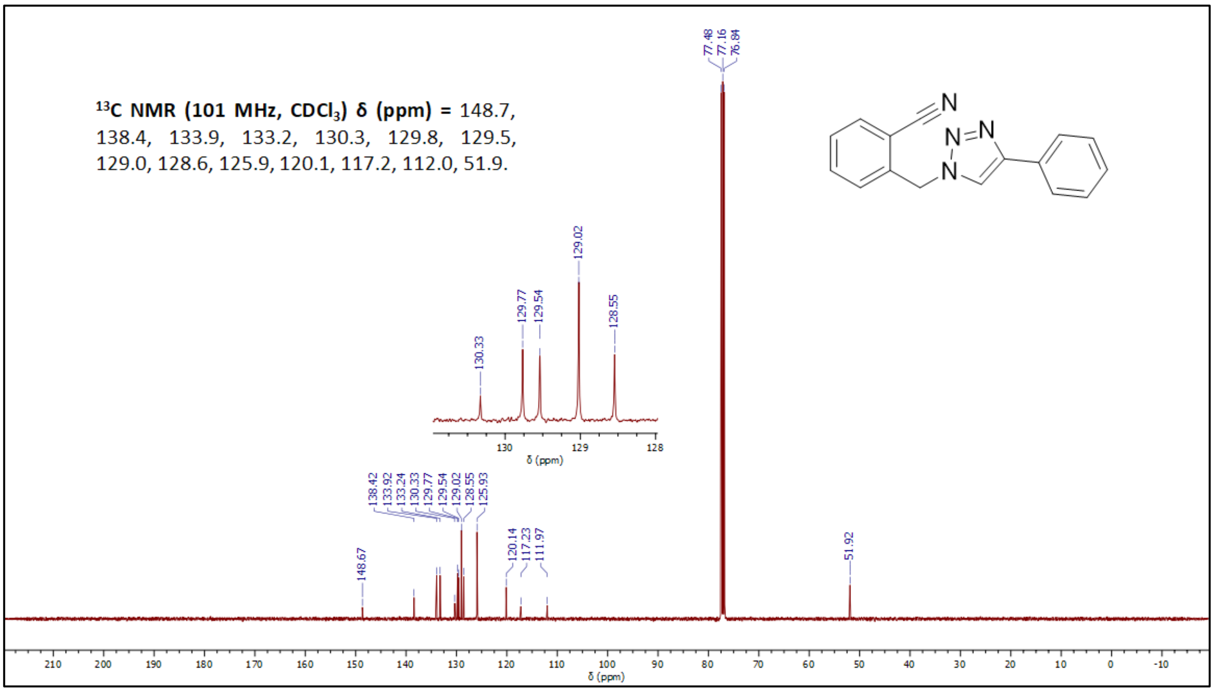

Figure S 61. ¹³C NMR spectrum of 2-((4-Phenyl-1H-1,2,3-triazol-1-yl)methyl)benzonitrile ***3s.***

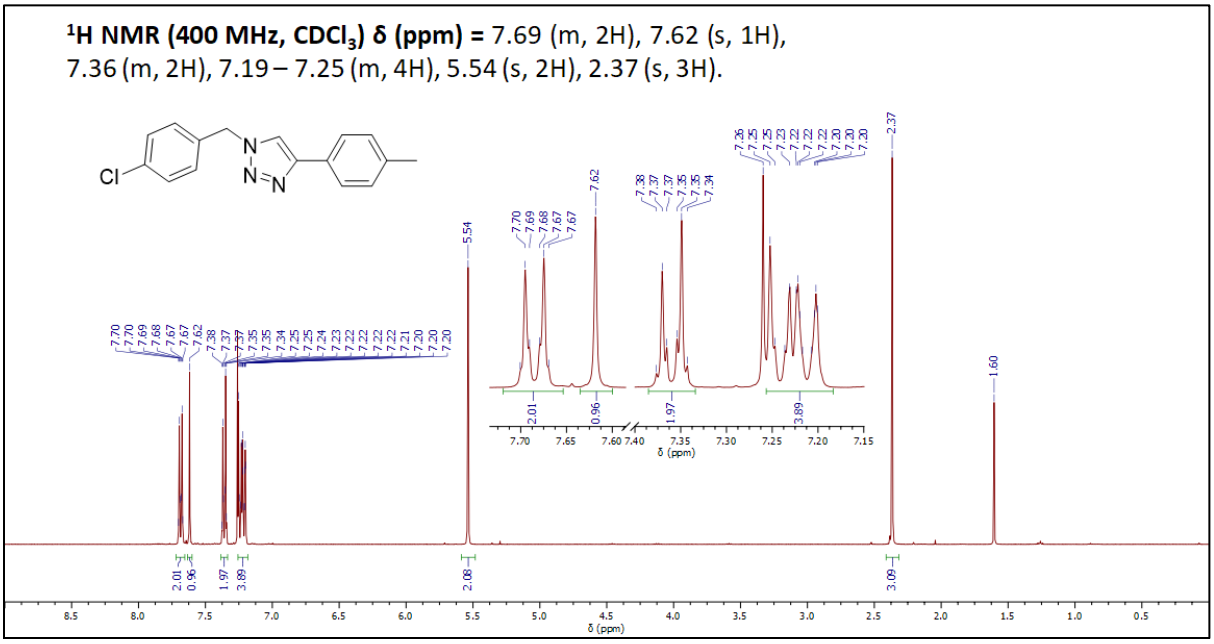

*Figure S 62. ^{1}H NMR spectrum of 1-(4-Chlorobenzyl)-4-(p-tolyl)-1H-1,2,3-triazole **3t.***

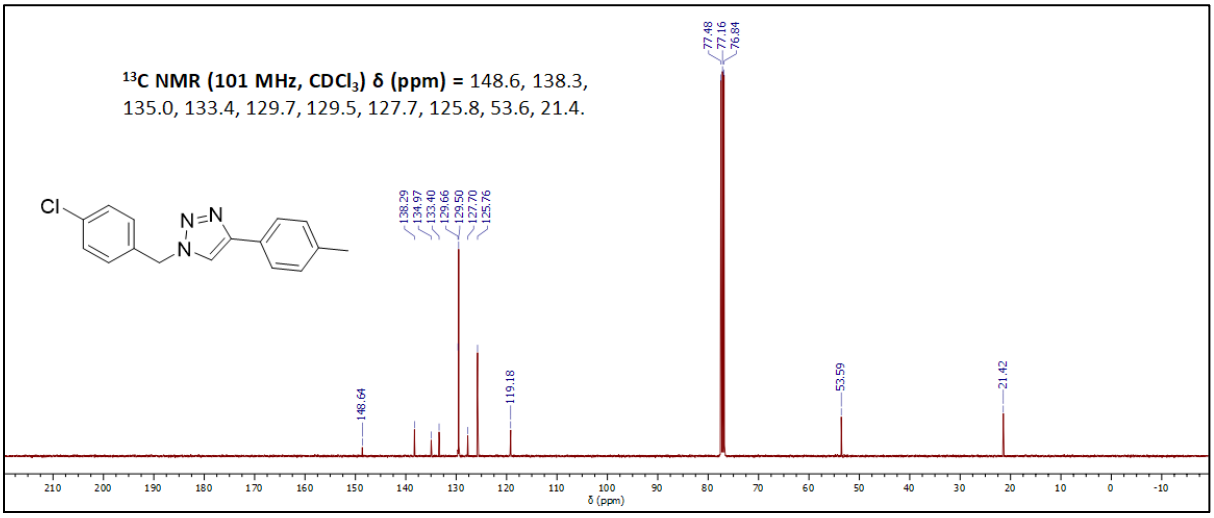

*Figure S 63. ^{13}C NMR spectrum of 1-(4-Chlorobenzyl)-4-(p-tolyl)-1H-1,2,3-triazole **3t.***

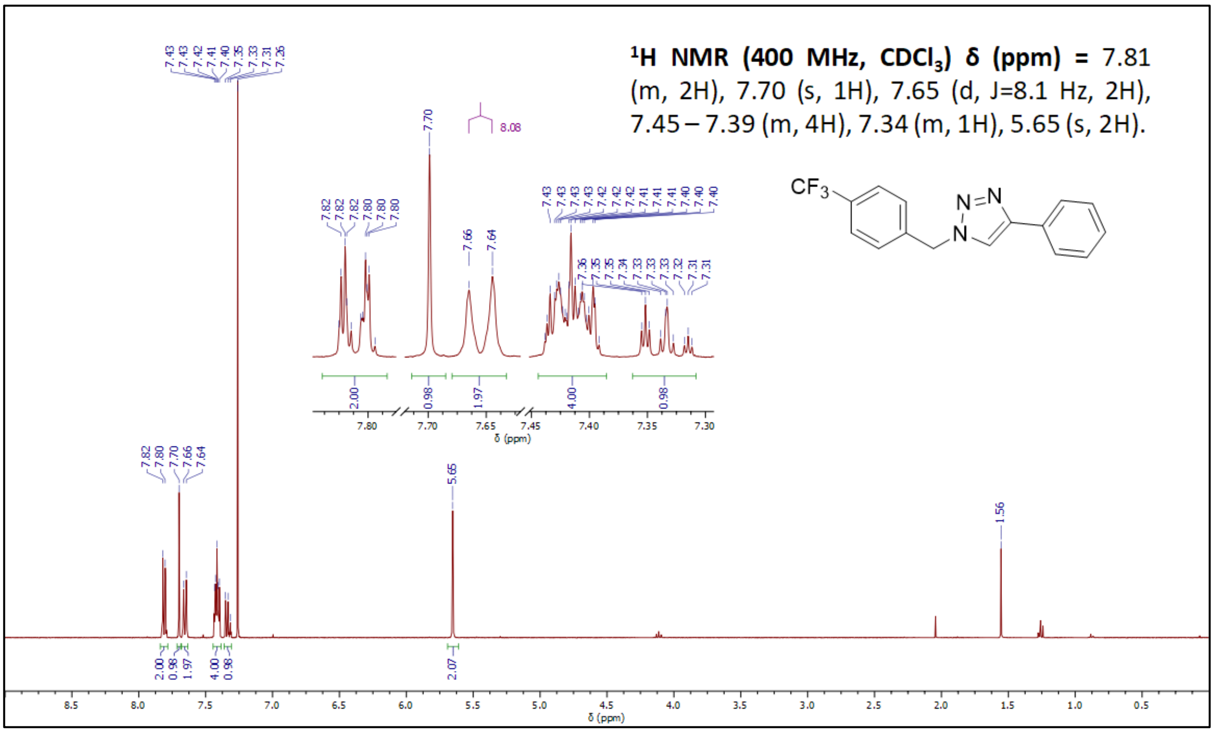

Figure S 64. ^{1}H NMR spectrum of 4-Phenyl-1-(4-(trifluoromethyl)benzyl)-1H-1,2,3-triazole ***3u.***

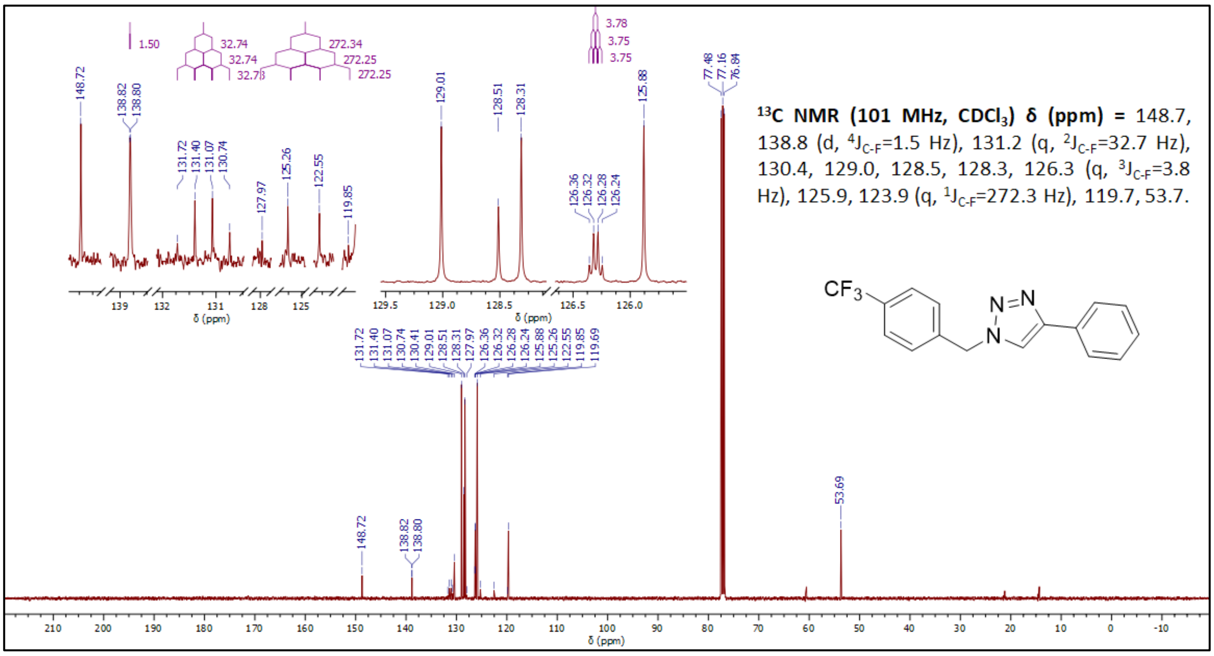

Figure S 65. ^{13}C NMR spectrum of 4-Phenyl-1-(4-(trifluoromethyl)benzyl)-1H-1,2,3-triazole ***3u.***

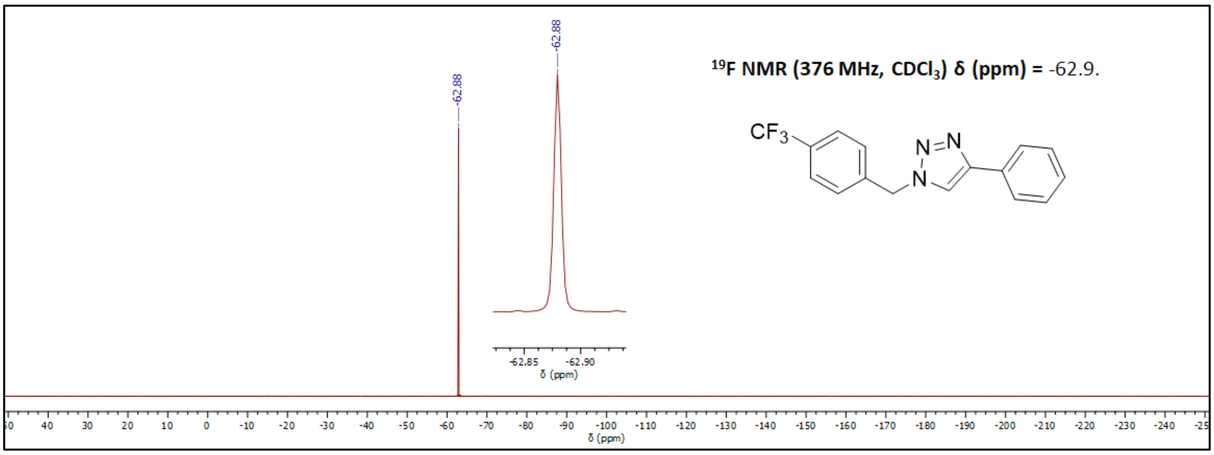

*Figure S 66. ^{19}F NMR spectrum of 4-Phenyl-1-(4-(trifluoromethyl)benzyl)-1H-1,2,3-triazole **3u.***

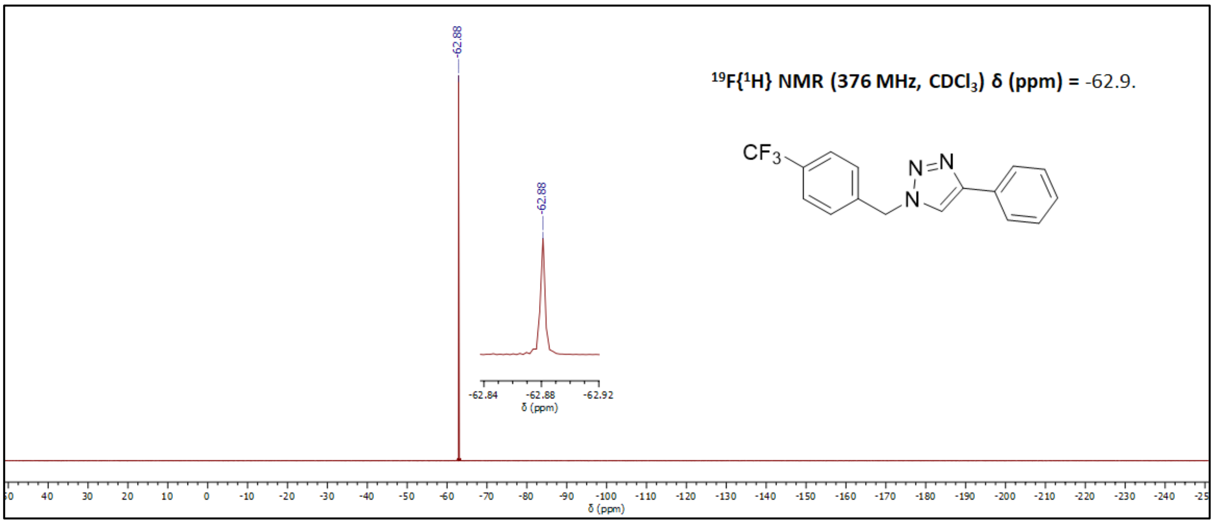

*Figure S 67. ^{19}F{^{1}H} NMR spectrum of 4-Phenyl-1-(4-(trifluoromethyl)benzyl)-1H-1,2,3-triazole **3u.***

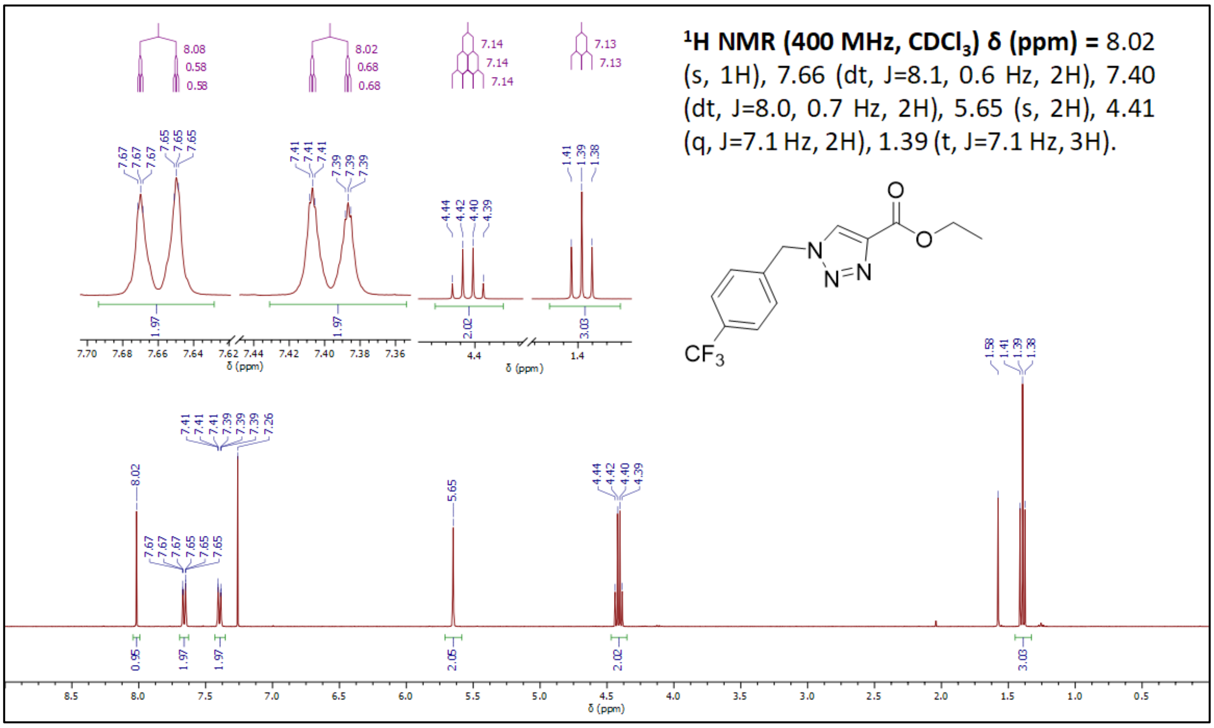

*Figure S 68. ^{1}H NMR spectrum of Ethyl 1-(4-(trifluoromethyl)benzyl)-1H-1,2,3-triazole-4-carboxylate **3v.***

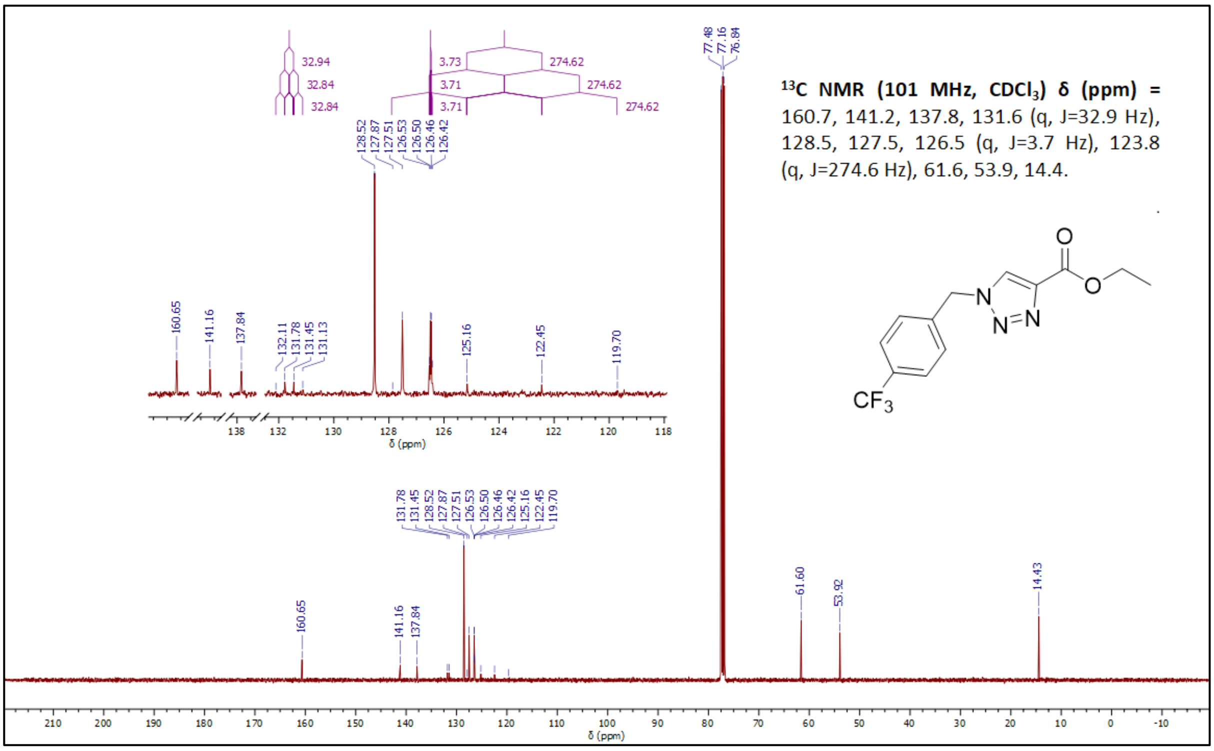

*Figure S 69. ^{13}C NMR spectrum of Ethyl 1-(4-(trifluoromethyl)benzyl)-1H-1,2,3-triazole-4-carboxylate **3v.***

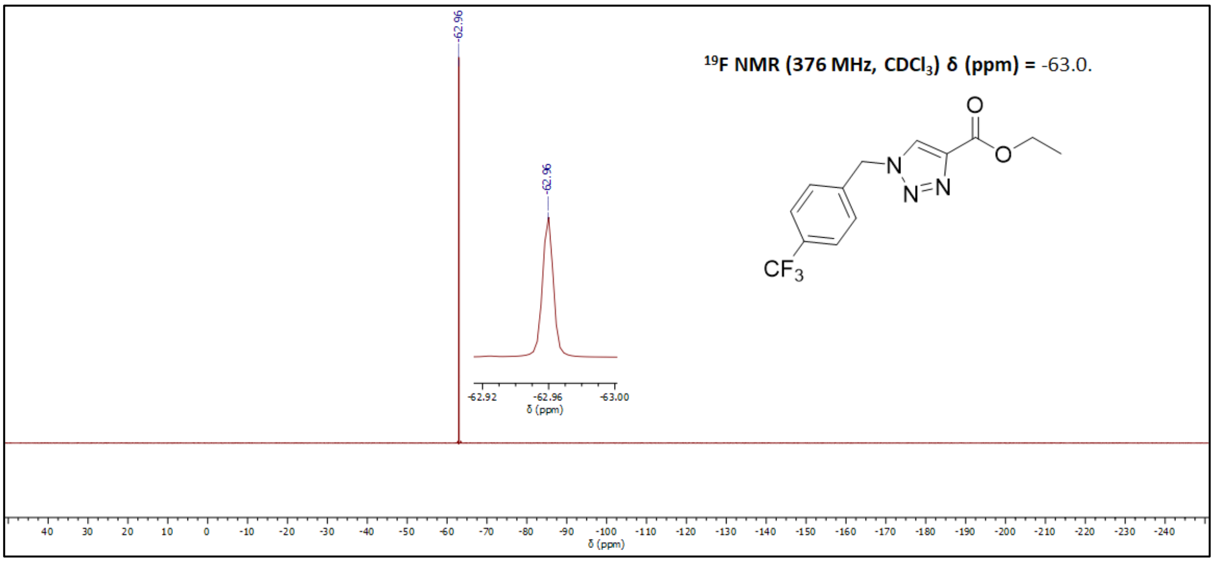

*Figure S 70. ^{19}F NMR spectrum of Ethyl 1-(4-(trifluoromethyl)benzyl)-1H-1,2,3-triazole-4-carboxylate **3v.***

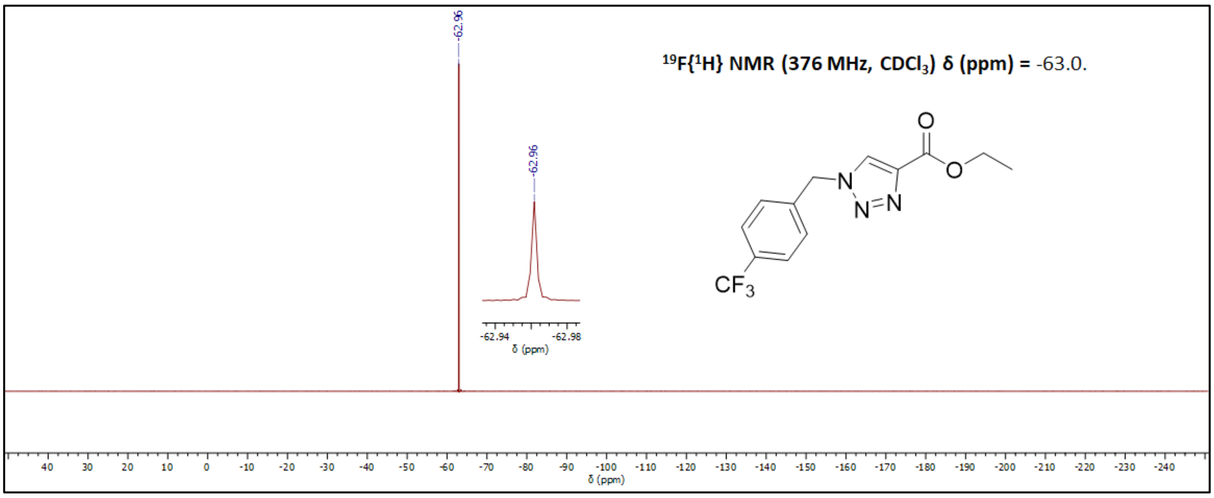

*Figure S 71. $^{19}F\{^1H\}$ NMR spectrum of Ethyl 1-(4-(trifluoromethyl)benzyl)-1H-1,2,3-triazole-4-carboxylate **3v.***

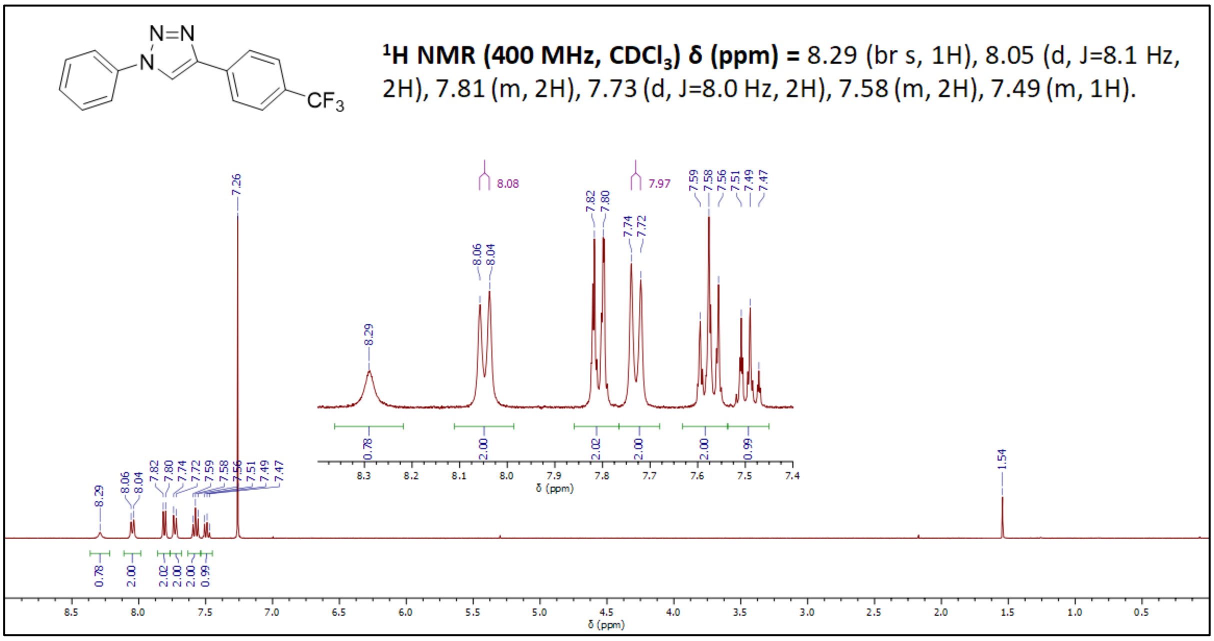

*Figure S 72. ^1H NMR spectrum of 1-Phenyl-4-(4-(trifluoromethyl)phenyl)-1H-1,2,3-triazole **3w.***

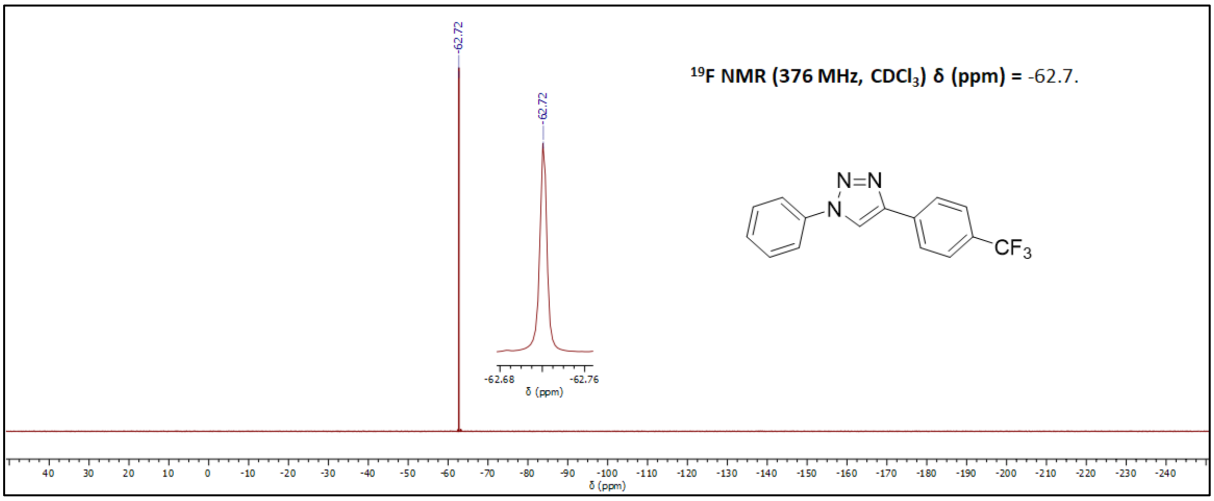

*Figure S 73. ^{19}F NMR spectrum of 1-Phenyl-4-(4-(trifluoromethyl)phenyl)-1H-1,2,3-triazole **3w.***

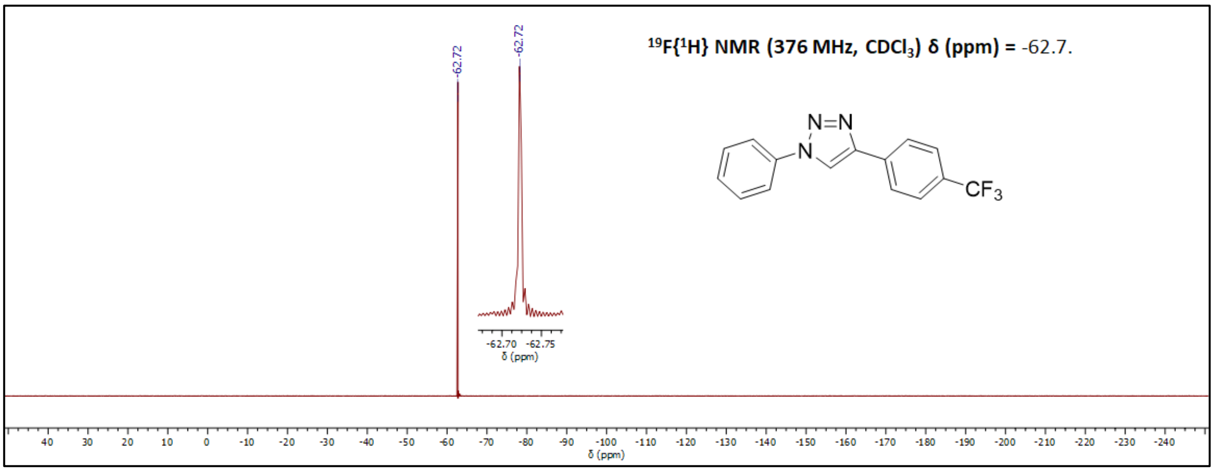

*Figure S 74. $^{19}F\{^1H\}$ NMR spectrum of 1-Phenyl-4-(4-(trifluoromethyl)phenyl)-1H-1,2,3-triazole **3w**.*

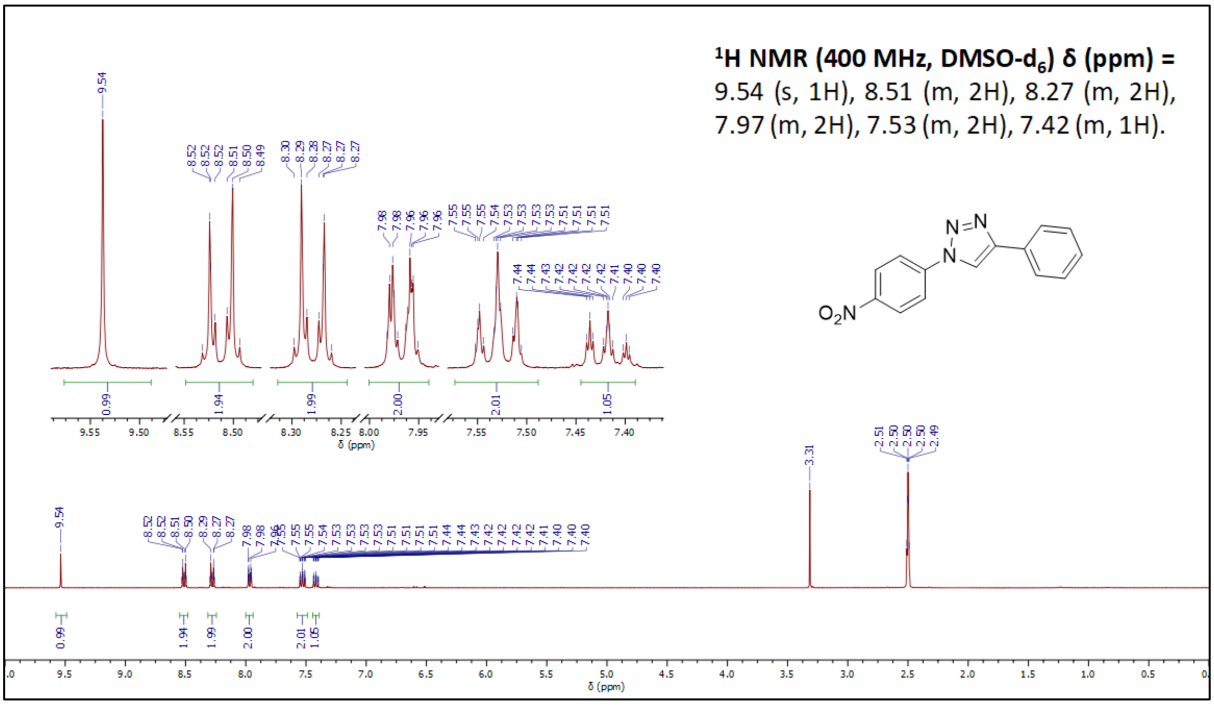

*Figure S 75. ^1H NMR spectrum of 1-(4-nitrophenyl)-4-phenyl-1H-1,2,3-triazole **3x**.*

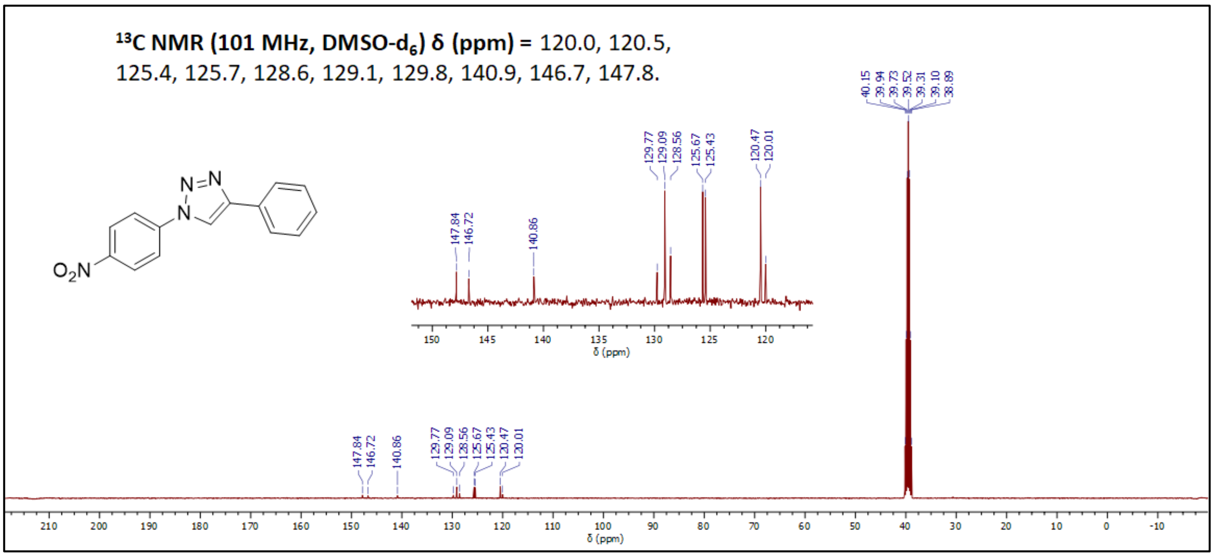

Figure S 76. ^{13}C NMR spectrum of 1-(4-nitrophenyl)-4-phenyl-1H-1,2,3-triazole ***3x****.*

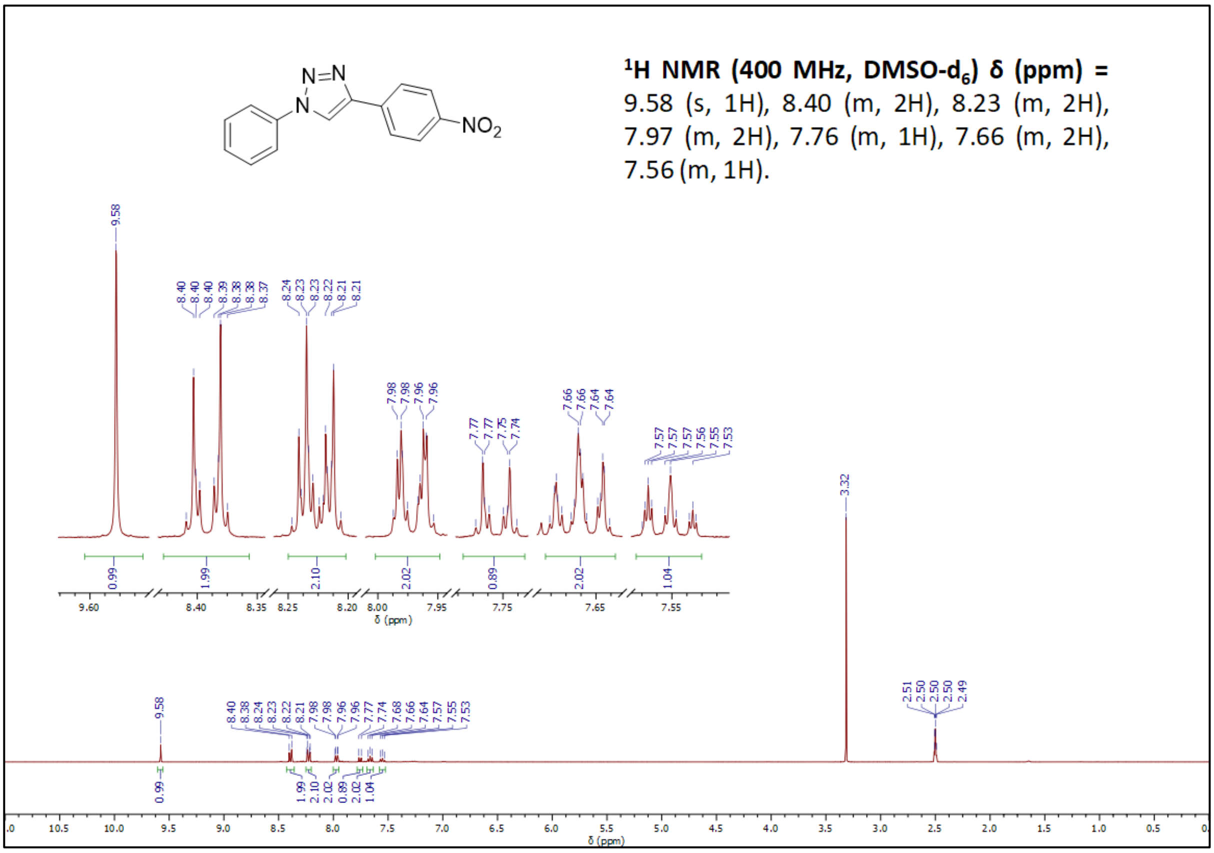

Figure S 77. ^{1}H NMR spectrum of 4-(4-nitrophenyl)-1-phenyl-1H-1,2,3-triazole ***3y****.*

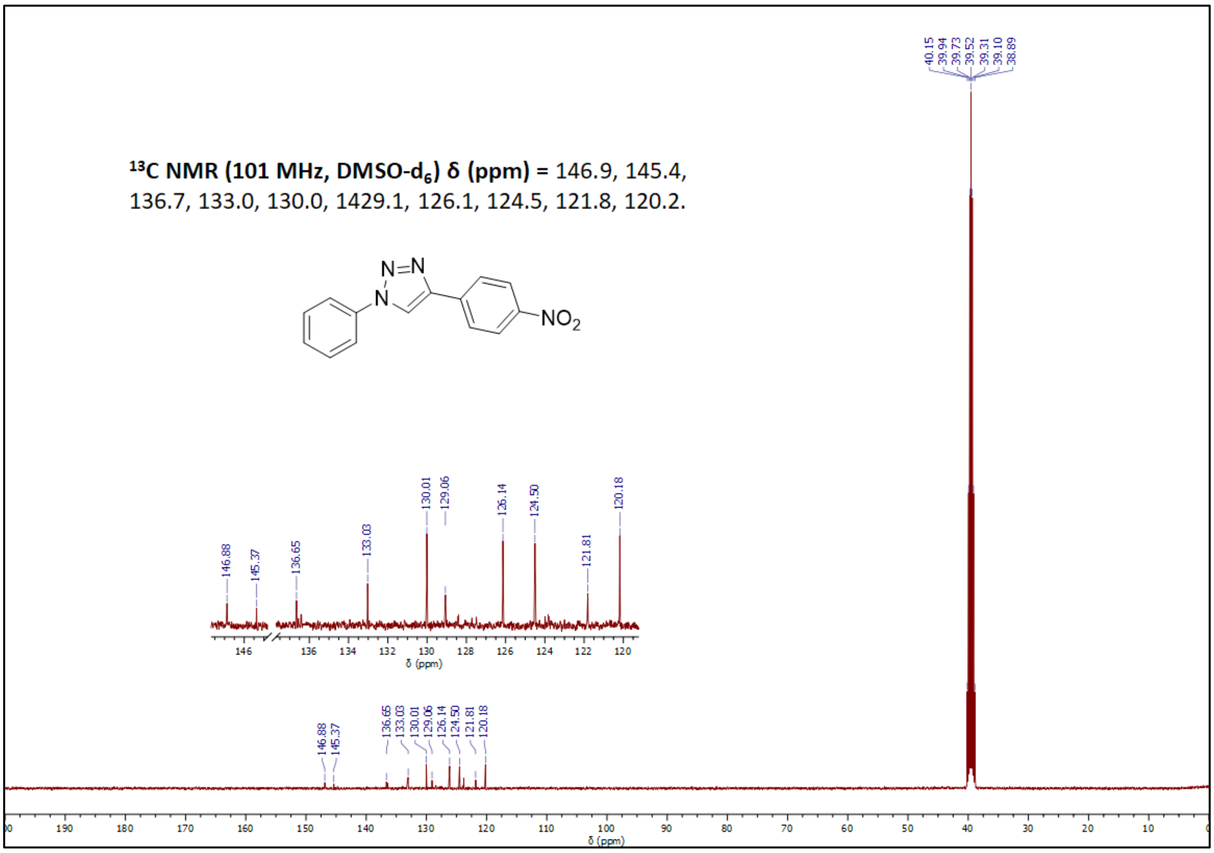

*Figure S 78. ^{13}C NMR spectrum of 4-(4-nitrophenyl)-1-phenyl-1H-1,2,3-triazole **3y**.*

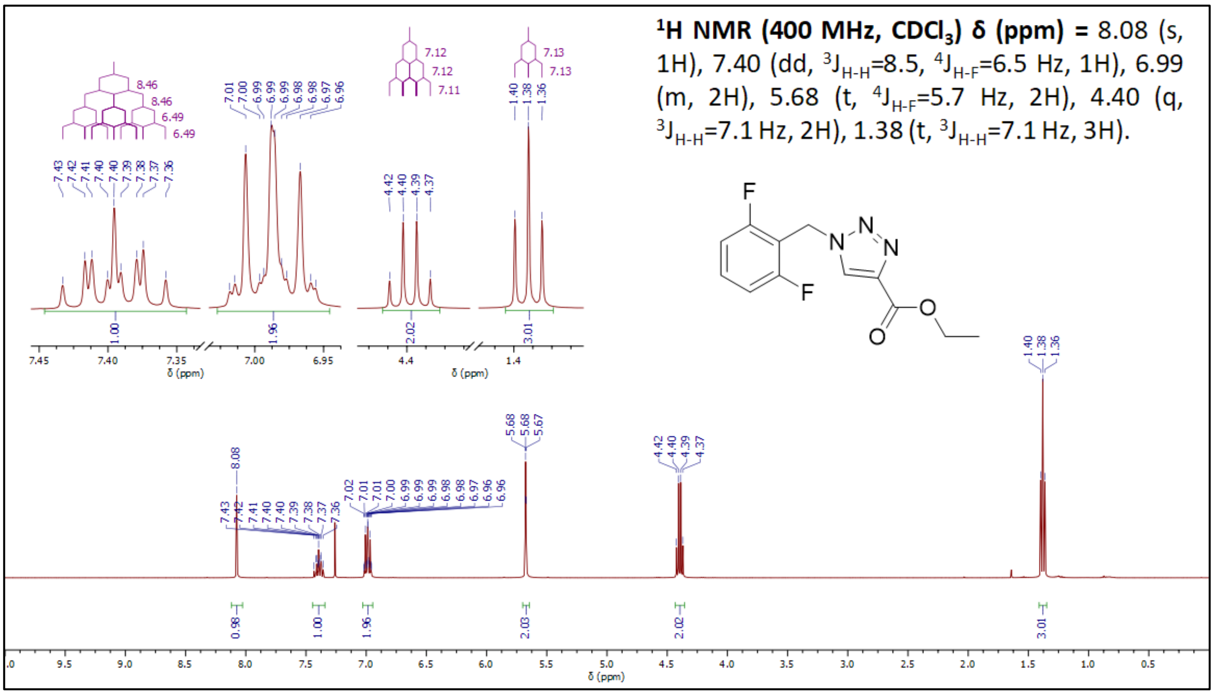

*Figure S 79. ^{1}H NMR spectrum of 1-(2,6-difluorobenzyl)-1H-1,2,3-triazole-4-carboxylic acid ethyl ester **3z**.*

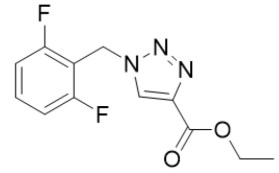

^{13}C NMR (101 MHz, CDCl$_3$) δ (ppm) = 161.5 (dd, $^1J_{C-F}$=251.7, $^3J_{C-F}$=6.8 Hz, C), 160.8 (s, C), 140.7 (s, C), 132.0 (t, $^3J_{C-F}$=10.4 Hz, CH), 127.5 (t, J_{C-F}=1.7 Hz, CH), 112.1 (dd, $^2J_{C-F}$=19.2, $^4J_{C-F}$=5.7 Hz, CH), 110.2 (t, $^2J_{C-F}$=18.9 Hz, C), 61.5 (s, CH_2), 41.8 (t, $^3J_{C-F}$=4.1 Hz, CH_2), 14.4 (s, CH_3).

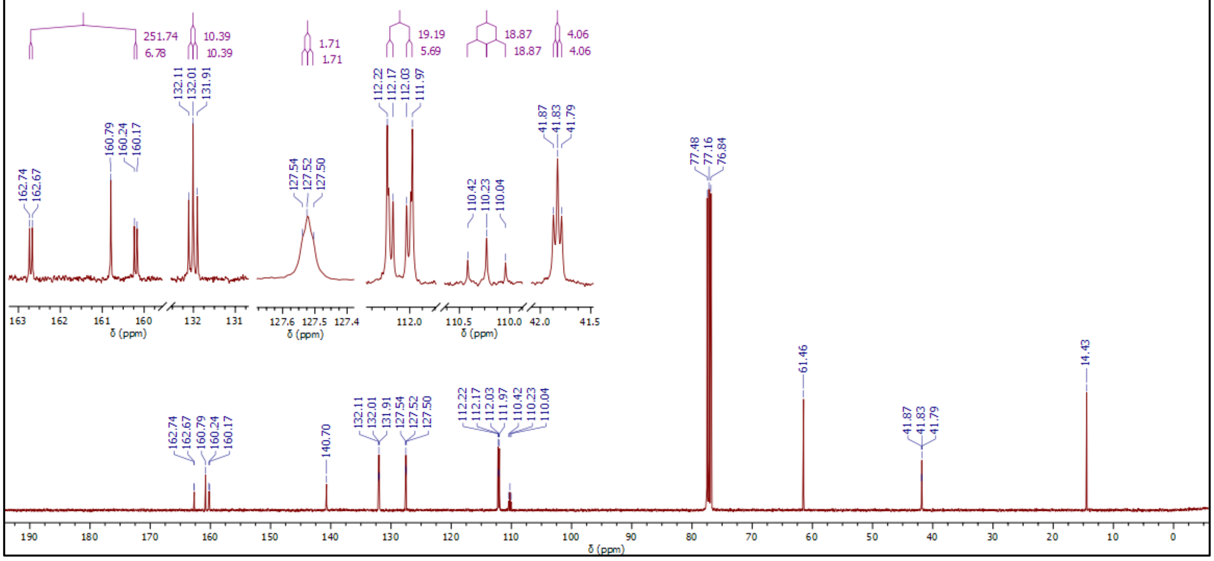

*Figure S 80. ^{13}C NMR spectrum of 1-(2,6-difluorobenzyl)-1H-1,2,3-triazole-4-carboxylic acid ethyl ester **3z**.*

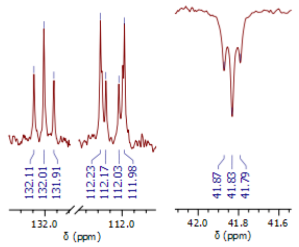

DEPT-135 NMR (101 MHz, CDCl$_3$) δ (ppm) = 132.0 (CH), 127.5 (CH), 112.1 (CH), 61.5 (CH_2), 41.8 (CH_2), 14.4 (CH_3).

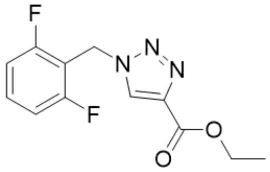

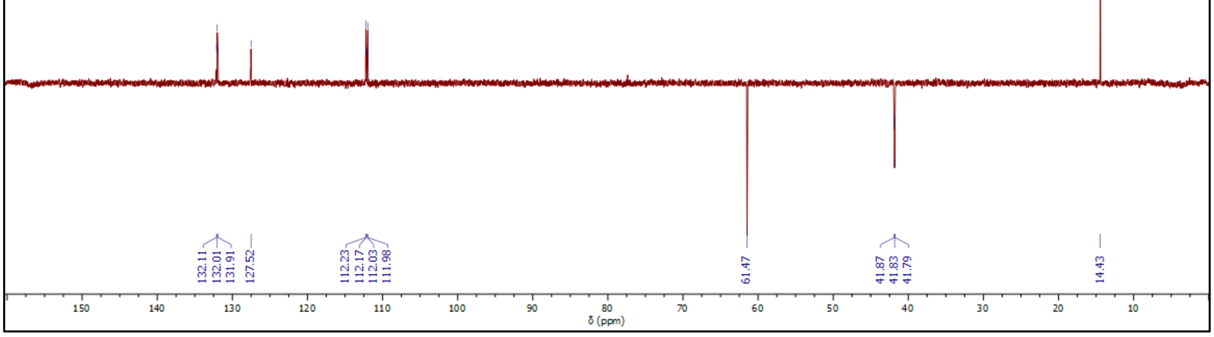

*Figure S 81. DEPT-135 NMR spectrum of 1-(2,6-difluorobenzyl)-1H-1,2,3-triazole-4-carboxylic acid ethyl ester **3z**.*

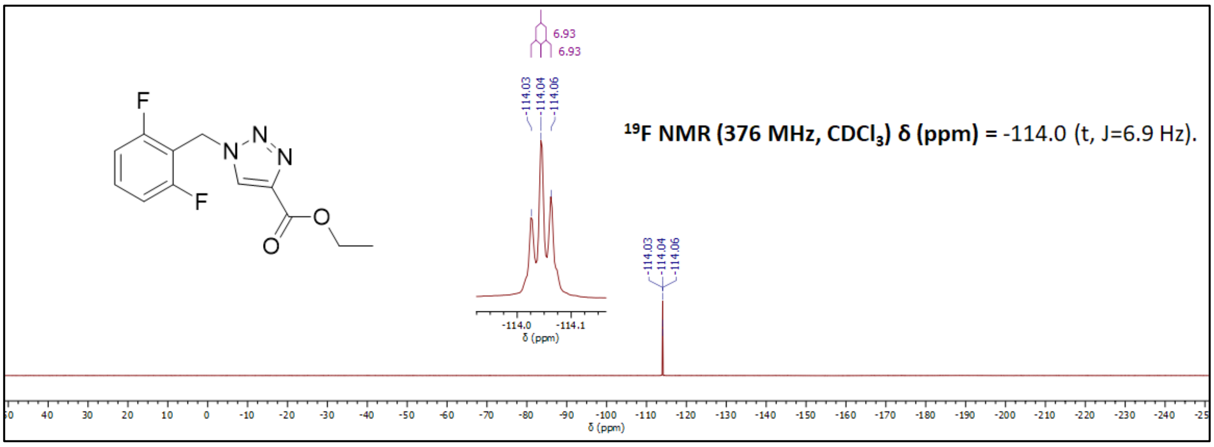

Figure S 82. *¹⁹F NMR spectrum of 1-(2,6-difluorobenzyl)-1H-1,2,3-triazole-4-carboxylic acid ethyl ester* ***3z****.*

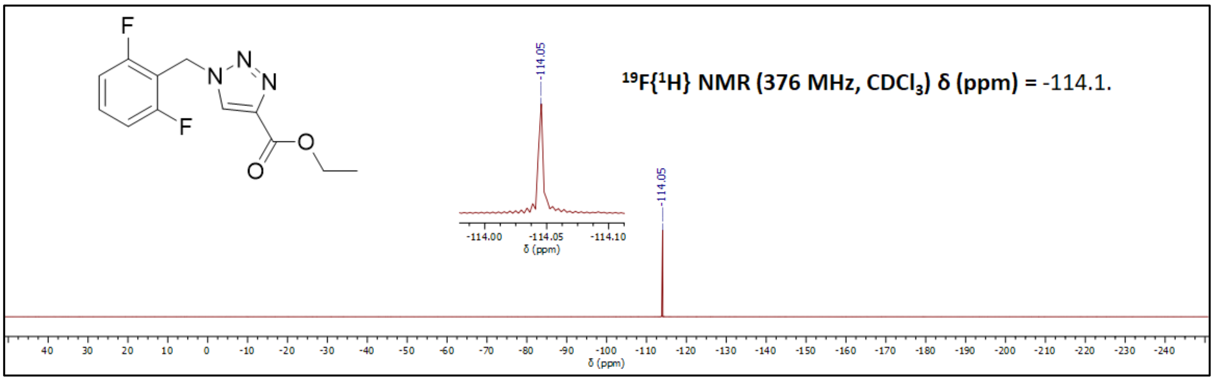

Figure S 83. *¹⁹F{¹H} NMR spectrum of 1-(2,6-difluorobenzyl)-1H-1,2,3-triazole-4-carboxylic acid ethyl ester* ***3z****.*

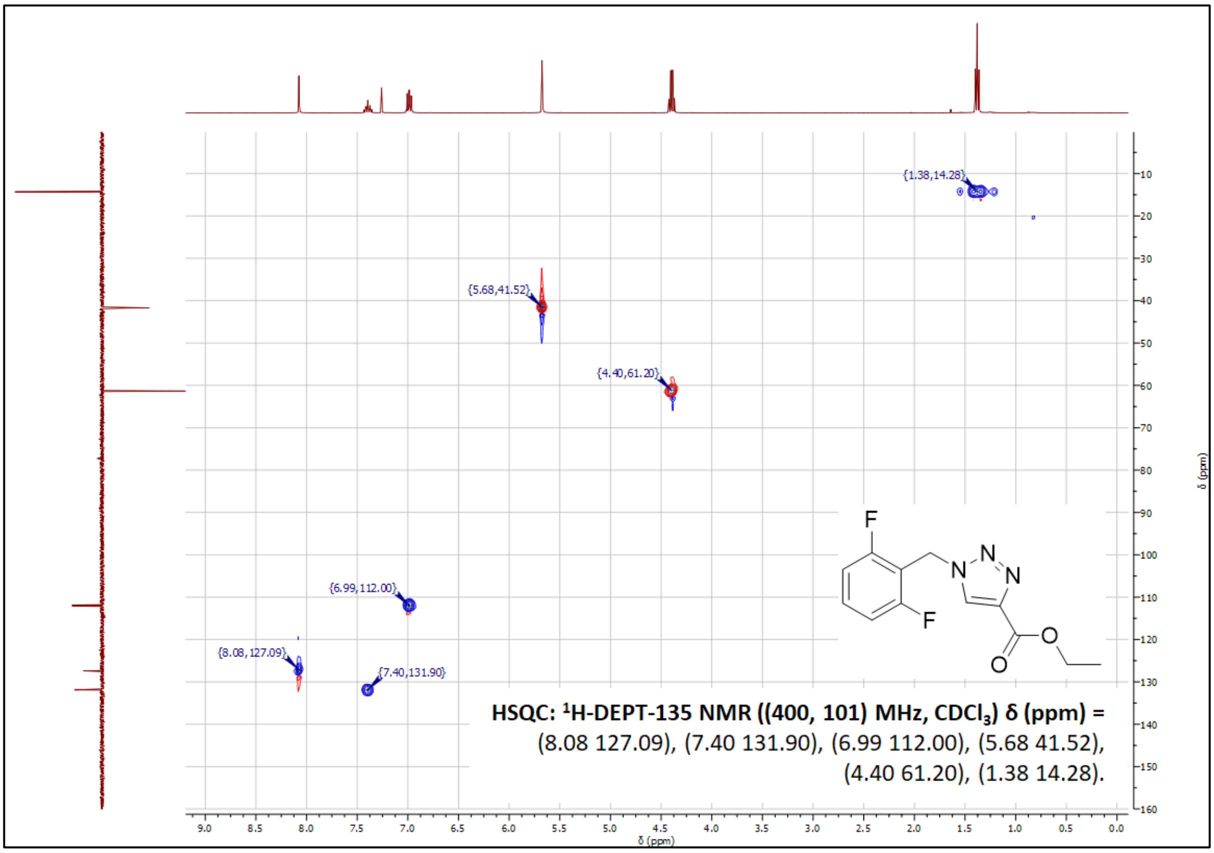

Figure S 84. *HSQC (¹H-DEPT-135) NMR spectrum of 1-(2,6-difluorobenzyl)-1H-1,2,3-triazole-4-carboxylic acid ethyl ester* ***3z****.*

¹H NMR (400 MHz, DMSO-d$_6$) δ (ppm) = 8.83 (br s, 1H), 7.52 (tt, $^3J_{H-H}$=8.5, $^4J_{H-F}$=6.7 Hz, 1H), 7.19 (m, 2H), 5.74 (t, $^4J_{H-F}$=1.15 Hz, 2H), 4.30 (q, $^3J_{H-H}$=7.1 Hz. 2H), 1.29 (t, $^3J_{H-H}$=7.1 Hz, 3H).

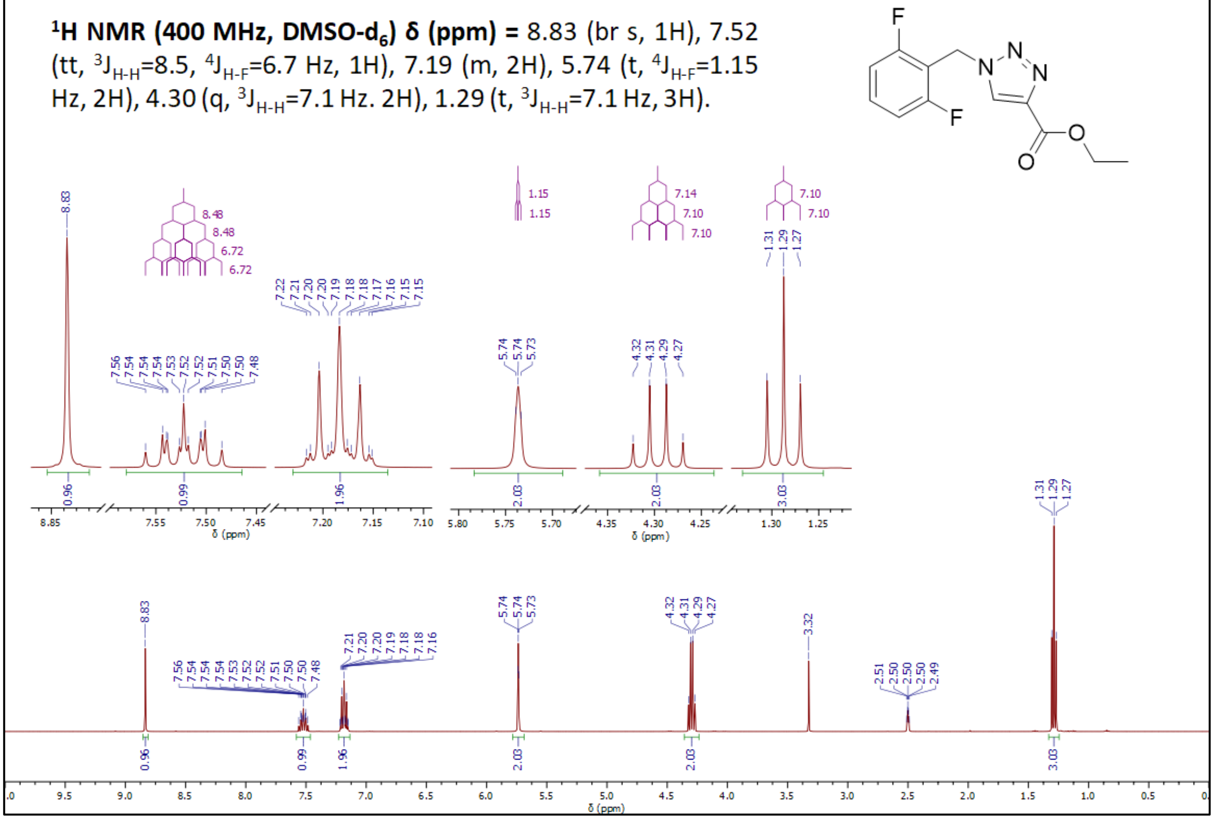

*Figure S 85. ¹H NMR spectrum of 1-(2,6-difluorobenzyl)-1H-1,2,3-triazole-4-carboxylic acid ethyl ester **3z**.*

¹³C NMR (101 MHz, DMSO-d$_6$) δ (ppm) = 160.8 (dd, $^1J_{C-F}$=249.5, $^3J_{C-F}$=7.3 Hz, C), 160.1 (s, C), 138.7 (s, C), 131.9 (t, $^3J_{C-F}$=10.5 Hz, CH), 129.4 (s, CH), 112.0 (dd, $^2J_{C-F}$=18.9, $^4J_{C-F}$=5.8 Hz, CH), 110.8 (t, $^2J_{C-F}$=19.0 Hz, C), 60.6 (s, CH_2), 41.3 (t, $^3J_{C-F}$=3.8 Hz, CH_2), 14.1 (s, CH_3).

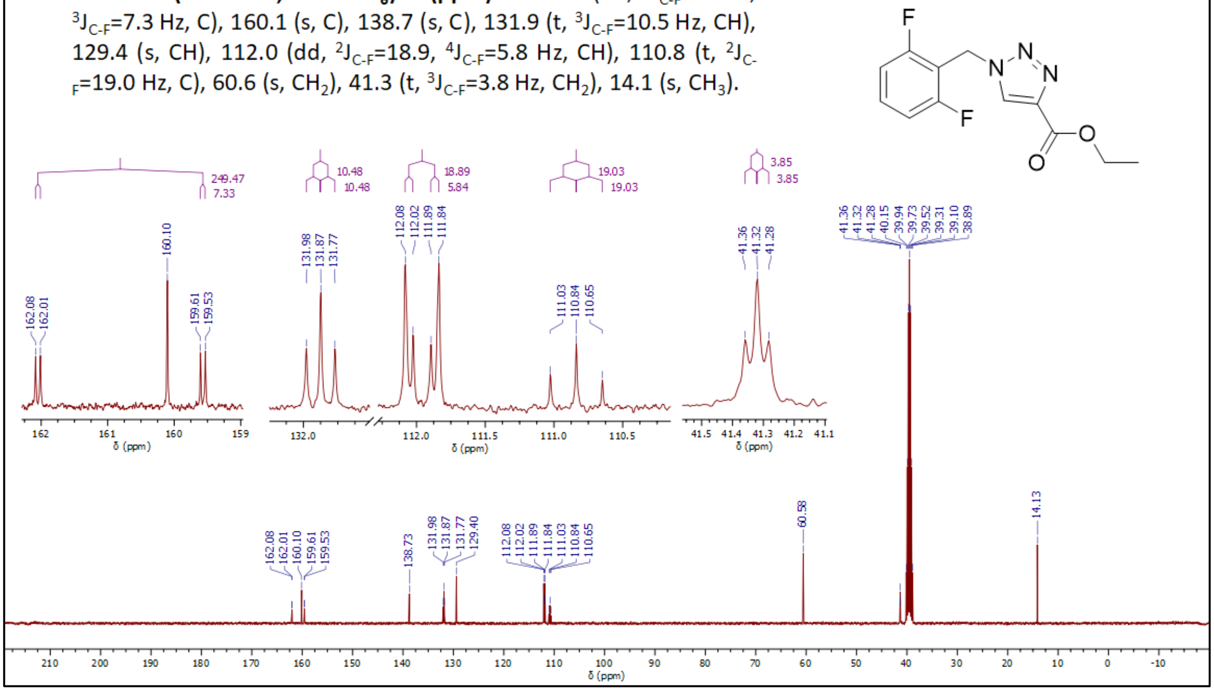

*Figure S 86. ¹³C NMR spectrum of 1-(2,6-difluorobenzyl)-1H-1,2,3-triazole-4-carboxylic acid ethyl ester **3z**.*

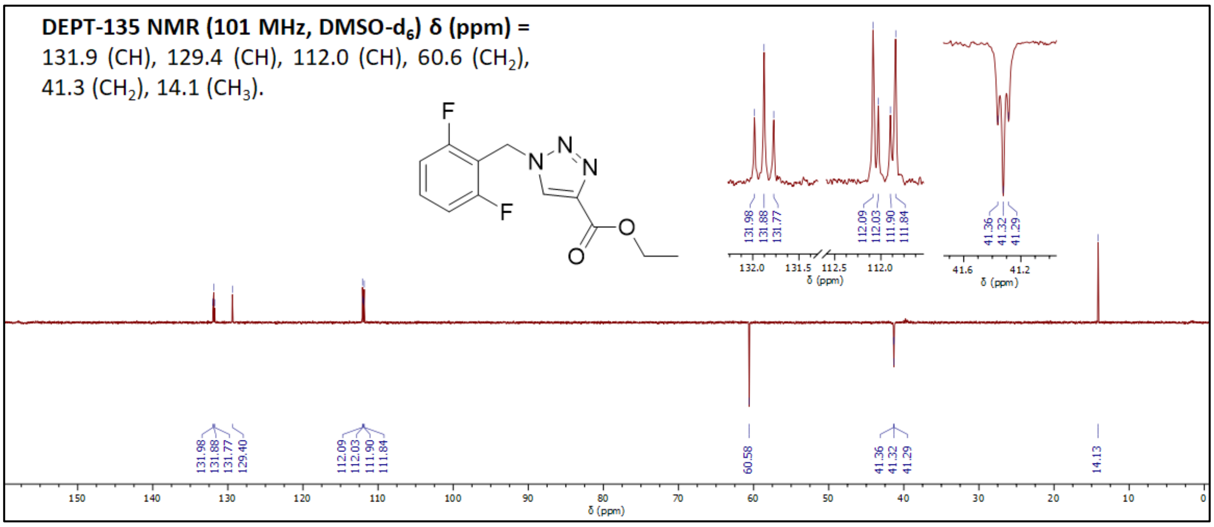

*Figure S 87. DEPT-135 NMR spectrum of 1-(2,6-difluorobenzyl)-1H-1,2,3-triazole-4-carboxylic acid ethyl ester **3z**.*

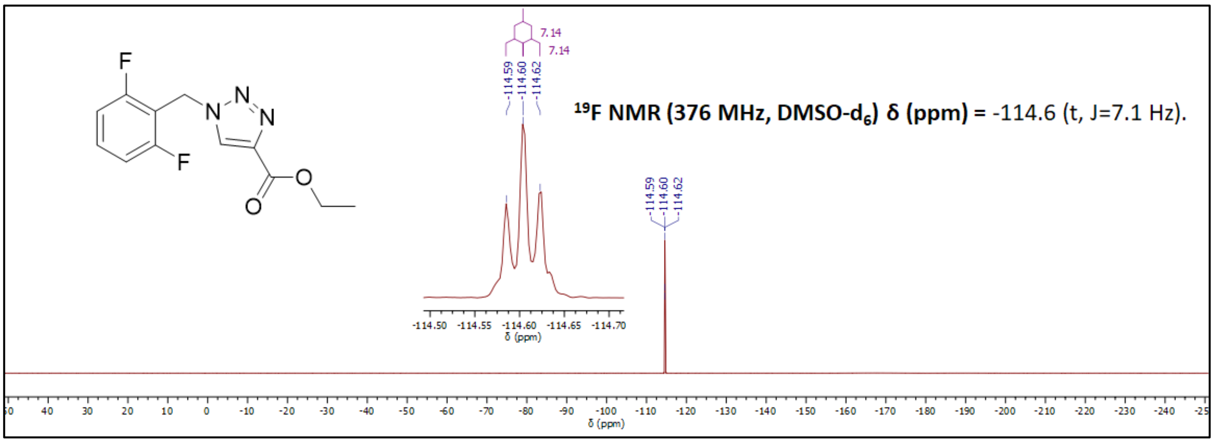

*Figure S 88. ^{19}F NMR spectrum of 1-(2,6-difluorobenzyl)-1H-1,2,3-triazole-4-carboxylic acid ethyl ester **3z**.*

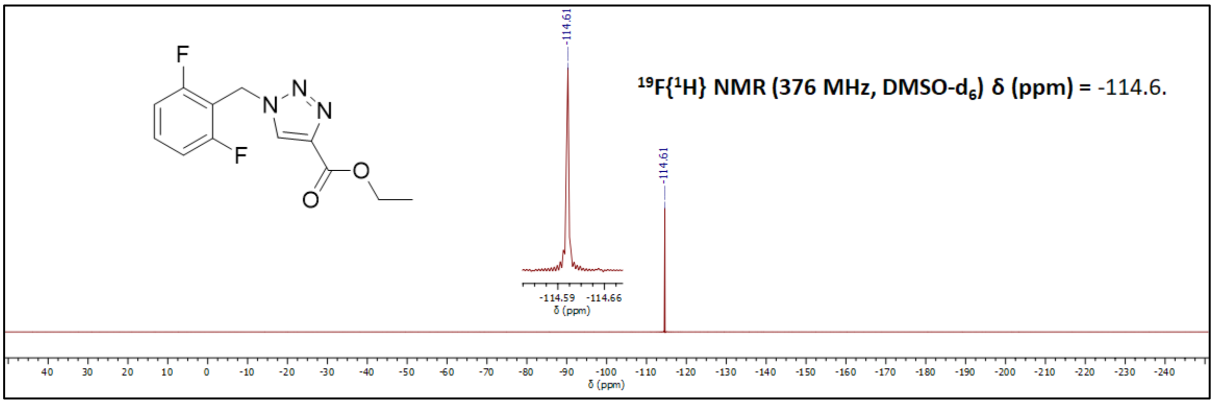

*Figure S 89. ^{19}F{^{1}H} NMR spectrum of 1-(2,6-difluorobenzyl)-1H-1,2,3-triazole-4-carboxylic acid ethyl ester **3z**.*

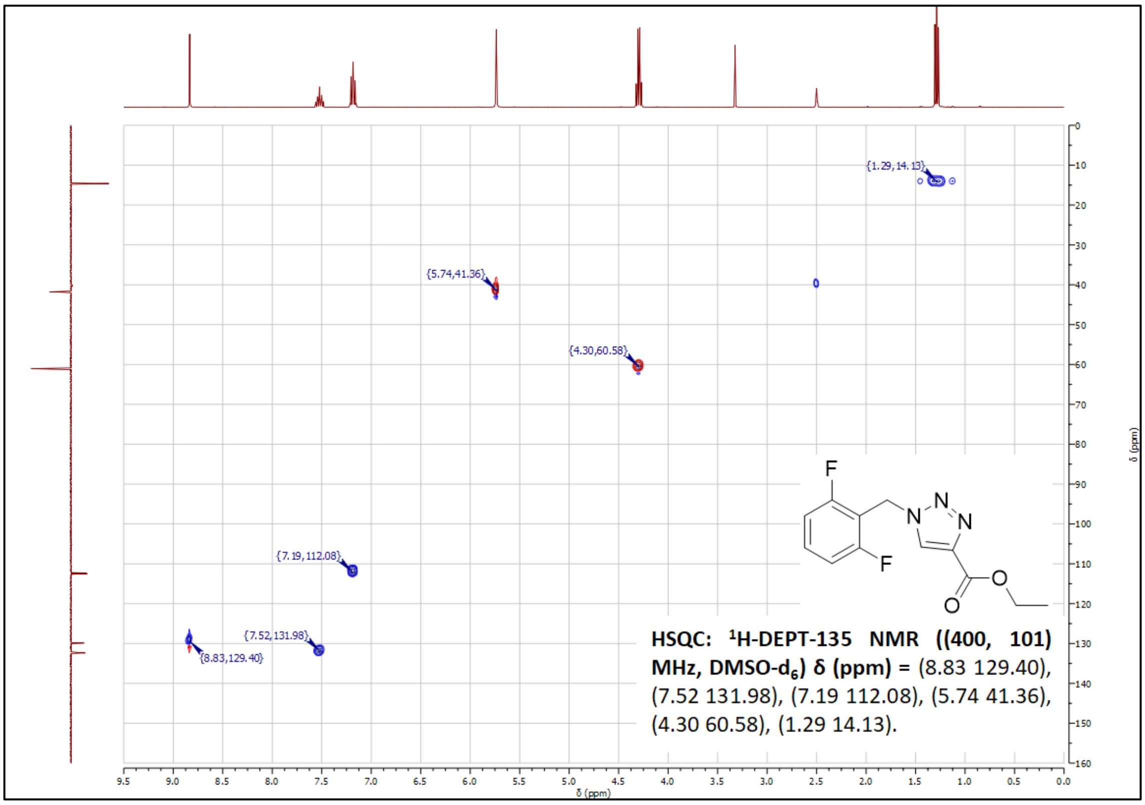

*Figure S 90. HSQC (¹H-DEPT-135) NMR spectrum of 1-(2,6-difluorobenzyl)-1H-1,2,3-triazole-4-carboxylic acid ethyl ester **3z**.*

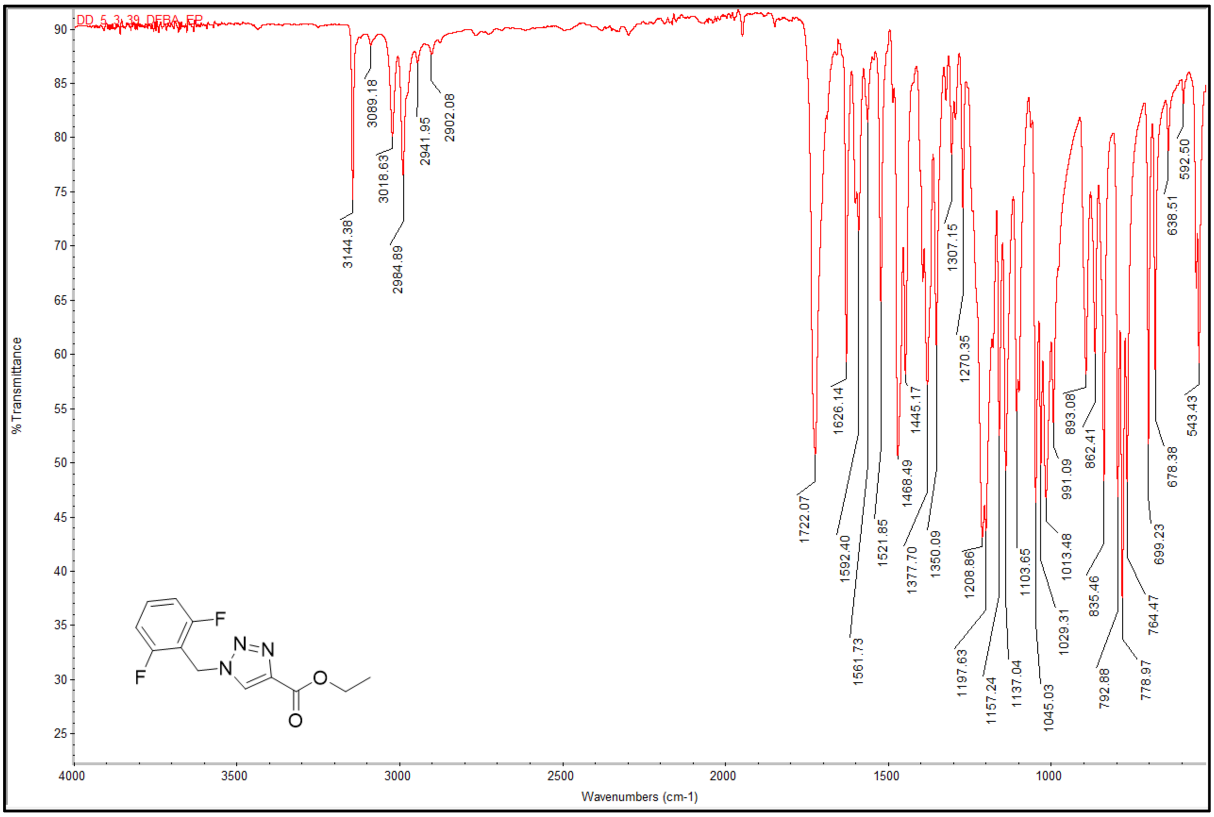

*Figure S 91. FT-ATR-IR spectrum of 1-(2,6-difluorobenzyl)-1H-1,2,3-triazole-4-carboxylic acid ethyl ester **3z**.*

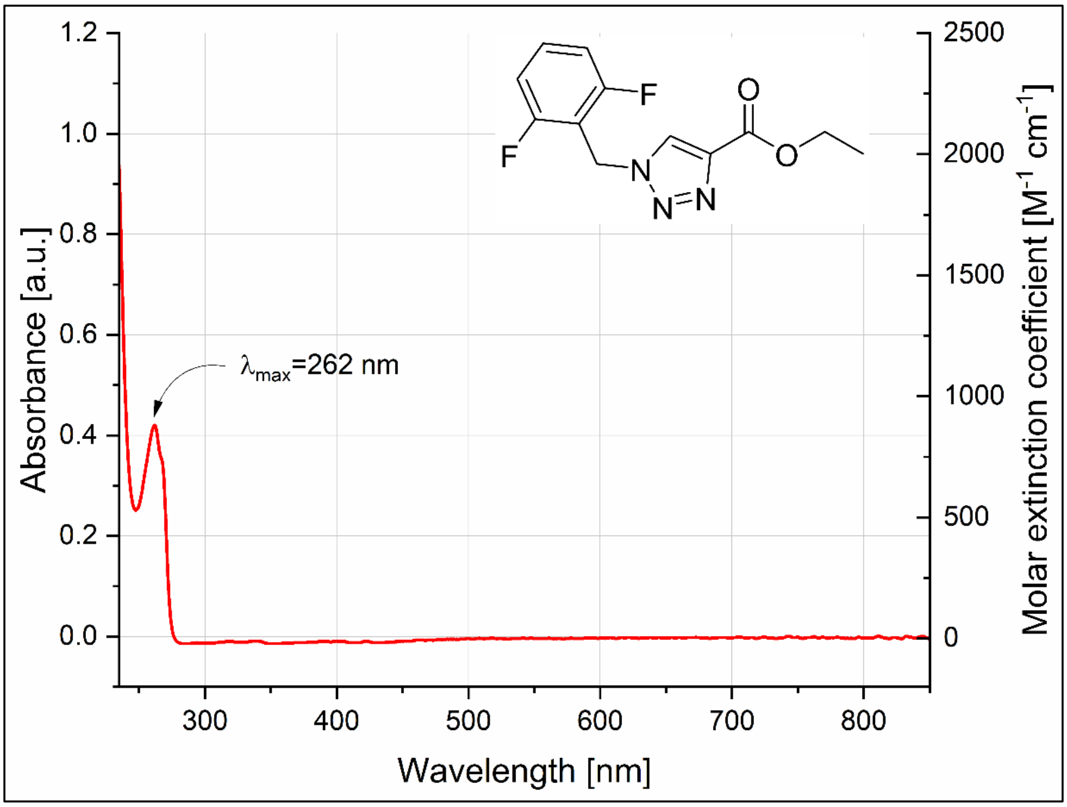

*Figure S 92. UV-Vis spectrum of 4.83E-4 M 1-(2,6-difluorobenzyl)-1H-1,2,3-triazole-4-carboxylic acid ethyl ester **3z** in DCM.*

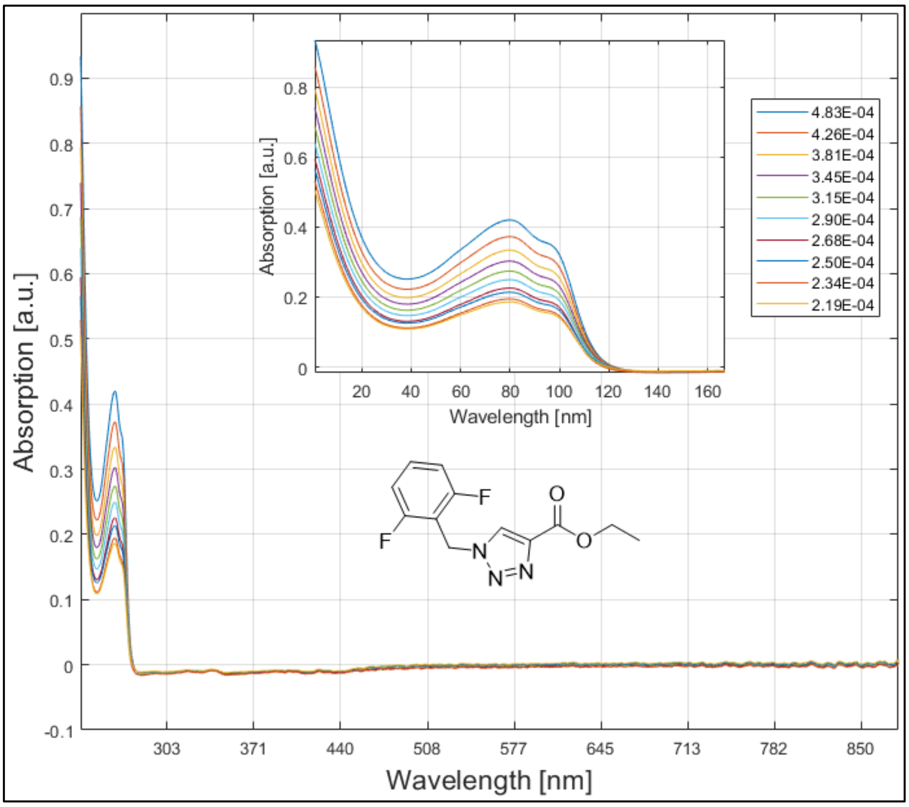

*Figure S 93. UV-Vis spectra of 1-(2,6-difluorobenzyl)-1H-1,2,3-triazole-4-carboxylic acid ethyl ester **3z** in DCM. Concentrations of measured samples are described in the legend and are in mol·dm^{-3}.*

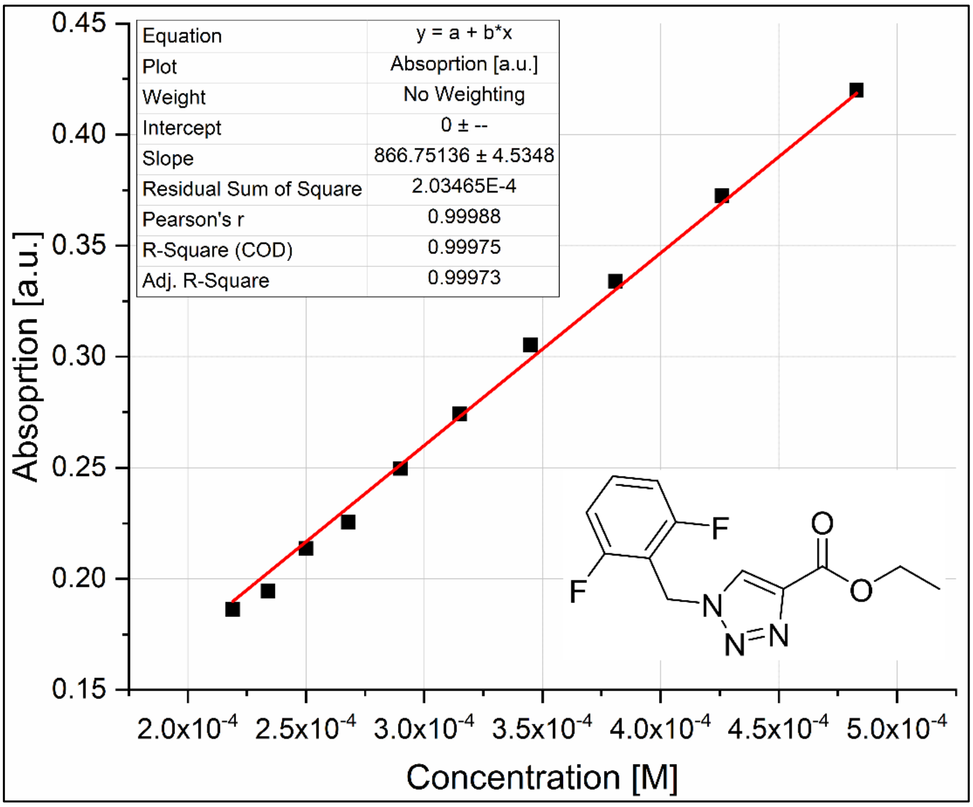

*Figure S 94. Absorption vs concentration graph of 1-(2,6-difluorobenzyl)-1H-1,2,3-triazole-4-carboxylic acid ethyl ester **3z** for determination of molar extinction coefficient using Beer-Lambert law.*

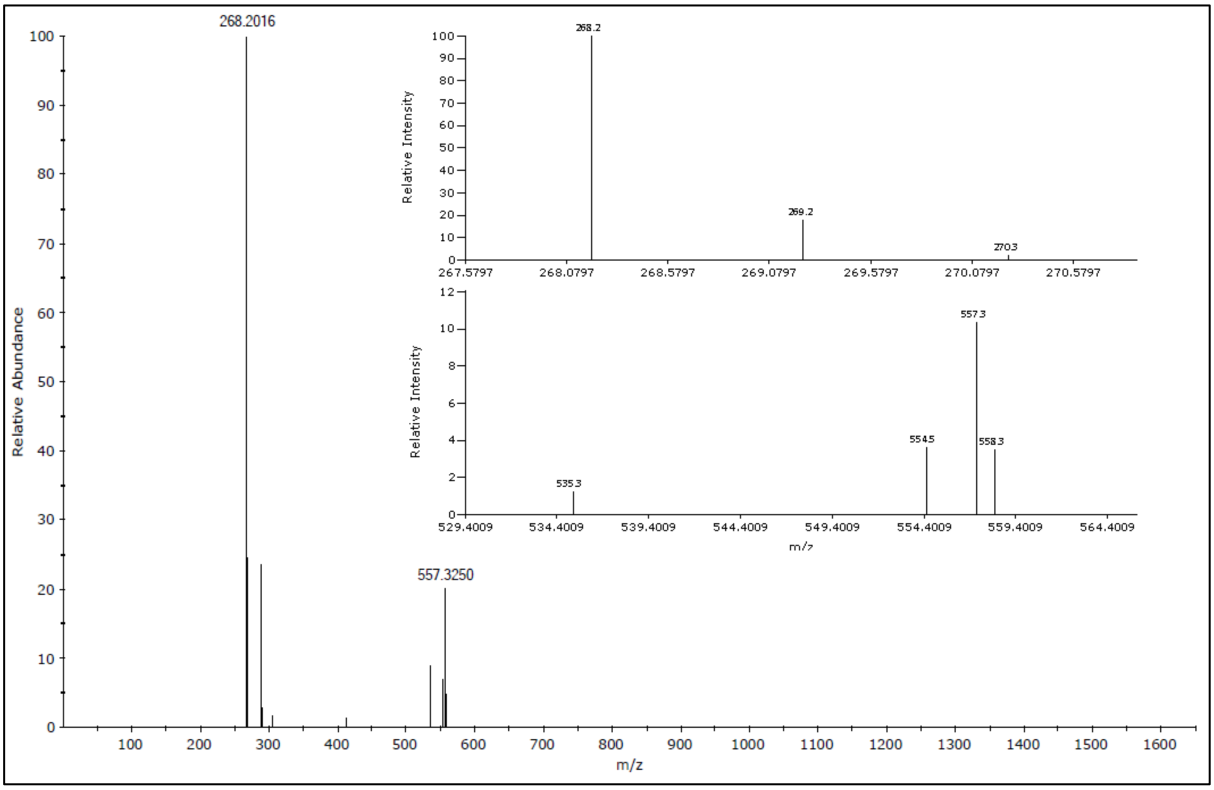

*Figure S 95. LR-ESI-MS spectrogram of 1-(2,6-difluorobenzyl)-1H-1,2,3-triazole-4-carboxylic acid ethyl ester **3z**.*

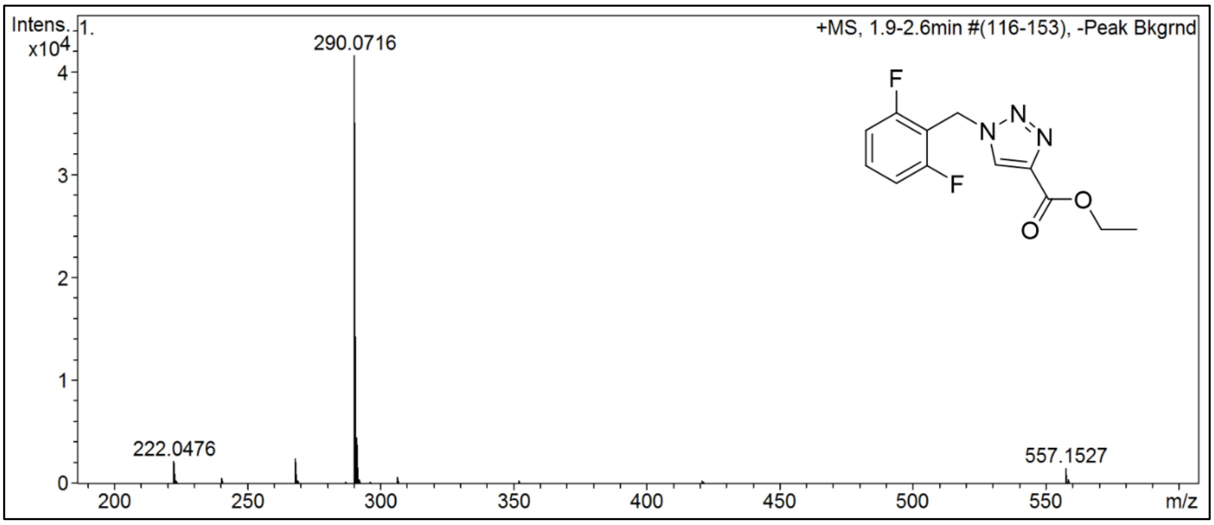

*Figure S 96. HR-ESI-Ms spectrogram of 1-(2,6-difluorobenzyl)-1H-1,2,3-triazole-4-carboxylic acid ethyl ester **3z**.*

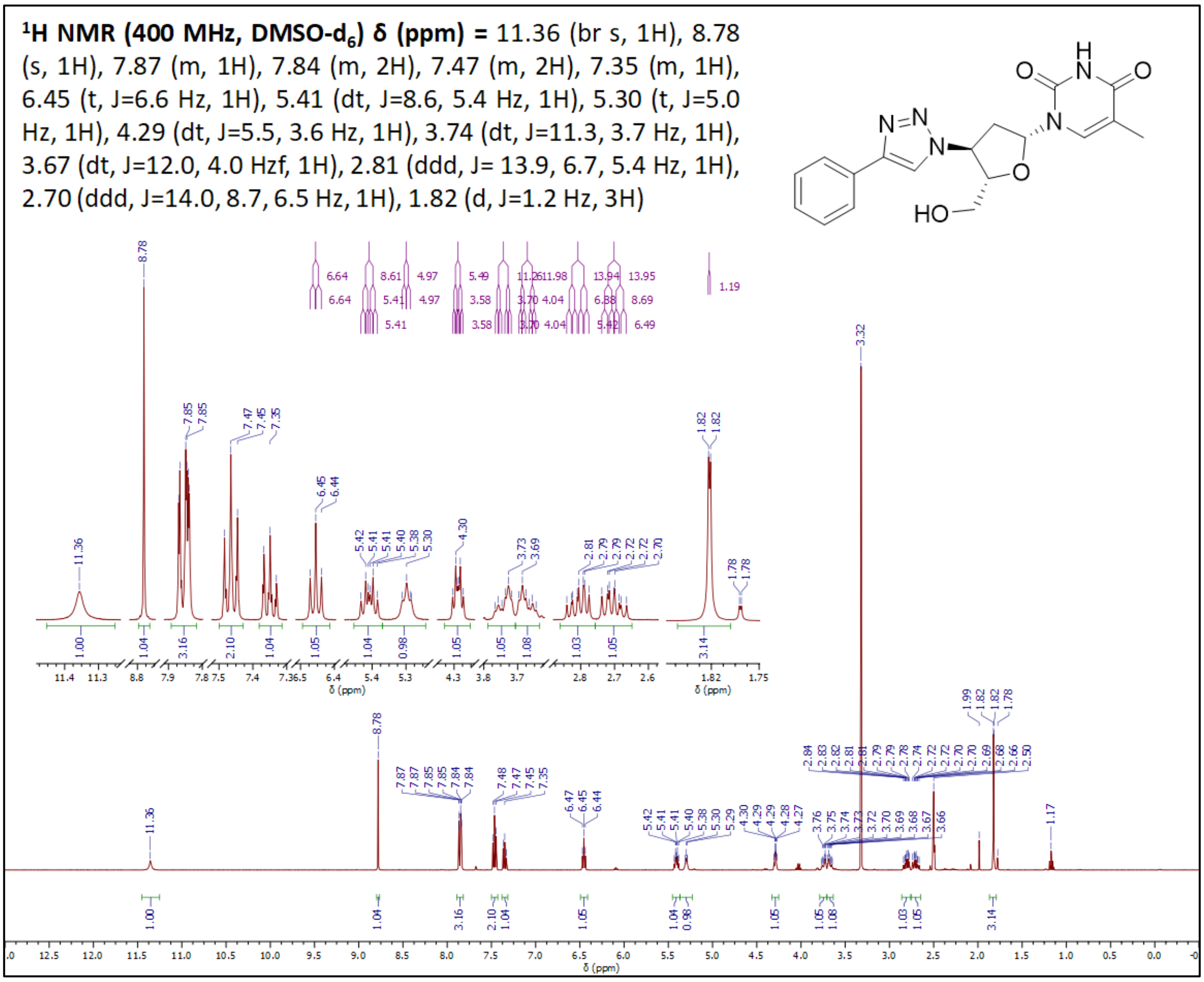

*Figure S 97. ^{1}H NMR spectrum of 3´-Deoxy-3´-(4-phenyl-1H-1,2,3-triazol-1-yl)thymidine, 1-((2R,4S,5S)-5-(hydroxymethyl)-4-(4-phenyl-1H-1,2,3-triazol-1-yl)tetrahydrofuran-2-yl)-5-methylpyrimidine-2,4(1H,3H)-dione **3aa.***

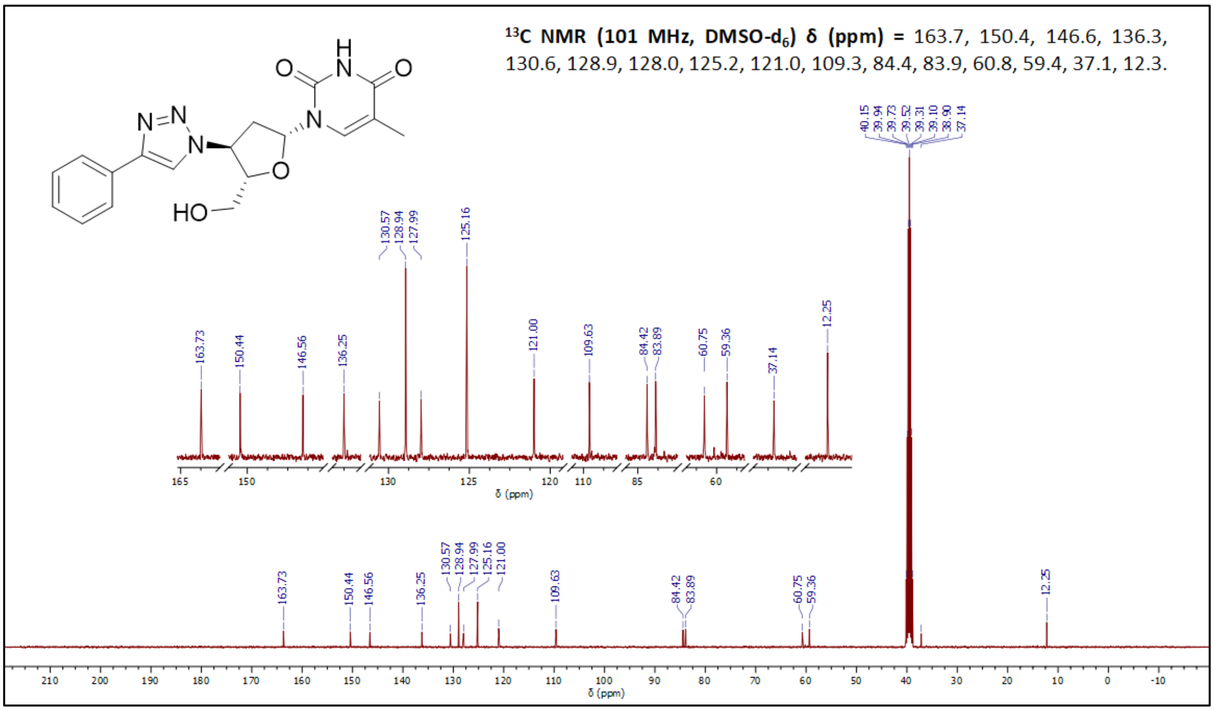

Figure S 98. ^{13}C NMR spectrum of 3´-Deoxy-3´-(4-phenyl-1H-1,2,3-triazol-1-yl)thymidine, 1-((2R,4S,5S)-5-(hydroxymethyl)-4-(4-phenyl-1H-1,2,3-triazol-1-yl)tetrahydrofuran-2-yl)-5-methylpyrimidine-2,4(1H,3H)-dione ***3aa.***

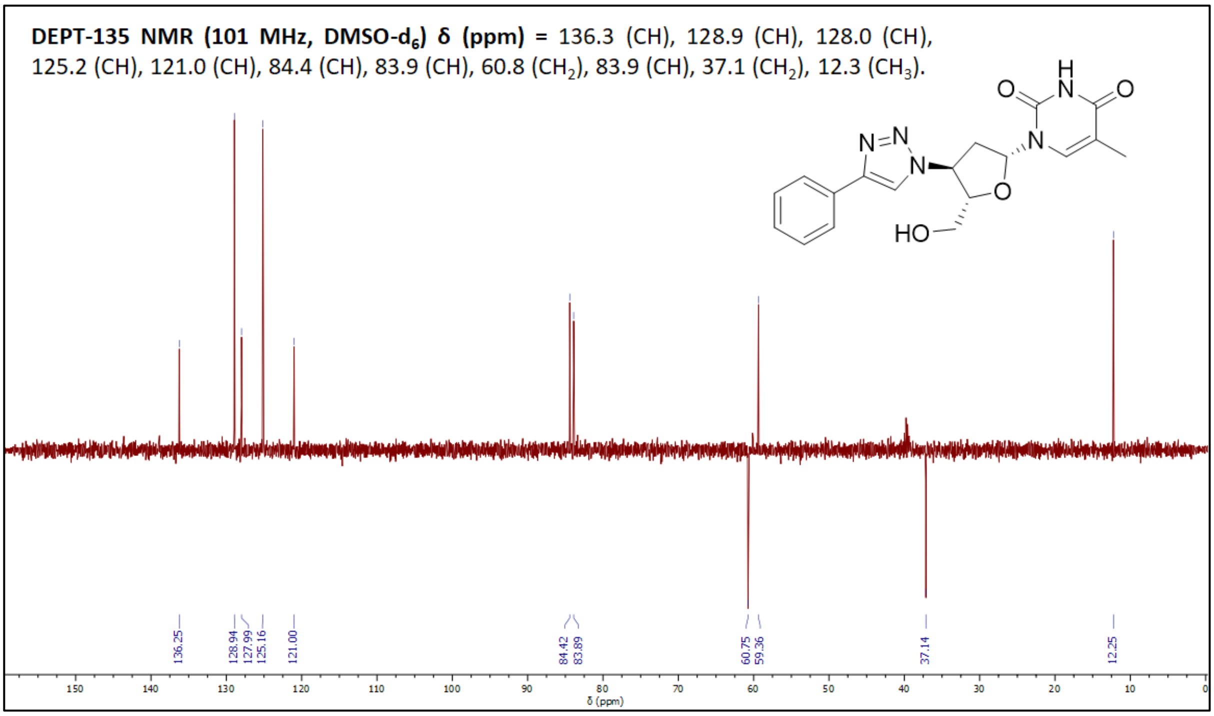

Figure S 99. DEPT-135 NMR spectrum of 3´-Deoxy-3´-(4-phenyl-1H-1,2,3-triazol-1-yl)thymidine, 1-((2R,4S,5S)-5-(hydroxymethyl)-4-(4-phenyl-1H-1,2,3-triazol-1-yl)tetrahydrofuran-2-yl)-5-methylpyrimidine-2,4(1H,3H)-dione ***3aa.***

^{1}H-^{13}C NMR ((400, 101) MHz, DMSO-d$_6$) δ (ppm) = (8.78 121.00), (7.86 125.16), (7.84 136.25), (7.47 128.94), (7.35 127.99), (6.45 83.89), (5.41 59.36), (4.29 84.42), (3.74 60.75), (3.67 60.75), (2.81 37.14), (2.70 37.14), (1.82 12.25).

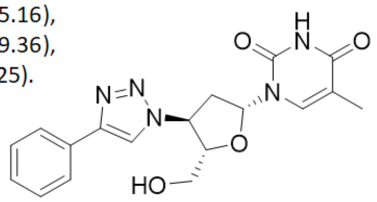

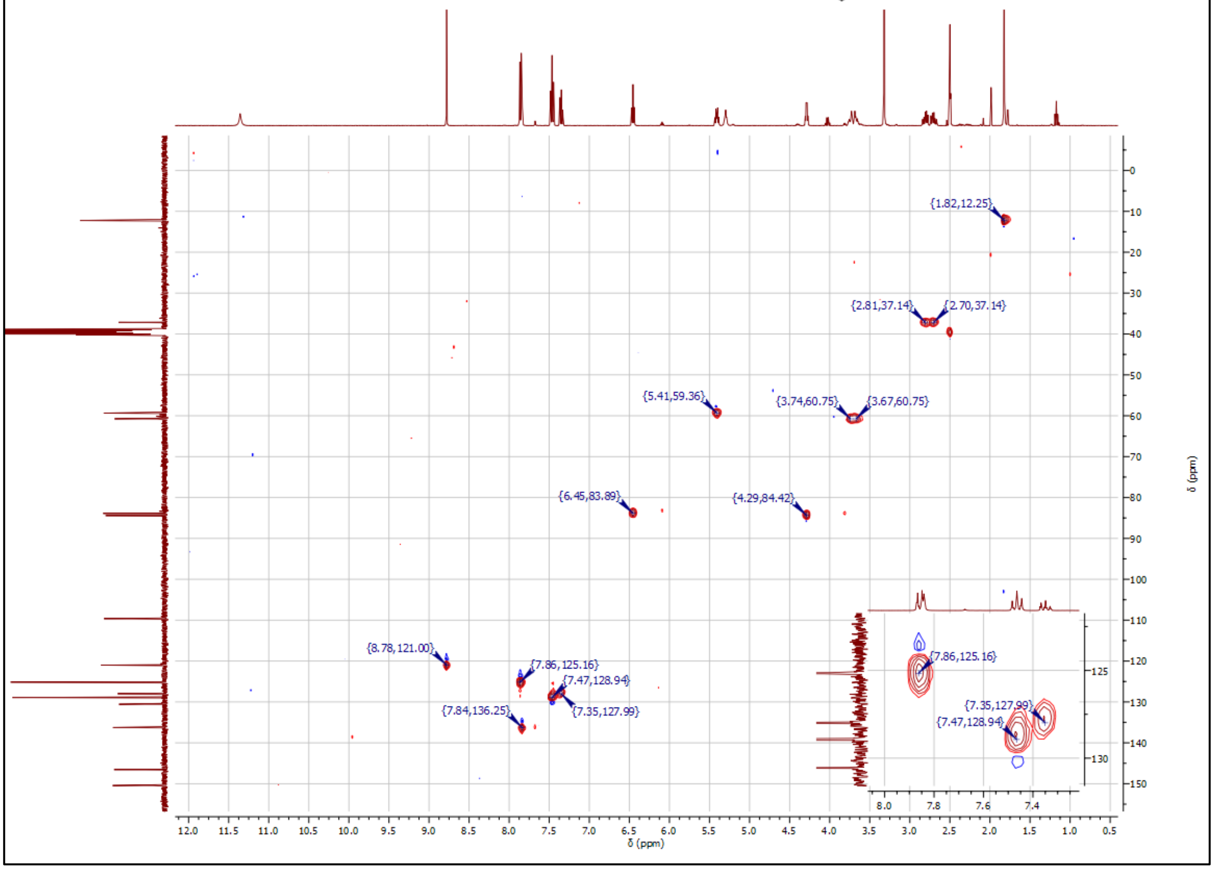

Figure S 100. HSQC (^{1}H-^{13}C) NMR spectrum of 3′-Deoxy-3′-(4-phenyl-1H-1,2,3-triazol-1-yl)thymidine, 1-((2R,4S,5S)-5-(hydroxymethyl)-4-(4-phenyl-1H-1,2,3-triazol-1-yl)tetrahydrofuran-2-yl)-5-methylpyrimidine-2,4(1H,3H)-dione ***3aa.***

^{1}H-^{13}C NMR ((400, 101) MHz, DMSO-d_6) δ (ppm) = (8.78 146.56), (8.78 121.00), (7.86 146.56), (7.84 12.25), (7.84 83.89), (7.84 84.42), (7.84 109.63), (7.84 150.44), (7.84 163.73), (7.47 125.16), (7.47 128.94), (7.47 130.57), (7.35 125.16), (6.45 84.42), (6.45 136.25), (6.45 150.44), (5.41 37.14), (5.41 60.75), (5.41 83.89), (5.40 121.00), (4.29 59.36), (2.81 59.36), (2.70 59.36), (2.70 83.89), (1.98 12.25), (1.82 109.63), (1.82 136.25), (1.82 163.73), (1.66 12.25), (1.17 59.36).

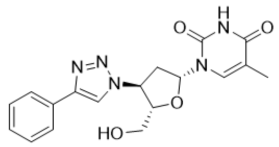

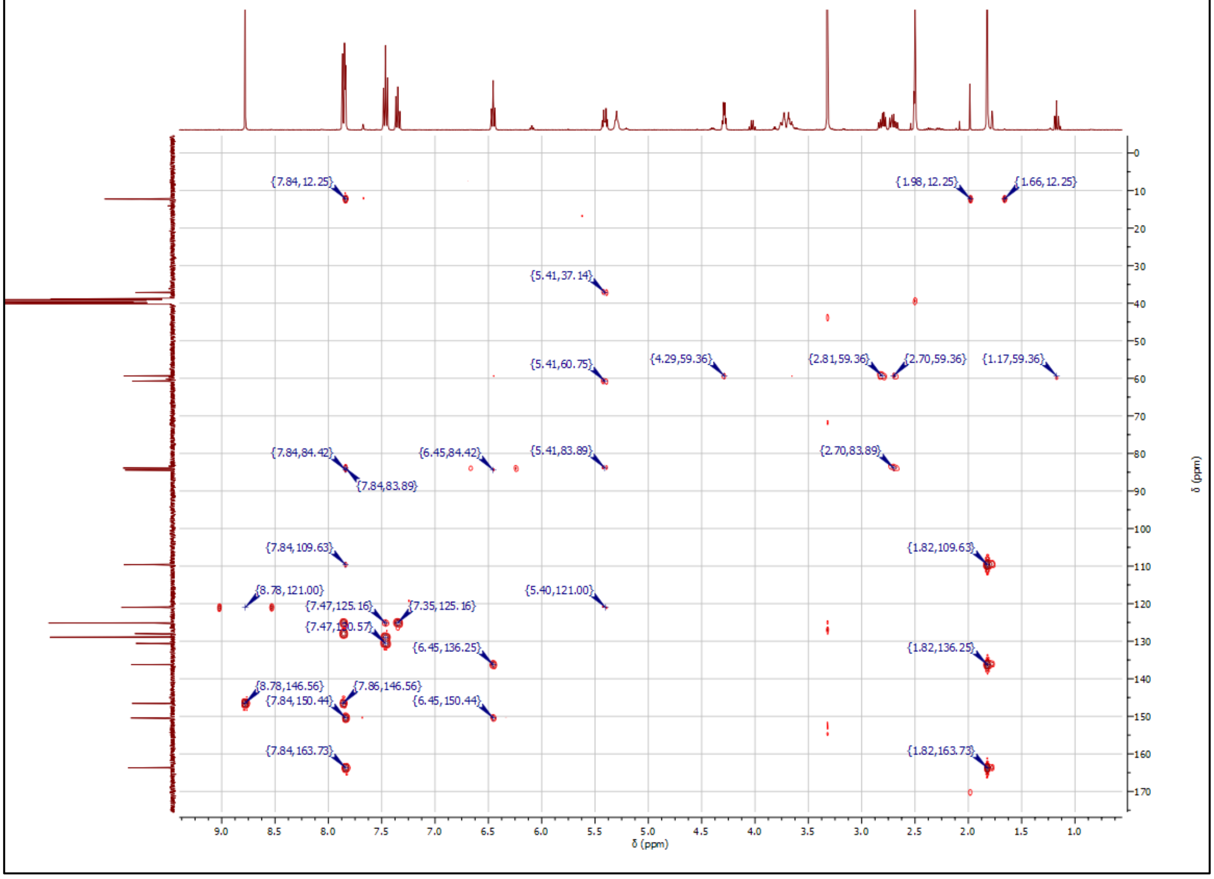

*Figure S 101. HMBC (^{1}H-^{13}C) NMR spectrum of 3′-Deoxy-3′-(4-phenyl-1H-1,2,3-triazol-1-yl)thymidine, 1-((2R,4S,5S)-5-(hydroxymethyl)-4-(4-phenyl-1H-1,2,3-triazol-1-yl)tetrahydrofuran-2-yl)-5-methylpyrimidine-2,4(1H,3H)-dione **3aa.***

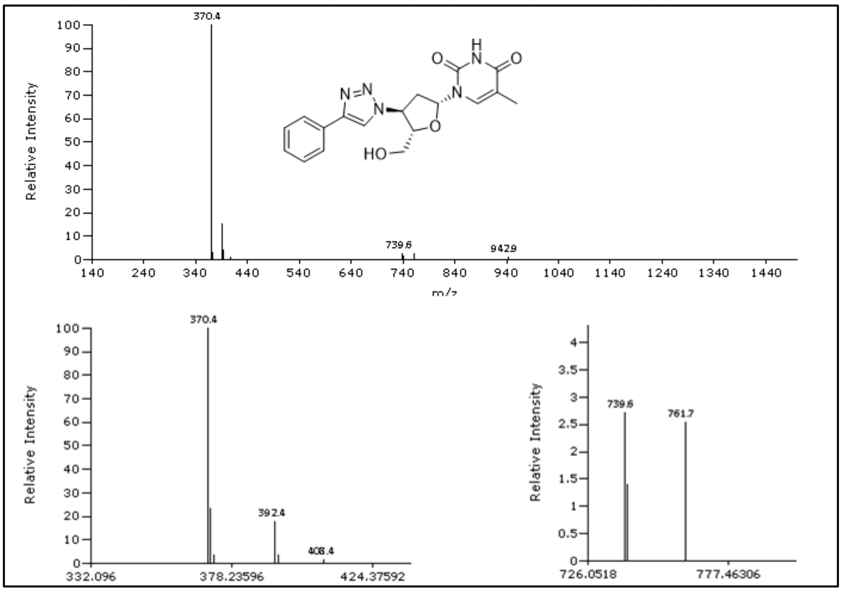

*Figure S 102. LR-ESI-MS of 3′-Deoxy-3′-(4-phenyl-1H-1,2,3-triazol-1-yl)thymidine, 1-((2R,4S,5S)-5-(hydroxymethyl)-4-(4-phenyl-1H-1,2,3-triazol-1-yl)tetrahydrofuran-2-yl)-5-methylpyrimidine-2,4(1H,3H)-dione **3aa.***

^{1}H NMR (400 MHz, CDCl$_3$) δ (ppm) = 7.79 (s, 2H), 7.72 (m, 4H), 7.51 (m, 4H), 7.42 (m, 2H), 2.93 (t, J=7.4 Hz, 4H), 2.22 (q, J=7.4 Hz, 2H).

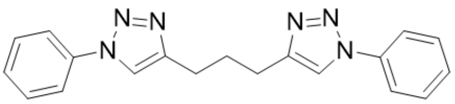

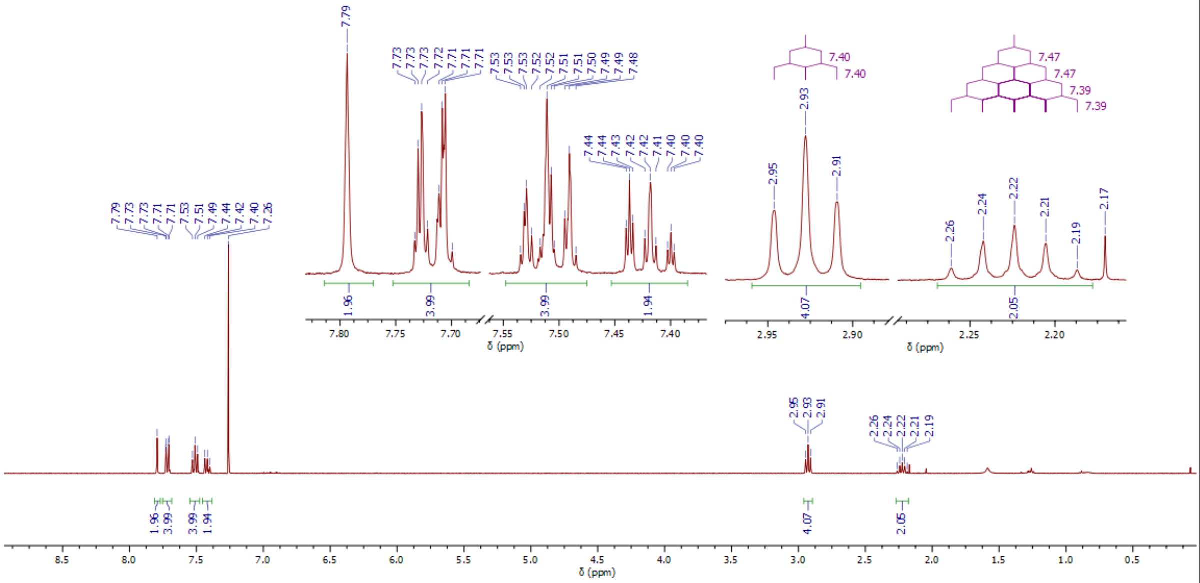

*Figure S 103. ^{1}H NMR spectrum of 1,3-bis(1-phenyl-1H-1,2,3-triazol-4-yl)propane **3ab**.*

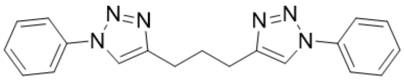

DEPT-135 NMR (101 MHz, CDCl$_3$) δ (ppm) = CH: 129.8, 128.6, 120.6, 119.4; **CH$_2$:** 29.1, 25.1.

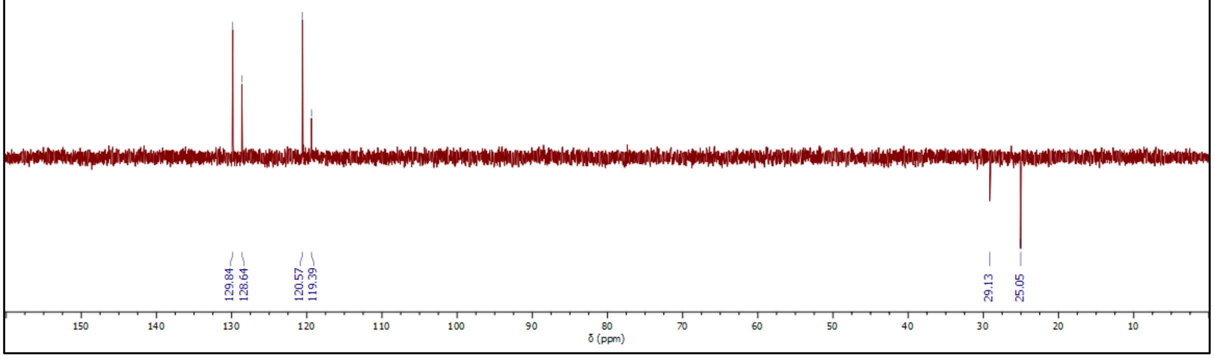

*Figure S 104. DEPT-135 NMR spectrum of 1,3-bis(1-phenyl-1H-1,2,3-triazol-4-yl)propane **3ab**.*

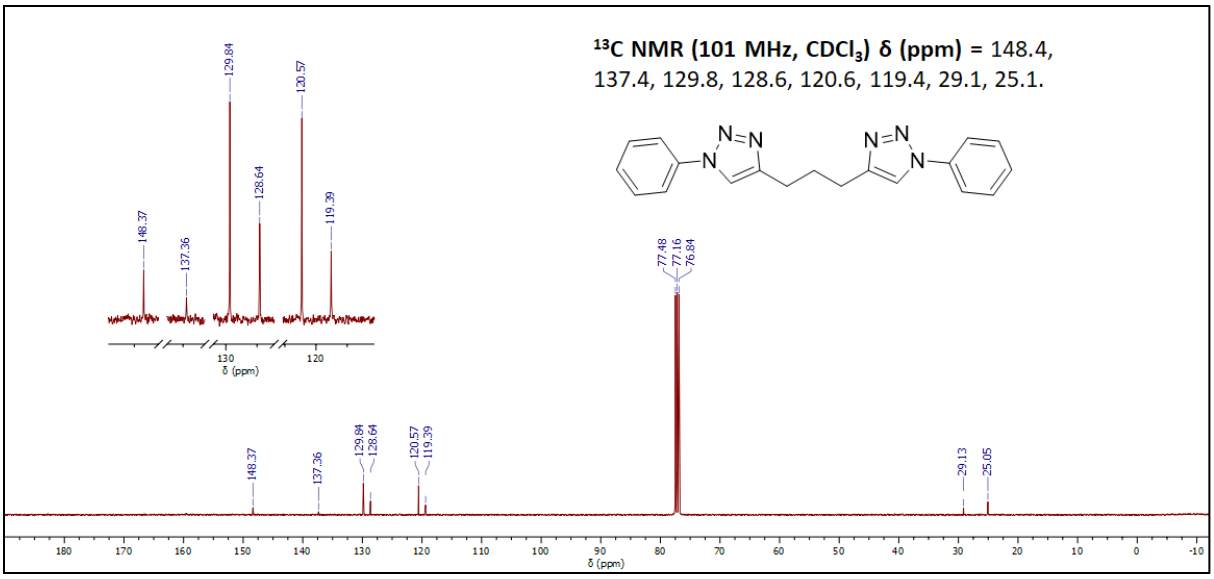

*Figure S 105. ^{13}C NMR spectrum of 1,3-bis(1-phenyl-1H-1,2,3-triazol-4-yl)propane **3ab**.*

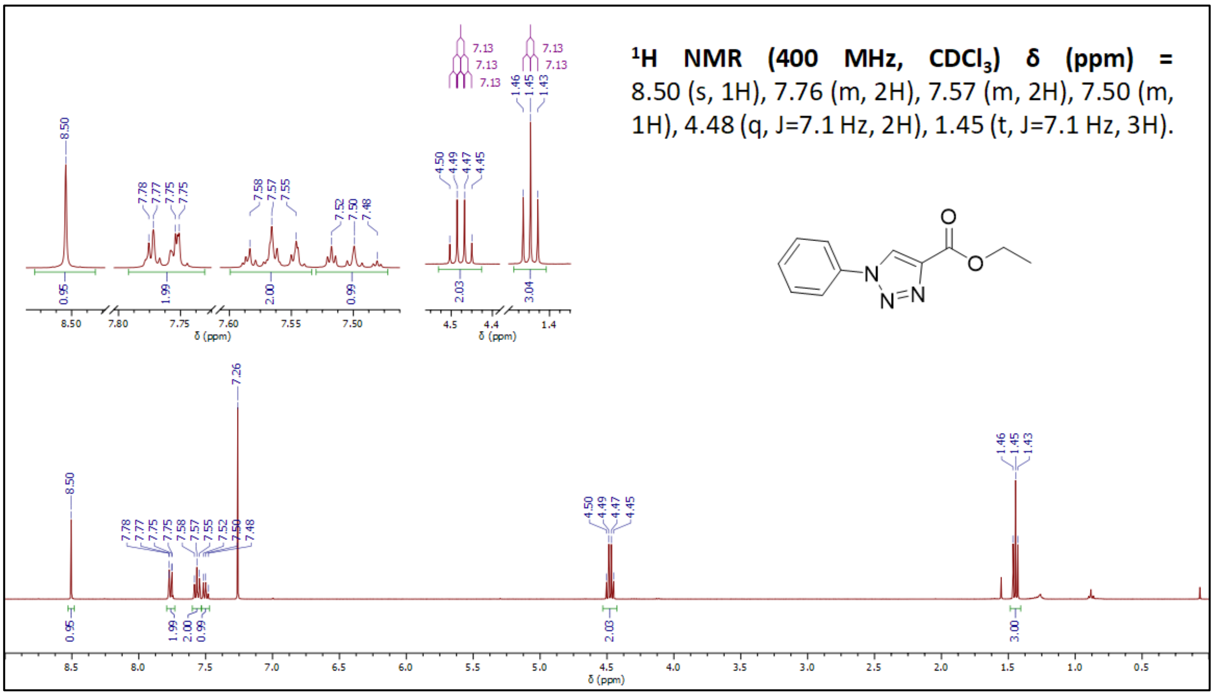

*Figure S 106. ^{1}H NMR spectrum of 1-phenyl--1H-1,2,3-triazolyl-4-carboxylic acid ethyl ester **3ac**.*

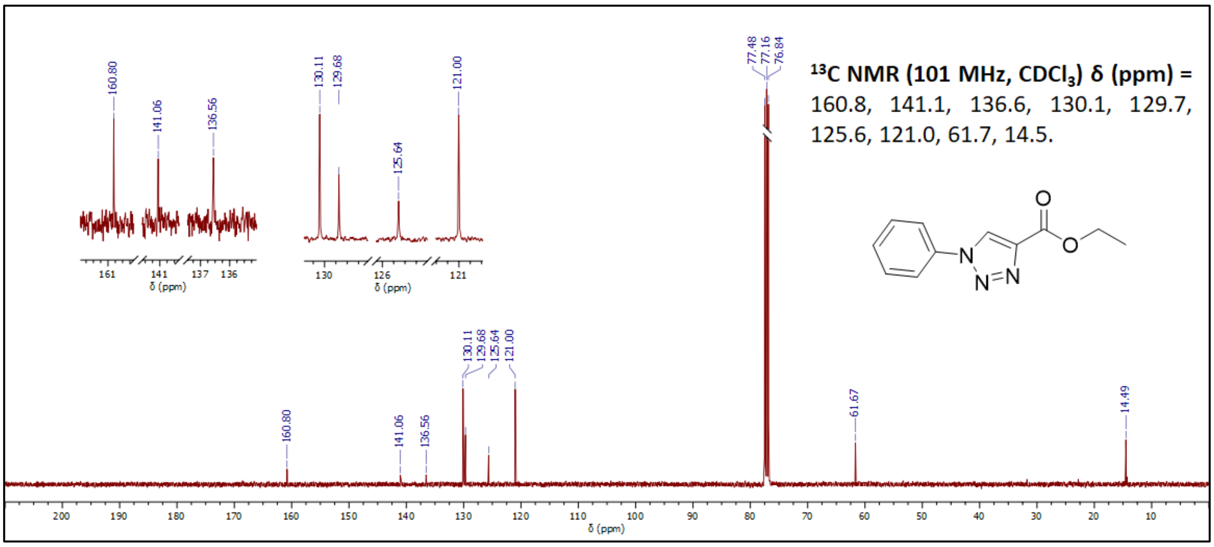

Figure S 107. ¹³C NMR spectrum of 1-phenyl--1H-1,2,3-triazolyl-4-carboxylic acid ethyl ester ***3ac****.*

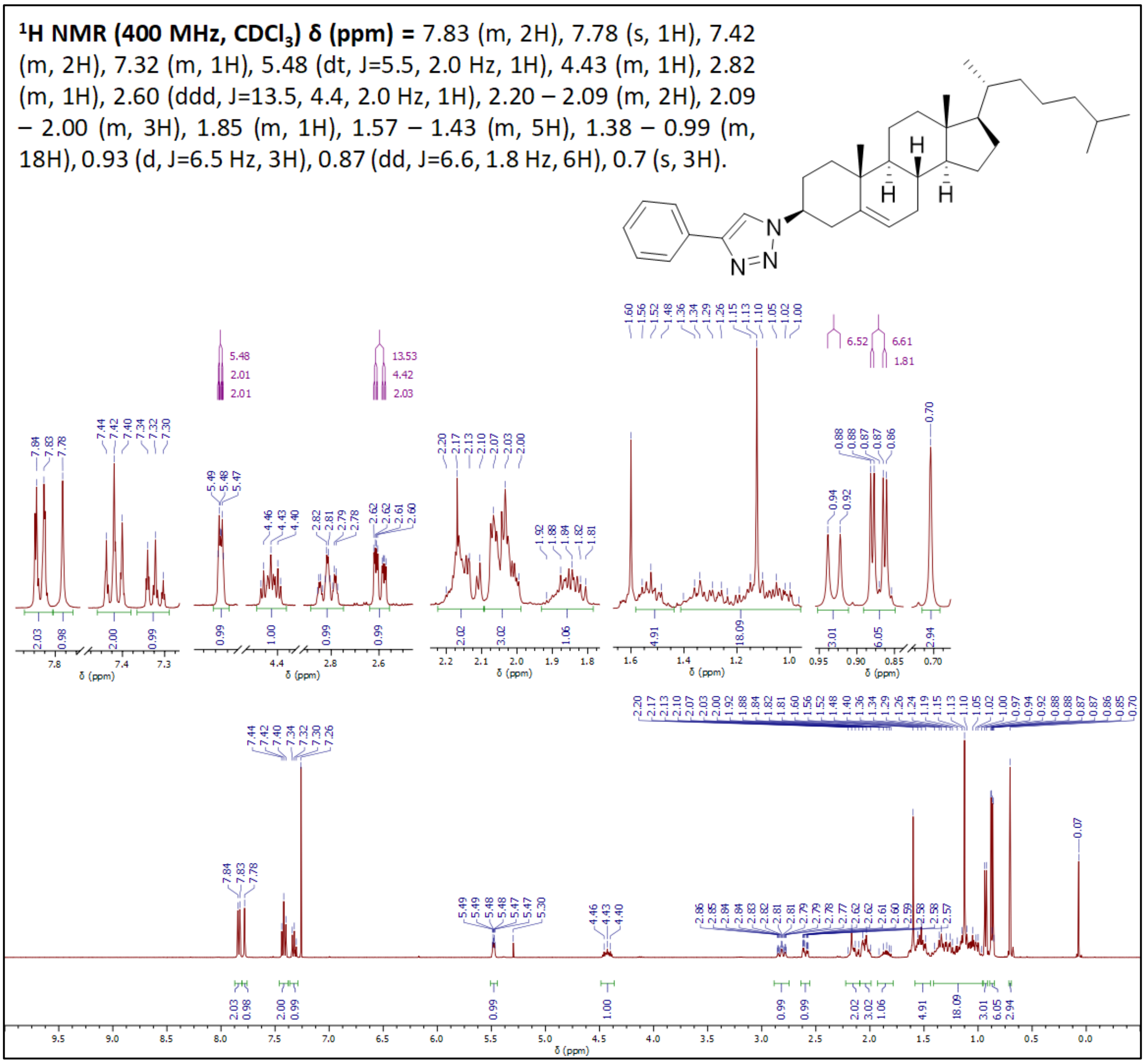

Figure S 108. ¹H NMR spectrum of 1-(3β)-Cholest-5-en-3-yl-4-phenyl-1H-1,2,3-triazole, 1-((3S,8S,9S,10R,13R,14S,17R)-10,13-dimethyl-17-((R)-6-methylheptan-2-yl)-2,3,4,7,8,9,10,11,12,13,14,15,16,17-tetradecahydro-1H-cyclopenta[a]phenanthren-3-yl)-4-phenyl-1H-1,2,3-triazole ***3ad****.*

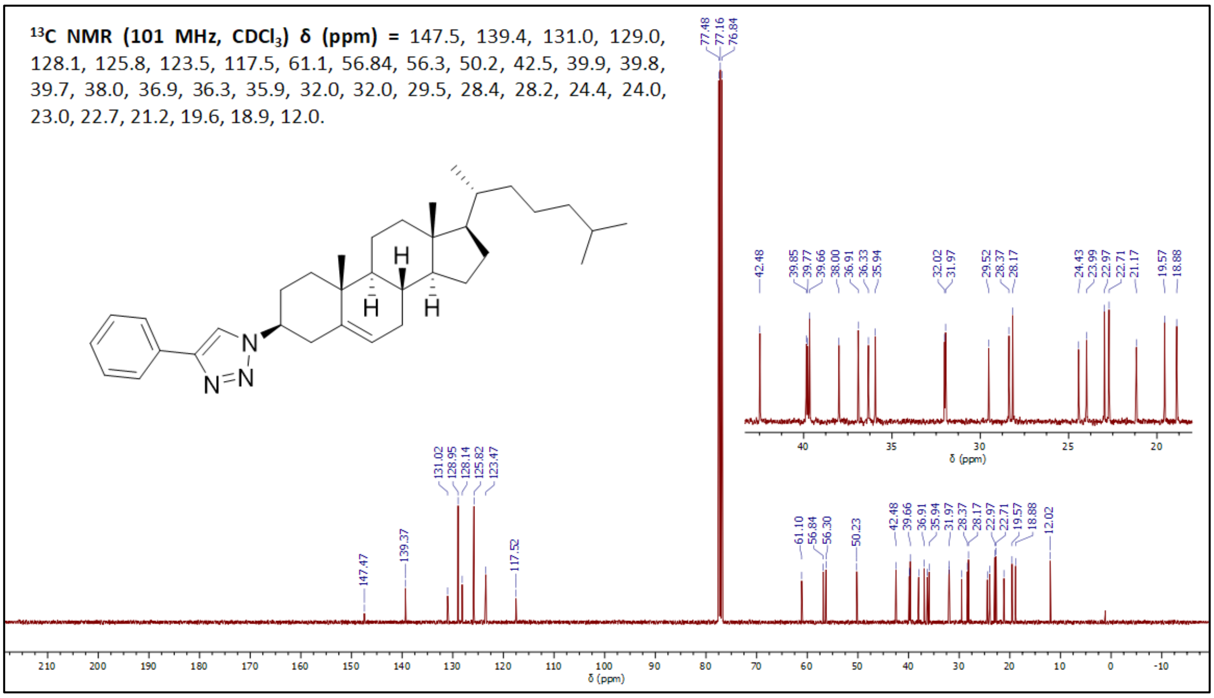

Figure S 109. ^{13}C NMR spectrum of 1-(3β)-Cholest-5-en-3-yl-4-phenyl-1H-1,2,3-triazole, 1-((3S,8S,9S,10R,13R,14S,17R)-10,13-dimethyl-17-((R)-6-methylheptan-2-yl)-2,3,4,7,8,9,10,11,12,13,14,15,16,17-tetradecahydro-1H-cyclopenta[a]phenanthren-3-yl)-4-phenyl-1H-1,2,3-triazole ***3ad.***

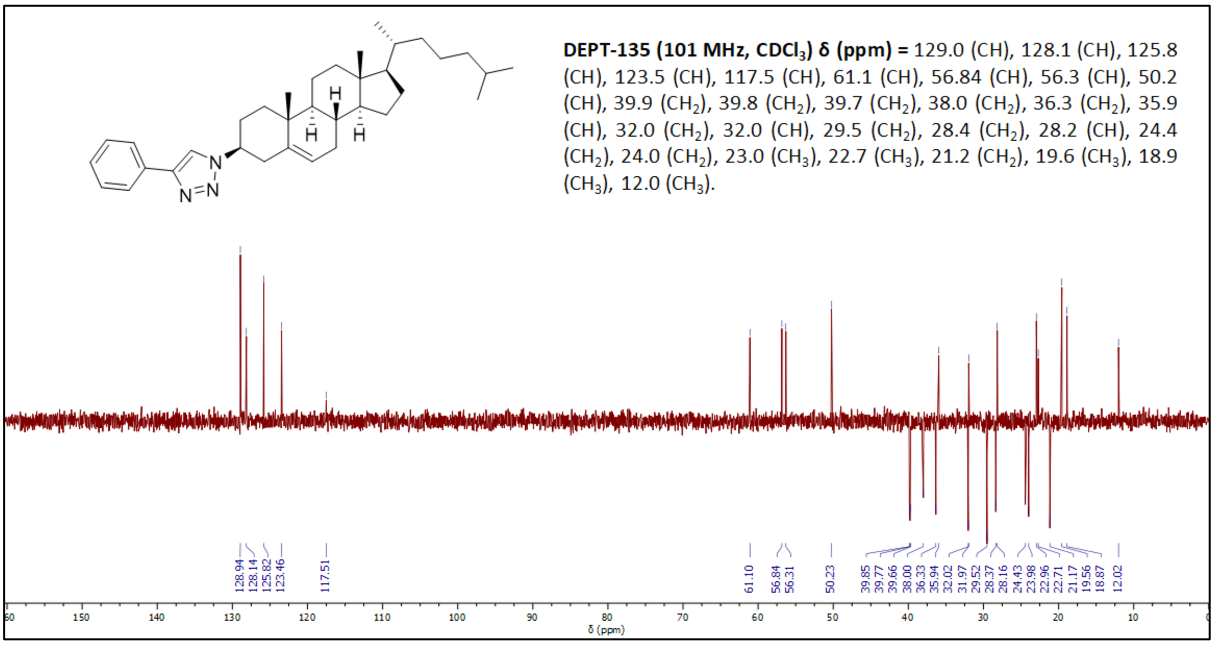

Figure S 110. DEPT-135 NMR spectrum of 1-(3β)-Cholest-5-en-3-yl-4-phenyl-1H-1,2,3-triazole, 1-((3S,8S,9S,10R,13R,14S,17R)-10,13-dimethyl-17-((R)-6-methylheptan-2-yl)-2,3,4,7,8,9,10,11,12,13,14,15,16,17-tetradecahydro-1H-cyclopenta[a]phenanthren-3-yl)-4-phenyl-1H-1,2,3-triazole ***3ad.***

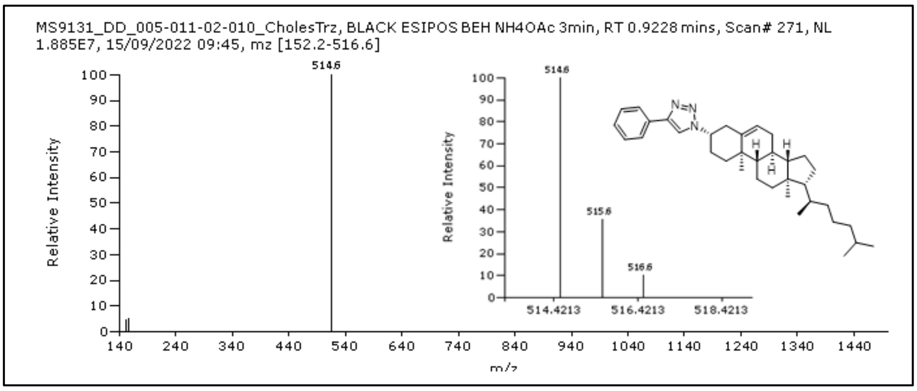

*Figure S 111. LR-ESI-MS spectrogram of 1-(3β)-Cholest-5-en-3-yl-4-phenyl-1H-1,2,3-triazole, 1-((3S,8S,9S,10R,13R,14S,17R)-10,13-dimethyl-17-((R)-6-methylheptan-2-yl)-2,3,4,7,8,9,10,11,12,13,14,15,16,17-tetradecahydro-1H-cyclopenta[a]phenanthren-3-yl)-4-phenyl-1H-1,2,3-triazole **3ad.***

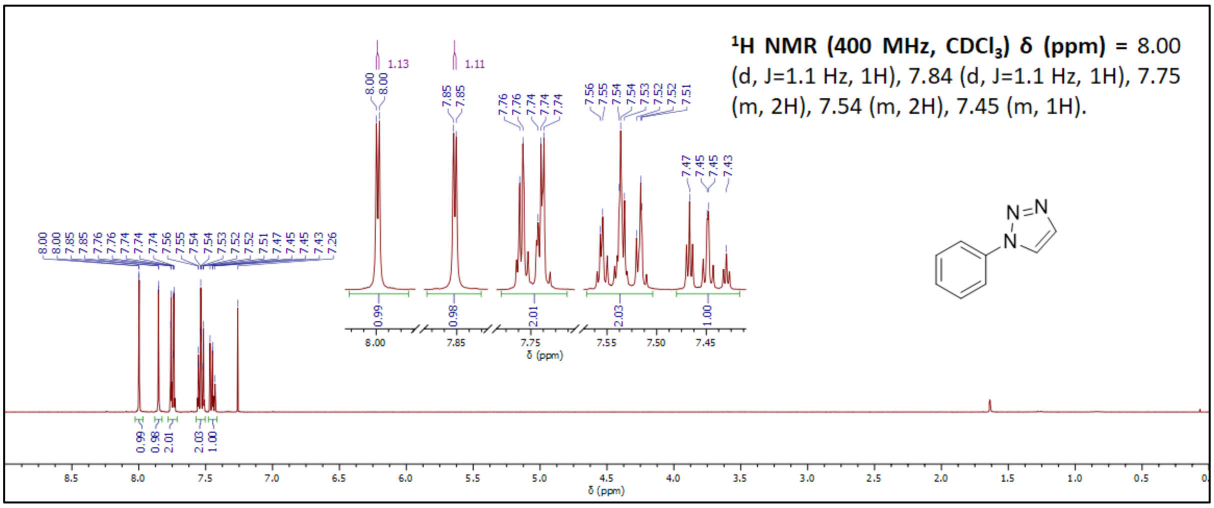

*Figure S 112. ^{1}H NMR spectrum of 1-phenyl-1H-1,2,3-triazole **3ae**.*

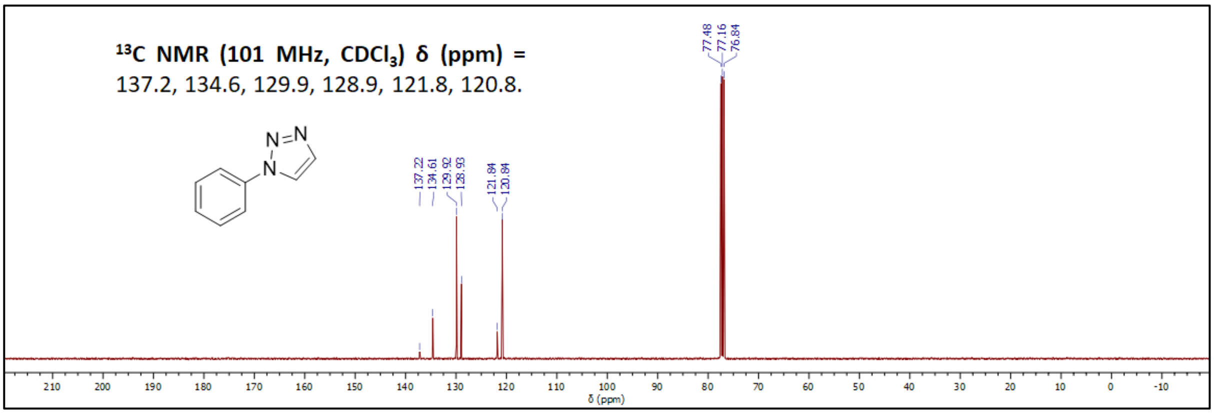

*Figure S 113. ^{13}C NMR spectrum of 1-phenyl-1H-1,2,3-triazole **3ae**.*

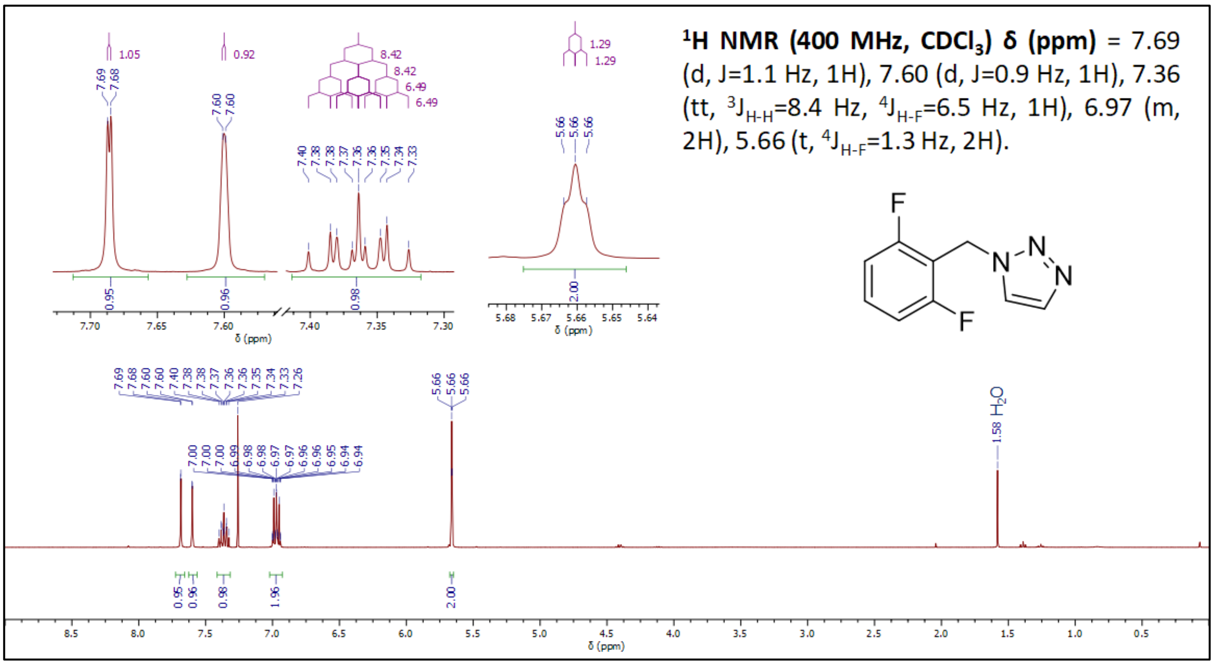

*Figure S 114. ¹H NMR spectrum of 1-(2,6-difluorobenzyl)-1H-1,2,3-triazole **3af**.*

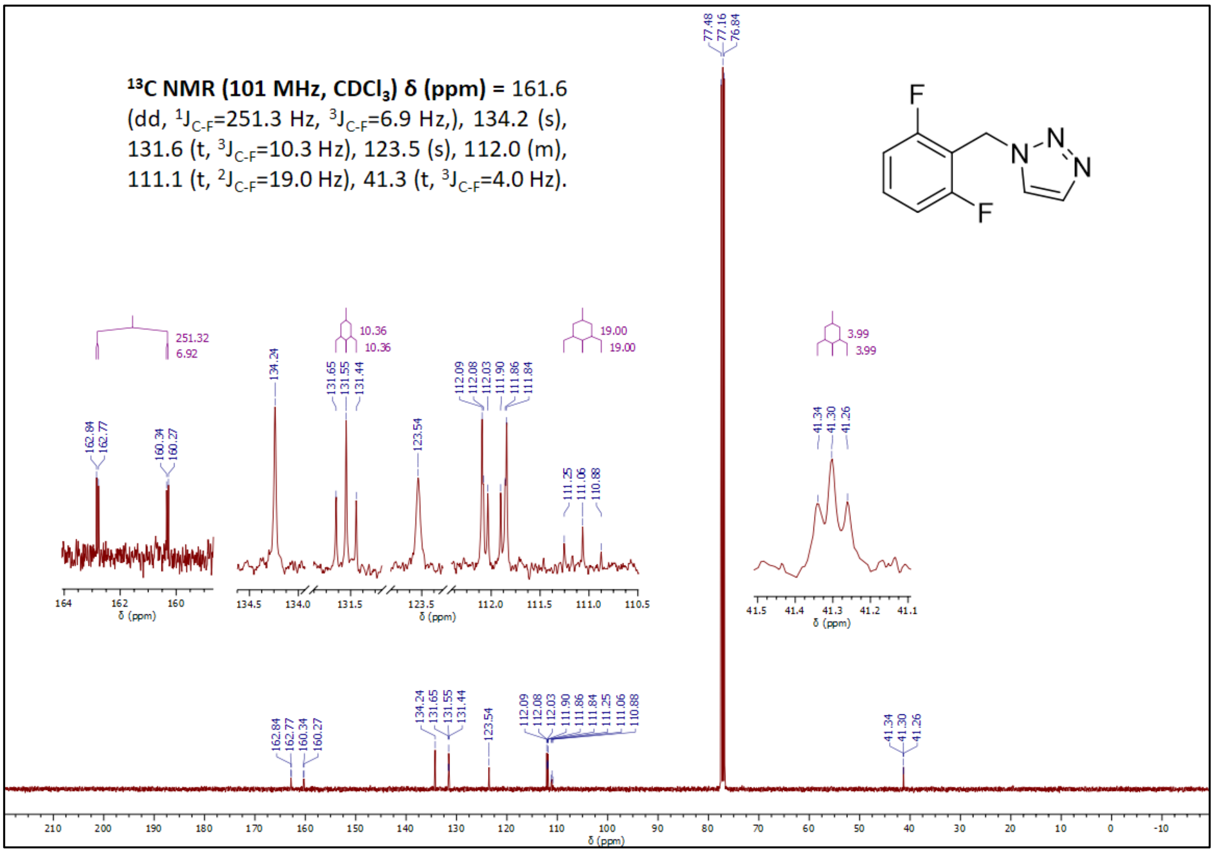

*Figure S 115. ¹³C NMR spectrum of 1-(2,6-difluorobenzyl)-1H-1,2,3-triazole **3af**.*

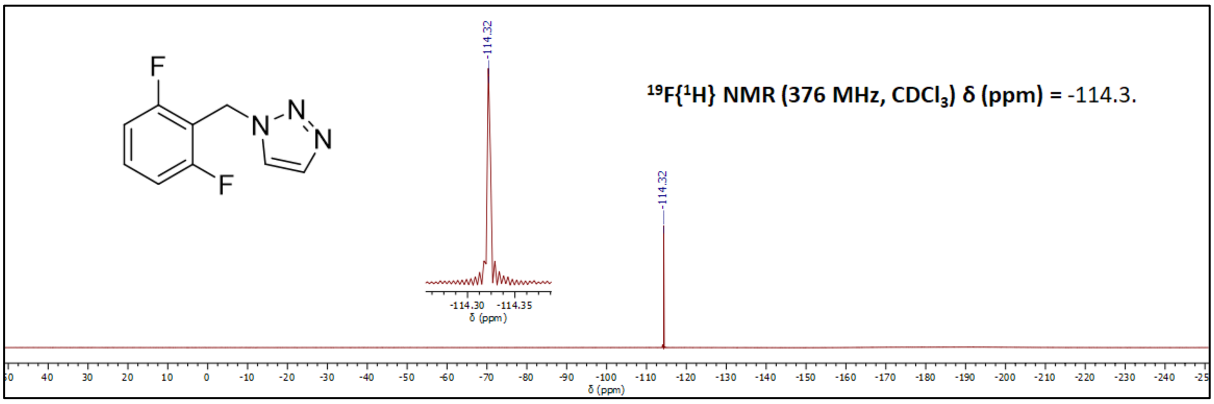

*Figure S 116. 19F{1H} NMR spectrum of 1-(2,6-difluorophenyl)-1H-1,2,3-triazole **3af**.*

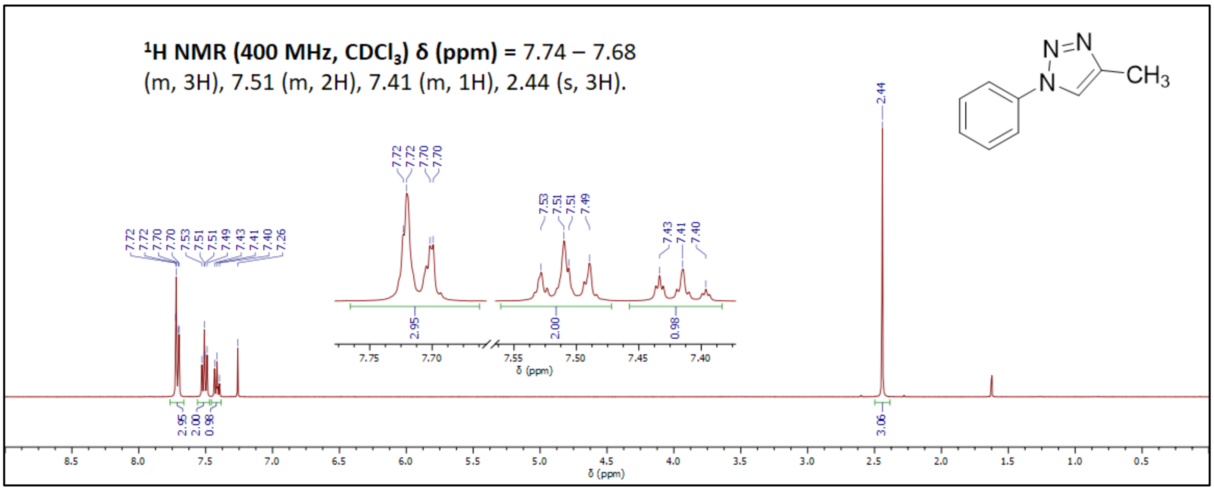

*Figure S 117. 1H NMR spectrum of 4-methyl-1-phenyl-1H-1,2,3-triazole **3af**.*

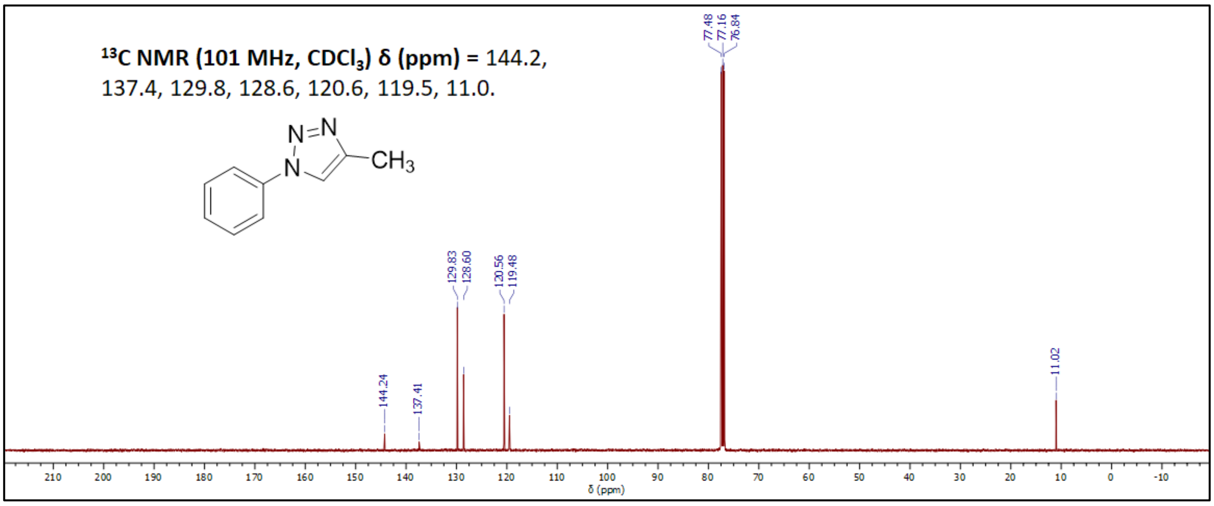

*Figure S 118. NMR spectrum of 4-methyl-1-phenyl-1H-1,2,3-triazole **3ag**.*

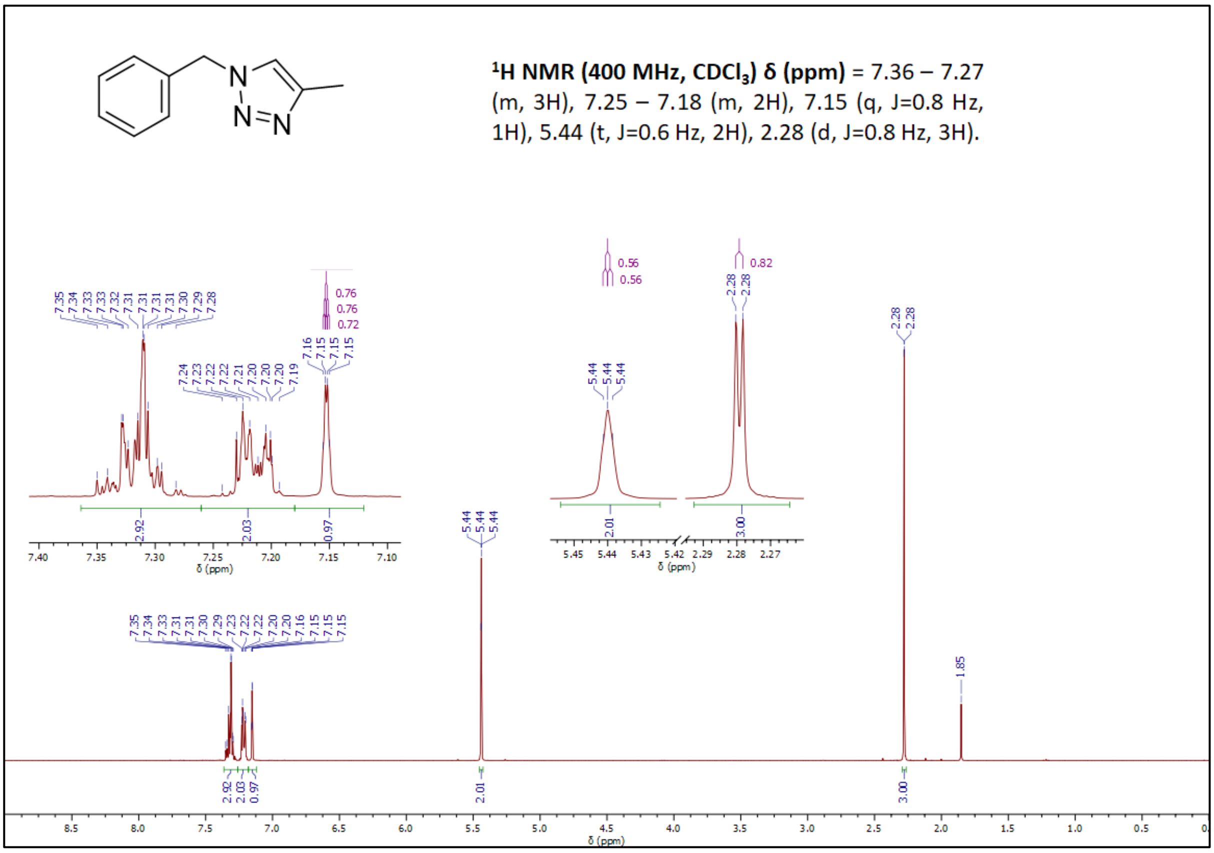

Figure S 119. ^{1}H NMR spectrum of 1-benzyl-4-methyl-1H-1,2,3-triazole ***3ah****.*

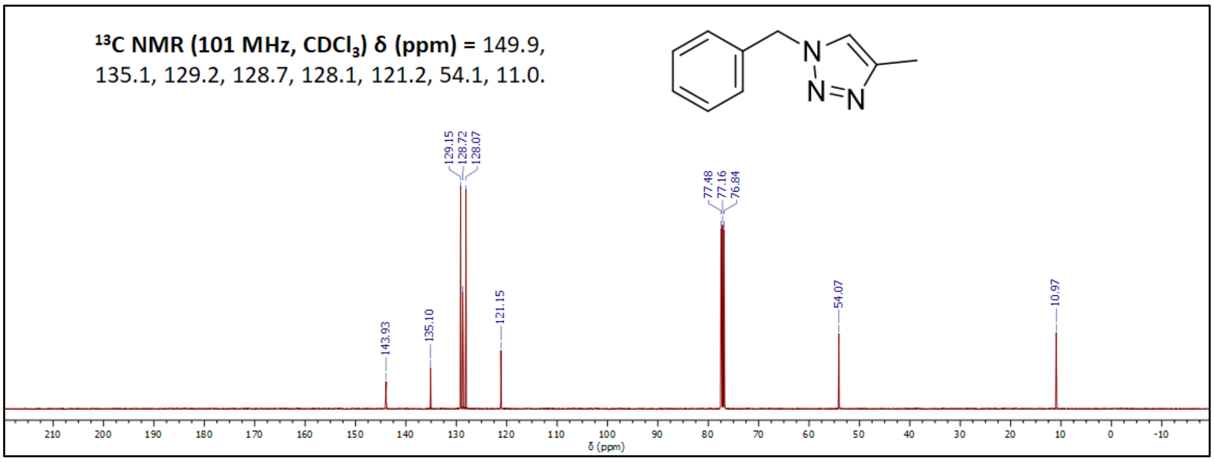

Figure S 120. ^{13}C NMR spectrum of 1-benzyl-4-methyl-1H-1,2,3-triazole ***3ah****.*

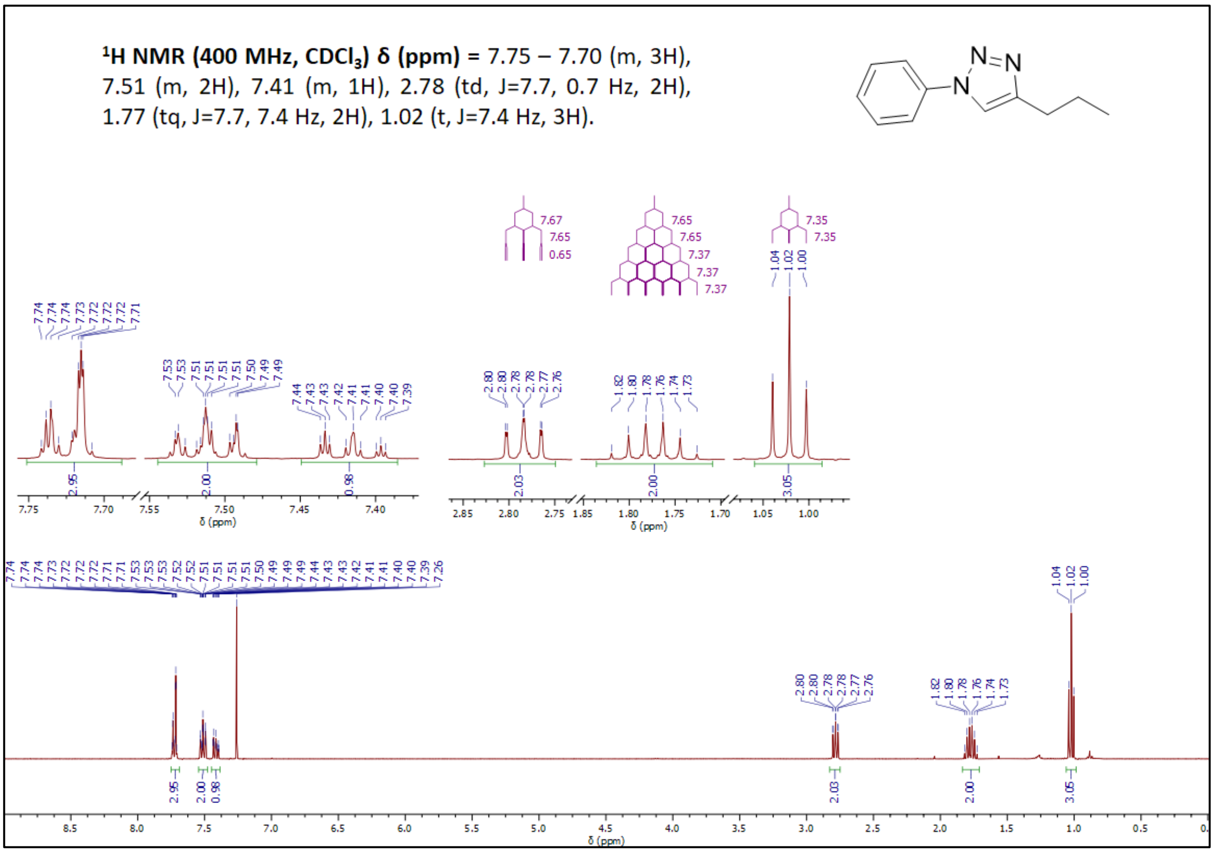

*Figure S 121. ^{1}H NMR spectrum of 1-phenyl-4-propyl-1H-1,2,3-triazole **3ai**.*

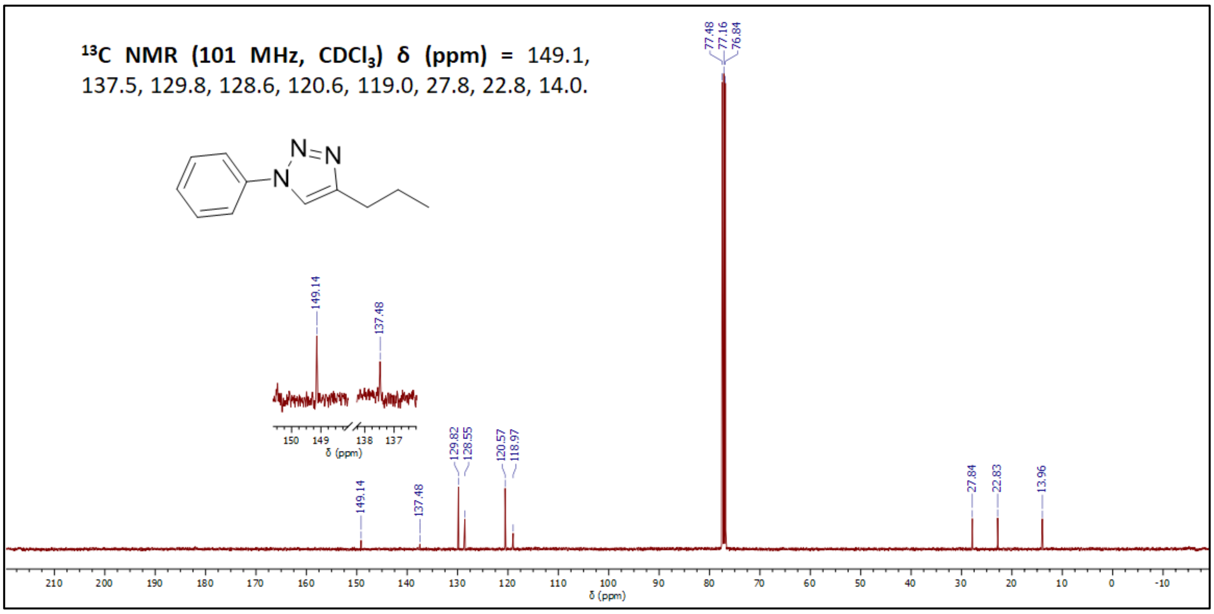

*Figure S 122.^{13}C NMR spectrum of 1-phenyl-4-propyl-1H-1,2,3-triazole **3ai**.*

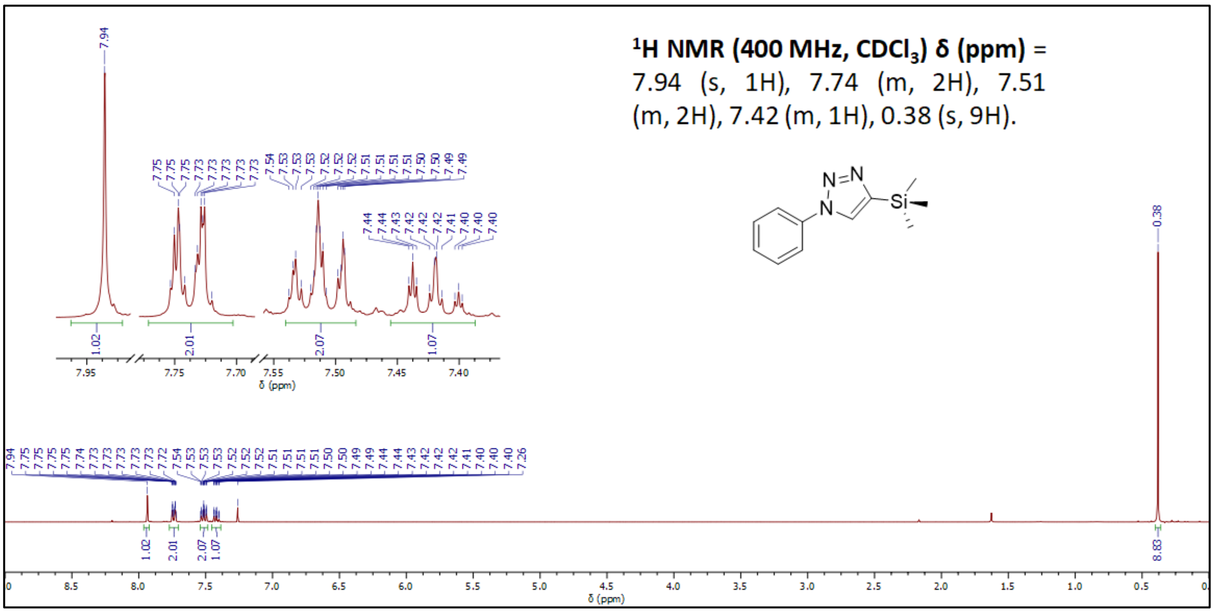

*Figure S 123. ^{1}H NMR spectrum of I 1-phenyl-4-(trimethylsilyl)-1H-1,2,3-triazole **3aj**.*

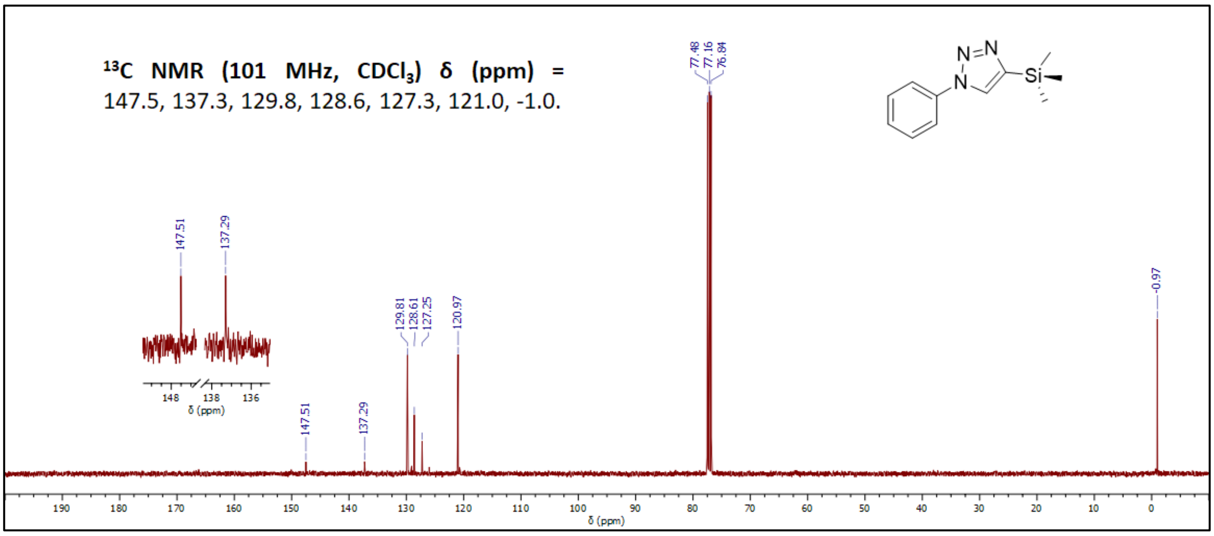

*Figure S 124. ^{13}C NMR spectrum of 1-phenyl-4-(trimethylsilyl)-1H-1,2,3-triazole **3aj**.*

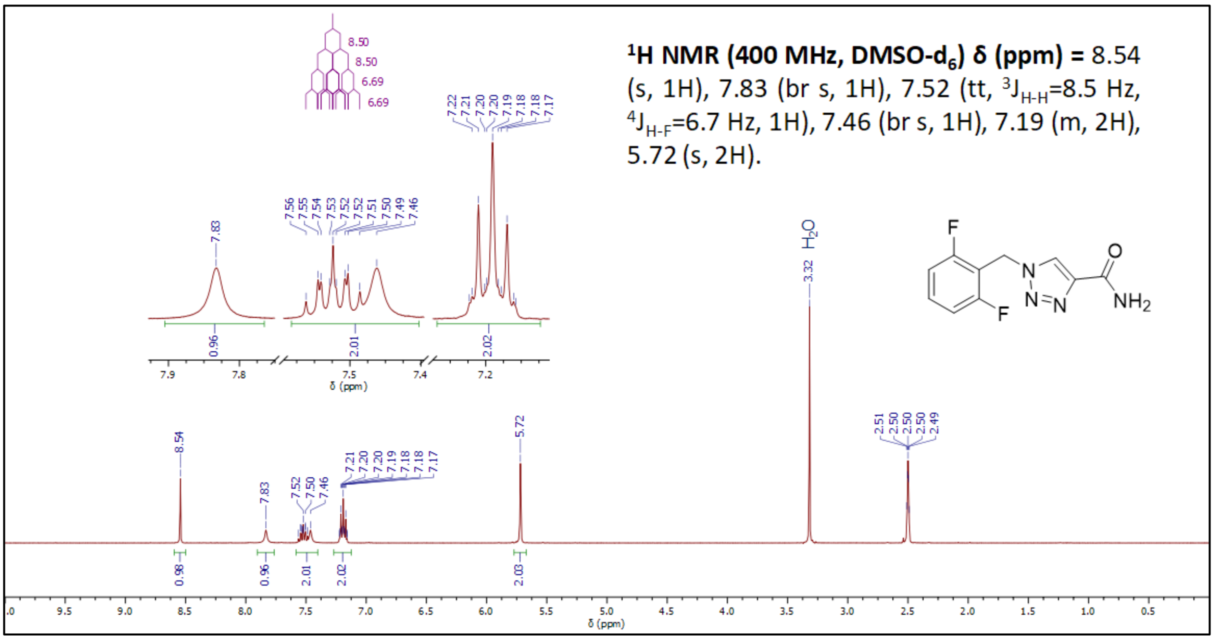

Figure S 125. ^{1}H NMR spectrum of 1-(2,6-difluorobenzyl)-1H-1,2,3-triazole-4-carboxamide (rufinamide) ***4****.*

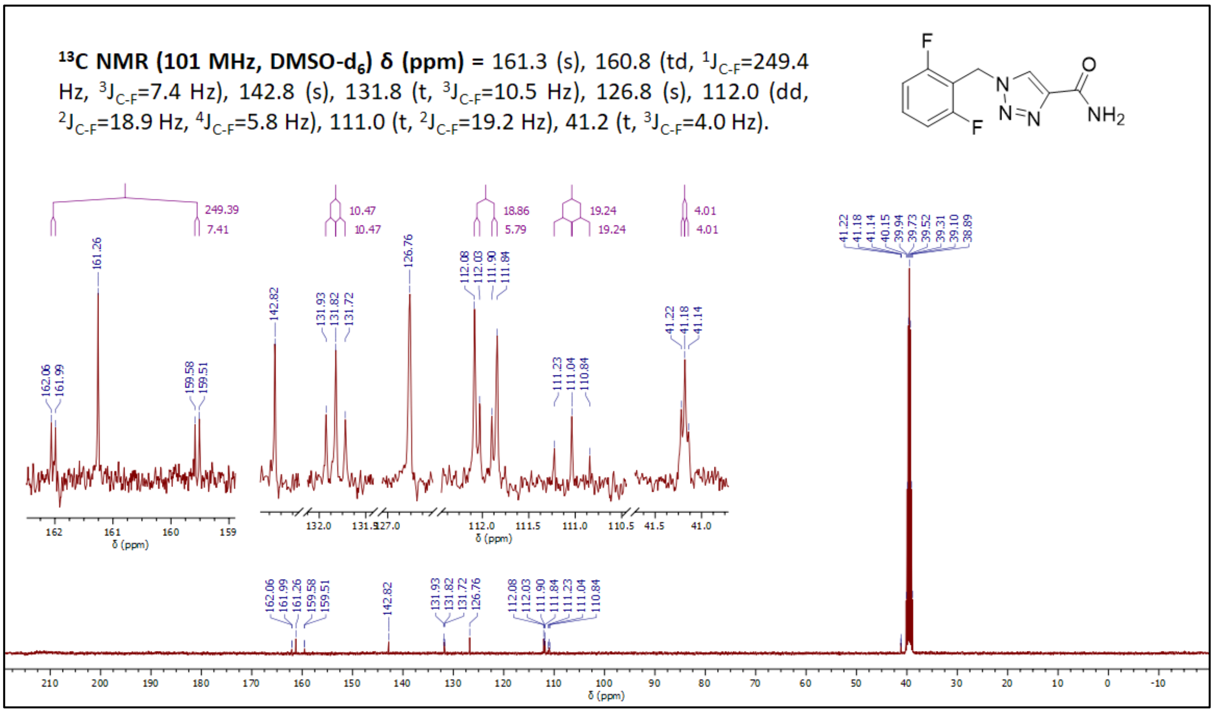

Figure S 126. ^{13}C NMR spectrum of 1-(2,6-difluorobenzyl)-1H-1,2,3-triazole-4-carboxamide (rufinamide) ***4****.*

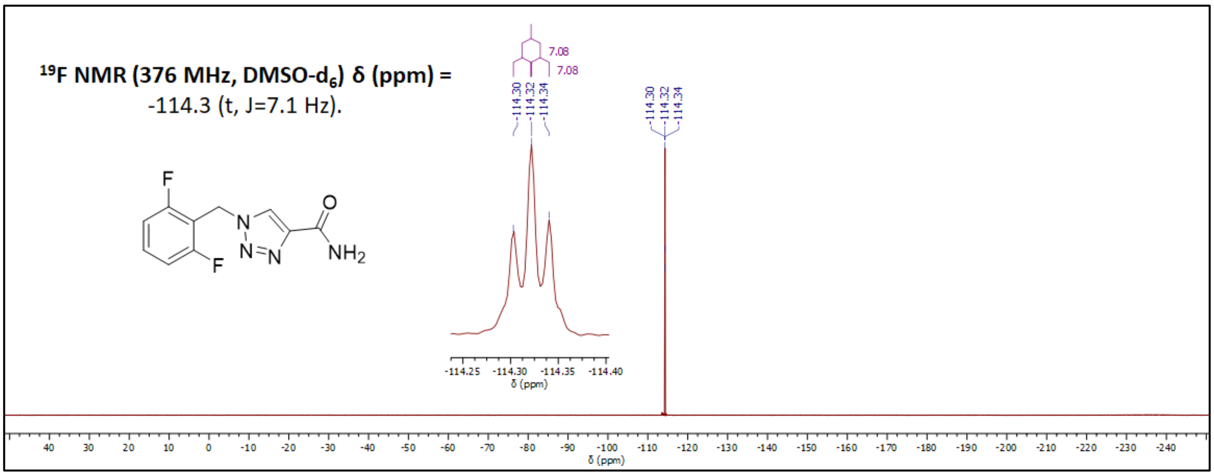

Figure S 127.[19]*F NMR spectrum of 1-(2,6-difluorobenzyl)-1H-1,2,3-triazole-4-carboxamide (rufinamide)* ***4****.*

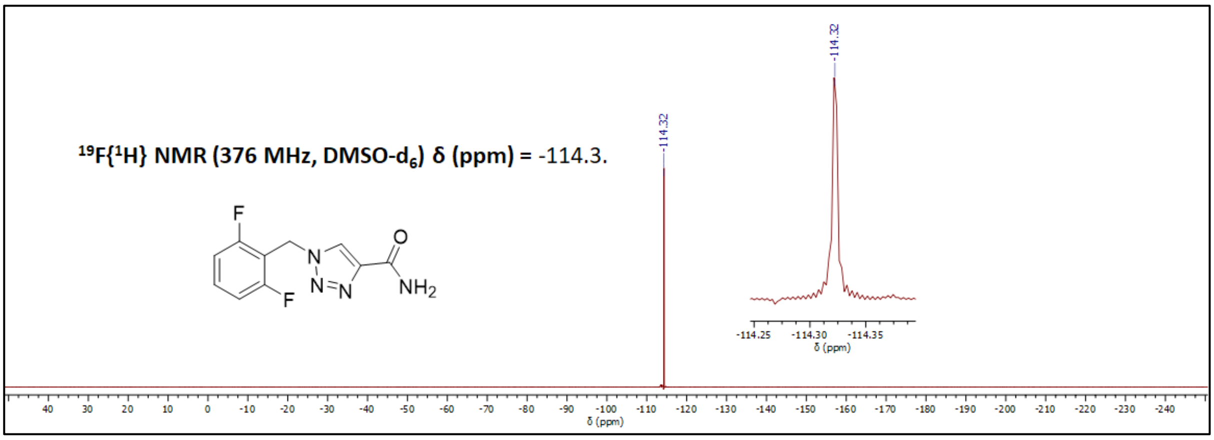

Figure S 128. ^{19}*F{*^{1}*H} NMR spectrum of 1-(2,6-difluorobenzyl)-1H-1,2,3-triazole-4-carboxamide (rufinamide)* ***4****.*

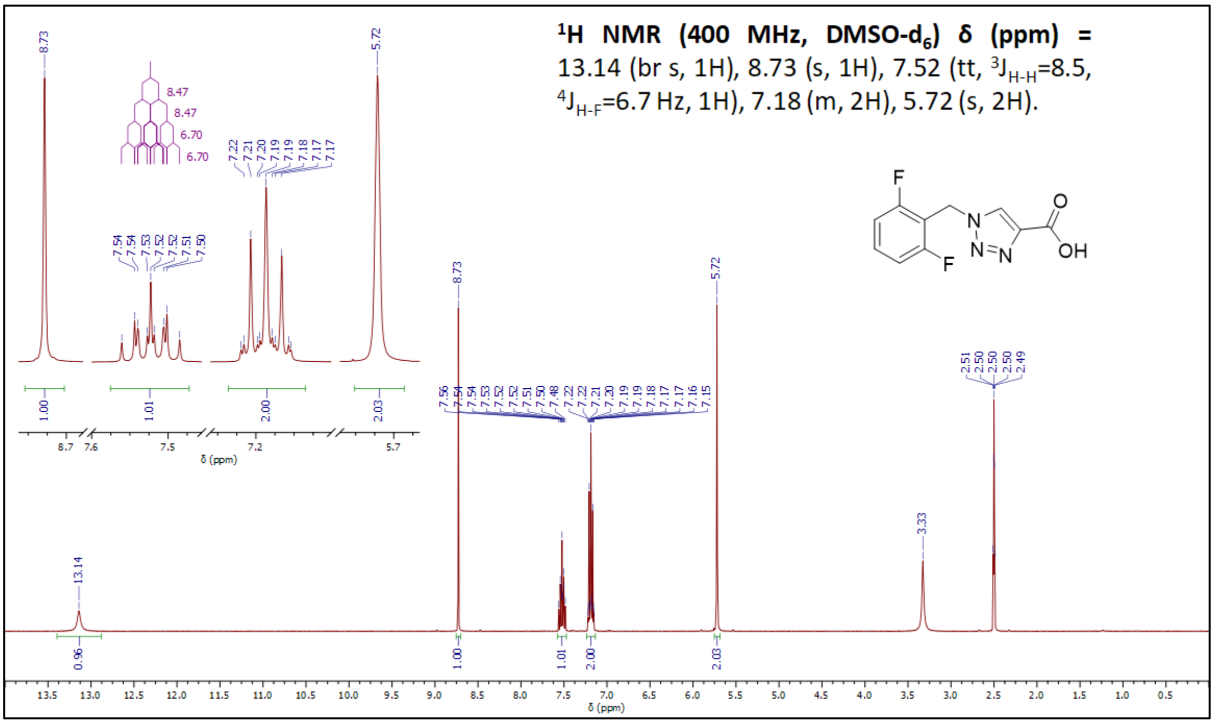

Figure S 129. ^{1}*H NMR spectrum of 1-(2,6-difluorobenzyl)-1H-1,2,3-triazole-4-carboxylic acid.*

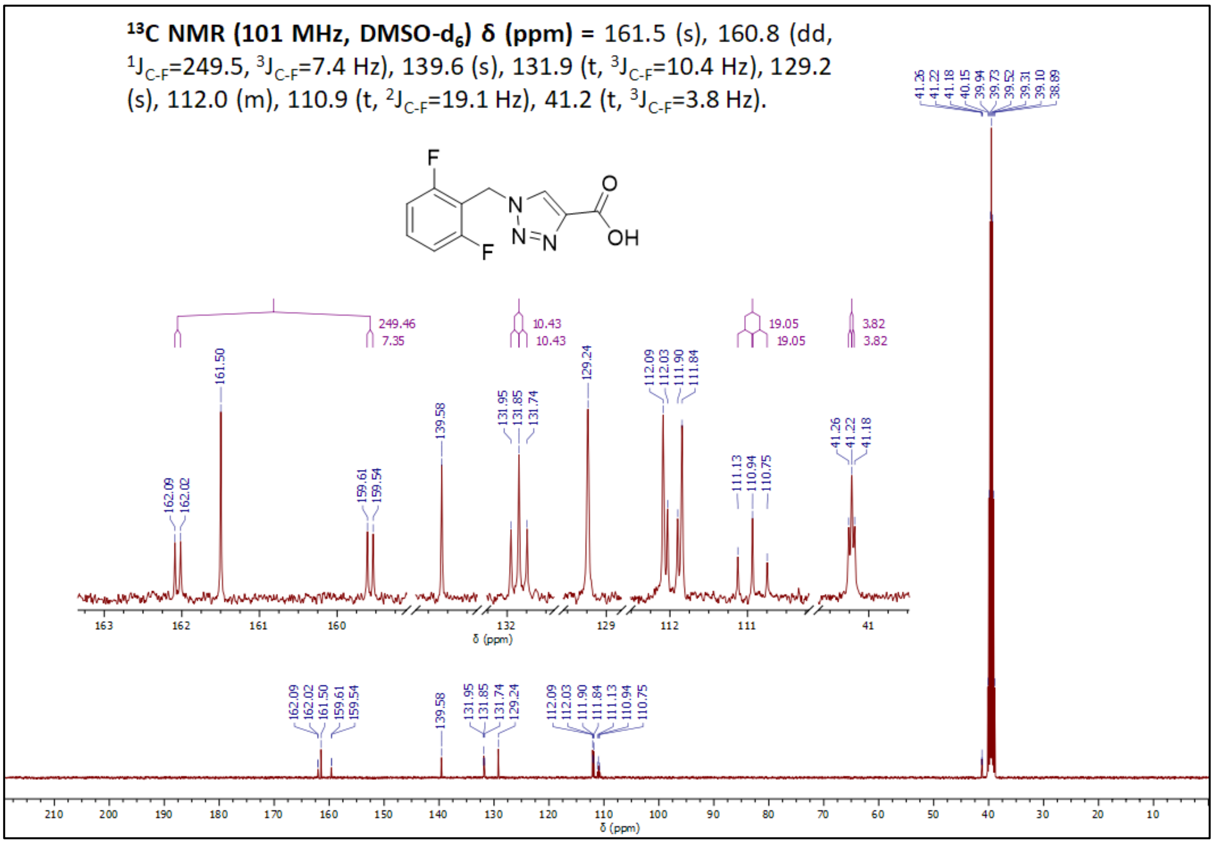

Figure S 130. ^{13}C NMR spectrum of 1-(2,6-difluorobenzyl)-1H-1,2,3-triazole-4-carboxylic acid.

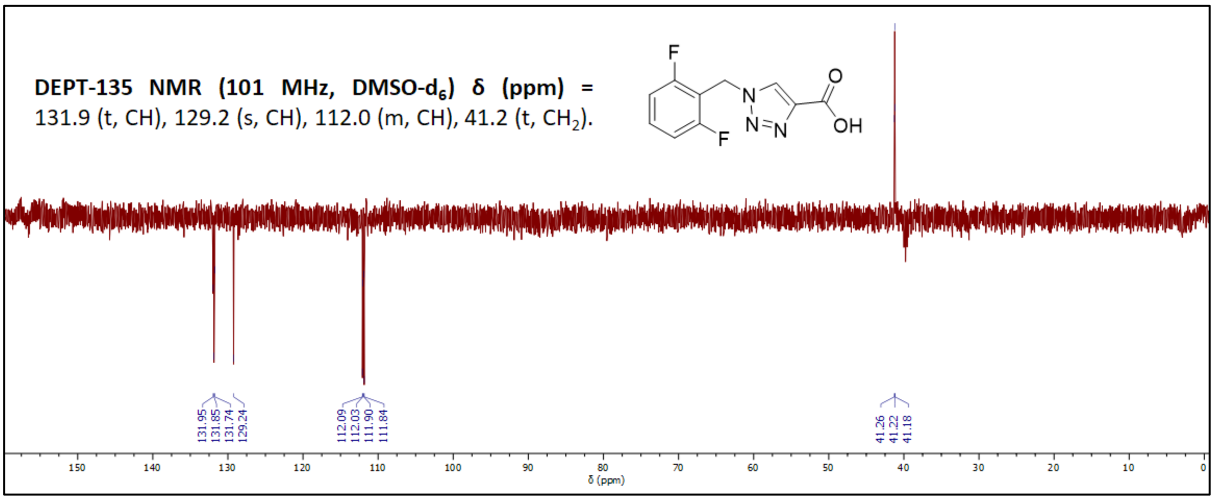

Figure S 131. DEPT-135 NMR spectrum of 1-(2,6-difluorobenzyl)-1H-1,2,3-triazole-4-carboxylic acid.

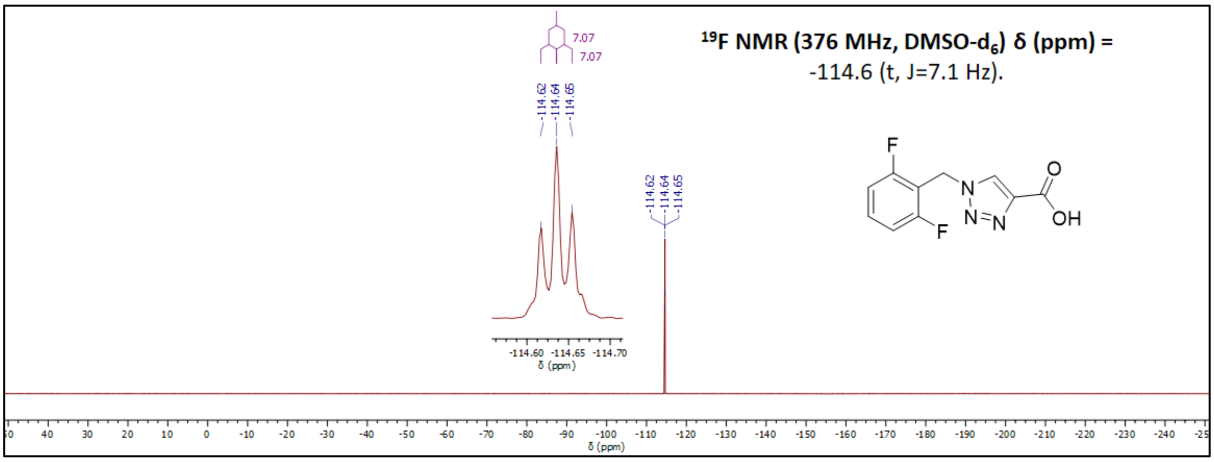

Figure S 132. 19F NMR spectrum of 1-(2,6-difluorobenzyl)-1H-1,2,3-triazole-4-carboxylic acid.

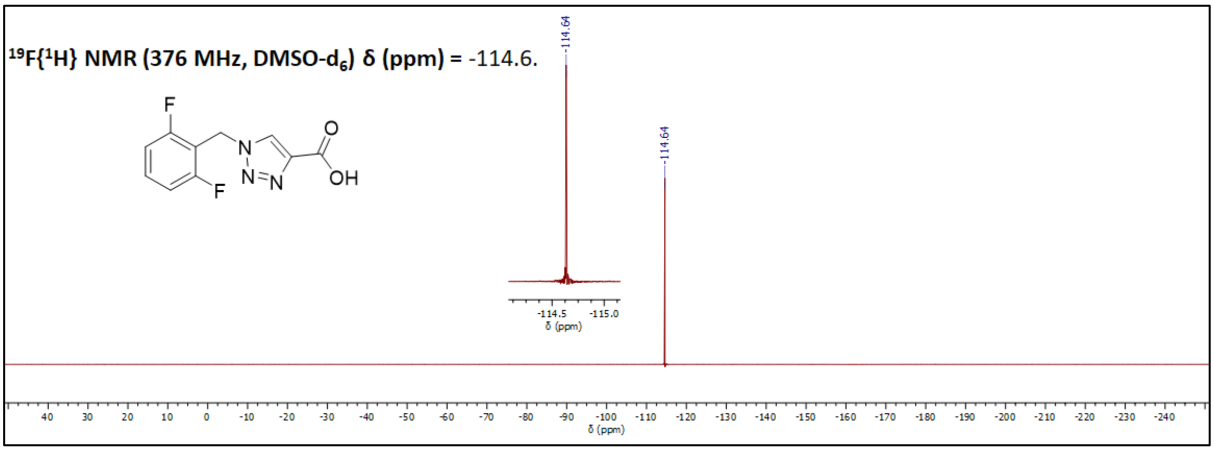

Figure S 133. 19F{1H} NMR spectrum of 1-(2,6-difluorobenzyl)-1H-1,2,3-triazole-4-carboxylic acid.

6. References

1 M.-A. Armour, *Hazardous Laboratory Chemicals Disposal Guide*, CRC Press, 1996.

2 M. T. Berry, D. Castrejon and J. E. Hein, *Org. Lett.*, 2014, **16**, 3676–3679.

3 S. Bai, S. Li, J. Xu, X. Peng, K. Sai, W. Chu, Z. Tu, C. Zeng and R. H. Mach, *J. Med. Chem.*, 2014, **57**, 4239–4251.

4 L. Ren and N. Jiao, *Chem. Commun.*, 2014, **50**, 3706–3709.

5 M. Li, K. Dong, Y. Zheng and W. Song, *Org. Biomol. Chem.*, 2019, **17**, 9933–9941.

6 C. Qin, T. Shen, C. Tang and N. Jiao, *Angew. Chemie - Int. Ed.*, 2012, **51**, 6971–6975.

7 P. Couvreur, J. Nicolas, T. Blin, A. Chtourou and N. Genevaz, in *US Patent, US 2017/0079928A1*, 2014.

8 D. Yadav, N. Singh, T. W. Kim, J. Y. Kim, N. J. Park and J. O. Baeg, *Green Chem.*, 2019, **21**, 2677–2685.

9 C. Z. Tao, X. Cui, J. Li, A. X. Liu, L. Liu and Q. X. Guo, *Tetrahedron Lett.*, 2007, **48**, 3525–3529.

10 K. Takagi, H. Fukuda, S. Shuto, A. Otaka and M. Arisawa, *Adv. Synth. Catal.*, 2015, **357**, 2119–2124.

11 J.-H. H. Li, X.-D. D. Zhang and Y.-X. X. Xie, *Synthesis (Stuttg).*, 2005, **2005**, 804–808.

12 J. Andersen, S. Bolvig and X. Liang, *Synlett*, 2005, **2005**, 2941–2947.

13 R. Karmakar, S. Y. Yun, J. Chen, Y. Xia and D. Lee, *Angew. Chemie - Int. Ed.*, 2015, **54**, 6582–6586.

14 R. Zeng, L. Chen and Q. Yan, *Angew. Chemie - Int. Ed.*, 2020, **59**, 18418–18422.

15 M. W. Amolins, L. B. Peterson and B. S. J. Blagg, *Bioorganic Med. Chem.*, 2009, **17**, 360–367.

16 A. Schulz, M. Thomas and A. Villinger, *Dalt. Trans.*, 2019, **48**, 125–132.

17 W. A. Carroll, A. Perez-Medrano, A. S. Florjancic, D. W. Nelson, S. Peddi, T. Li, E. M. Bunnelle, G. C. Hirst and B. Li, *WO2005111003A1*, , DOI:WO2005111003A1.

18 D. Hédou, E. Deau, C. Dubouilh-Benard, M. Sanselme, A. Martinet, E. Chosson, V. Levacher and T. Besson, *European J. Org. Chem.*, 2013, 7533–7545.

19 J. J. Yang, W. W. Yu, L. L. Hu, W. J. Liu, X. H. Lin, W. Wang, Q. Zhang, P. L. Wang, S. W. Tang, X. Wang, M. Liu, W. Lu and H. K. Zhang, *J. Med. Chem.*, 2020, **63**, 569–590.

20 Y. Wu, K. Chen, X. Ge, P. Ma, Z. Xu, H. Lu and G. Li, *Org. Lett.*, 2020, **22**, 6143–6149.

21 B. H. Lipshutz and B. R. Taft, *Angew. Chemie Int. Ed.*, 2006, **45**, 8235–8238.

22 M. A. Topchiy, A. A. Ageshina, P. S. Gribanov, S. M. Masoud, T. R. Akmalov, S. E. Nefedov, S. N. Osipov, M. S. Nechaev and A. F. Asachenko, *European J. Org. Chem.*, 2019, **2019**, 1016–1020.

23 R. S. Chauhan, D. Oza, S. Yadav, C. Dash, A. M. Z. Slawin and N. Shivran, *New J. Chem.*, 2019, **43**, 2381–2388.

24 Y. Zhao, H. Van Nguyen, L. Male, P. Craven, B. R. Buckley and J. S. Fossey, *Organometallics*, 2018, **37**, 4224–4241.

25 E. Loukopoulos, A. Abdul-Sada, G. Csire, C. Kállay, A. Brookfield, G. J. Tizzard, S. J. Coles, I. N. Lykakis and G. E. Kostakis, *Dalt. Trans.*, 2018, **47**, 10491–10508.

26 H. Ben El Ayouchia, H. ElMouli, L. Bahsis, H. Anane, R. Laamari, C. J. Gómez-García, M. Julve and S. E. Stiriba, *J. Organomet. Chem.*, 2019, **898**, 120881.

27 J. Yan and L. Wang, *Synthesis (Stuttg).*, 2009, **2010**, 447–452.

28 A. A. Ali, M. Chetia, B. Saikia, P. J. Saikia and D. Sarma, *Tetrahedron Lett.*, 2015, **56**, 5892–5895.

29 C. Shao, R. Zhu, S. Luo, Q. Zhang, X. Wang and Y. Hu, *Tetrahedron Lett.*, 2011, **52**, 3782–3785.

30 S. Guo, Y. Zhou, B. Dai, C. Huo, C. Liu and Y. Zhao, *Synth.*, 2018, **50**, 2191–2199.

31 X. Xiong and L. Cai, *Catal. Sci. Technol.*, 2013, **3**, 1301–1307.

32 Z. Jin, P. Xue and E. Fu, *Synth. Commun.*, 2014, **44**, 68–75.

33 J. Q. Shang, H. Fu, Y. Li, T. Yang, C. Gao and Y. M. Li, *Tetrahedron*, 2019, **75**, 253–259.

34 H. Xu and Z. Sun, *Adv. Synth. Catal.*, 2016, **358**, 1736–1740.

35 P. Kautny, D. Bader, B. Stöger, G. A. Reider, J. Fröhlich and D. Lumpi, *Chem. - A Eur. J.*, 2016, **22**, 18887–18898.

36 P. Li, Y. Liu, L. Wang, J. Xiao and M. Tao, *Adv. Synth. Catal.*, 2018, **360**, 1673–1684.

37 V. B. Purohit, S. C. Karad, K. H. Patel and D. K. Raval, *RSC Adv.*, 2014, **4**, 46002–46007.

38 P. V. Chavan, K. S. Pandit, U. V. Desai, M. A. Kulkarni and P. P. Wadgaonkar, *RSC Adv.*, 2014, **4**, 42137–42146.

39 Z. G. Wu, X. J. Liao, L. Yuan, Y. Wang, Y. X. Zheng, J. L. Zuo and Y. Pan, *Chem. - A Eur. J.*, 2020, **26**, 5694–5700.

40 B. E. Velasco, G. López-Téllez, N. González-Rivas, I. García-Orozco and E. Cuevas-Yañez, *Can. J. Chem.*, 2013, **91**, 292–299.

41 M. González-Lainez, M. Gallegos, J. Munarriz, R. Azpiroz, V. Passarelli, M. V. Jiménez and J. J. Pérez-Torrente, *Organometallics*, 2022, **41**, 2154–2169.

42 M. M. Khakzad Siuki, M. Bakavoli and H. Eshghi, *Appl. Organomet. Chem.*, 2019, **33**, e4774.

43 S. Ito, A. Kakehi, K. Okada, S. Goto and H. Kawaguchi, *Heterocycles*, 1997, **45**, 889–896.

44 H. W. Bai, Z. J. Cai, S. Y. Wang and S. J. Ji, *Org. Lett.*, 2015, **17**, 2898–2901.

45 J. P. Williams, D. Slee, M. Moorjani and N. J. Ashweek, in *International Patent, WO2016123533A1*, 2016.

46 P. Wigerinck, A. Van Aerschot, P. Claes, J. Balzarini, E. De Clercq and P. Herdewijn, *J. Heterocycl. Chem.*, 1989, **26**, 1635–1642.

47 L. Garlatti, R. Huet and K. Alvarez, *Tetrahedron*, 2021, **92**, 132252.

48 K. Farrell, H. Müller-Bunz and M. Albrecht, *Dalt. Trans.*, 2016, **45**, 15859–15871.

49 K. Dabak, Ö. Sezer, A. Akar and O. Anaç, *Eur. J. Med. Chem.*, 2003, **38**, 215–218.

50 M. K. Barman, A. K. Sinha and S. Nembenna, *Green Chem.*, 2016, **18**, 2534–2541.

51 A. M. Deobald, L. R. S. Camargo, D. Alves, J. Zukerman-Schpector, A. G. Corrêa and M. W. Paixão, *Synthesis (Stuttg).*, 2011, **2011**, 4003–4010.

52 S. J. M. Patterson, P. R. Clark, G. D. Williams and N. C. O. Tomkinson, *Tetrahedron Lett.*, 2020, **61**, 152483.

53 Z. Zhang and C. Kuang, *Chinese J. Chem.*, 2013, **31**, 1011–1014.

54 M. E. Munk and Y. K. Kim, *J. Am. Chem. Soc.*, 1964, **86**, 2213–2217.

55 J. Soellner, I. Císařová and T. Strassner, *Organometallics*, 2018, **37**, 4619–4629.

56 F. Cuevas, A. I. Oliva and M. A. Pericàs, *Synlett*, 2010, **2010**, 1873–1877.

57 B. Kaboudin, Y. Abedi and T. Yokomatsu, *Org. Biomol. Chem.*, 2012, **10**, 4543–4548.

58 T. Sakamoto, D. Uchiyama, Y. Kondo and H. Yamanaka, *Heterocycles*, 1993, **35**, 1273–1278.

59 P. Eisenberger, B. P. Bestvater, E. C. Keske and C. M. Crudden, *Angew. Chemie - Int. Ed.*, 2015, **54**, 2467–2471.

60 O. Jennah, R. Beniazza, C. Lozach, D. Jardel, F. Molton, C. Duboc, T. Buffeteau, A. El Kadib, D. Lastécouères, M. Lahcini and J. M. Vincent, *Adv. Synth. Catal.*, 2018, **360**, 4615–4624.

61 W. H. Mudd and E. P. Stevens, *Tetrahedron Lett.*, 2010, **51**, 3229–3231.
